# Supplementary material for: The Escherichia coli transcriptome mostly consists of independently regulated modules
Source: Nat Commun. 2019 Dec 4;10:5536. doi: 10.1038/s41467-019-13483-w (PMC6892915; doi:10.1038/s41467-019-13483-w)
Supplement: Supplementary file 1 — Supplementary Information [file 41467_2019_13483_MOESM1_ESM.pdf]

Supplementary Information

The *Escherichia coli* Transcriptome Mostly Consists of Independently Regulated Modules

Authors: Anand V. Sastry, Ye Gao, Richard Szubin, Ying Hefner, Sibei Xu, Donghyuk Kim, Kumari Sonal Choudhary, Laurence Yang, Zachary A. King, Bernhard O. Palsson

Table of Contents:

Supplementary Figures *2*

Supplementary Figure 1: Summary of data 2

Supplementary Figure 2: Overview of i-modulons 3

Supplementary Figure 3: Validation of i-modulon-regulator relationships 4

Supplementary Figure 4: Additional properties of i-modulons 5

Supplementary Figure 5: Significance thresholds for components and differential activity 6

Supplementary Figure 6: Descriptive characteristics of the EvgA i-modulon with explanations 7

Supplementary Notes *8*

Supplementary Note 1: Overview of i-modulons 8

Supplementary Note 2: Explanation of ALE-derived i-modulons 21

Supplementary Methods *22*

Automated characterization of i-modulons 22

Motif Search 23

Supplementary Tables *24*

Supplementary Table 1: Overview of i-modulons derived from PRECISE 24

Supplementary Table 2: ChIP-exo binding sites for MetJ 27

Supplementary Table 3: ChIP-exo binding sites for CysB 28

Supplementary Table 4: Computationally-detected novel TF binding sites upstream of i-modulon genes 30

Supplementary Table 5: I-modulon membership for uncharacterized genes. 34

Supplementary Table 6: Mutated regulators in evolved strains 38

Supplementary Table 7: Genes in the BW25113 i-modulon 39

Supplementary Table 8: Primers used in this study 40

Supplementary References *41*

Supplementary Figures

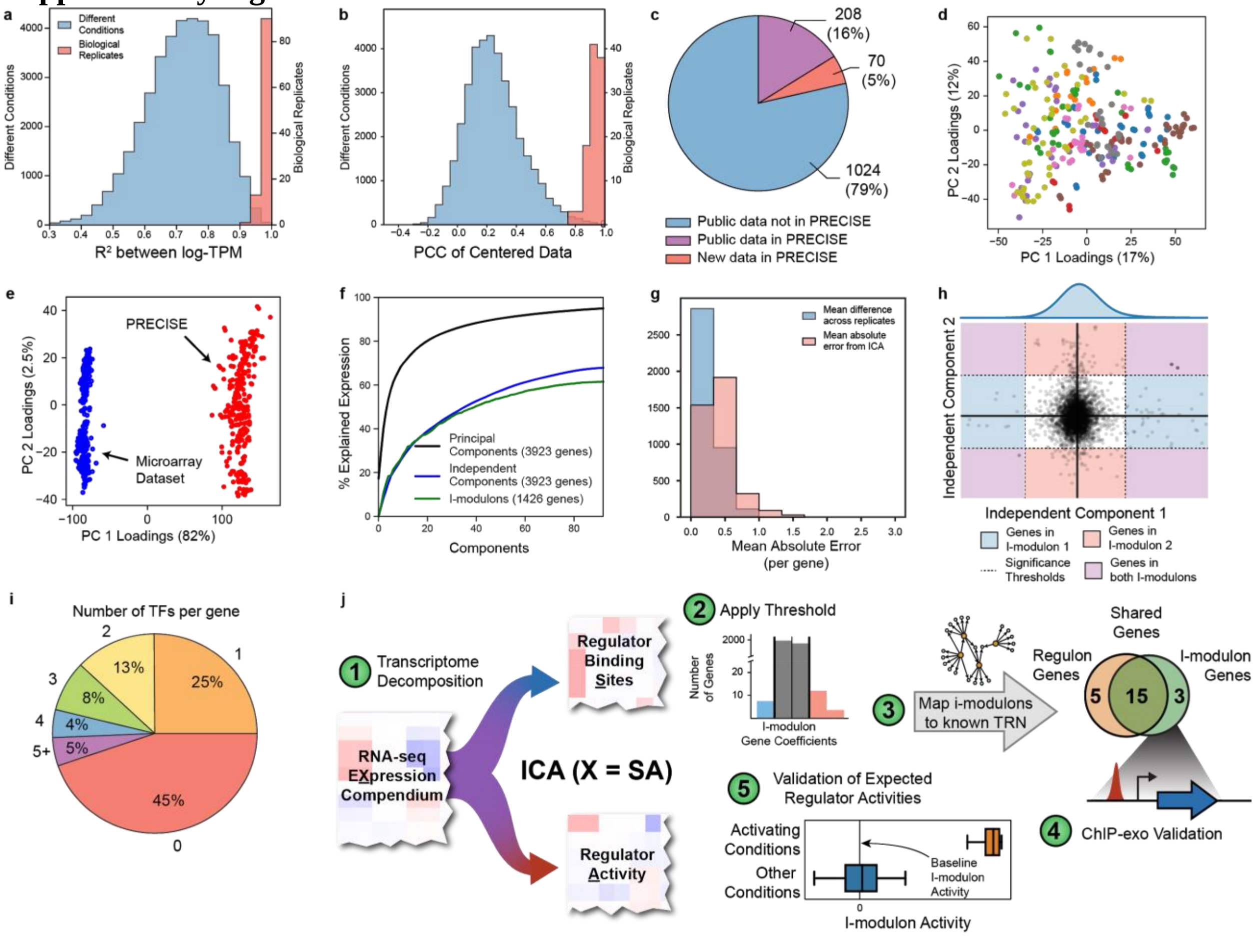

Supplementary Figure 1: Summary of data

(a) Histogram of data quality, as measured by the coefficient of determination (R^2) between log-TPM. Comparison between biological replicates are shown in red, whereas comparison between all pairwise non-replicates are shown in blue. (b) Histogram of the Pearson correlation coefficient (PCC) between log-TPM, after centering via subtraction of a reference condition (See Methods). (c) Pie chart of all published RNA-seq data for *E. coli* MG1655 or BW25113 on NCBI GEO as of January 2019. (d) Loadings of the first two principal components (PC) of PRECISE, colored by the researcher who created the dataset. The high overlap of various colors demonstrates the self-consistency of the compendium. (e) Loadings of the first two principal component of PRECISE merged with a microarray dataset[1], illustrating the clear batch effects introduced by integrating data from different sources. (f) Cumulative explained variance for the first 92 components calculated by principal component analysis (black) and ICA (blue). Cumulative explained variance using only significant genes in each i-modulon is shown in green. (g) Histogram of mean absolute difference between log-TPM values in the original expression matrix compared to mean absolute error between the original expression matrix and the reconstructed matrix (**X**-**SA**) (h) Scatterplot of two independent components, illustrating statistical independence. Given any value of independent component 2, the distribution of values across independent component 1 will be nearly identical to the distribution displayed above the plot. Thus, the coefficient of a gene in independent component 1 does not depend on the coefficient of the gene in independent component 2. The plot also illustrates that a gene may be significant in multiple components, and therefore a member of multiple i-modulons. (i) Distribution of TF connectivity in *Escherichia coli* using interactions from all confidence levels in RegulonDB v10.0[2]. The pie chart indicates the fraction of genes that are regulated by the indicated number of TFs. (j) Schematic illustration of data processing pipeline. Validation steps (4 and 5) are discussed in the results section "Validation of I-modulon-Regulator Relationships".

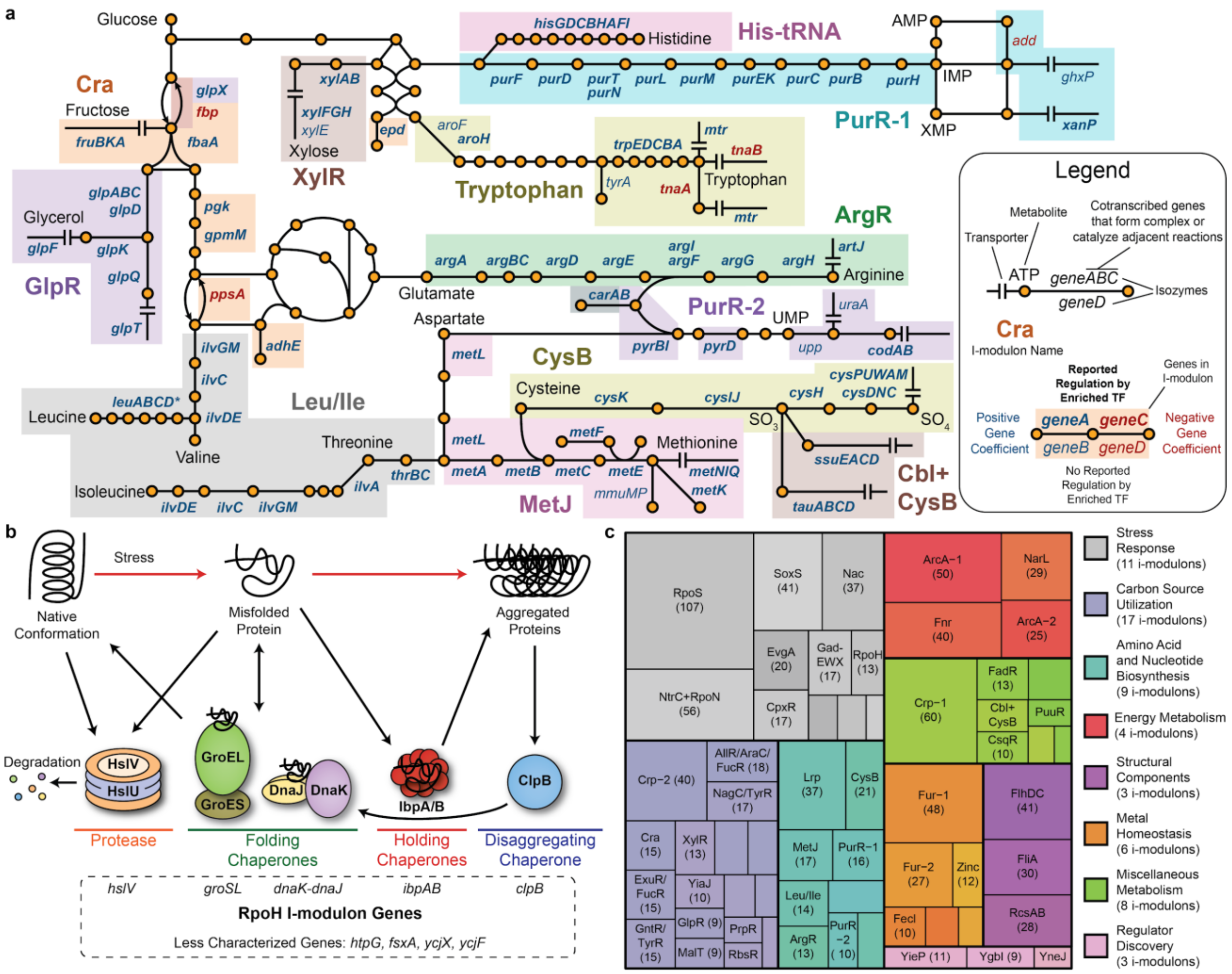

Supplementary Figure 2: Overview of i-modulons

(a) Schematic illustration of metabolic pathways captured by 12 regulatory i-modulons. Gene names within a colored box are members of the corresponding i-modulon. Co-transcribed genes whose products form a complex or catalyze adjacent reactions share gene names (e.g., trpEDCBA), and isozymes (i.e., enzymes that catalyze identical reactions) share reaction lines (e.g., glpABC/glpD). Genes with positive i-modulon weights are colored blue, and genes with negative weights are red. Bold font indicates that the genes belong to the associated regulon. (b) Simplified schematic illustration of the protein misfolding response in *E. coli* emphasizing genes in the RpoH i-modulon. Adapted from Baneyx and Mujacic, 2004[3]. (c) Illustration of the 61 regulatory i-modulons, colored by functional category. Number of genes in each i-modulon are indicated by the size of the box.

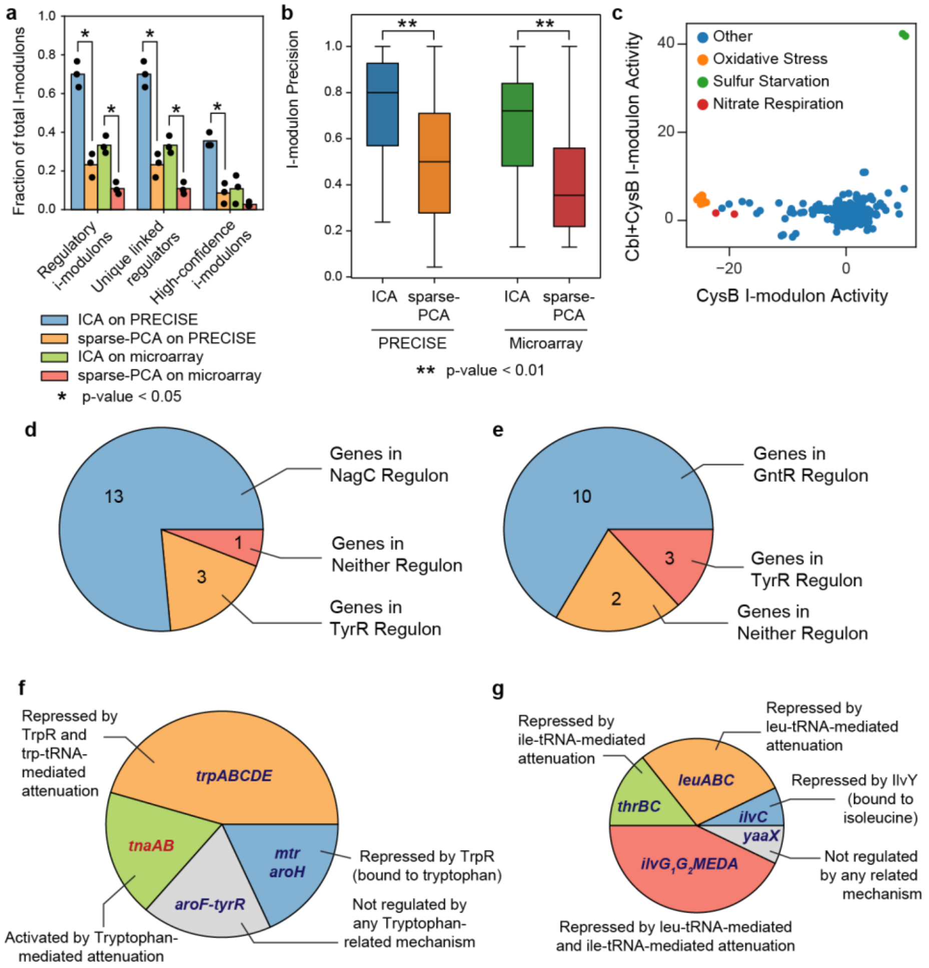

Supplementary Figure 3: Validation of i-modulon-regulator relationships

(a) Evaluation of ICA and sparse-PCA on PRECISE and a microarray dataset (NCBI GEO GSE6836)[1]. ICA consistently outperforms sparse-PCA (p-value < .05) in number of regulatory i-modulons, unique linked regulators, and high-confidence i-modulons (precision > 0.5, recall > 0.25). (b) The precision of ICA-derived i-modulons are higher than sparse-PCA-derived i-modulons for both datasets (p-value < .01, Mann-Whitney U test). Boxplot whiskers represent extrema of data, box bounds represent upper and lower quartiles, and center-line represents the median value. (c) Scatterplot of CysB i-modulon activities against Cbl+CysB i-modulon activities. The Cbl+CysB i-modulon was only active under sulfur starvation conditions. (d) Distribution of gene regulation in the combined NagC/TyrR i-modulon. (e) Distribution of gene regulation in the combined GntR/TyrR i-modulon. (f) Distribution of tryptophan-related regulatory mechanisms in the TrpR i-modulon. Gene names in blue indicate positive gene coefficients, and gene names in red indicate negative gene coefficients. (g) Distribution of leucine- and isoleucine-related regulatory mechanisms in the Leu/Ile i-modulon. High concentrations of leucine or isoleucine either charge tRNAs that pause transcription to form termination loops, or activate the transcription factor IlvY to repress transcription of *ilvC*. Subscripts indicate pseudogene fragments.

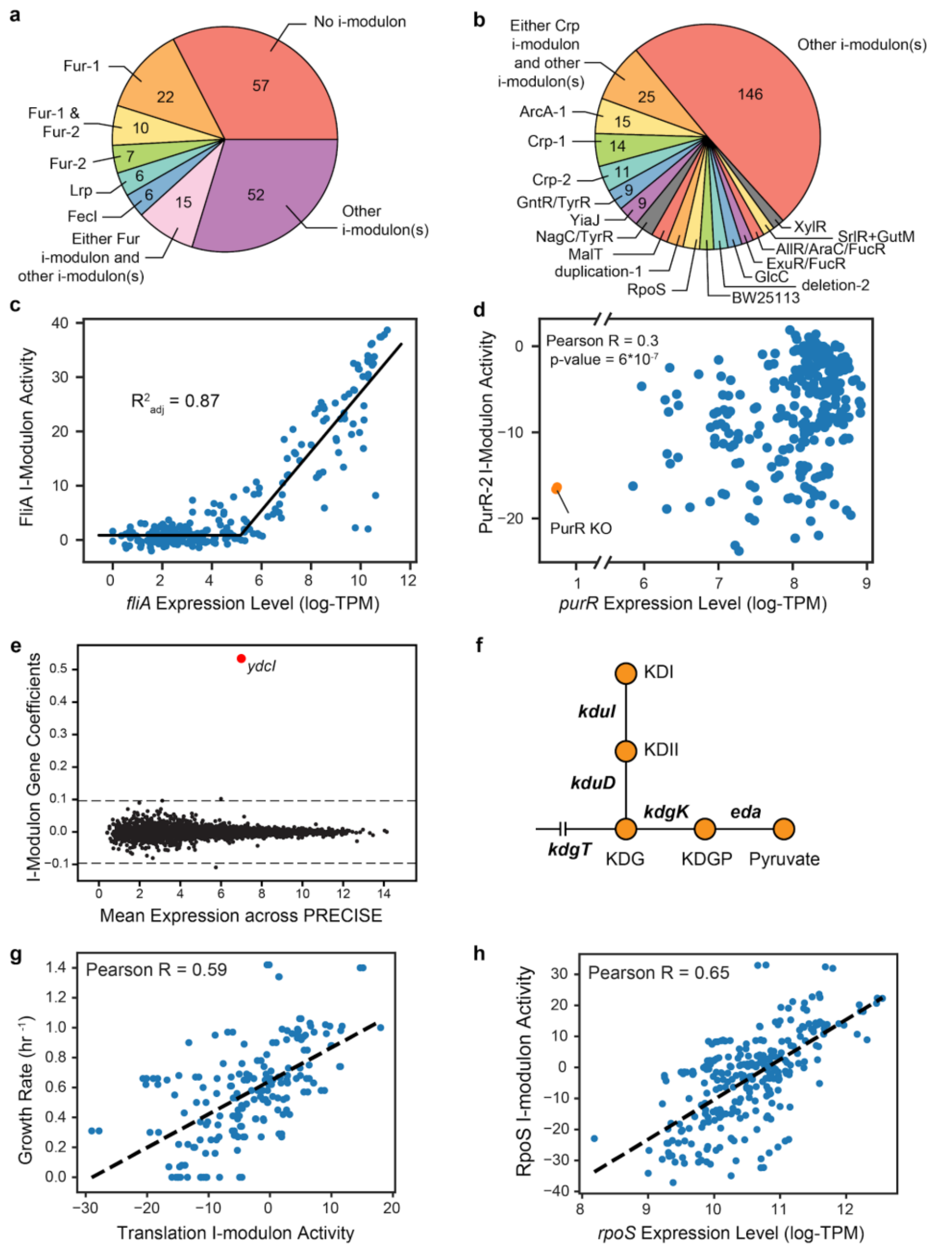

Supplementary Figure 4: Additional properties of i-modulons

(a,b) I-modulon composition of the reported Fur and Crp regulons. 291 genes in the reported Crp regulon were not in any i-modulon and are not shown. (c) The FliA i-modulon activity level was highly correlated with the log-transformed *fliA* expression level above a certain expression level. See methods for explanation of the adjusted R^2 value. (d) Unlike the PurR-1 i-modulon, the PurR-2 i-modulon activity level was poorly correlated with *purR* expression. (e) Example of a genomic i-modulon, activated when *ydcI* is knocked-out. (f) Metabolic pathways related to KDG degradation. Gene names are shown in bold adjacent to the reaction they catalyze. Metabolite abbreviations are: KDG - 2-dehydro-3-deoxy-D-gluconate; KDGP - 2-dehydro-3-deoxy-D-gluconate 6-phosphate; KDII - 3-deoxy-D-glycero-2,5-hexodiulosonate; KDI - 5-dehydro-4-deoxy-D-glucuronate. (g) The Translation i-modulon activity level was correlated with growth rate. (h) The RpoS i-modulon activity level was correlated with *rpoS* gene expression.

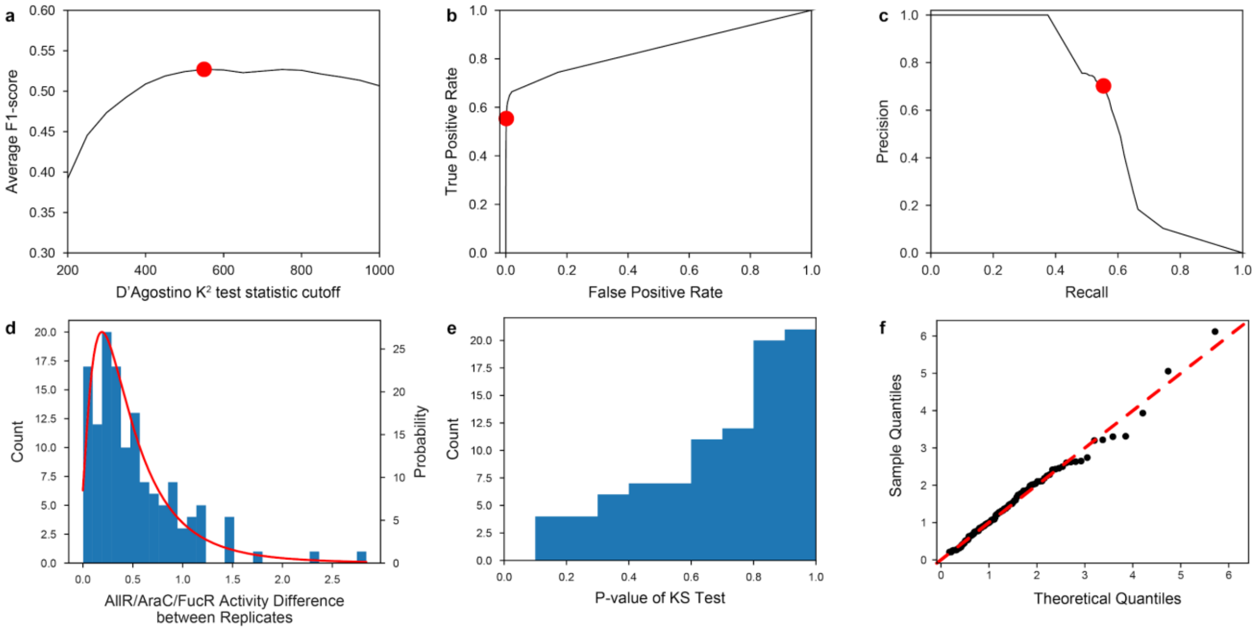

Supplementary Figure 5: Significance thresholds for components and differential activity

(a) Sensitivity analysis on the D'Agostino K^2 test statistic cutoff. Red dot indicates optimal value. (b) Precision-recall curve and (c) receiver operating characteristic (ROC) curve from varying the cutoff. (d) The difference in ArgR i-modulon activities between biological replicates follows a log-normal distribution (shown in red). (e) P-values from K-S tests on each i-modulon are higher than 0.1, indicating that an inability to reject the null hypothesis that the activity differences between replicates are from a log-normal distribution. (f) Quantile-quantile plot of difference in AllR/AraC/FucR i-modulon activities between biological replicates provides further evidence of log-normal distribution.

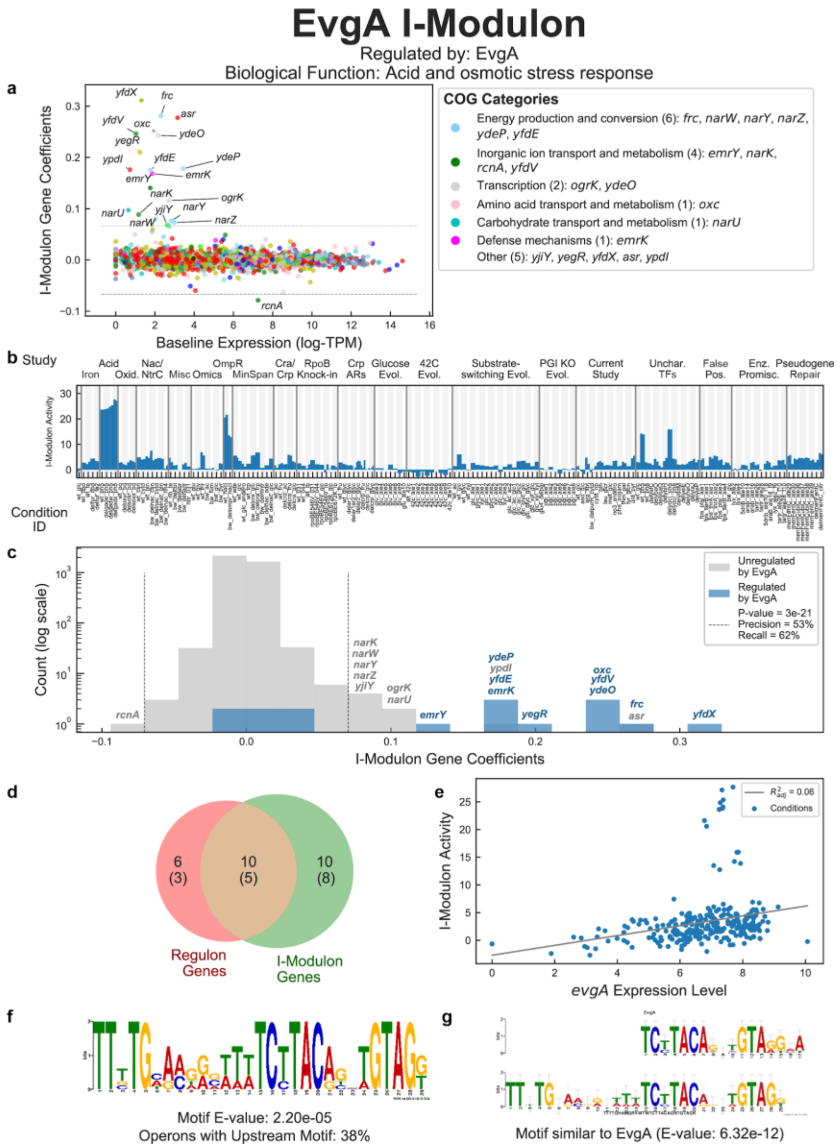

Supplementary Figure 6: Descriptive characteristics of the EvgA i-modulon with explanations

Similar characteristics are shown for all 92 i-modulons in Supplementary Data 2. (a) Scatterplot of i-modulon gene coefficients against gene expression (log-TPM) in the reference condition (*E. coli* MG1655 grown on glucose M9 minimal medium). Genes are colored by their cluster of orthologous groups (COG) categories. (b) I-modulon activities across the entire PRECISE compendium, grouped by original study. Supplementary Data 1 contains experimental metadata (e.g. carbon source, pH, growth rate, GEO accession, etc.) for each condition ID. Each condition occupies a constant width, regardless of the number of biological replicates. (c) Histogram of i-modulon gene coefficients on a logarithmic count scale. Two histograms are overlaid, one for genes with reported regulation by the associated regulator, and one for genes without reported regulation. If multiple regulators are associated, additional histograms are overlaid. Gene names are listed above bars outside of the significance threshold (dashed vertical line). Statistics on regulator enrichment are reported in the legend. (d) Venn diagram of genes in associated regulon vs. significant genes in i-modulon. Numbers in parenthesis indicate number of operons in each category. (e) Scatterplot of i-modulon activity against expression level of associated regulator. Regulator knock-outs, if applicable, are indicated in orange. The R^2_{adj} accounts for minimum expression levels, as discussed in Supplementary Figure 4c and Methods. (f) Motif identified upstream of i-modulon genes, if found. (g) Similar motif in RegulonDB to the motif identified in (f), if found.

Supplementary Notes

Supplementary Note 1: Overview of i-modulons

AllR/AraC/FucR
Genes: Contains three carbohydrate uptake regulons
Activating Conditions: ALE strains with mutations in *araC* and *fucR* [4]
Confidence: High

ArcA-1
Genes: Primarily contains genes in energy production and conversion. Although the precision is low (66%), the ArcA motif was identified upstream of 85% of genes.
Activating Conditions: Downregulated in anaerobic and nitrate respiration media Conditions. Also downregulated in various ALE endpoints.
Confidence: Medium

ArcA-2
Genes: Contains various cytochromes and hydrogenases. These operons may have complex regulation, limiting the confidence of this regulator association.
Activating Conditions: Activated in anaerobic and nitrate respiration media conditions
Confidence: Medium

ArgR
Genes: Only contains 10% of the ArgR regulon, but contains all genes in arginine biosynthetic pathway (See Supplementary Figure 2a).
Activating Conditions: Strongest downregulation when grown in LB and under paraquat-induced oxidative stress. Not activated by arginine supplement.
Confidence: Medium

AtoC
Genes: *dmlA* is mutated in the two strains with high activity, indicating that it may not be part of the AtoC regulon. Precision increases from 67% to 80% after removing this gene.
Activating Conditions: Active when weaning *E. coli* from glycerol substrate to m-Tartrate substrate [4]. Not active after *E. coli* has evolved in response to the new substrate
Confidence: High

BW25113: See section in Paper

Cbl+CysB

Genes: Missing *ssuB* is in the *ssuABCDE* transcription unit, but falls just below threshold potentially due to expression noise. Includes *iraD*, which was not previously reported to be in the regulon, and may not have a cysB binding site.
Activating Conditions: Growth on sub-optimal sulfur source (glutathione). Consistent with published regulator activity [5]
Confidence: High

CdaR:
Genes: *dmlA* is mutated in some strains with high activity, indicating that it may not be part of the CdaR regulon.
Activating Conditions: Upregulated when grown on the following carbon sources: D-glucarate (consistent with literature), D-ribose, m-tartrate, and D-arabinose.
Confidence: High

CecR
Genes: Perfect concordance with known regulon.
Activating Conditions: Strongly upregulated in Δ*cecR* (Δ*ybiH*) strain, indicating it is a constitutive repressor. Somewhat upregulated under osmotic stress (0.3M NaCl)
Confidence: High

Copper
Genes: Genes are regulated by two distinct transcription factors directly activated by copper ions (CusR and CueR). HprR has an identical regulon to CueR, but senses hydrogen peroxide instead.
Activating Conditions: Highly variable, similar to other metal-dependent i-modulons (NikR and Zinc).
Confidence: High

CpxR
Genes: Low precision (47%) and recall (13%) limits the confidence of this i-modulon. However, *cpxP* has the highest gene coefficient, and is only known to be regulated by CpxR
Activating Conditions: Active under osmotic stress only when OmpR is deleted. Also active in Δ*ybiH* strain
Confidence: Low

Cra
Genes: Glycolysis genes have positive gene coefficients, gluconeogenic genes have negative gene coefficients. Thus this i-modulon directs the direction of flux through glycolysis. The *fruABK* transcription unit contains the largest gene coefficients, indicating that its expression changes the most in response to changing Cra activity.

Activating Conditions: Highly upregulated when Cra is knocked-out, indicating that Cra constitutively represses glycolytic genes and activates gluconeogenic genes (consistent with literature [6]). Also highly upregulated when fructose is substrate, and downregulated when suboptimal carbon sources (acetate/pyruvate) are substrates.
Confidence: High

Crp-1
Genes: 83% of genes have an upstream CRP motif
Activating Conditions: Upregulated when CRP activating region-II is deleted [7].
Confidence: Medium

Crp-2
Genes: Various carbohydrate uptake genes, 63% of genes have an upstream CRP motif
Activating Conditions: Downregulated in all strains with complete or partial CRP deletion.
Confidence: Medium

CsqR
Genes: All genes are directly adjacent, suggesting they belong to two transcription units (*yihV-csqR* and *ompL-yihOPQRSTU*).
Activating Conditions: Upregulated in ALE strains containing mutations in CsqR, likely causing loss-of-function[4].
Confidence: High

CysB
Genes: CysB binding confirmed upstream of all genes in i-modulon (precision increased from 76% to 100%)
Activating Conditions: Strongly downregulated under nitrate respiration, paraquat-induced oxidative stress, and growth on ribose as a substrate.
Confidence: High

DhaR/Mlc
Genes: Contains 3 phosphotransferase systems: *mtlAD, manXYZ,* and *ptsG*. Also contains *galP* and dihydroxyacetone kinase (*dhaKLM*).
Activating Conditions: Strongest activity in Δ*ptsI* strains.
Confidence: Medium

EvgA
Genes: 50% precision may be improved by additional genome-binding data.
Activating Conditions: Strongly upregulated by acid stress, and moderately upregulated by osmotic stress (consistent with literature[8])

Confidence: High

ExuR/FucR
Genes: Contains 4 y-genes of unknown function: *yagE, yagF, yeiQ*, and *yqhD*. Many of these are hypothesized to be enzymes related to glycolaldehyde metabolism.
Activating Conditions: Most active in arabinose tolerization ALE strains [4], which have a mutation in FucR.
Confidence: High

FadR
Genes: High precision (77%) and low recall (40%) may be explained by the activating condition. FabR is mutated in BW25113, which is the base strain for this knock-out, potentially affecting the rest of the regulon.
Activating Conditions: Upregulated in Δ*fadR* strain
Confidence: Medium

FecI
Genes: Contains various genes in addition to the known FecI regulon (*fecABCDE*), likely due to mutations or knock-outs in ALE strains with high i-modulon activaties.
Activating Conditions: Most active when Ferric Citrate is available in the media (consistent with literature [9])
Confidence: Medium

FlhDC
Genes: 39/41 of genes are directly related to flagellar biosynthesis. We propose that the remaining genes (*yecR, ymdA*) are also related to flagellar biosynthesis.
Activating Conditions: Highly variable across compendium
Confidence: High

FliA
Genes: Motif reminiscent of -35 and -10 box identified for *E. coli* sigma factors
Activating Conditions: Highly variable across compendium
Confidence: High

Fnr
Genes: 82% of genes have upstream FNR motif
Activating Conditions: Highly variable across compendium, but never significantly lower than the reference condition (aerobic growth on glucose minimal media).
Confidence: Medium

Fur-1
Genes: Fur is known to primarily repress genes. However, the negative coefficients of *sodB*, *yoeG*$_1$, and *bfr* indicate that these genes are activated by Fur[10].
Activating Conditions: Highly variable, but strongly upregulated (derepressed) under iron starvation conditions (consistent with literature[11])
Confidence: High

Fur-2
Genes: Shares 11 of 27 genes with Fur-1 i-modulon
Activating Conditions: Not as variable as Fur-1, but activation pattern is less clear.
Confidence: Medium

GadEWX
Genes: Contains genes regulated by all three glutamate-dependent acid response regulators (*gadE*, *gadW*, and *gadX*).
Activating Conditions: Strongly downregulated in ALE endpoints, likely due to the fear vs. greed trade-off mentioned in the main paper. Also active under iron starvation, oxidative stress, and acid stress. Strongly downregulated in Δ*gadE* strain, but unaltered in Δ*gadW* and Δ*gadX* strains, indicating that this i-modulon is *gadE*-dependent.
Confidence: Medium

GadWX
Genes: Shares 6 of 8 genes with GadEWX i-modulon
Activating Conditions: Similar to GadEWX, except for Δ*gadW* and Δ*gadX* strains, indicating this response is dependent on both genes.
Confidence: High

GcvA
Genes: Only one discrepancy *ygfF*, which is uncharacterized. May not be in GcvA regulon.
Activating Conditions: Strongest upregulation when supplemented with glycine or grown in LB. Strongest downregulation in ALE endpoint strains, iron starvation, and oxidative stress.
Confidence: High

GlcC
Genes: 100% precision, only missing *glcC* indicating it may not be self-regulating.
Activating Conditions: Carbon-source dependent activation
Confidence: High

GlpR
Genes: 100% accuracy

Activating Conditions: Activated when grown on glycerol, as it regulates glycerol uptake
Confidence: High

GntR/TyrR
Genes: GntR represses genes in the absence of gluconate, whereas tyrosine represses most genes (except *mtr*) in the presence of tyrosine. Thus the signs of the genes in each regulon are consistent with the underlying biology. See Supplementary Figure 3e.
Activating Conditions: Active on a single condition, when grown with gluconate as the carbon source and tyrosine in the media.
Confidence: High

His-tRNA
Genes: The regulatory mechanism of the histidine operon was mutated in the Δ*serB* ALE strains, indicating that the presence of *serB* in this i-modulon not regulation-related.
Activating Conditions: Highly active in Δ*serB* ALE strains, where the histidine tRNA-regulatory region was mutated.
Confidence: High

Leu/Ile
Genes: See Supplementary Figure 3g. High concentrations of leucine or isoleucine either charge tRNAs that pause transcription to form termination loops, or activate the transcription factor IlvY to repress transcription of ilvC. *yaaX* may not be a true member of the i-modulon, as its coefficient is just above the threshold.
Activating Conditions: Strongly upregulated under oxidative stress and iron-starvation (only WT strain, not Δ*fur* strain). Strongly downregulated under osmotic stress (0.3M NaCl), or growth on LB.
Confidence: Medium

Lrp
Genes: Primarily amino acid transport and metabolism. High precision (86%) and low recall (16%) is consistent with other global regulators (See Supplementary Figure 4a,b). Other genes in the Lrp regulon may belong to other i-modulons (e.g. Leu/Ile).
Activating Conditions: Upregulated in LB, iron starvation, and amino acid supplementation (specifically arginine, leucine, and methionine)
Confidence: High

MalT
Genes: Near-perfect overlap with MalT regulon. Missing genes *malZ* and *malS* may be under more complex regulation than MalT.

Activating Conditions: Downregulated in all CRP mutants missing AR-1[7], but upregulated in CRP mutant missing AR-2 only. Lowest activity in PGI ALE6[12], which has a truncation of MalT and near-zero expression.
Confidence: High

MetJ
Genes: ChIP-exo validation increases precision from 65% to 100%.
Activating Conditions: Down-regulated in presence of methionine, up-regulated under iron starvation. SNP at V46E in FPS thrA ALE2 may result in derepression of MetJ-regulated genes.
Confidence: High

Nac
Genes: High precision (86%), low recall (6%), palindromic motif identified upstream of 72% of genes.
Activating Conditions: Upregulated in nitrogen starved conditions (nitrogen-limited chemostat, and non-ammonia nitrogen sources), and when cytidine is present in media. Downregulated in Δ*nac* strains.
Confidence: High

NagC/TyrR
Genes: See GntR/TyrR and Supplementary Figure 3d
Activating Conditions: High activity in phenylalanine/N-acetylglucosamine media. Also upregulated in ALE strains with mutations in *nagBAC*[13].
Confidence: High

NarL
Genes: Genes involved in nitrate respiration. High precision (93%) low recall (23%).
Activating Conditions: Upregulated when nitrate is terminal electron acceptor (*ica__no3_anaero*). Also upregulated in CRP mutants and various yTF deletion strains (*yafC*, *ybaO*, *yeiE*, *yheO*, *yiaJ*, *yieP*).
Confidence: Medium

NikR
Genes: Near-perfect overlap. *yafC* may not be in regulon as it is very close to threshold.
Activating Conditions: Highly variable, similar to other metal-dependent i-modulons (Copper and Zinc).
Confidence: High

NtrC+RpoN
Genes: Co-regulated by NtrC and RpoN, which are known to act together.

Activating Conditions: Upregulated in nitrogen starvation conditions (non-ammonia nitrogen sources and nitrogen-limited chemostats).
Confidence: High

OxyR
Genes: Perfect precision, but low recall (17%).
Activating Conditions: Oxidative stress (except in Δ*oxyR* strain), and ALE endpoints with OxyR mutations.
Confidence: High

PrpR
Genes: Two hitchhikers (*ptsI* and *galP*) may be due to the ptsI-KO ALE strain.
Activating Conditions: Activated in ptsI-KO ALE strain, and in the enzyme promiscuity study. Also highly active when grown on galactose as a carbon source.
Confidence: High

PurR-1
Genes: Genes in purine biosynthetic pathway, plus *add*, *ydhC*, and *cvpA*. *purR* is just below the significance threshold.
Activating Conditions: Downregulated when adenine is supplemented.
Confidence: High

PurR-2
Genes: Genes in pyrimidine biosynthetic pathway. Most operons are known to be regulated by UTP-dependent reiterative transcription[14], but may also be coregulated by PurR. No overlap between PurR-1 and PurR-2. Unlike other split i-modulons (e.g. Fur, GadEWX) these i-modulons seem to modulate completely different systems.
Activating Conditions: Lowest activity under iron starvation, oxidative stress, nitrogen starvation, growth in LB media, and cytidine supplementation.
Confidence: Medium

PuuR
Genes: 100% precision and recall.
Activating Conditions: Upregulated when grown with ribose as a carbon source and in the *serB* KO ALE.
Confidence: High

Pyruvate
Genes: This i-modulon was dominated by the pyruvate transporter gene *btsT35* and the putative pyruvate transporter gene *yhjX*. The gene coefficients were consistent with their reported

regulatory strategies; the BtsSR two-component system regulates *btsT* and contains a high-affinity pyruvate receptor, whereas the YpdAB two-component system regulates *yhjX* at lower pyruvate concentrations[15]. Four additional genes were regulated by the pyruvate-responsive regulator PdhR.
Activating Conditions: Growth on minimal media with pyruvate as the primary carbon source.
Confidence: Medium

RbsR
Genes: High precision (83%); three genes (*purH, purD*, and *add*) may be incorrectly annotated as part of this regulon.
Activating Conditions: Growth on minimal media with ribose as the primary carbon source. Also ALE endpoints with RbsR mutations[4]
Confidence: High

RcsAB
Genes: Low precision (36%), but high recall (62%). Genes with no known rcsAB binding share similar function (capsule formation) to other genes in i-modulon, indicating that there may be many previously undetected RcsAB binding sites. In addition, rcsA is known to be degraded by *lon* protease, which is on the negative side of the i-modulon[16].
Activating Conditions: ΔCRP strains (explains *crp* presence in i-modulon), osmotic stress (0.3M NaCl), and PGI KO ALE endpoints[12]. Activity level is correlated with *rcsA* (but not *rcsB*) expression levels above a threshold.
Confidence: Medium

RpoH
Genes: 100% precision, 10% recall, significance threshold could be relaxed and still maintain precision. See Supplementary Figure 2b.
Activating Conditions: Variable conditions, but significantly higher when misfolded proteins are present (data not shown).
Confidence: High

RpoS
Genes: Largest i-modulon containing over 100 genes related to stress response. Contains the catalase *katE* and pyruvate oxidase *poxB*, which are often used as a proxy for RpoS activity [17,18]. Although the precision and recall are low (37% and 13%, respectively), a sigma-factor like motif (e.g. -10 and -35 boxes) was found upstream of 98% of genes.
Activating Conditions: See Figure 5C.
Confidence: High

SoxS

Genes: Precision and recall are medium (56% and 43%, respectively).
Activating Conditions: Oxidative stress, except in Δ*soxS* strain.
Confidence: High

SrlR+GutM
Genes: Near-perfect overlap (missing srlR and gutQ)
Activating Conditions: Growth on minimal media with either sorbitol or ribose as carbon sources.
Confidence: High

Thiamine
Genes: 100% precision and recall, regulated by thiamine riboswitch
Activating Conditions: Downregulated when thiamine is present in media and in LB media.
Confidence: High

Tryptophan
Genes: Regulated by combination of L-tryptophan riboswitch, tryptophan-tRNA-mediated transcriptional attenuation, and TrpR. See Supplementary Figure 3f. Tryptophan riboswitch upregulates tryptophan degradation operon *tnaABC* in the presence of tryptophan, whereas TrpR represses tryptophan biosynthetic genes in presence of tryptophan. Also includes *aroF* and *tyrA*, which are regulated by tyrosine.
Activating Conditions: Strong negative in presence of tryptophan (*tnaAB* are upregulated, and TrpR-regulated genes are downregulated).
Confidence: High

XylR
Genes: Low precision, likely because some members of the regulon have not yet been discovered.
Activating Conditions: Growth on xylose and lyxose.
Confidence: High

YgbI
Genes: See supplementary text and Figure 4f
Activating Conditions: ALE endpoints with mutations
Confidence: High

YiaJ
Genes: Near perfect overlap, see Figure 4d
Activating Conditions: Δ*yiaJ* strain, and CRP AR-II mutant strain[7].
Confidence: High

YieP
Genes: *yohJK*, *ykgEFG* have upstream binding sites[19], unsure about other genes, see Figure 4e
Activating Conditions: Δ*yieP* strain.
Confidence: Medium

YneJ
Genes: See supplementary text and Figure 4f
Activating Conditions: ALE endpoint strain with mutation[20]
Confidence: High

Zinc
Genes: Contains genes regulated by two zinc-sensing regulators (ZntR and Zur). Also contains some copper-related genes (*copA* and *cueO*).
Activating Conditions: Similar to other metal sensing i-modulons (Copper, NikR), i-modulon activites are fairly variable.
Confidence: High

Non-regulatory I-modulons:

crp-KO
Genes: Dominated by *crp*, but also includes some unknown function genes that are known to be regulated by CRP
Activating Conditions: Strongly upregulated in CRP complete knock-out, but strongly downregulated in partial CRP knock-out.

curli
Genes: *csgDEFG* curli subunits
Activating Conditions: Strongly downregulated under osmotic stress

deletion-1
Genes: Genes in 36 kilobase deletion, plus 4 genes putatively regulated by deleted regulator KdgR. See Figure 4g.
Activating Conditions: Strain with 36 kb deletion[4]

deletion-2
Genes: Genes in 100 kilobase deletion, plus a few additional genes with negative coefficients
Activating Conditions: Strain with 100 kb deletion[4]

duplication-2

Genes: Genes in a 129-gene duplication, plus a few additional genes with negative coefficients
Activating Conditions: Strain with duplication[4]

e14-deletion
Genes: e14-prophage between *icdC* and *icd*
Activating Conditions: CRP partial and full knockouts (presumably e14-prophage deletion occurred during strain construction), and one ALE strain (verified e14-prophage deletion) [20,21].

efeU-repair
Genes: *efeUOB*, *entEBAH*
Activating Conditions: *entC* deletion strains (*entC* is part of *entCEBAH* operon), one of which has a repair of the *efeU* pseudogene[20].

entC-menF-KO
Genes: *entC*, *menF*, and *menD*
Activating Conditions: Strains with *entC* and/or *menF* removed

fimbriae
Genes: Fimbrial complex proteins, plus *thrA*, likely due to high activity in a *thrA* deletion strain.
Activating Conditions: Highly variable

flu-yeeRS
Genes: *flu-yeeRS* CP4-44 prophage genes, plus various genes likely due to deletions in strains (*fur*, *thrA*, *menF*, *entC*, *serB*)
Activating Conditions: Highly variable

fur-KO
Genes: Dominated by *fur*, but also includes some genes in the e14-prophage and DNA repair proteins (*recN*, *yebG*) indicating DNA damage during construction of strain [21].
Activating Conditions: Fur KO strains

gadWX-KO
Genes: *gadW*, *gadX*
Activating Conditions: Δ*gadW* and Δ*gadX*

insertion
Genes: *insCD-1*, and three genes of unknown function with negative coefficients
Activating Conditions: ALE strain with insertion of insCD-1

iron-related

Genes: Dominated by *yjjZ* an uncharacterized gene. Also contains various genes related to iron transport, iron-sulfur formation, and oxidative stress detoxification
Activating Conditions: Negative activity in iron starvation, Fur KO, and oxidative stress.

lipopolysaccharide
Genes: Contains many genes related to lipopolysaccharide biosynthesis, but no regulator is known for these genes.
Activating Conditions: Highly variable

membrane
Genes: All genes are membrane-bound, but most are uncharacterized
Activating Conditions: Highly variable

nitrate-related
Genes: Various genes related to nitric oxide response
Activating Conditions: Nitrate respiration

proVWX
Genes: Glycine-betaine osmoprotectant transport (*proVWX*) and *kdpA* potassium ion pump subunit.
Activating Conditions: Osmotic stress (0.3M NaCl)

purR-KO
Genes: *purR, pyrE,* and *add*. *pyrE* is likely related to the *pyrE-rph* defect [22], whereas *add* is likely decoupling the regulation of *add* from PurR itself.
Activating Conditions: Strong negative activity on PurR KO

sgrT
Genes: *sgrT*, plus two genes adjacent to the significance threshold. *sgrT* is a putative inhibitor of *ptsG* transporter[23]
Activating Conditions: All PGI KO ALEs[12]

thrA-KO
Genes: *thrA*, and two genes adjacent to the threshold
Activating Conditions: Δ*thrA* strains

translation
Genes: Various ribosomal subunit-encoding genes and translation-related genes. Also includes two ion transporters and two energy production proteins (*zraP* has negative coefficient).
Activating Conditions: Correlated with growth rate (See Supplementary Figure 4g)

ydcI-KO
Genes: *ydcI*, and two genes adjacent to the threshold
Activating Conditions: Δ*ydcI* strains

yheO-KO
Genes: *yheO*, and many genes adjacent to the threshold. Some genes with negative coefficients have YheO binding sites, indicating this may be a regulatory i-modulon [19]
Activating Conditions: Δ*yheO* strain

Supplementary Note 2: Explanation of ALE-derived i-modulons

The YgbI i-modulon contains five consecutive genes with positive coefficients (*ygbJKLMN*), two other genes with positive coefficients (*dmlA*, *glxR*), and two genes with negative coefficient (*kduI*, *araA*). Of the three strains with mutations in YgbI, two strains have a mutation upstream of *dmlA* that likely affects its expression, and one strain has a large deletion including *kdgR* (Supplementary Table 6). We hypothesized that *kdgR* was a regulator of *kduI* (Figure 4f,g), potentially explaining why *kduI* may appear in this component. Thus, only *ygbJKLMN* and potentially *glxR* are likely to be directly regulated by YgbI.

Similarly, the strain with the active YneJ i-modulon is missing the genes *entC* and *proQ* due to genome deletions, explaining their presence in this i-modulon.

Supplementary Methods

Automated characterization of i-modulons

Supplementary Data 2 contains detailed information on each i-modulon. Each page characterizes one i-modulon, which are sorted by name. The EvgA i-modulon page is described in Supplementary Figure 6a-g.

The scatter plot (Supplementary Figure 6a) compares the i-modulon gene weights to the reference expression profile (defined as the average log-TPM of sample IDs *control__wt_glc__1* and *control__wt_glc__2*). The horizontal dashed lines represent the threshold separating significant and non-significant genes in an i-modulon. Each gene is colored by its functional category, defined by clusters of orthologous groups of genes queried from EggNOG[24]. Gene names are provided on the scatterplot if the total number of significant genes is below 25. Subscripts indicate split genes in *E. coli* MG1655, classified as pseudogenes.

The bar chart (Supplementary Figure 6b) displays the i-modulon activities across all conditions in the database, grouped by study. Each condition shares the same total width, regardless of the number of biological replicates. The sample IDs correspond to the IDs in Supplementary Data 1.

The histograms (Supplementary Figure 6c) show the gene weights in an i-modulon on a log-scale, split by enriched regulon. The vertical bars represent the significance threshold for the component. The gray histogram captures genes that are not in an enriched regulon. The brown histogram captures genes that are in more than one enriched regulon.

The venn diagram (Supplementary Figure 6d) compares genes in an i-modulon (above the threshold) to genes in the enriched regulons. If the i-modulon name contains a slash (/), the regulon circle contains the union of genes in any enriched regulon (i.e. genes in either regulon). If the i-modulon name contains a plus sign (+), the regulon circle contains the intersection of the regulons (i.e. genes in both regulons). Numbers in parentheses indicate number of operons (complete or partial) in each category. Operons were defined using RegulonDB. This panel is hidden if no enriched regulons were identified.

The regulon scatter plot (Supplementary Figure 6e) compares the expression level of enriched regulators to the i-modulon activity. Each point represents an expression profile. Two lines were fit to each scatter plot, a simple line and a broken line. The broken line represents a minimal expression level required before a correlation is observed between the i-modulon activity and the regulator expression. Only the line with the highest R^2_{adj} is shown, where

(1) $R^2_{\mathrm{adj}} = 1 - (1-R^2)(n-1)/(n-k-1)$

The broken line is modeled as:

(2) $y = a*c + b$ if $x < c$
(3) $y = a*x + b$ if $x >= c$,

where *x* is the expression level of the gene encoding the TF, and *y* is the i-modulon activity level. Parameter *c* is optimized using the *optimize.curve_fit* function in the Python *scipy* package[25]. Three initial values for *c* were tested to identify the optimal fit: minimum expression, maximum expression, and mean expression.

This panel is hidden if no enriched regulons were identified, or if expression levels were not measured for the regulator (see Thiamine i-modulon). If there are two or more enriched regulons with expression levels, the two TFs with the strongest association to the i-modulon are shown. Experimental conditions with knocked-out TFs are marked in orange and were not used to find the best-fit line.

Motif Search

The motifs (Supplementary Figure 6f) were identified by searching upstream sequences using MEME[26]. Genes in each i-modulon were grouped into operons. A stringent upstream sequence was defined as the region from -300 to the start of the operon. We searched for zero or one motif per sequence using an E-value threshold of 10^{-3}, searching for motifs with all widths between 6 and 30 basepairs, and minimum number of found motifs equal to ⅓ the total number of operons for all non-genomic i-modulons. For i-modulons with no enriched motifs in the stringent upstream sequence, we searched for motifs in a broader upstream sequence of -600 to +100, keeping all other parameters constant. The E-value of the motif and the percent of i-modulon operons with the upstream motif are listed below the motif.

The motif comparison (Supplementary Figure 6g) was generated by comparing i-modulon motifs against known motifs from RegulonDB using TOMTOM[27], with an E-value threshold of 0.01 and allowing incomplete matches. The E-value of the comparison is shown below the figure.

For regulatory i-modulons, genes with identified motif sites but no known regulation by the associated TF are reported in Supplementary Table 4.

Supplementary Tables

Supplementary Table 1: Overview of i-modulons derived from PRECISE.
I-modulons are either named after their associated regulator(s) or their biological function. Strong activity-TF relationships ($R^2_{adj} > 0.4$) are marked in bold.

I-modulon Name	Number of Genes	Regulator(s)	Enrichment P-value	Precision	Recall	Activity-TF R^2_{adj}	Biological Function
**Amino Acid and Nucleotide Biosynthesis (9)**							
ArgR	13	ArgR	6E-18	0.92	0.10	0.38	Arginine biosynthesis
CysB	21	CysB	4E-32	0.76	0.52	0.28	Inorganic sulfate assimilation
His-tRNA	9	His-tRNA attenuation	6E-24	0.89	1.		Histidine biosynthesis
Leu/Ile	14	IlvY or<br>leu-tRNA attenuation or<br>ile-tRNA attenuation	4E-33	0.93	0.76	0.02	Branched-chain amino acid biosynthesis
Lrp	37	Lrp	1E-37	0.86	0.16	0.13	Amino acid and peptide transport
MetJ	17	MetJ	2E-25	0.65	0.73	**0.46**	Methionine biosynthesis
PurR-1	16	PurR	2E-25	0.81	0.36	0.23	Purine Biosynthesis
PurR-2	10	PurR	3E-13	0.70	0.19	0.11	Pyrimidine biosynthesis
Tryptophan	11	TrpR or<br>trp-tRNA attenuation or<br>Tryptophan attenuation	4E-21	0.82	0.5	0.02	Tryptophan Biosynthesis
**Carbon Source Utilization (17)**							
AllR/AraC/FucR	18	AllR or<br>AraC or<br>FucR	1E-41	1.	0.6	0.33<br>0.38<br>**0.48**	Allantoin, glyoxylate, L-arabinose, and L-fucose catabolism
CdaR	10	CdaR	2E-26	0.9	1.	**0.74**	Glucarate catabolism
Cra	15	Cra	7E-16	0.8	0.09	0.02	Central carbon metabolism
Crp-2	40	Crp	4E-15	0.7	0.05		Various carbon source catabolism
DhaR/Mlc	11	DhaR or<br>Mlc	4E-19	0.73	0.57	0<br>0.28	Dihydroxyacetone kinase and phosphotransferase systems
ExuR/FucR	15	ExuR or<br>FucR	1E-22	0.67	0.63	0.07<br>**0.44**	D-galacturonate and L-fucose catabolism
GlcC	6	GlcC	1E-18	1.	0.86	**0.42**	Glycolate catabolism
GlpR	9	GlpR	0	1.	1.	0.02	Glycerol catabolism
GntR/TyrR	15	GntR or<br>TyrR	3E-29	0.87	0.54	0 0	Gluconate catabolism and tyrosine biosynthesis
MalT	9	MalT	3E-22	0.89	0.8	**0.69**	Maltose catabolism
NagC/TyrR	17	NagC or<br>TyrR	4E-31	0.94	0.33	0.03<br>0.01	N-acetylglucosamine catabolism and tyrosine biosynthesis
PrpR	6	PrpR	8E-12	0.67	0.8	0.36	Propionate catabolism
Pyruvate	8	BtsR or<br>YpdB or<br>pdhR	9E-07	0.50	0.09	0.04<br>0<br>0.24	Pyruvate transport and metabolism
RbsR	6	RbsR	1E-13	0.83	0.56	**0.64**	D-ribose catabolism
SrlR+GutM	5	SrlR and<br>GutM	3E-15	1.	0.71	0.38<br>**0.40**	Sorbitol catabolism
XylR	13	XylR	2E-15	0.46	0.86	**0.83**	Xylose catabolism
YiaJ	10	YiaJ	5E-29	1.	0.91	0.07	Ascorbate utilization
**Energy Metabolism (4)**							
ArcA-1	50	ArcA	9E-20	0.66	0.07	0.03	Electron Transport Chain
ArcA-2	25	ArcA	1E-16	0.84	0.05	0	Anaerobic response
Fnr	40	Fnr	3E-27	0.85	0.08	**0.57**	Anaerobic response
NarL	29	NarL	1E-40	0.93	0.23	0.02	Nitrate respiration
**Metal Homeostasis (6)**							
Copper	8	CusR or<br>HprR or<br>CueR	5E-20	1.	0.42	**0.76**<br>0.25<br>0.03	Copper homeostasis
FecI	10	FecI	3E-14	0.5	1.	0.07	Ferric citrate transport
Fur-1	48	Fur	5E-55	0.9	0.25	0	Iron homeostasis
Fur-2	27	Fur	3E-26	0.81	0.13	0.03	Iron homeostasis

NikR	6	NikR	5E-15	0.83	0.83	0.04	Nickel homeostasis
Zinc	12	ZntR or<br>Zur	3E-19	0.58	1.	0<br>0.01	Zinc homeostasis
**Miscellaneous Metabolism (7)**							
AtoC	6	AtoC	2E-12	0.67	1.	0.04	Acetoacetate degradation
Cbl+CysB	10	Cbl and<br>CysB	3E-22	0.80	0.89	**0.73**<br>0.10	Aliphatic sulfonate utilization
Crp-1	60	Crp	7E-10	0.48	0.05	0.05	Miscellaneous Functions
CsqR	10	CsqR	3E-14	0.5	1.	**0.89**	Sulfoquinovose catabolism
FadR	13	FadR	6E-19	0.69	0.41	0.06	Fatty acid degradation
GcvA	4	GcvA	2E-9	0.75	0.75	0.02	Glycine cleavage system
PuuR	7	PuuR	0	1.	1.	**0.7**	Putrescine catabolism
Thiamine	11	Thiamine	0	1.	1.		Thiamine biosynthesis
**Stress Response (11)**							
CecR	5	CecR	0	1.	1.	0.08	Related to antibiotic sensitivity
CpxR	17	CpxR	5E-11	0.47	0.13	0.06	Various stress responses
EvgA	20	EvgA	6E-21	0.5	0.63	0.06	Acid and osmotic stress response
GadEWX	17	GadE and<br>GadW and<br>GadX	1E-24	0.59	0.91	**0.92**<br>**0.61**<br>**0.66**	Acid stress response
GadWX	8	GadW and<br>GadX	5E-21	1.	0.53	**0.68**<br>**0.77**	Acid stress response
Nac	37	Nac	7E-24	0.86	0.06	**0.67**	Nitrogen starvation response
NtrC+RpoN	56	NtrC and<br>RpoN	3E-52	0.57	0.67	**0.51**<br>0.05	Nitrogen starvation response
OxyR	8	OxyR	2E-14	1.	0.17	**0.55**	Peroxide reductases
RpoH	13	RpoH	6E-20	1.	0.1	0	Heat shock response
RpoS	107	RpoS	1E-18	0.37	0.13	**0.43**	General stress response
SoxS	41	SoxS	6E-35	0.56	0.43	**0.74**	Oxidative stress response
**Structural Components (3)**							
FlhDC	41	FlhDC	5E-66	0.93	0.49	**0.72**	Flagella assembly
FliA	30	FliA	4E-54	0.97	0.45	**0.87**	Chemotaxis
RcsAB	28	RcsAB	4E-19	0.36	0.63	**0.63**	Colanic acid capsule formation
**Regulator Discovery (3)**							
YgbI	9	YgbI				**0.7**	Unknown
YieP	11	YieP				0.03	Unknown
YneJ	5	YneJ				**0.63**	Unknown
**Genomic Alterations (15)**							
BW25113	17						Transcriptional difference between BW25113 and MG1655
crp-KO	14						Accounts for crp knock-out
deletion-1	41						Large deletion of 39 genes during evolution
deletion-2	110						Large deletion of 171 genes during evolution
duplication-1	84						Large duplication of 129 genes during evolution
e14-deletion	11						Removal of e14 prophage
efeU-repair	8						Accounts for repair and expression of efeU operon
entC-menF-KO	3						Accounts for entC and menF knock-outs
fur-KO	13						Accounts for fur knock-out
gadWX-KO	2						Accounts for gadW and gadX knock-outs
insertion	9						IS2 insertion element after laboratory evolution
purR-KO	3						Accounts for purR knock-out
thrA-KO	3						Accounts for thrA knock-out
ydcI-KO	3						Accounts for ydcI knock-out
yheO-KO	16						Accounts for yheO knock-out
**Biological Enrichment (10)**							

curli	4						Curli assembly
fimbriae	8						Fimbriae assembly
flu-yeeRS	8						Genes in CP4-44 prophage
iron-related	20						Related to iron metabolism
lipopolysaccharide	36						Lipopolysaccharide biosynthesis
membrane	15						Enriched in membrane-bound proteins
nitrate-related	14						Nitric oxide response
proVWX	4						Glycine betaine transport
sgrT	4						Contains single dominating gene: sgrT
translation	24						Enriched in translation machinery
**Uncharacterized (7)**							
uncharacterized-1	15						Unknown
uncharacterized-2	42						Unknown
uncharacterized-3	2						Unknown
uncharacterized-4	10						Unknown
uncharacterized-5	4						Unknown
uncharacterized-6	54						Unknown

Supplementary Table 2: ChIP-exo binding sites for MetJ

Peak Name	Binding peak location	Peak Intensity	Transcription Unit (+ strand)	Gene Loci (+ strand)	Transcription Unit (- strand)	Gene Loci (- strand)
MetJ-1	83550	35			leuLABCD	b0071;b0072;b0073;b0074;b0075
MetJ-2	179560	23	dgt	b0160	mtn-btuF-yadS	b0157;b0158;b0159
MetJ-3	222715	11	gmhB	b0200	metNIQ	b0197;b0198;b0199
MetJ-4	275303	56	mmuPM	b0260;b0261	insH-1	b0259
MetJ-5	633492	63	ybdL	b0600	ybdH	b0599
MetJ-6	1154392	27	pabC-mltG-tmk-holB-ycfH	b1096;b1097;b1098;b1099;b1100		
MetJ-7	1398937	34			uspE	b1333
MetJ-8	1459265	43	paaABCDEFGHIJK	b1388;b1389;b1390;b1391;b1392;b1393;b1394;b1395;b1396;b1397;b1398		
MetJ-9	1610723	36			uxaB	b1521
MetJ-10	1632086	27			ydfJ_2	b4600_2
MetJ-11	1714512	17	gstA	b1635		
MetJ-12	2132965	26			wza-wzb-wzc-wcaAB	b2058;b2059;b2060;b2061;b2062
MetJ-13	2154128	37	mdtABCD-baeSR	b2074;b2075;b2076;b2077;b2078;b2079	ibsB	b4668
MetJ-14	2563581	46	yffQR	b2448;b2449		
MetJ-15	2564631	29	yffS	b2450		
MetJ-16	3086219	18	yqgC;metK	b2940;b2942	speAB;yqgB	b2937;b2938;b2939
MetJ-17	3152198	78	metC	b3008	exbBD	b3005;b3006
MetJ-18	3243319	39			uxaCA	b3091;b3092
MetJ-19	3353095	58			arcB	b3210
MetJ-20	3458579	49	gspCDEFGHIJKLMO	b3324;b3325;b3326;b3327;b3328;b3329;b3330;b3331;b3332;b3333;b3334;b3335		
MetJ-21	3492425	67	tsgA	b3364	ppiA	b3363
MetJ-22	3719905	39	cspA	b3556		
MetJ-23	3891520	105	mdtL	b3710		
MetJ-24	4011713	126				
MetJ-25	4012917	76	metE	b3829	metR	b3828
MetJ-26	4128305	119	metBL	b3939;b3940	yiiX;metJ	b3937;b3938
MetJ-27	4128579	105	metBL	b3939;b3940	metJ	b3938
MetJ-28	4132531	98	metF	b3941		
MetJ-29	4214175	75	metA	b4013	yjaB	b4012
MetJ-30	4496913	26	intB	b4271		
MetJ-31	4572359	38	yjiT	b4342		

Supplementary Table 3: ChIP-exo binding sites for CysB

Peak Name	Binding peak location	Peak Intensity	Transcription Unit (+ strand)	Gene Loci (+ strand)	Transcription Unit (- strand)	Gene Loci (- strand)
CysB-1	50380	25				
CysB-2	70016	15	araC	b0064	araBAD	b0061;b0062;b0063
CysB-3	113246	22	guaC	b0104	coaE-zapD-yacG	b0101;b0102;b0103
CysB-4	135689	28			yacC-speED	b0120;b0121;b0122
CysB-5	169892	120	fhuACDB	b0150;b0151;b0152;b0153		
CysB-6	252209	229	dinB-yafNOP	b0231;b0232;b0233;b0234		
CysB-7	385122	158	tauABCD	b0365;b0366;b0367;b0368		
CysB-8	401038	216	iraP	b0382	ddlA	b0381
CysB-9	431231	58				
CysB-10	451813	168			cyoABCDE	b0428;b0429;b0430;b0431;b0432
CysB-11	516238	34	fetAB	b0490;b0491	qmcA-ybbJ	b0488;b0489
CysB-12	609875	27			fepA-entD	b0583;b0584
CysB-13	754783	34	sdhCDAB-sucABCD	b0721;b0722;b0723;b0724;b0726;b0727;b0728;b0729	gltA	b0720
CysB-14	815328	26				
CysB-15	850236	23	ompX	b0814	rhtA	b0813
CysB-16	866398	235	iaaA-gsiABCD	b0828;b0829;b0830;b0831;b0832	moeAB	b0826;b0827
CysB-17	904593	26	ybjQ-amiD	b0866;b0867	ybjP	b0865
CysB-18	943784	52	dmsABC	b0894;b0895;b0896		
CysB-19	997106	227			ssuEADCB	b0933;b0934;b0935;b0936;b0937
CysB-20	1053409	23			yccM	b0992
CysB-21	1098910	33	ycdXY	b1034;b1035		
CysB-22	1165389	12	hinT-ycfL-lpoB-thiK-nagZ-ycfP	b1103;b1104;b1105;b1106;b1107;b1108		
CysB-23	1195322	14	icd	b1136	rluE-nudJ	b1134;b1135
CysB-24	1250937	32	dhaR	b1201	dhaKLM	b1198;b1199;b1200
CysB-25	1333725	39	cysB	b1275		
CysB-26	1350219	68			yciW	b1287
CysB-27	1526120	273	yncG	b1454	ansP	b1453
CysB-28	1555778	15			maeA	b1479
CysB-29	1567237	117			dosCP	b1489;b1490
CysB-30	1600329	25			lsrRK	b1511;b1512
CysB-31	1667332	6			mlc-ynfK	b1593;b1594
CysB-32	1706881	12	ydgK-rsxABCDGE-nth	b1626;b1627;b1628;b1629;b1630;b1631;b1632;b1633		
CysB-33	1810832	58	ydjN	b1729		
CysB-34	1818923	13			chbBCARFG	b1733;b1734;b1735;b1736;b1737;b1738
CysB-35	1842359	41	gdhA	b1761	ynjH	b1760
CysB-36	1924627	44	holE	b1842	yobA-yebZY	b1839;b1840;b1841
CysB-37	1977895	273			flhDC	b1891;b1892
CysB-38	2032198	12			dcm-vsr	b1960;b1961
CysB-39	2060953	122			cbl	b1987
CysB-40	2084561	69			yeeED	b2012;b2013
CysB-41	2085611	113			yeeED	b2012;b2013
CysB-42	2110587	12			rfbBDACX	b2037;b2038;b2039;b2040;b2041
CysB-43	2133482	17			wza-wzb-wzc-wcaAB	b2058;b2059;b2060;b2061;b2062
CysB-44	2192823	32			yehE	b2112
CysB-45	2240873	21				
CysB-46	2249630	26	yeiH	b2158	yeiE	b2157
CysB-47	2413410	33	ackA-pta	b2296;b2297	yfbV	b2295
CysB-48	2434800	29			pdxB-usg-truA-dedA	b2317;b2318;b2319;b2320
CysB-49	2489195	9			yfdX-frc-oxc-yfdVE	b2371;b2372;b2373;b2374;b2375
CysB-50	2532389	194	cysK	b2414		
CysB-51	2543583	227			cysPUWAM	b2421;b2422;b2423;b2424;b2425
CysB-52	2551298	36			yfeYX;ypeA-yfeZ	b2431;b2432;b2433;b2434

CysB-53	2563572	21	yffQR	b2448;b2449		
CysB-54	2616204	11	bepA-yfgD	b2494;b2495	yfgO	b2493
CysB-55	2630311	36	yfgHI	b2505;b2506		
CysB-56	2876380	287	iap	b2753	cysDNC	b2750;b2751;b2752
CysB-57	2891912	189	queD	b2765	cysJIH	b2762;b2763;b2764
CysB-58	2971189	26	ygdR;tas	b2833;b2834		
CysB-59	3029593	8				
CysB-60	3263874	9			tdcABCDEFG	b4471;b3113;b3114;b3115;b3116;b3117;b3118
CysB-61	3354689	15	gltBDF	b3212;b3213;b3214		
CysB-62	3367136	19	yhcE_2F	b4569_2;b3219	insH-10	b3218
CysB-63	3373600	11			nanATEK-yhcH	b3221;b3222;b3223;b3224;b3225
CysB-64	3384357	42	argR	b3237	mdh	b3236
CysB-65	3418320	15	yhdV	b3267		
CysB-66	3558468	9	rtcR	b3422	rtcBA	b4475;b3421
CysB-67	3712010	23			yhjX	b3547
CysB-68	3828710	32	xanP	b3654	gltS	b3653
CysB-69	3859989	39	yidL	b3680	yidKJ	b3678;b3679
CysB-70	3839993	41	yicS	b4555	nlpA	b3661
CysB-71	3867710	17	yidQ	b3688	ibpAB	b3686;b3687
CysB-72	3891605	293	mdtL	b3710		
CysB-73	4077596	8	yihXY-dtd-yiiD	b3885;b3886;b3887;b3888		
CysB-74	4092321	18			frvABXR	b3897;b3898;b3899;b3900
CysB-75	4108796	195	sbp	b3917		
CysB-76	4214123	86	metA	b4013	yjaB	b4012
CysB-77	4270438	8	yjbQR	b4056;b4057		
CysB-78	4337654	5			adiY	b4116
CysB-79	4556320	26			yjiC	b4325

Supplementary Table 4: Computationally-detected novel TF binding sites upstream of i-modulon genes

Identified Motif [a]	I-modulon	Operon	Locus tag(s) in I-modulon	Site p-value	Site start position	Site sequence
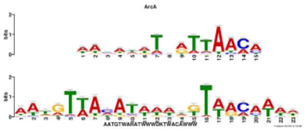	ArcA-1	pdhR-aceEF-lpd	b0113	8.99E-06	121866	ATAGTTTAATAATCGTTAAAAAA
		gcd	b0124	1.17E-05	141244	TCATTAATATTTTAGTAGCAATT
		prpR	b0330	6.83E-06	348483	CTTGTTTCATAATTGTTGCAATG
		ylaC	b0458	1.80E-05	479337	AATTTTGCATAACAGTTGCGAAA
		borD	b0557	7.23E-05	578969	GAAATTTCATATTGTTAATATTT
		rybA-mntS	b4705	8.31E-05	853068	TATATTATATAGCTATTGCTAAA
		ybjM	b0848	4.67E-05	889930	AAAGTAACAATATATTTTACTAG
		dadAX	b1190, b1189	2.72E-05	1237485	CATGTTGAATAATATTTTCAACT
		ydcI	b1422	4.30E-07	1495133	ATTGCAAAATATTATTAACAATT
		trxC	b2582	2.85E-06	2718666	TATGTAACATATTAGAAACATAC
		yqeIJ	b2847, b2848	8.91E-05	2988408	AATATAAAATTAATATATATTTA
		kefGB-yheV	b3351	3.18E-05	3481246	ATCATAATGATAAGTTAACATAG
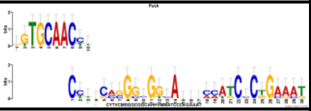	CdaR	dmlA	b1800	3.09E-12	1881730	CCGCCACGGCGGCAAAACCAGCCCGGCGAG
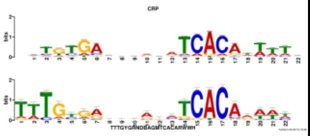	Crp-1	cra	b0080	9.48E-05	87898	TTTGCGAAATCAGTGGGAAC
		yagEF	b0269, b0268	2.61E-05	282110	TTTGGGGAGAGGGCCGGGGT
		ykgEFG	b0306	4.93E-05	321477	TTTGTCATACAAATGAGGGG
		nmpC	b0553_1	4.93E-05	576933	TTTGTGAAGTAGATCTCTAT
		rihA	b0651	4.13E-05	684435	TTTGCGACAAGGGTAACGCC
		ybhQ	b0791	0.00011	824571	TTTTCCGTAAAGTTCGGTAC
		bssR	b0836	5.85E-05	877976	TTTTTCGGACAGCCAAGGGA
		ycaM	b0899	4.52E-05	946929	TTTATCATGATCGCCAGCGT
		rutABCDEFG	b1008	0.000148	1071028	GTTTCGGGATCGGTCACGTT
		ydcJ	b1423	3.45E-05	1495195	TTTCGCGGCGAGATCACAGT
		ydjLKJIHG	b1776	1.15E-05	1861436	TTTACAACTCAGATCACAAT
		yebK	b1853	2.37E-05	1936552	TTTGTCATTAACACGGCACA
		psuKG	b2166	1.76E-05	2259505	TTTGTGACGGGATGCACAGA
		mepS	b2175	1.76E-05	2269849	TTTGTGCGTTAGTCCACAGA
		ygeV	b2869	8.77E-05	3006008	TTTCTGATACTTAGCAGCAA
		bax	b3570	3.78E-05	3737217	TTTTTCACAAATTTGAGAGT
		aphA	b4055	4.05E-06	4269357	TTTGCAACAAATCTCACAAT
		rpiB	b4090	5.37E-05	4313240	TCTGTAAAACAGATCAGGAA
		yjdP	b4487	2.87E-05	4313583	TTTGCCGGAATTCGCGCGGT
		ghoST	b4128	2.37E-05	4352380	TTTTTCATTCTCTCCGCGAT
		ytfJ	b4216	1.19E-06	4439360	TTTGCGGTCAAGCGCACAAA
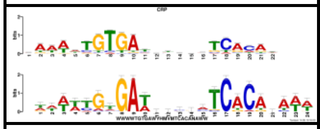	Crp-2	nmpC	b0553_1	1.49E-06	576931	GATTTGTGAAGTAGATCTCTATTT
		ydcH	b1426	1.70E-06	1498553	TAAACACGATCCCGCTCGCATTTT
		ilvC	b3774	2.72E-07	3957902	ATATAGTGAATTCAATCTCGCAAA
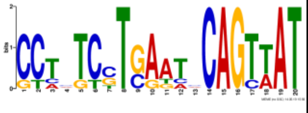	CsqR	ompL	b3875	1.58E-08	4064474	CCCATCGTCAATGCAGCAAT
		yihPO	b3876, b3877	4.32E-08	4067359	CCAGGCGTGGACCCAGTTAT
		yihQ	b3878	4.32E-08	4069515	GTTTTCCTGATTCCAGTAAT
		yihR	b3879	8.56E-08	4070498	CCTATTTTCAGATCAGTTAT
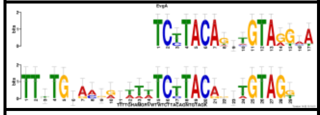	EvgA	ypdI	b2376	6.25E-15	2494486	TTTTGAAAGGGTTATCTTACAGTTGTAGT
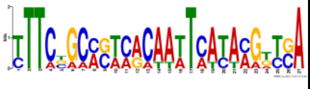	ExuR/FucR	yeiQ	b2172	8.21E-10	2266109	CTTACGCCCTCAATTTTCACTCGTTGA
		exuT	b3093	4.61E-13	3244995	TTTCTGCCGTCGCAAATCATAAGTCGA
		uxaCA	b3091, b3092	4.61E-13	3244995	TTTCTGCCGTCGCAAATCATAAGTCGA

		pgi	b4025	8.86E-10	4233716	CTTCCAAAGTCACAATTCTCAAAATCA
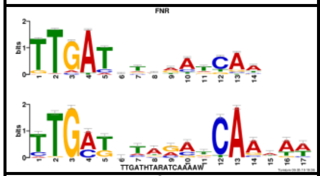	Fnr	abrB	b0715	0.000116	747802	TCGATACAGCATAAAGAA
		ycbJ	b0919	3.54E-05	971518	TTGCTGTTGCTCAGGAAG
		yoeA_1	b4582_1	1.22E-05	2068504	ATGATTAAGGTCAAAAAT
		iscRSUA	b2531	0.000162	2662361	CTGATAAGACGCATTACG
		ttdR	b3060	5.62E-06	3206313	CTGCTCTGGCGCAATATT
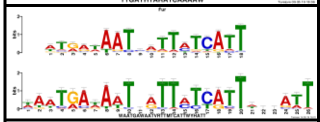	Fur-2	ydhYVWXUT	b1674, b1673	1.73E-08	1754721	AACTGATATTTATTATCATTTGAAAT
		preTA	b2146	1.55E-07	2233859	TATTGAGAATAATTATTACTTCACCT
		bfd-bfr	b3337	8.14E-11	3467020	AAATGAAAATAGTTCTTATTTCAATT
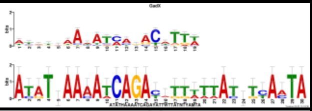	GadEWX	hdeD	b3511	1.34E-13	3656838	TATCAAAATCAGATATTTTTATTTCAAT
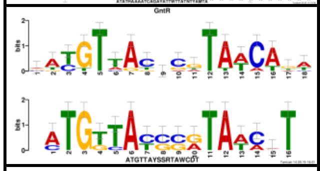	GntR/TyrR	tyrP	b1907	4.83E-07	1989419	CTGGCATGCGTATATT
		fdoGHI-fdhE	b3892, b3893	1.77E-07	4082910	ATGGTATCGATATCTT
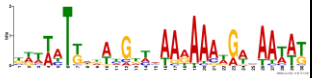	Lrp	ddlA	b0381	6.15E-08	401144	CTTAATTATAAGTTAACGAAGAGAATAT AT
		ydeE	b1534	8.73E-07	1620894	GTTAATGGCACAAAAAAGAAAAGCAAA CTG
		yqeG	b2845	5.88E-09	2985643	TTATCTTAATTGTTTAAAAAAAGTGATTT T
		ilvC	b3774	1.13E-06	3957853	TGCAATGTGACGTTGTGAATATATCAAT TT
		metE	b3829	5.95E-10	4013032	TATAATTAGAGGAAGAAAAAATGACAA TAT
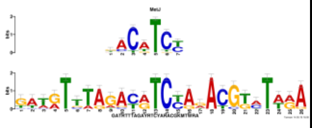	MetJ	mmuPM	b0261, b0260	6.36E-12	275291	GATGTTTAGATGTCCATACGTTTAGA
		hcxA	b0599	1.23E-11	633496	TTTATTTAGACATCTAAACGTCTTGA
		ybdL	b0600	1.23E-11	633496	TTTATTTAGACATCTAAACGTCTTGA
		metJ	b3938	6.36E-12	4128577	GAAGTTTAGATGTCCAGATGTATTGA
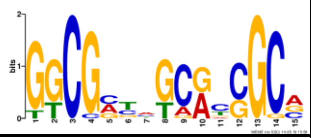	Nac	ybeQ	b0644	2.65E-07	677013	GTCGCCAGCACCGCC
		yrdE_2	b4646_2	1.13E-06	3404182	GGCGGCTGCGTGGCG
		cycA	b4208	6.54E-07	4429477	GGCGAGCGCGGCGCA
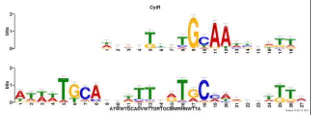	NtrC+RpoN	prpBCDE	b0331	2.76E-06	348546	ATATTTGCGGATTAGTTCATGACTTTA
		ynfM	b1596	8.87E-06	1669583	AATATTCATATTTAAAACATCTTATTT
		asr	b1597	1.64E-07	1671243	TTTATTCAGCGTTTGTACATATCGTTA
		yeaR-yoaG	b1797	1.23E-06	1880014	AATTTGCATTTTAAATACCATTTATTG
		yedL	b1932	5.42E-06	2010566	ATATTGCAAAAATAACACCAATACGGA
		rcnAB	b2106	6.75E-07	2185884	AGATTGCCGAATTAATACTAAGAATTA
		csiD-lhgO-gabDTP	b2660, b2663, b2659	2.76E-06	2788876	AATATGTCGCTTTTGTGCGCATTTTTC
		hypABCDE-fhlA	b2726	5.85E-09	2850467	TTTAAGCATTTTTTGTGCCAACTTTTA
		asnA	b3744	6.18E-07	3927080	TTATTGAATGATTATTGCATGTGTGTC
		zraP	b4002	1.49E-07	4201719	GTATTGCATCTTCCGTGCCAACGATGA
		fdhF	b4079	2.86E-10	4299413	AGTATGCATCTTTTATGCCACATTTTA
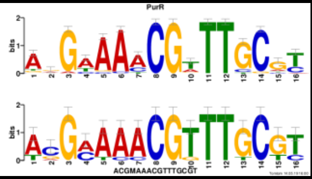	PurR-1	ghxP	b4064	1.27E-08	4278206	ACGATAACGTTTGCGC
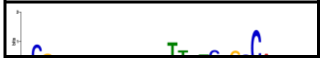	RpoS	rclABC	b0304	2.57E-05	320541	CCGGAAACGCTGTTTTTAGCACCGGTTA
		yahK	b0325	0.000173	342406	TGGTGGACGCCTCCTGTTCCGCCGAGGC

		yahO	b0329	1.66E-05	345995	GCTCATTTGCGGTGTTGACTGGCGGCGC
		phoA-psiF	b0384	0.000122	403311	TCGGTCTGGTTTTTTTAACCACCGTCGG
		ybaY	b0453	5.82E-05	475405	TTAGCGGTTGCGATTGCGTTGGCGGCTT
		ybaA	b0456	1.85E-05	476184	GGGGCGATCCCGCCAGTTTCGCCACATC
		ybdK	b0581	1.95E-06	607307	GCGGATTAACCACCTGCATTTCCAGTTC
		ybeL	b0643	5.09E-07	674491	CCGGAGCGGTGTTGTTTTTCACCGCCGC
		ybgS	b0753	2.86E-05	785787	TCGCCTGTTGGTTGTGATTGGCCCATGC
		ybhP-clsB-ybhN	b0788, b0789, b0790	1.17E-05	824965	CCGGCGGTAGTGTCTTTGCTGGCGAAAG
		ybiO	b0808	2.63E-09	845988	GCATCATTTTCGGCTTCACCGCCAGCGC
		yliI	b0837	0.000159	878155	TCGGCGTGAAGAATTGATCCTGGACAGC
		ycaC	b0897	9.43E-07	946006	TCAGCTCATCCTATTTTACCGGCAACCT
		ycaP	b0906	4.98E-06	956277	CTGGCACATCGTCATTTTCGGTCAGTGG
		ycgX	b1161	9.33E-05	1213607	GCTGCGTATTCAATTAATGCGCCGTCAA
		ycgB	b1188	0.00063	1237442	GCAGAGTCAGGGAGATGTGAGCCAGCT C
		ymgE	b1195	1.48E-05	1244373	CCAGTTTATCCGGGGTTATTGCCAGTAA
		dgcM	b1341	0.000771	1408177	CCACCTTAATTATGTACACCATGATAGC
		ydcK	b1428	5.28E-05	1500848	GTGGCGATTGGTTTTGTTCCGTGGTTTC
		curA	b1449	3.36E-06	1518808	GCAGCACAGTCGGCTTTGCGGGCAAAAC
		mcbR	b1450	0.000381	1519841	GCTGAAAAAACGTATTCGCTTGCAAGGT
		yncG	b1454	1.17E-05	1525939	GCGGCGTGTTGATCTGAAGTGTCGGTGT
		yddH	b1462	6.41E-05	1534308	TTATCAAATCCGCCAGCATTACCGCCGC
		adhP	b1478	1.09E-06	1554120	TCTGAATATCTTTCAGTTCCGGCAGTAC
		ydeI	b1536	0.000588	1624878	CTACCGCCGAATCATGCACCGGCGGTAA
		ydhS	b1668	0.000122	1746925	TCGGCGTTGAGGAAGTTGTCGCCAAAGT
		ldtE	b1678	1.27E-06	1759256	GCAGCAACTTTTCTTTATCCGGCAATAA
		ydiZ	b1724	1.27E-06	1806853	GCTGCATCGTCGACAGTTCTGGCGAAGC
		yniA	b1725	5.82E-05	1807312	CCAGCATTGGGGGAATCATCACCAACCT
		yeaGH	b1784, b1783	0.000548	1866447	GTGCCAGAAGTTTGAGTTTGGTCACAAT
		yeaQ	b1795	1.85E-05	1879342	GCATCACGGAGGTTTTTTTTGCGATGGC
		yebV	b1836	1.32E-05	1921391	GCACAACAAAGGTTATCGCTGGCAGCAT
		yebF	b1847	1.11E-07	1930542	TCAGCACATCTTTTTGTTCTGCCAGCCA
		yodD	b1953	7.75E-05	2024512	GCATAATAAACAGGTTATCTACCATGTC
		yedP	b1955	3.91E-05	2025315	TCACCTTATCCGCAATTTTTTTCGCTGA
		yegP	b2080	3.91E-05	2164982	CTGCCGCATCGTTTAGTTTTAGCGACAT
		yegS	b2086	0.000133	2168608	CTTGAACTGACTTTTTATCCTCCGACTT
		yehE	b2112	5.65E-06	2193243	GCTGTGTATGGTATTTTTCAGCCAGTTT
		yohF	b2137	3.69E-07	2227522	CCTGATGCTGGTTTTTCTCCGCCAGTGT
		yfcG	b2302	8.11E-07	2420079	CCGTTAGTGGCGCTTTTTTCGCCCCCGC
		yfdC	b2347	6.41E-05	2464736	CCTGCAAGAAGTAGTTAACCATCACCGC
		yphA	b2543	5.65E-06	2672839	CCTGCGATCCCGCGATCTTTGCCGGTGG
		yfiL	b2602	0.000204	2741113	CGGTAATATGTACGTTATTCAGCGCGTC
		ygaM	b2672	2.86E-05	2799546	TGGCTACTTCCTCGATTATTGCCGCCGA
		ygdI	b2809	0.000122	2943394	GCGTCTATCTCGACAGCGCCGCGACCGC
		yghA	b3003	1.95E-06	3149323	CCGCCATCGAGAAGATATTCGGCAGTAC
		ygiW	b3024	9.33E-05	3169774	GCGTTACAACACGGTTTACTGGCAGCAA
		yqjG	b3102	1.17E-05	3250766	GCACCACAGCCCTGTTTACTGCGGGCTT
		yhbO	b3153	9.25E-06	3298749	GCGCAGCAATGGCGTTAACTTTCGGTTC
		yhcO	b3239	5.28E-05	3386206	GCGTCGGTTTTGACTTAACTATCGGTCA
		bfd-bfr	b3336	0.000188	3466819	GCAGCTGCATTAATTCATCCTCCATCAC
		ggt	b3447	5.28E-05	3586811	CGGTTTTATCATCGTTATTCTCCAGAGA
		yhjY	b3548	0.000221	3713215	CTGGATTATCGTGGATCACCGTCCTCAA
		ubiCA	b4039	0.00051	4252332	CGGTTGCCCTCGCCAGCACGGGCATCGG
		yjbJ	b4045	4.98E-06	4259046	GCGTTGCGCGGGCTTTCTCTGGCGGCTA
		yjdN	b4107	4.98E-06	4326057	GCGGCGTAAACGCCTTATCCGGCCTACG
		ahr	b4269	8.20E-06	4496165	TCGCCGCCCGCTTCTTTTGCGGCATAGC
		mdtM-yjiN	b4336	2.31E-05	4567453	CCATCAGGATCACCATATTTAGCGATGC
		ytjA	b4568	3.53E-05	4611717	GCGTTGAAGGGGTGACCTCTGTCAGCGA
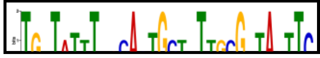	XylR	yiaB	b3563	2.69E-11	3728000	TGGTAGATGCGACTGTTCTAACGGTAGT TG

		yicJI	b3656, b3657	6.27E-12	3836034	TGTTTTATGTGATCGTGGTAGCGTTAATTC
		xylE	b4031	7.29E-11	4242329	TCATTTTTTACAATGCGTTTACGTAATGTC
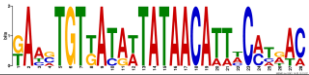	Zinc	pliG	b1178	1.75E-13	1227502	GAACTGTTATAATATAACAATCCCTAAC
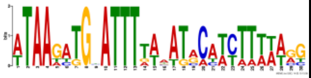	nitrate-related	hcp-hcr-poxB-ltaE-ybjT	b0872, b0873	4.49E-12	913876	ATAAAAGGGATTTTTCATGCAACTTTAAGG
		fdnGHI	b1476, b1474, b1475	4.78E-10	1547201	TTAAGCTGAATTTTATAGCATTTTTTTAAC
		yeaR-yoaG	b1796, b1797	1.36E-11	1880012	ATAATTTGCATTTTAAATACCATTTATTGG
		hmp	b2552	4.10E-12	2685783	ATAAGATGCATTTGAGATACATCAATTAAG
		ygbA	b2732	5.60E-11	2856821	TTAAGATGTATTTATATTACATCTTAATCT
		ytfE	b4209	4.42E-17	4432007	ATAAGATGTATTTTAAATGCATCTTTAAGG

[a]Motifs were identified by searching all upstream sequences of genes in an i-modulon, and compared to reported motifs in RegulonDB (See Methods). If a match was found, the top motif is the RegulonDB motif, and the bottom motif is the motif found upstream of i-modulon genes.

Supplementary Table 5: I-modulon membership for uncharacterized genes.
(as defined by Ghatak et al. 2018[28]. Additional annotations are derived from i-modulon membership)

b-number	Gene Name	I-modulon	Current Annotation from Ecocyc	Additional annotation from i-modulon membership
b0511	ybbW	AllR/AraC/FucR	putative allantoin transporter	
b0975	hyaD	ArcA-1	putative hydrogenase 1 maturation protease HyaD	
b0976	hyaE	ArcA-1	putative HyaA chaperone	
b0977	hyaF	ArcA-1	protein HyaF	
b2725	hycA	ArcA-1	regulator of the transcriptional regulator FhlA	
b2998	yghW	ArcA-1	DUF2623 domain-containing protein YghW	
b0458	ylaC	ArcA-2	putative inner membrane protein	
b0848	ybjM	ArcA-2	putative inner membrane protein	
b2181	yejG	ArcA-2	protein YejG	
b2847	yqeI	ArcA-2	putative transcriptional regulator YqeI	
b3820	yigI	ArcA-2	putative thioesterase YigI	
b2221	atoD	AtoC	acetyl-CoA:acetoacetyl-CoA transferase subunit α	
b2223	atoE	AtoC	short chain fatty acid transporter	
b2788	gudX	CdaR	glucarate dehydratase-related protein	
b2789	gudP	CdaR	galactarate/glucarate/glycerate transporter GudP	
b0795	ybhG	CecR	HlyD_D23 family protein YbhG	
b0287	yagU	CpxR	inner membrane protein that contributes to acid resistance	
b0461	tomB	CpxR	protein that modulates Hha toxicity	
b1843	yobB	CpxR	putative carbon-nitrogen hydrolase family protein YobB	
b1846	yebE	CpxR	conserved inner membrane protein YebE	
b4188	yjfN	CpxR	protease activator	
b2166	psuK	Cra	putative pseudouridine kinase	
b3875	ompL	CsqR	putative outer membrane porin L	
b3877	yihP	CsqR	putative 2,3-dihydroxypropane-1-sulfonate export protein	
b3879	yihR	CsqR	putative aldose 1-epimerase YihR	
b3884	yihW	CsqR	putative DNA-binding transcriptional regulator YihW	
b1287	yciW	CysB	putative oxidoreductase	
b2012	yeeD	CysB,membrane	putative sulfurtransferase YeeD	
b2013	yeeE	CysB,membrane	inner membrane protein YeeE	
b1466	narW	EvgA	NarW, putative private chaperone for NarZ nitrate reductase subunit	
b1501	ydeP	EvgA	putative oxidoreductase YdeP	
b2082	ogrK	EvgA	prophage P2 late control protein OgrK	
b2085	yegR	EvgA	uncharacterized protein YegR	
b2367	emrY	EvgA	tripartite efflux pump membrane subunit EmrY	
b2368	emrK	EvgA	tripartite efflux pump membrane fusion protein EmrK	
b2372	yfdV	EvgA	putative transport protein YfdV	
b2375	yfdX	EvgA	protein YfdX	
b2376	ypdI	EvgA	colanic acid synthesis putative lipoprotein YpdI	
b2172	yeiQ	ExuR/FucR	putative dehydrogenase, NAD-dependent	
b0557	borD	FadR/IclR	DLP12 prophage; prophage lipoprotein BorD	Related to fatty acid degradation
b4315	fimI	fimbriae	putative fimbrial protein FimI	
b1044	ymdA	FlhDC	uncharacterized protein YmdA	Flagellar protein
b1072	flgA	FlhDC	flagellar basal body P-ring formation protein FlgA	
b1075	flgD	FlhDC	flagellar biosynthesis, initiation of hook assembly	
b1081	flgJ	FlhDC	putative peptidoglycan hydrolase FlgJ	
b1878	flhE	FlhDC	flagellar protein	
b1904	yecR	FlhDC	lipoprotein YecR	
b1943	fliK	FlhDC	flagellar hook-length control protein	
b1566	flxA	FliA	Qin prophage; protein FlxA	Chemotaxis protein
b1742	ves	FliA	HutD family protein Ves	Chemotaxis protein
b1760	ynjH	FliA	DUF1496 domain-containing protein YnjH	Chemotaxis protein
b4110	yjcZ	FliA	uncharacterized protein YjcZ	Chemotaxis protein
b1070	flgN	FliA,FlhDC	flagellar biosynthesis protein FlgN	
b1925	fliS	FliA,FlhDC	flagellar biosynthesis protein FliS	
b1926	fliT	FliA,FlhDC	flagellar biosynthesis protein FliT	
b0836	bssR	Fnr	regulator of biofilm formation	
b0919	ycbJ	Fnr	putative phosphotransferase YcbJ	
b1541	ydfZ	Fnr	putative selenoprotein YdfZ	

b1587	ynfE	Fnr	putative selenate reductase YnfE	
b1593	ynfK	Fnr	putative dethiobiotin synthetase	
b1750	ydjX	Fnr	DedA family protein YdjX	
b1751	ydjY	Fnr	4Fe-4S ferredoxin-type domain-containing protein YdjY	
b1785	yeaI	Fnr	putative c-di-GMP binding protein CdgI	
b1906	yecH	Fnr	DUF2492 domain-containing protein YecH	
b3158	yhbU	Fnr	putative peptidase YhbU	
b3159	yhbV	Fnr	putative peptidase YhbV	
b3211	yhcC	Fnr	radical SAM family oxidoreductase YhcC	
b1256	ompW	Fnr,ArcA-1	outer membrane protein W	
b4380	yjjI	Fnr,SoxS	DUF3029 domain-containing protein YjjI	
b0587	fepE	Fur-1	polysaccharide co-polymerase family protein FepE	
b0803	ybiI	Fur-1	zinc finger domain-containing protein YbiI	
b0804	ybiX	Fur-1	PKHD-type hydroxylase YbiX	
b1494	pqqL	Fur-1	putative zinc peptidase	
b1496	yddA	Fur-1	ABC transporter family protein YddA	
b1705	ydiE	Fur-1	PF10636 family protein YdiE	
b1452	yncE	Fur-1,Fur-2	PQQ-like domain-containing protein YncE	
b4567	yjjZ	Fur-1,iron-related	protein YjjZ	
b1495	yddB	Fur-1,membrane	putative TonB-dependent receptor	
b0468	ybaN	Fur-2	conserved inner membrane protein YbaN	
b1673	ydhV	Fur-2	putative oxidoreductase	
b1674	ydhY	Fur-2	putative 4Fe-4S ferredoxin-type protein	
b3337	bfd	Fur-2	bacterioferritin-associated ferredoxin	
b3491	yhiM	GadEWX	inner membrane protein with a role in acid resistance	
b3508	yhiD	GadEWX	inner membrane protein YhiD	Acid resistance protein
b3511	hdeD	GadEWX	acid-resistance membrane protein	
b3507	dctR	GadEWX,GadWX	putative DNA-binding transcriptional regulator DctR	
b0486	ybaT	GadWX	putative transporter YbaT	Acid resistance protein
b1426	ydcH	GatR/GalRS	protein YdcH	Acid resistance protein
b2977	glcG	GlcC	putative heme-binding protein GlcG	
b2979	glcD	GlcC	glycolate dehydrogenase, putative FAD-linked subunit	
b4467	glcF	GlcC	glycolate dehydrogenase, putative iron-sulfur subunit	
b4468	glcE	GlcC	glycolate dehydrogenase, putative FAD-binding subunit	
b1137	ymfD	lipopolysaccharide	e14 prophage; putative SAM-dependent methyltransferase	Lipopolysaccharide-related protein
b1457	ydcD	lipopolysaccharide	uncharacterized protein YdcD	Lipopolysaccharide-related protein
b1550	gnsB	lipopolysaccharide	Qin prophage; protein GnsB	Lipopolysaccharide-related protein
b2032	wbbK	lipopolysaccharide	putative lipopolysaccharide biosynthesis protein	
b2035	wbbH	lipopolysaccharide	putative O-antigen polymerase	
b2352	gtrS	lipopolysaccharide	CPS-53 (KpLE1) prophage; serotype specific glucosyl transferase	
b2642	yfjW	lipopolysaccharide	CP4-57 prophage; uncharacterized protein YfjW	Lipopolysaccharide-related protein
b3618	yibB	lipopolysaccharide	protein HtrL	Lipopolysaccharide-related protein
b4253	yjgL	lipopolysaccharide	protein YjgL	Lipopolysaccharide-related protein
b1534	ydeE	Lrp	dipeptide exporter	
b1605	ydgI	Lrp	putative arginine:ornithine antiporter	
b2845	yqeG	Lrp	putative transporter YqeG	Amino acid or peptide transporter
b4045	yjbJ	Lrp	putative stress response protein	
b3523	yhjE	Lrp,Nac	putative transporter YhjE	Amino acid or peptide transporter
b4037	malM	MalT	maltose regulon periplasmic protein	
b2971	yghG	membrane	lipoprotein YghG	
b2972	pppA	membrane	prepilin peptidase	
b3151	yraQ	membrane	permease family protein YraQ	
b4466	sslE	membrane	putative lipoprotein YghJ	
b3937	yiiX	MetJ	putative lipid binding hydrolase	Methionine or folate related hydrolase
b0644	ybeQ	Nac	Sel1 repeat-containing protein YbeQ	Nitrogen starvation protein
b1339	abgR	Nac	putative LysR-type DNA-binding transcriptional regulator AbgR	
b1441	ydcT	Nac	putative ABC transporter ATP-binding protein YdcT	Putative peptide transporter
b1442	ydcU	Nac	putative ABC transporter membrane subunit YdcU	Putative peptide transporter
b1443	ydcV	Nac	putative ABC transporter membrane subunit YdcV	Putative peptide transporter
b3043	ygiL	Nac	putative fimbrial protein YgiL	Nitrogen starvation protein
b3446	yrhB	Nac	putative heat shock chaperone	Nitrogen starvation protein

b1497	ydeM	NagC/TyrR	putative anaerobic sulfatase maturation enzyme YdeM	
b1498	ydeN	NagC/TyrR	putative sulfatase	
b2111	yehD	NarL	putative fimbrial protein YehD	Nitrate-related protein
b4072	nrfC	NarL	putative menaquinol-cytochrome <i>c</i> reductase 4Fe-4S subunit	
b4073	nrfD	NarL	putative menaquinol-cytochrome <i>c</i> reductase subunit NrfD	
b2208	napF	NarL,Fnr	ferredoxin-type protein	
b0208	yafC	NikR	putative LysR family transcriptional regulator YafC	
b1796	yoaG	nitrate-related	DUF1869 domain-containing protein YoaG	Nitrate-related protein
b1797	yeaR	nitrate-related	DUF1971 domain-containing protein YeaR	Nitrate-related protein
b2732	ygbA	nitrate-related	protein YgbA	Nitrate-related protein
b1008	rutE	NtrC+RpoN	putative malonic semialdehyde reductase	
b1009	rutD	NtrC+RpoN	putative aminoacrylate hydrolase	
b1010	rutC	NtrC+RpoN	putative aminoacrylate peracid reductase	
b1483	ddpF	NtrC+RpoN	putative D,D-dipeptide ABC transporter ATP-binding subunit DdpF	
b1484	ddpD	NtrC+RpoN	putative D,D-dipeptide ABC transporter ATP-binding subunit DdpD	
b1485	ddpC	NtrC+RpoN	putative D,D-dipeptide ABC transporter membrane subunit DdpC	
b1486	ddpB	NtrC+RpoN	putative D,D-dipeptide ABC transporter membrane subunit DdpB	
b1487	ddpA	NtrC+RpoN	putative D,D-dipeptide ABC transporter periplasmic binding protein	
b1596	ynfM	NtrC+RpoN	putative transporter YnfM	
b2870	ygeW	NtrC+RpoN	putative carbamoyltransferase YgeW	
b2875	yqeB	NtrC+RpoN	XdhC-CoxI family protein YqeB	
b2876	yqeC	NtrC+RpoN	uncharacterized protein YqeC	Nitrogen starvation protein
b3269	yhdX	NtrC+RpoN	putative ABC transporter membrane subunit YhdX	Putative peptide transporter
b3270	yhdY	NtrC+RpoN	putative ABC transporter membrane subunit YhdY	Putative peptide transporter
b3271	yhdZ	NtrC+RpoN	putative ABC transporter ATP-binding subunit YhdZ	Putative peptide transporter
b1932	yedL	NtrC+RpoN,Nac	putative acetyltransferase YedL	
b1660	ydhC	PurR-1	putative transporter YdhC	Purine/purine precursor efflux pump
b2313	cvpA	PurR-1	colicin V production protein	Purine biosynthetic protein
b3547	yhjX	Pyruvate	putative pyruvate transporter	
b4353	yjiX	Pyruvate	conserved protein YjiX	Pyruvate-responsive protein
b2044	wcaL	RcsAB	putative colanic biosynthesis glycosyl transferase	
b2045	wcaK	RcsAB	putative colanic acid biosynthesis pyruvyl transferase	
b2046	wzxC	RcsAB	G7097-MONOMER	
b2050	wcaI	RcsAB	putative colanic biosynthesis glycosyl transferase	
b2054	wcaF	RcsAB	putative acyl transferase	
b2055	wcaE	RcsAB	putative colanic acid biosynthesis glycosyl transferase	
b2056	wcaD	RcsAB	putative colanic acid polymerase	
b2057	wcaC	RcsAB	putative colanic acid biosynthesis glycosyl transferase	
b2058	wcaB	RcsAB	putative colanic acid biosynthesis acyl transferase	
b2059	wcaA	RcsAB	putative colanic acid biosynthesis glycosyl transferase	
b4026	yjbE	RcsAB	uncharacterized protein YjbE	Colanic acid-related protein
b4027	yjbF	RcsAB	lipoprotein YjbF	
b4028	yjbG	RcsAB	capsule biosynthesis GfcC family protein YjbG	
b1321	ycjX	RpoH	DUF463 domain-containing protein YcjX	Chaperone-related protein
b4140	fxsA	RpoH	protein FxsA	Chaperone-related protein
b0329	yahO	RpoS	DUF1471 domain-containing protein YahO	Stress-related protein
b0453	ybaY	RpoS	PF09619 family lipoprotein YbaY	Stress-related protein
b0707	ybgA	RpoS	DUF1722 domain-containing protein YbgA	Stress-related protein
b0788	ybhN	RpoS	conserved inner membrane protein YbhN	Stress-related protein
b0790	ybhP	RpoS	endonuclease/exonuclease/phosphatase domain-containing protein YbhP	Stress-related protein
b0897	ycaC	RpoS	putative hydrolase	Stress-related protein
b1003	yccJ	RpoS	PF13993 family protein YccJ	Stress-related protein
b1051	msyB	RpoS	acidic protein that suppresses heat sensitivity of a <i>secY</i> mutant	Stress-related protein
b1188	ycgB	RpoS	PF04293 family protein YcgB	Stress-related protein
b1195	ymgE	RpoS	PF04226 family protein YmgE	Stress-related protein

b1536	ydeI	RpoS	BOF family protein YdeI	Stress-related protein
b1668	ydhS	RpoS	FAD/NAD(P) binding domain-containing protein YdhS	Stress-related protein
b1784	yeaH	RpoS	DUF444 domain-containing protein YeaH	Stress-related protein
b1836	yebV	RpoS	protein YebV	Stress-related protein
b1953	yodD	RpoS	stress-induced protein	Stress-related protein
b2080	yegP	RpoS	DUF1508 domain-containing protein YegP	Stress-related protein
b2266	elaB	RpoS	tail anchored inner membrane protein	Stress-related protein
b2543	yphA	RpoS	putative inner membrane protein	Stress-related protein
b2672	ygaM	RpoS	DUF883 domain-containing protein YgaM	Stress-related protein
b3100	yqjK	RpoS	conserved protein YqjK	Stress-related protein
b3239	yhcO	RpoS	putative barnase inhibitor	Stress-related protein
b3555	yiaG	RpoS	putative DNA-binding transcriptional regulator YiaG	Stress-related protein
b4107	yjdN	RpoS	conserved protein YjdN	Stress-related protein
b4149	blc	RpoS	outer membrane lipoprotein Blc	Stress-related protein
b4568	ytjA	RpoS	DUF1328 domain-containing protein YtjA	Stress-related protein
b0389	yaiA	SoxS	protein YaiA	Oxidative stress-related protein
b0448	mdlA	SoxS	ABC transporter family protein MdlA	
b0449	mdlB	SoxS	ABC transporter family protein MdlB	
b0850	ybjC	SoxS	DUF1418 domain-containing protein YbjC	Oxidative stress-related protein
b1580	rspB	SoxS	putative zinc-binding dehydrogenase RspB	
b1581	rspA	SoxS	mandelate racemase/muconate lactonizing enzyme family protein RspA	
b2160	yeiI	SoxS	putative sugar kinase YeiI	
b2237	inaA	SoxS	putative lipopolysaccharide kinase InaA	
b2962	yggX	SoxS	putative Fe²⁺-trafficking protein	
b3238	yhcN	SoxS	DUF1471 domain-containing stress-induced protein YhcN	
b3898	frvX	SoxS	peptidase M42 family protein	
b4536	yobH	SoxS	protein YobH	Oxidative stress-related protein
b0270	yagG	XylR	putative D-xylonate transporter YagG	
b0271	yagH	XylR	CP4-6 prophage; putative xylosidase/arabinosidase	
b3562	yiaA	XylR	conserved inner membrane protein YiaA	Putative xylose or xyloside transporter
b3657	yicJ	XylR	putative xyloside transporter YicJ	
b3563	yiaB	XylR,RcsAB	conserved inner membrane protein YiaB	Putative xylose or xyloside transporter
b2736	ygbJ	YgbI	putative L-threonate dehydrogenase	
b2737	ygbK	YgbI	putative 3-oxo-tetronate kinase YgbK	
b2738	ygbL	YgbI	putative 3-oxo-tetronate 4-phosphate decarboxylase YgbL	
b2739	ygbM	YgbI	putative 2-oxo-tetronate isomerase YgbM	
b2740	ygbN	YgbI	putative transporter YgbN	
b3576	yiaL	YiaJ	DUF386 domain-containing protein YiaL	
b3582	sgbU	YiaJ	putative L-xylulose 5-phosphate 3-epimerase	
b0306	ykgE	YieP	putative lactate utilization oxidoreductase YkgE	
b0307	ykgF	YieP	putative amino acid dehydrogenase with NAD(P)-binding domain and ferridoxin-like domain	
b0308	ykgG	YieP	DUF162 domain-containing lactate utilization protein YkgG	
b2141	yohJ	YieP	PF03788 family membrane protein YohJ	
b2142	yohK	YieP	PF04712 family membrane protein YohK	
b3755	yieP	YieP	putative transcriptional regulator YieP	
b1523	yneG	YneJ	conserved protein YneG	
b1526	yneJ	YneJ	putative LysR-type DNA-binding transcriptional regulator YneJ	
b0296	ykgM	Zinc	putative ribosomal protein	Zinc-related protein
b1974	yodB	Zinc	putative cytochrome	Zinc-related protein
b4506	ykgO	Zinc	putative ribosomal protein	Zinc-related protein

Supplementary Table 6: Mutated regulators in evolved strains.

ALEdb ID [a]	Mutations in Transcriptional Regulators	Activated I-modulons	Mean Activity Level
FPS_thrA.A1.F5.I1	Intergenic SNP between metJ and metB		
FPS_thrA.A2.F1.I0	SNP in MetJ (V46E)	MetJ	7
FPS_serB.A1.F5.I1	SNP in leader region of his operon	His-tRNA	13
FPS_serB.A4.F1.I0	SNP in His-tRNA encoding gene hisR	His-tRNA	14
FPS_ptsI.A1.F5.I1	SNP in Crp (A145V)		
FPS_ptsI.A3.F1.I0	SNP in Crp (T141P)		
PAL.A2.F6.I1	Δ2 bp in CsqR resulting in truncation	CsqR	36
PAL.A2.F73.I0	Δ1 bp in CsqR resulting in truncation	CsqR	33
		duplication-1	25
PAL.A4.F90.I0	SNP in CsqR (I156S)	CsqR	33
		duplication-1	25
PAL.A10.F18.I1	IS2 insertion in RbsR coding region	RbsR	28
PAL.A10.F118.I0	100kb deletion including TFs: AbgR, RacR, FeaR, PaaX, YdcI, YdcR, McbR, YddM, YdeO	deletion-2	-41
	IS2 insertion in RbsR coding region	RbsR	19
PAL.A14.F9.I1	SNP in FucR (D82Y)	AllR/AraC/FucR	10
		ExuR/FucR	31
PAL.A14.F78.I0	Δ6 bp in AraC	AllR/AraC/FucR	25
	SNP in FucR (S75R)	AllR/AraC/FucR	25
		ExuR/FucR	24
PAL.A16.F70.I0	SNP in AraC (L156I)	AllR/AraC/FucR	31
	SNP in FucR (S75R)	AllR/AraC/FucR	31
		ExuR/FucR	23
	SNP in FucR (*244C)	AllR/AraC/FucR	31
		ExuR/FucR	23
PAL.A26.F10.I1	36 kb deletion including TFs: KdgR, YebK	deletion-1	-29
PAL.A26.F86.I0	36 kb deletion including TFs: KdgR, YebK	deletion-1	-51
	2bp substitution in YgbI	YgbI	15
PAL.A28.F10.I1	Intergenic SNP between dmlR and dmlA		
	SNP in YgbI (R147W)	YgbI	23
PAL.A28.F88.I0	Intergenic SNP between dmlR and dmlA		
	Δ20 bp in YgbI resulting in truncation	YgbI	23
EEP_menF_entC.A39.F91.I1			
EEP_menF_entC.A29.F82.I1	SNP in OxyR (A204E)	OxyR	21
EEP_menF_entC.A30.F83.I1	SNP in OxyR (A147E)	OxyR	9
EEP_menF_entC_ubiC.A36.F86.I1	SNP in YneJ (I226F)	YneJ	20
EEP_menF_entC_ubiC.A37.F25.I1			
EEP_menF_entC_ubiC.A38.F28.I1			

[a] Strain IDs follow identification tabs in ALEdb[29] (https://aledb.org/)

Supplementary Table 7: Genes in the BW25113 i-modulon

I-modulon Gene/Operon	I-modulon Gene Coefficient(s)	Genotypic Change	Putative Phenotypic Explanation
araBAD	-0.19; -0.25; -0.12	Deletion of *araBA*, Truncation of *araD*	No expression in BW25113
rhaA	-0.11; -0.08	Deletion	No expression in BW25113
lacZ	-0.14	Truncation	Low expression in BW25113
hsdR	-0.09	Truncation	Low expression in BW25113
yjjB	-0.10	Truncation	Low expression in BW25113
mhpCDEF	0.19; 0.28; 0.18; 0.21	Insertion of IS30 in *mhpC*	IS30 contains internal promoter[30] leading to increased expression in BW25113
tabA-ygjL	0.32; 0.22	SNP at transcription start site (A→G. 4,474,834)	Increased expression in BW25113
fabAB	0.09; 0.11	SNP in FabR	Decreased repression of *fabAB* by FabR
pheA	0.10	SNP in *pheV* tRNA	Altered tRNA-mediated attenuation via phe-tRNA

Supplementary Table 8: Primers used in this study

Primer names	Sequence
cysB_tag_forward	GCATTGCGCTCTAATGAAGAAATTGAGGTCATGTTTAAAGATATAAAACTGCCG GAAAAAGTCGGATCCAGTCTTCGTGAT
cysB_tag_reverse	AAAAGAGAATATATTCCGGCACCTTCGCTACATAAAAGGTGCCGAAAATAACG CAAGAAAAATTCCGGGGATCCGTCGACC
cysB_conf_forward	GCGTGTTGATGCTCACGATA
cysB_conf_reverse	CGAGGCGGGTAATTAGACACT
metJ_tag_forward	CCGGAAGCGGCAAAAGAGATCATGCGTGAGATGGGGATTAACCCGGAGACGTG GGAATACGTCGGATCCAGTCTTCGTGAT
metJ_tag_reverse	ACGTAGGCCTGATAAGCGTAGCGCATCAGGCGATTCCACTCCGCGCCGCTCTTT TTTGCTAATTCCGGGGATCCGTCGACC
metJ_conf_forward	ACAACCTGCGTCACGCTAC
metJ_conf_reverse	TTATCCGGCCTACAAGTTCG
PCR Enrichment Non-Indexed Oligo	AATGATACGGCGACCACCGAGAT
PCR Enrichment Indexed Oligo	CAAGCAGAAGACGGCATACGAGATatccggcGTGACTGGAGTTCAGACGTGTGCTC TTCCGATCT

Supplementary References

1. Faith, J. J. *et al.* Large-scale mapping and validation of Escherichia coli transcriptional regulation from a compendium of expression profiles. *PLoS Biol.* **5**, e8 (2007).
2. Santos-Zavaleta, A. *et al.* A unified resource for transcriptional regulation in Escherichia coli K-12 incorporating high-throughput-generated binding data into RegulonDB version 10.0. *BMC Biol.* **16**, 91 (2018).
3. Baneyx, F. & Mujacic, M. Recombinant protein folding and misfolding in Escherichia coli. *Nat. Biotechnol.* **22**, 1399–1408 (2004).
4. Guzmán, G. I. *et al.* Enzyme promiscuity shapes adaptation to novel growth substrates. *Mol. Syst. Biol.* **15**, e8462 (2019).
5. Ploeg, J. R. van der *et al.* Identification of sulfate starvation-regulated genes in Escherichia coli: a gene cluster involved in the utilization of taurine as a sulfur source. *Journal of Bacteriology* **178**, 5438–5446 (1996).
6. Kim, D. *et al.* Systems assessment of transcriptional regulation on central carbon metabolism by Cra and CRP. *Nucleic Acids Res.* **46**, 2901–2917 (2018).
7. Latif, H. *et al.* ChIP-exo interrogation of Crp, DNA, and RNAP holoenzyme interactions. *PLoS One* **13**, e0197272 (2018).
8. Nishino, K., Inazumi, Y. & Yamaguchi, A. Global analysis of genes regulated by EvgA of the two-component regulatory system in Escherichia coli. *J. Bacteriol.* **185**, 2667–2672 (2003).
9. Stiefel, A. *et al.* Control of the ferric citrate transport system of Escherichia coli: mutations in region 2.1 of the FecI extracytoplasmic-function sigma factor suppress mutations in the FecR transmembrane regulatory protein. *J. Bacteriol.* **183**, 162–170 (2001).
10. Seo, S. W. *et al.* Deciphering Fur transcriptional regulatory network highlights its complex role beyond iron metabolism in Escherichia coli. *Nat. Commun.* **5**, 4910 (2014).
11. McHugh, J. P. *et al.* Global iron-dependent gene regulation in Escherichia coli. A new mechanism for iron homeostasis. *J. Biol. Chem.* **278**, 29478–29486 (2003).
12. McCloskey, D. *et al.* Multiple Optimal Phenotypes Overcome Redox and Glycolytic Intermediate Metabolite Imbalances in Escherichia coli pgi Knockout Evolutions. *Appl. Environ. Microbiol.* **84**, (2018).
13. Sandberg, T. E. *et al.* Evolution of Escherichia coli to 42 °C and subsequent genetic engineering reveals

adaptive mechanisms and novel mutations. *Mol. Biol. Evol.* **31**, 2647–2662 (2014).

14. Turnbough, C. L., Jr & Switzer, R. L. Regulation of pyrimidine biosynthetic gene expression in bacteria: repression without repressors. *Microbiol. Mol. Biol. Rev.* **72**, 266–300, table of contents (2008).
15. Behr, S. *et al.* Identification of a High-Affinity Pyruvate Receptor in Escherichia coli. *Sci. Rep.* **7**, 1388 (2017).
16. Torres-Cabassa, A. S. & Gottesman, S. Capsule synthesis in Escherichia coli K-12 is regulated by proteolysis. *J. Bacteriol.* **169**, 981–989 (1987).
17. Chang, Y. Y., Wang, A. Y. & Cronan, J. E., Jr. Expression of Escherichia coli pyruvate oxidase (PoxB) depends on the sigma factor encoded by the rpoS(katF) gene. *Mol. Microbiol.* **11**, 1019–1028 (1994).
18. Chiang, S. M., Dong, T., Edge, T. A. & Schellhorn, H. E. Phenotypic diversity caused by differential RpoS activity among environmental Escherichia coli isolates. *Appl. Environ. Microbiol.* **77**, 7915–7923 (2011).
19. Gao, Y. *et al.* Systematic discovery of uncharacterized transcription factors in Escherichia coli K-12 MG1655. *Nucleic Acids Res.* (2018). doi:10.1093/nar/gky752
20. Anand, A. *et al.* Pseudogene repair driven by selection pressure applied in experimental evolution. *Nat Microbiol* (2019). doi:10.1038/s41564-018-0340-2
21. Mehta, P., Casjens, S. & Krishnaswamy, S. Analysis of the lambdoid prophage element e14 in the E. coli K-12 genome. *BMC Microbiol.* **4**, 4 (2004).
22. Jensen, K. F. The Escherichia coli K-12‘ wild types’ W3110 and MG1655 have an rph frameshift mutation that leads to pyrimidine starvation due to low pyrE expression levels. *J. Bacteriol.* **175**, 3401–3407 (1993).
23. Lloyd, C. R., Park, S., Fei, J. & Vanderpool, C. K. The Small Protein SgrT Controls Transport Activity of the Glucose-Specific Phosphotransferase System. *J. Bacteriol.* **199**, (2017).
24. Huerta-Cepas, J. *et al.* eggNOG 4.5: a hierarchical orthology framework with improved functional annotations for eukaryotic, prokaryotic and viral sequences. *Nucleic Acids Res.* **44**, D286–93 (2016).
25. Oliphant, T. E. Python for Scientific Computing. *Computing in Science Engineering* **9**, 10–20 (2007).
26. Bailey, T. L. *et al.* MEME SUITE: tools for motif discovery and searching. *Nucleic Acids Res.* **37**, W202–8 (2009).
27. Gupta, S., Stamatoyannopoulos, J. A., Bailey, T. L. & Noble, W. S. Quantifying similarity between motifs. *Genome Biol.* **8**, R24 (2007).
28. Ghatak, S., King, Z. A., Sastry, A. & Palsson, B. O. The y-ome defines the 35% of Escherichia coli genes that

lack experimental evidence of function. *Nucleic Acids Res.* **47**, 2446–2454 (2019).

29. Phaneuf, P. V., Gosting, D., Palsson, B. O. & Feist, A. M. ALEdb 1.0: a database of mutations from adaptive laboratory evolution experimentation. *Nucleic Acids Res.* **47**, D1164–D1171 (2019).
30. Dalrymple, B. & Arber, W. Promotion of RNA transcription on the insertion element IS30 of E. coli K12. *EMBO J.* **4**, 2687–2693 (1985).
