# Supplementary material for: The Escherichia coli transcriptome mostly consists of independently regulated modules
Source: Nat Commun. 2019 Dec 4;10:5536. doi: 10.1038/s41467-019-13483-w (PMC6892915; doi:10.1038/s41467-019-13483-w)
Supplement: Supplementary file 3 — Description of Additional Supplementary Files [file 41467_2019_13483_MOESM3_ESM.pdf]

Description of Additional Supplementary Files

File Name: Supplementary Data 1
Description: Expression levels, experimental conditions, and i-modulon decomposition of the PRECISE compendium.

File Name: Supplementary Data 2
Description: Descriptive characteristics of the 92 i-modulons.
