# Supplementary material for: The Escherichia coli transcriptome mostly consists of independently regulated modules
Source: Nat Commun. 2019 Dec 4;10:5536. doi: 10.1038/s41467-019-13483-w (PMC6892915; doi:10.1038/s41467-019-13483-w)
Supplement: Supplementary file 5 — Supplementary Data 2 [file 41467_2019_13483_MOESM5_ESM.pdf]

AllR/AraC/FucR I-Modulon

Regulated by: AllR or AraC or FucR

Biological Function: Allantoin, glyoxylate, L-arabinose, and L-fucose catabolism

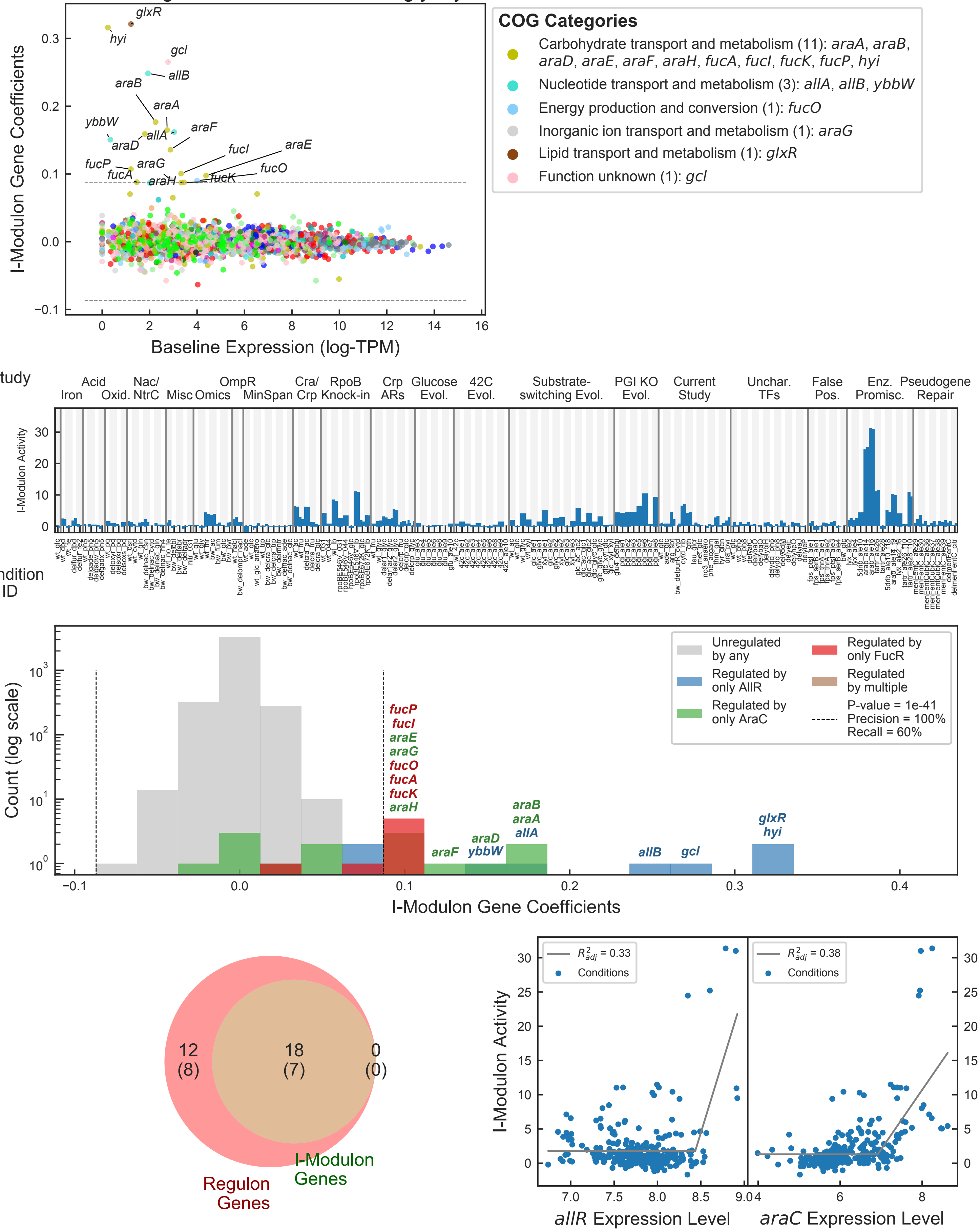

ArcA – 1 I-Modulon

Regulated by: ArcA

Biological Function: Anaerobic response

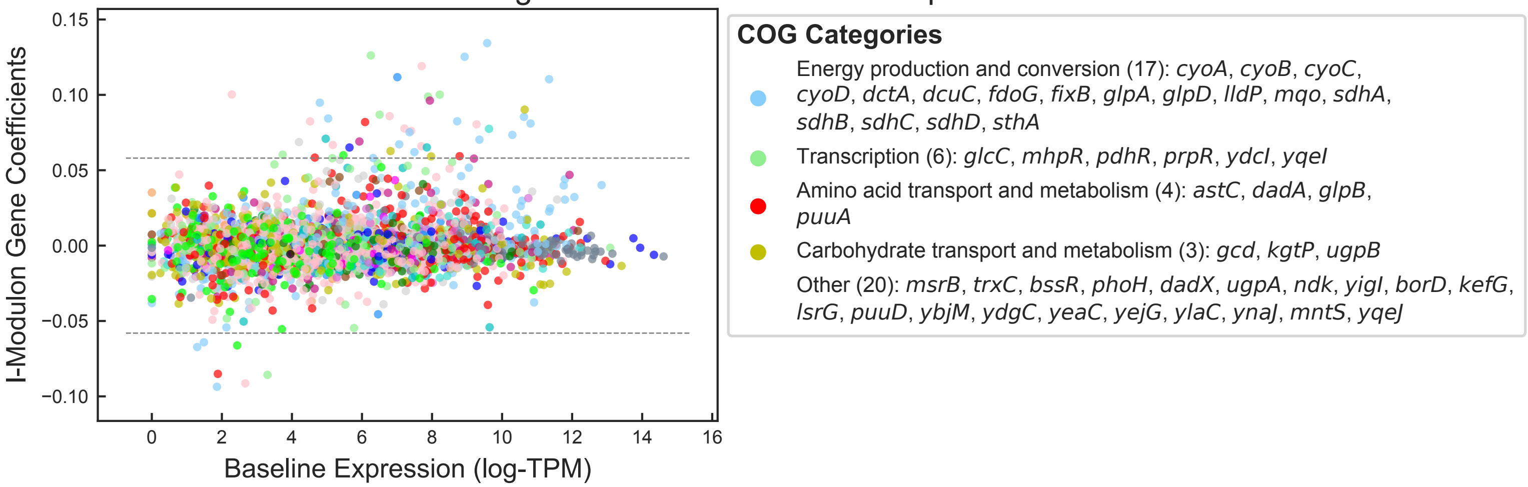

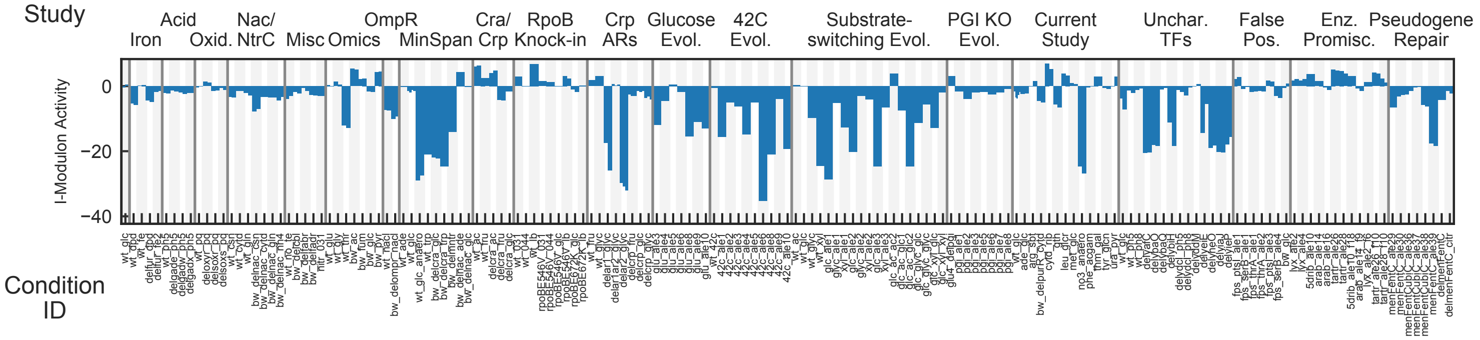

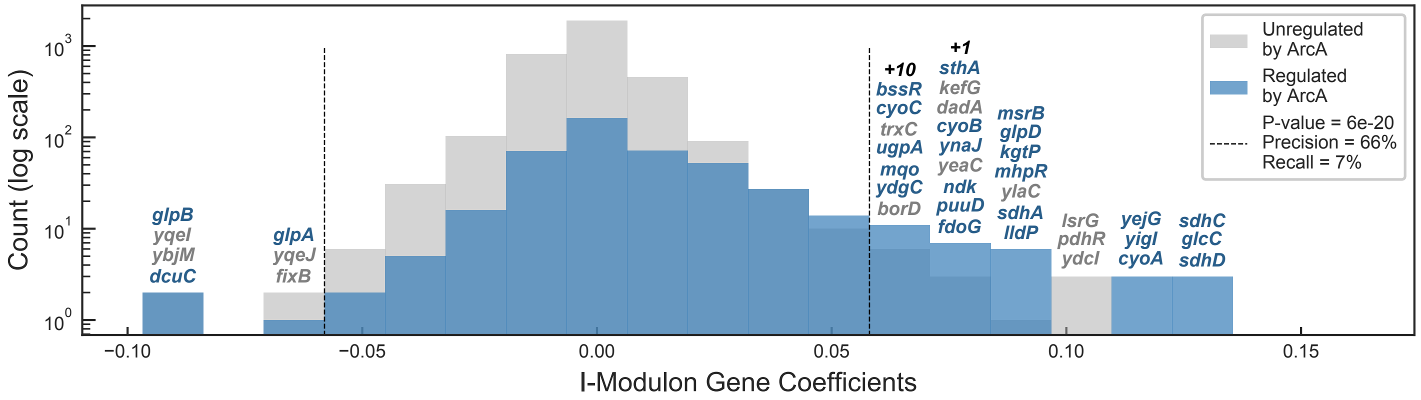

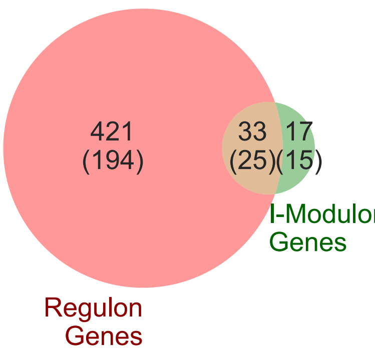

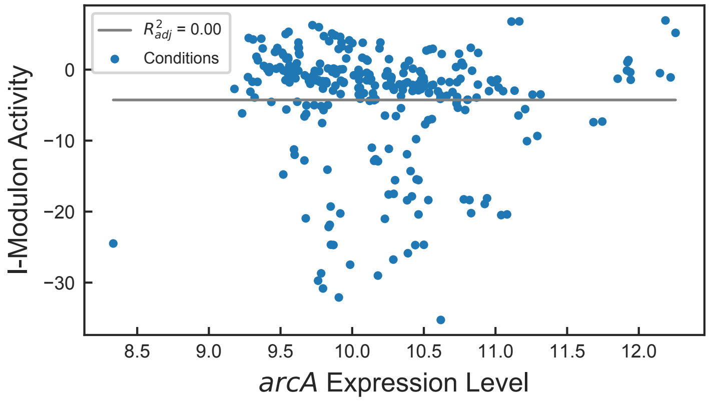

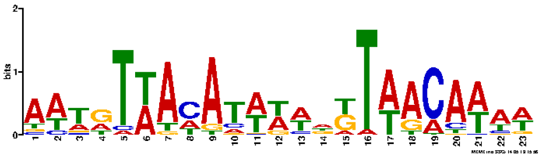

Motif E-value: 1.40e-12

Operons with Upstream Motif: 85%

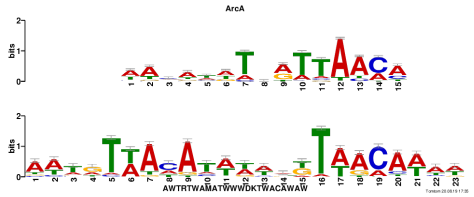

Motif similar to ArcA (E-value: 3.24e-04)

ArcA−2 I-Modulon

Regulated by: ArcA

Biological Function: Electron Transport Chain

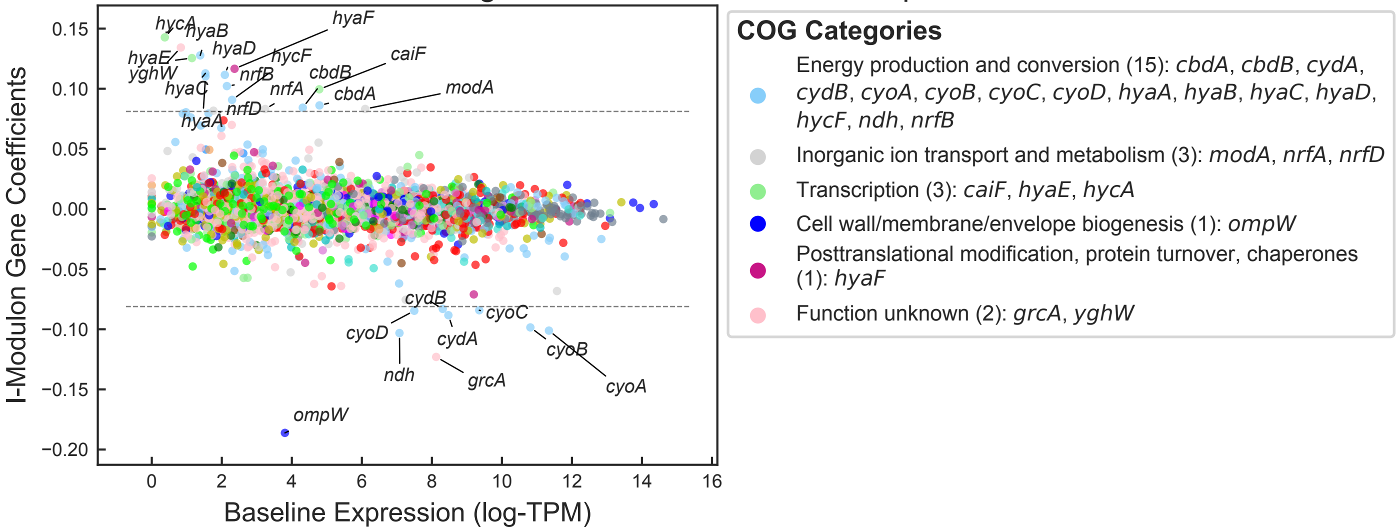

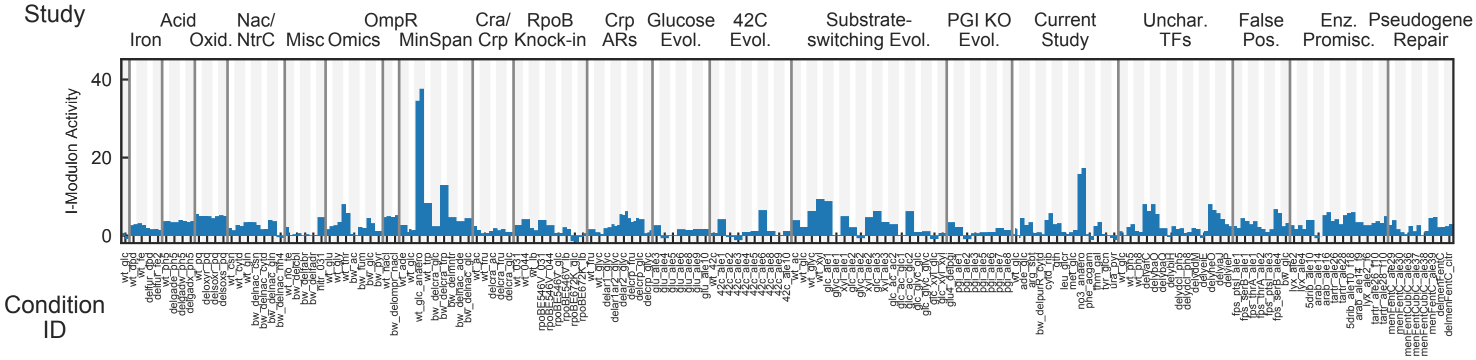

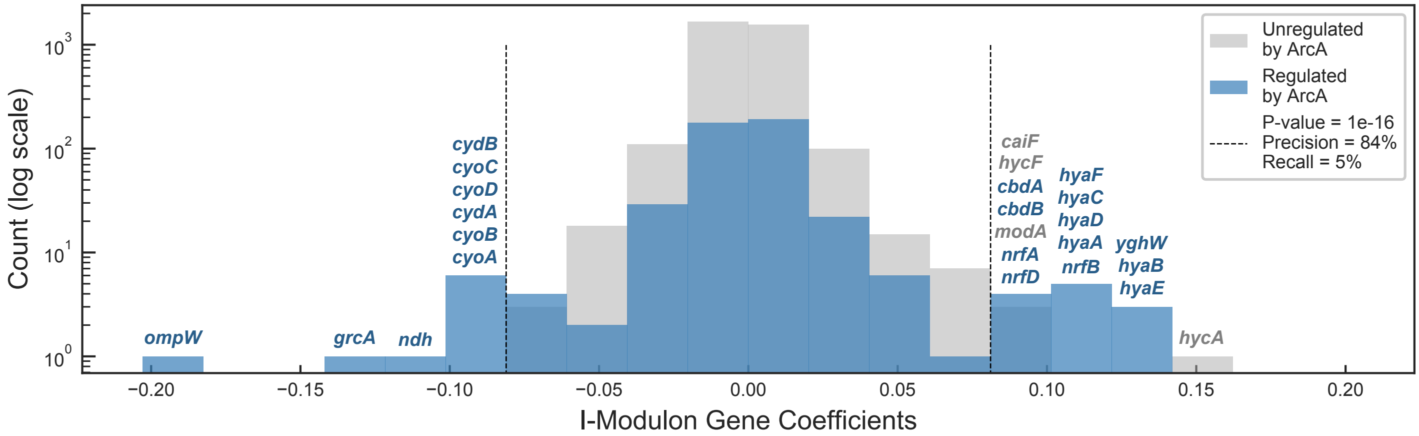

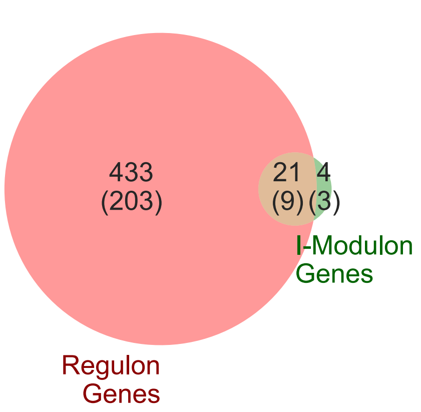

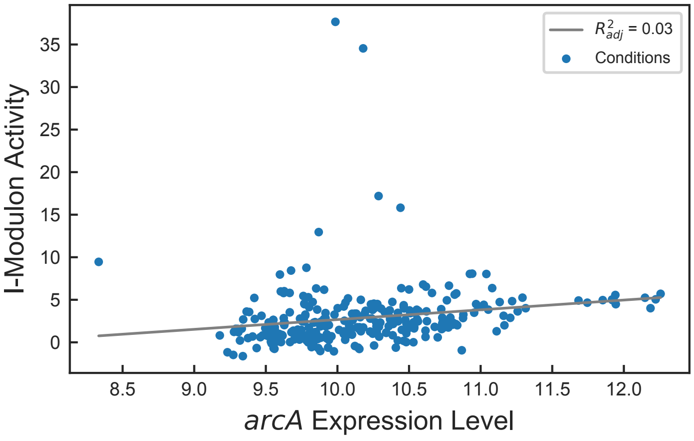

ArgR I-Modulon

Regulated by: ArgR

Biological Function: Arginine biosynthesis

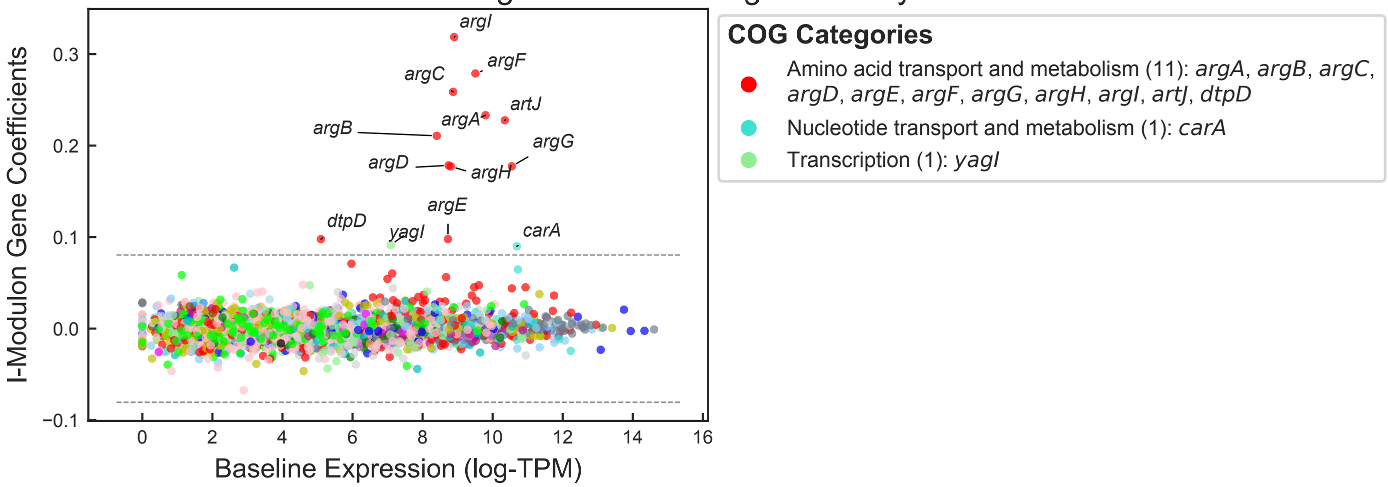

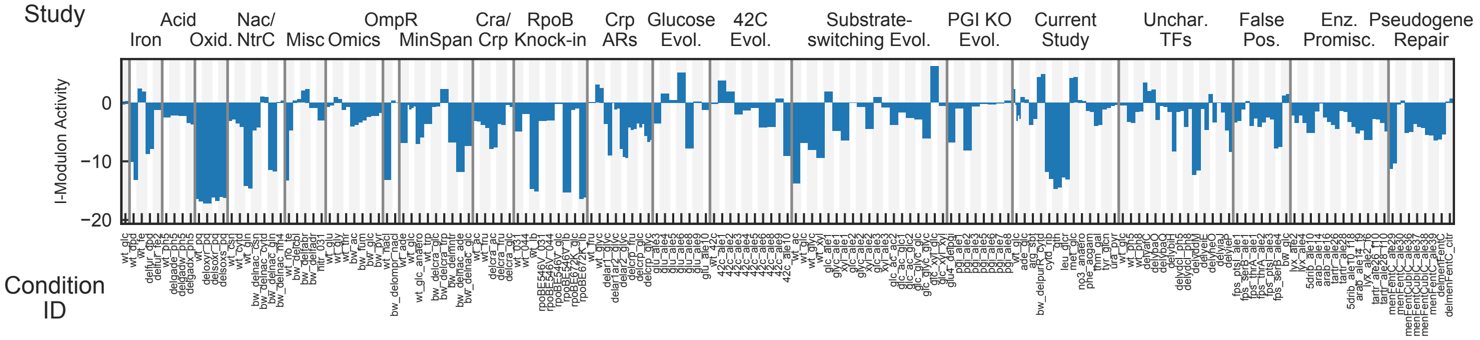

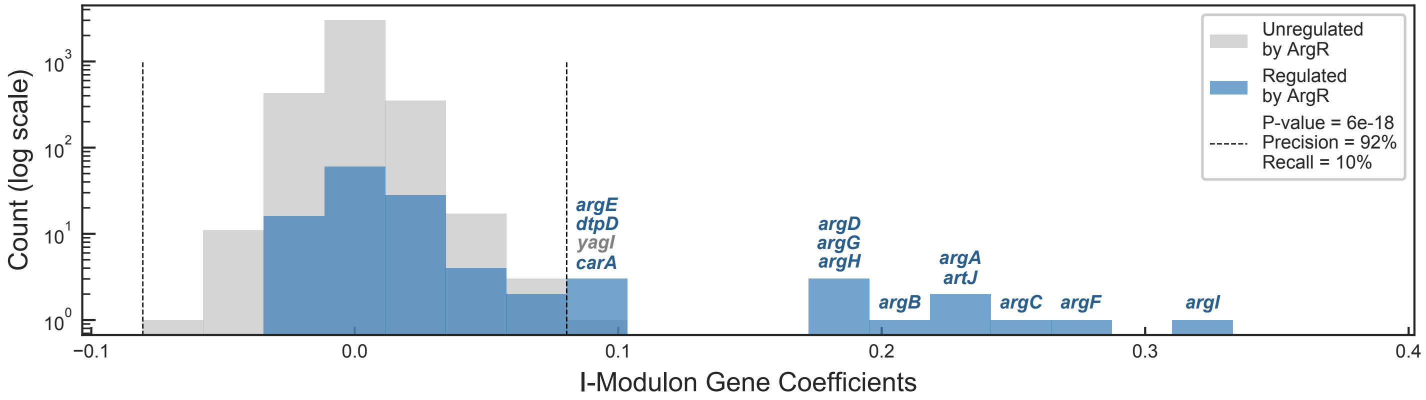

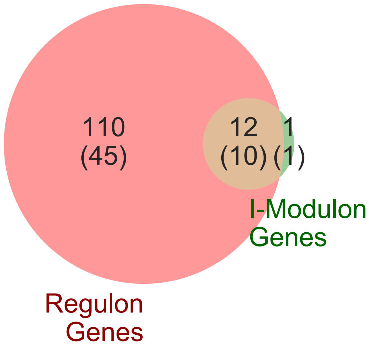

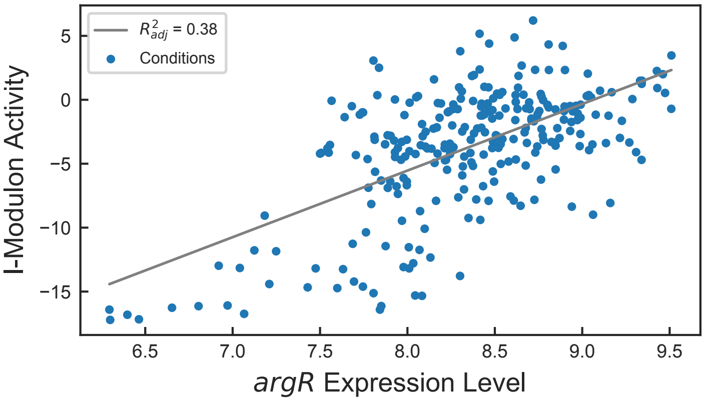

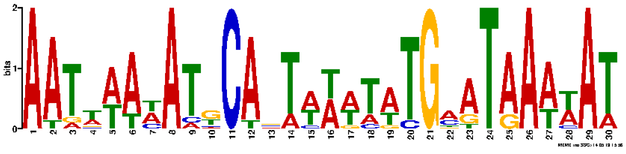

Motif E-value: 9.60e-21

Operons with Upstream Motif: 91%

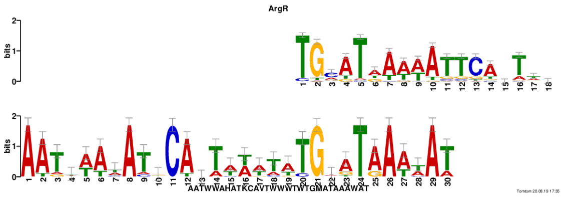

Motif similar to ArgR (E-value: 1.91e-04)

AtoC I-Modulon

Regulated by: AtoC

Biological Function: Acetoacetate degradation

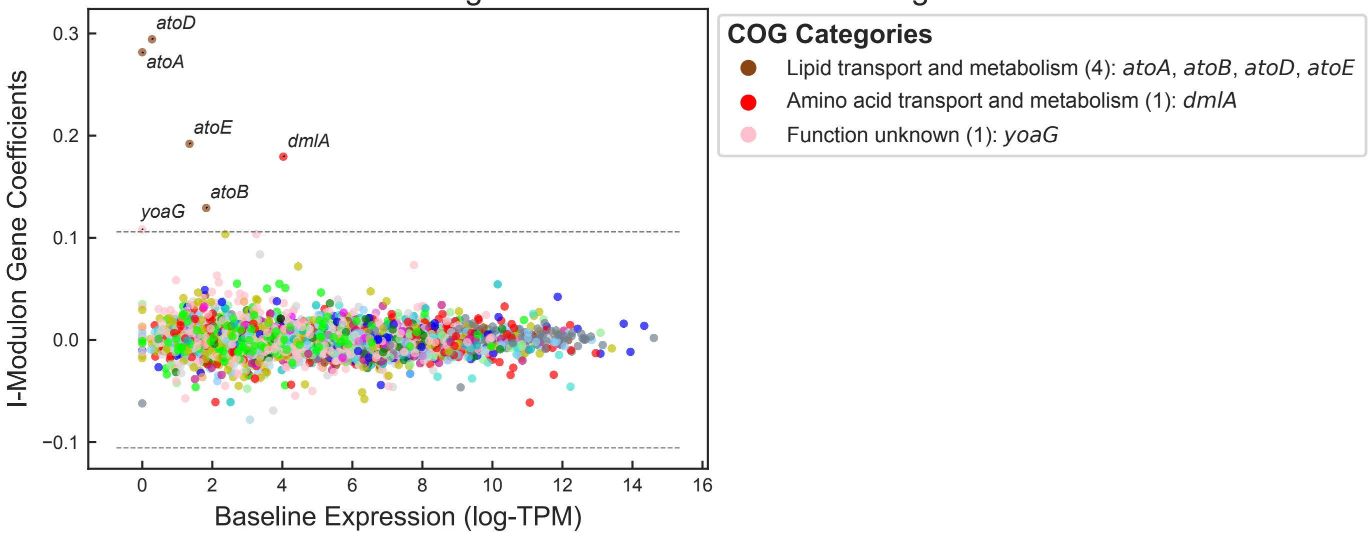

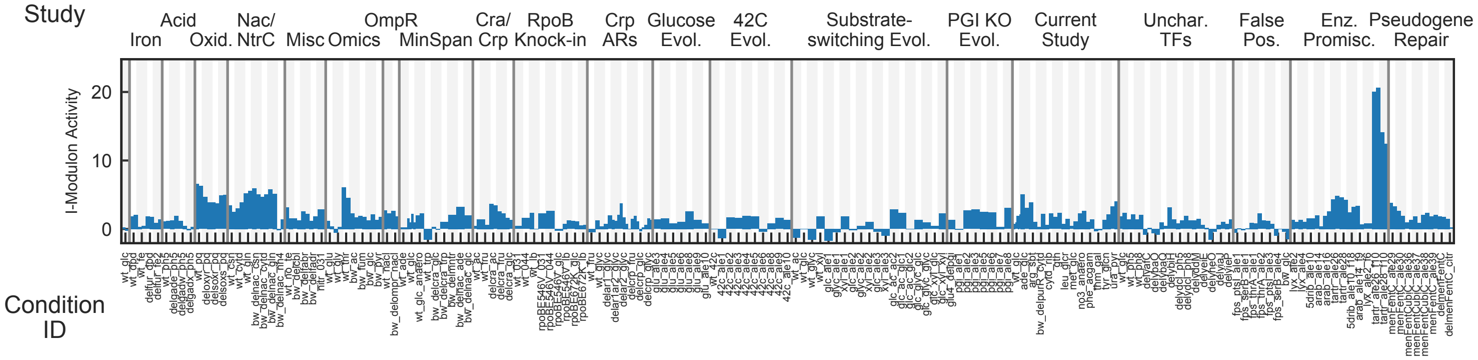

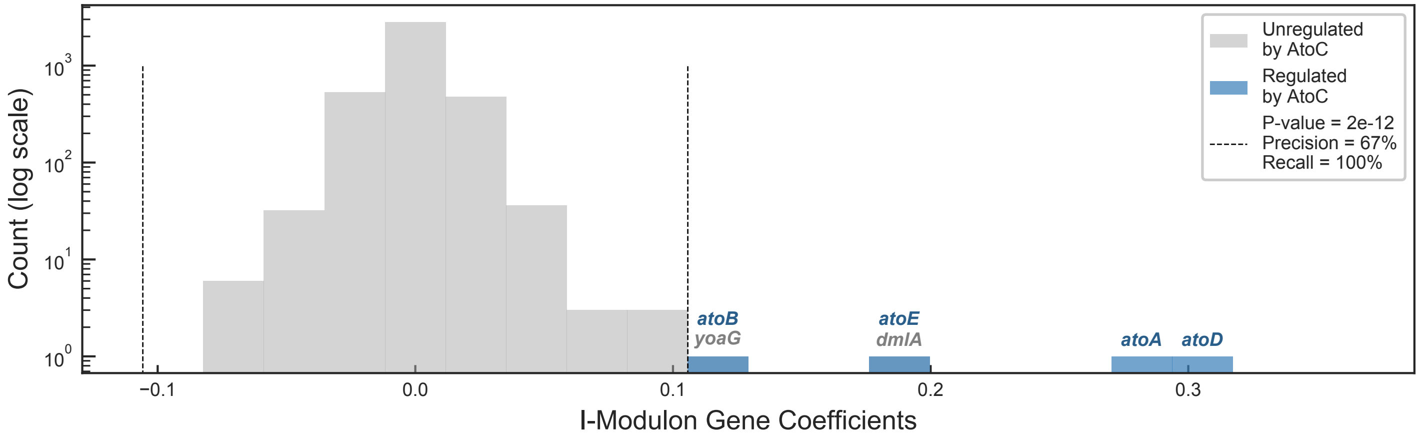

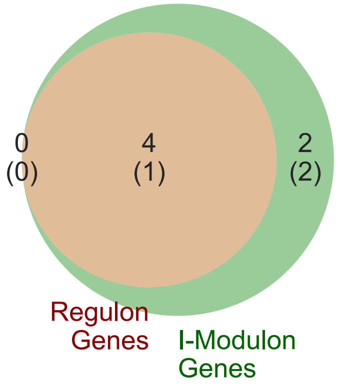

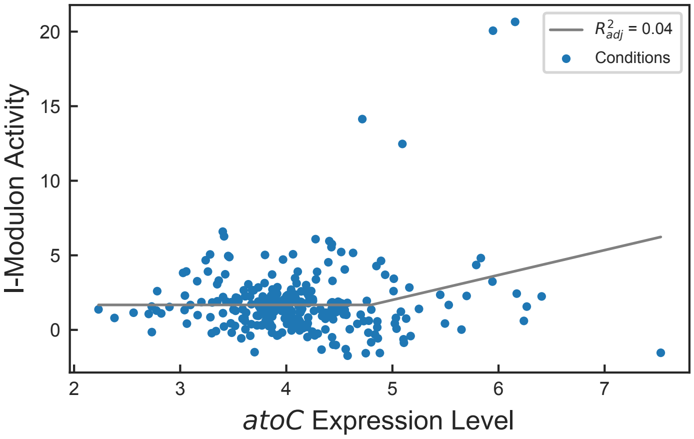

BW25113 I-Modulon

Biological Function: Transcriptional difference between BW25113 and MG1655

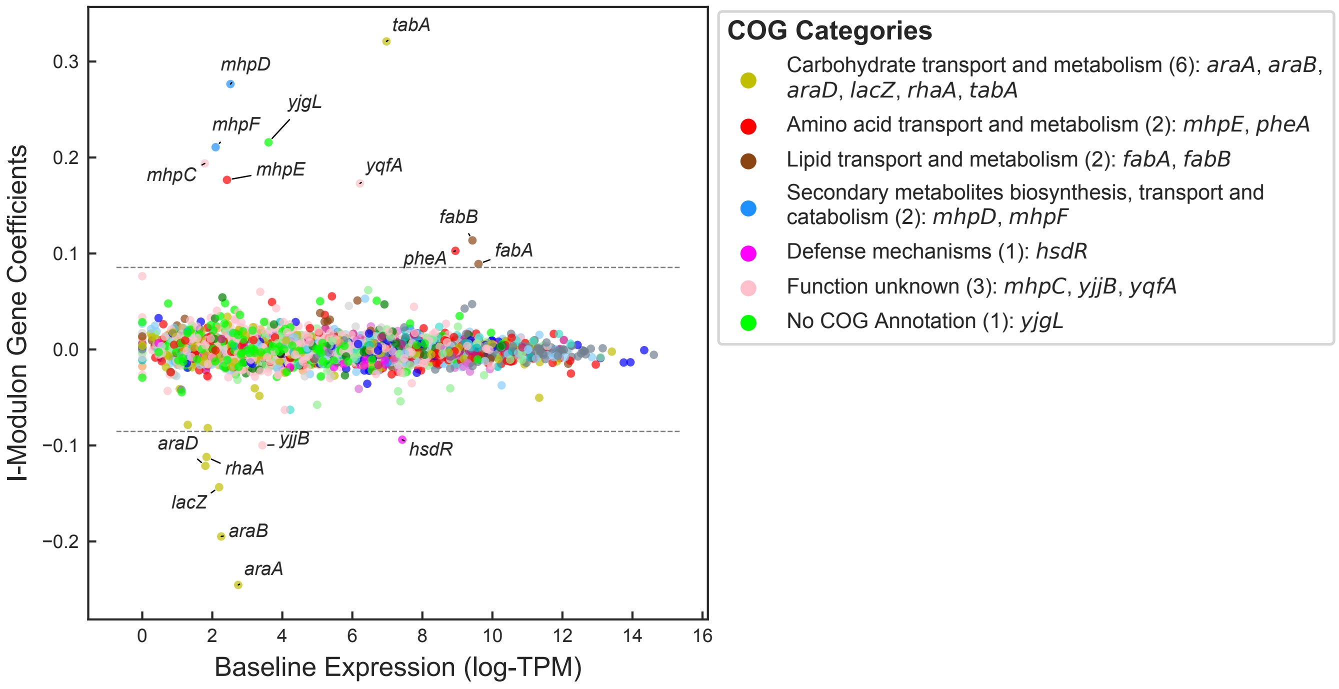

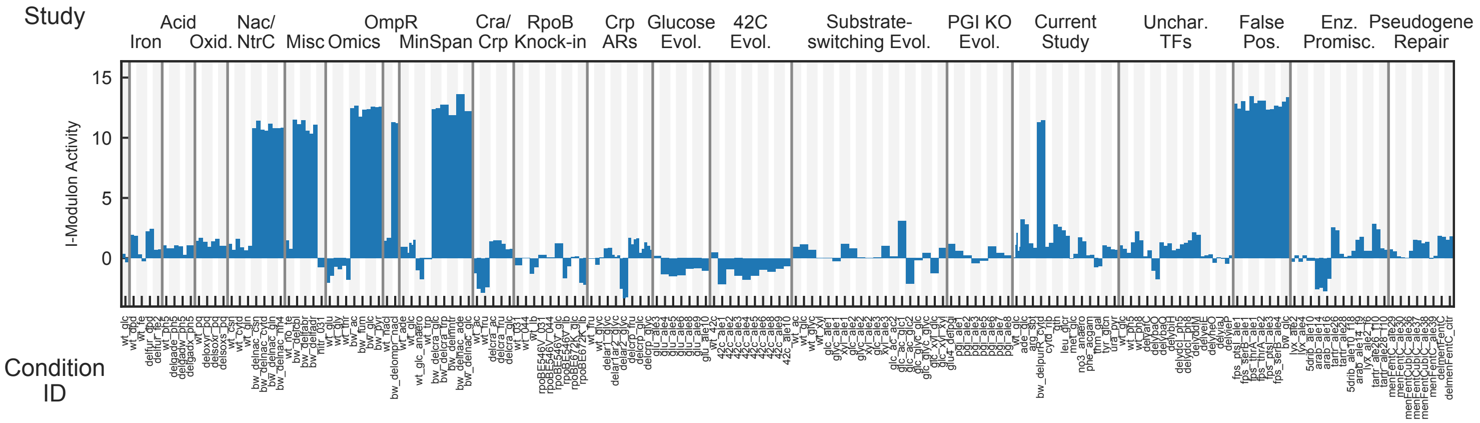

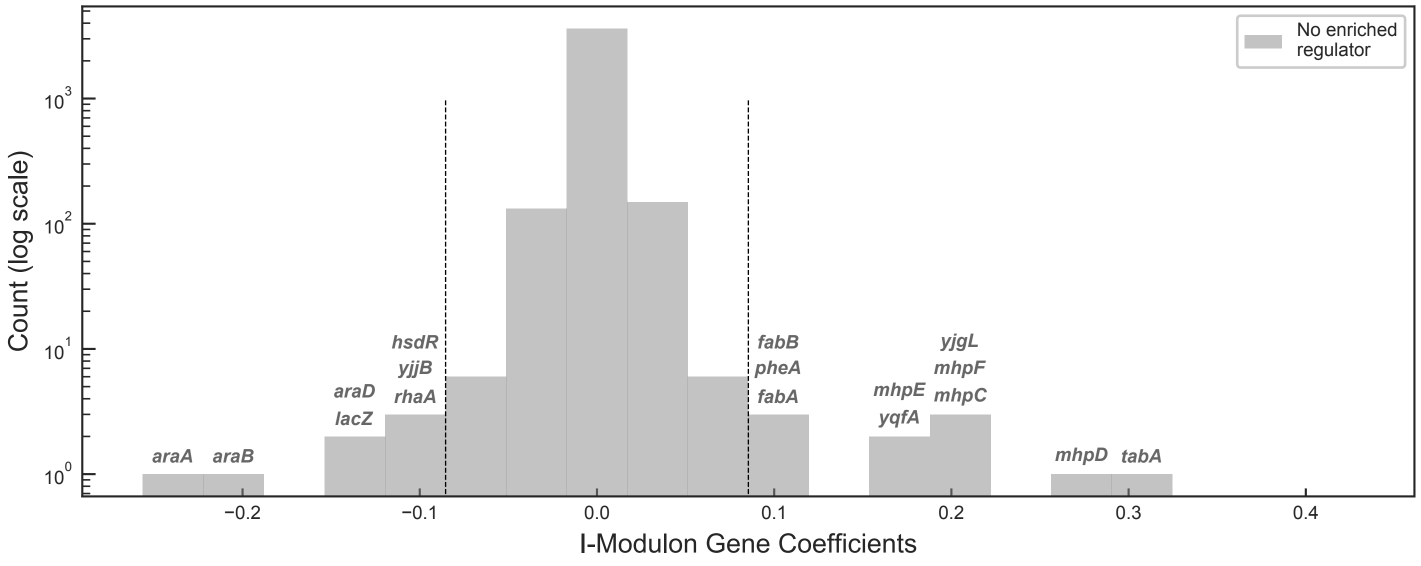

Cbl + CysB I-Modulon

Regulated by: Cbl and CysB

Biological Function: Aliphatic sulfonate utilization

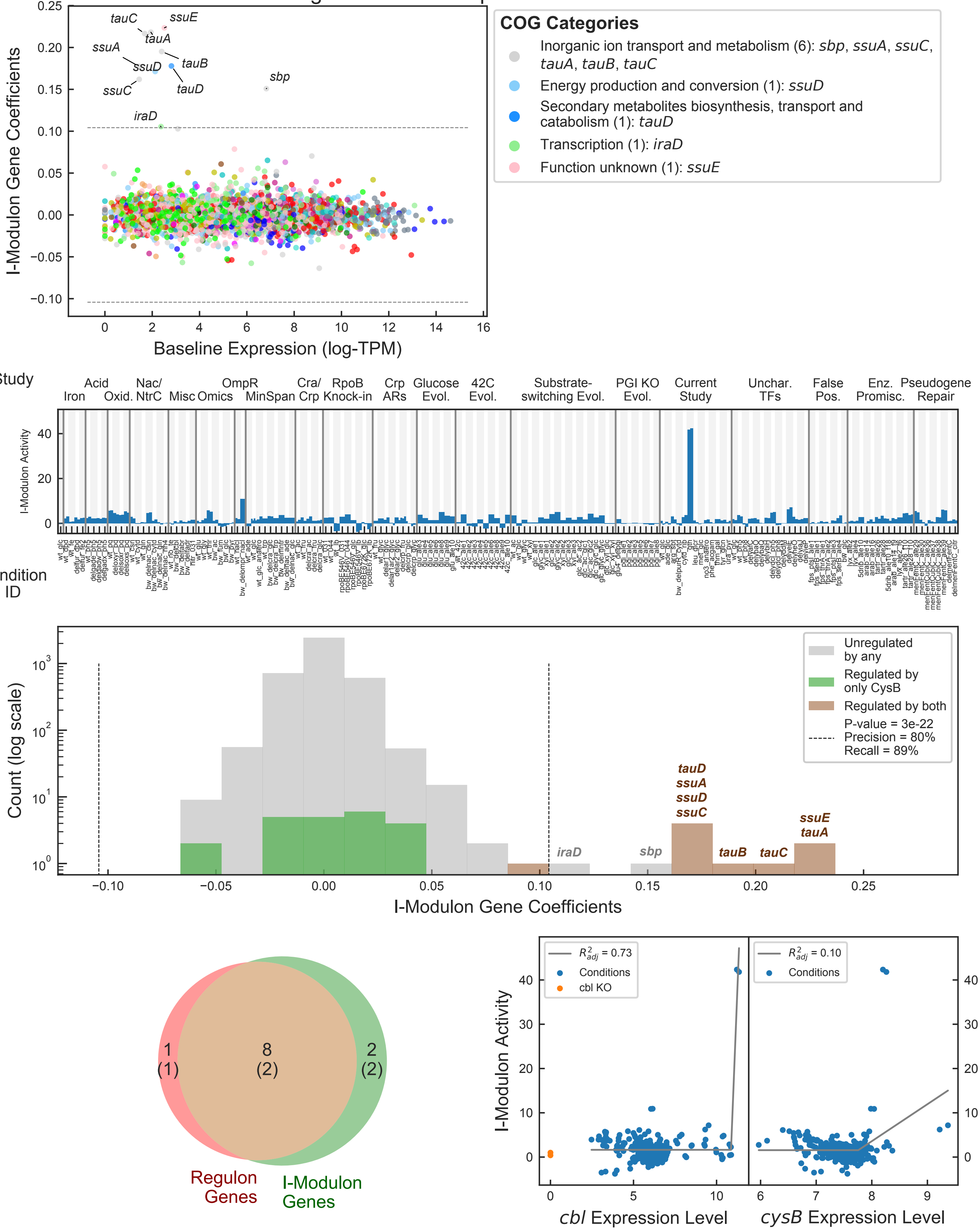

CdaR I-Modulon

Regulated by: CdaR

Biological Function: Glucarate catabolism

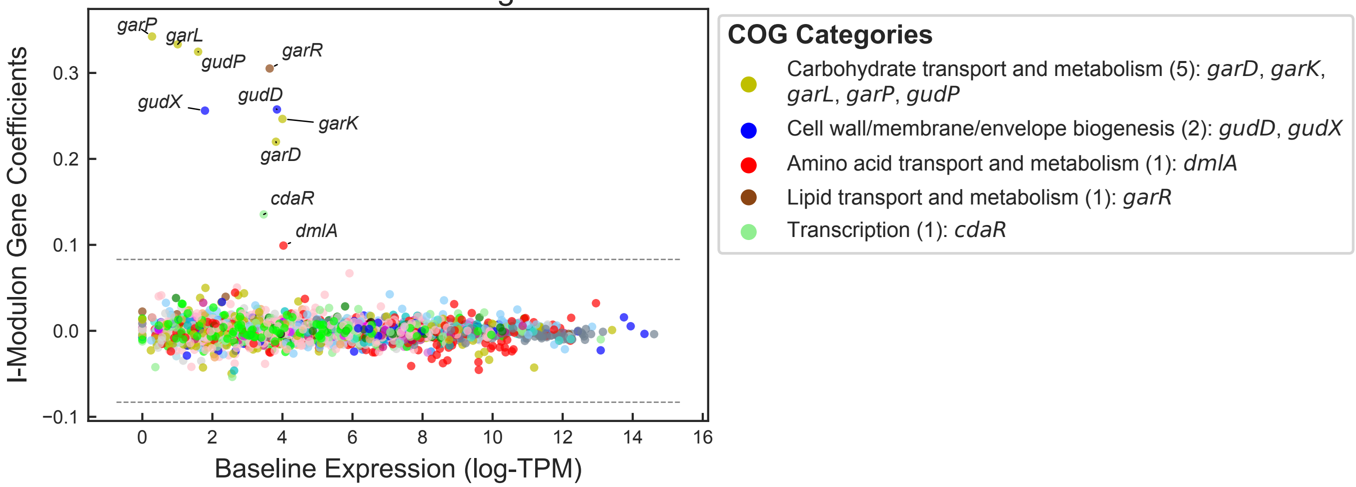

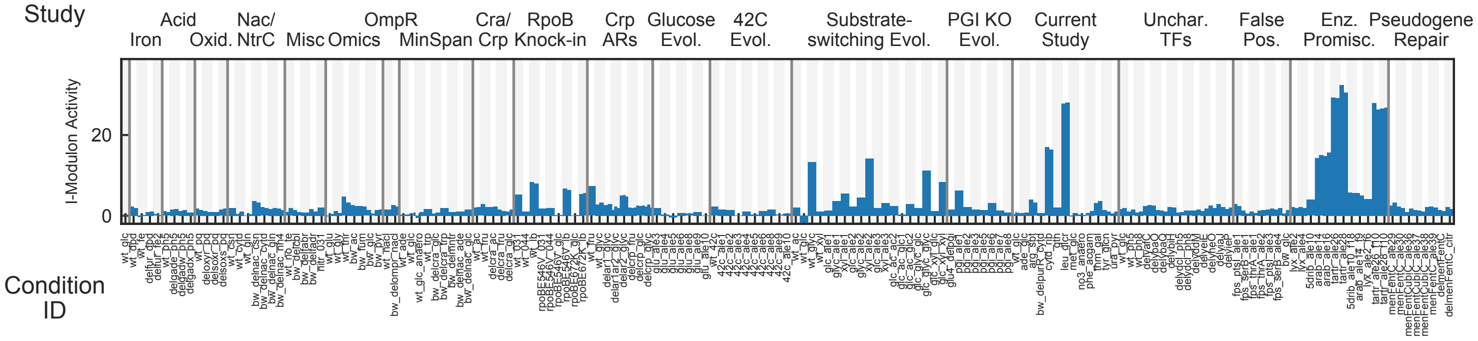

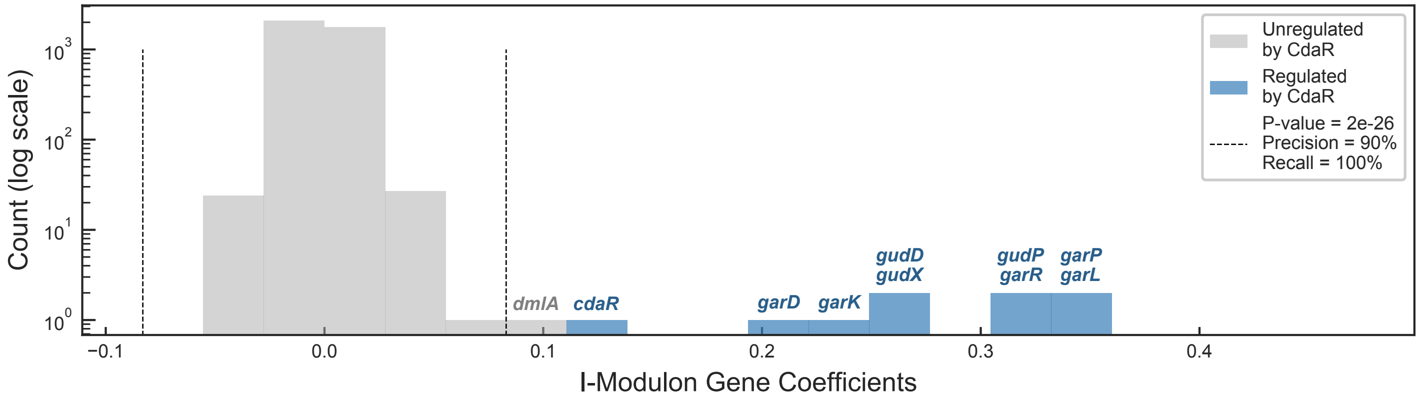

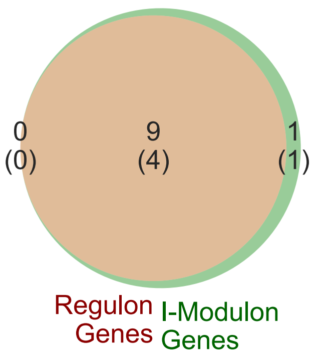

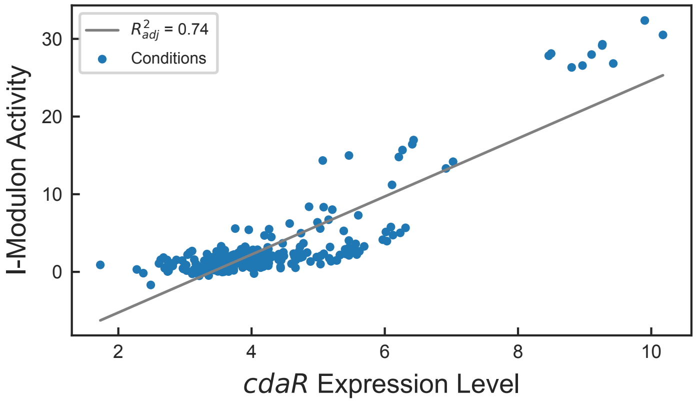

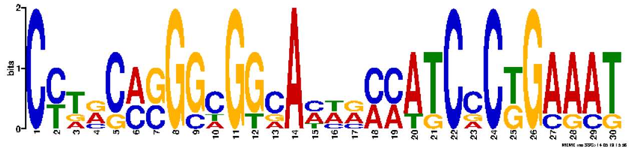

Motif E-value: 2.70e-05

Operons with Upstream Motif: 100%

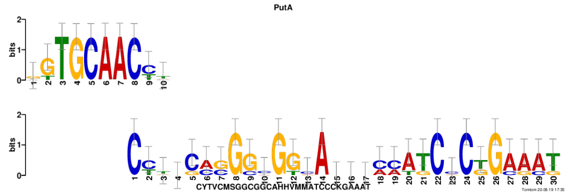

Motif similar to PutA (E-value: 1.53e-04)

CecR I-Modulon

Regulated by: CecR

Biological Function: Related to antibiotic sensitivity

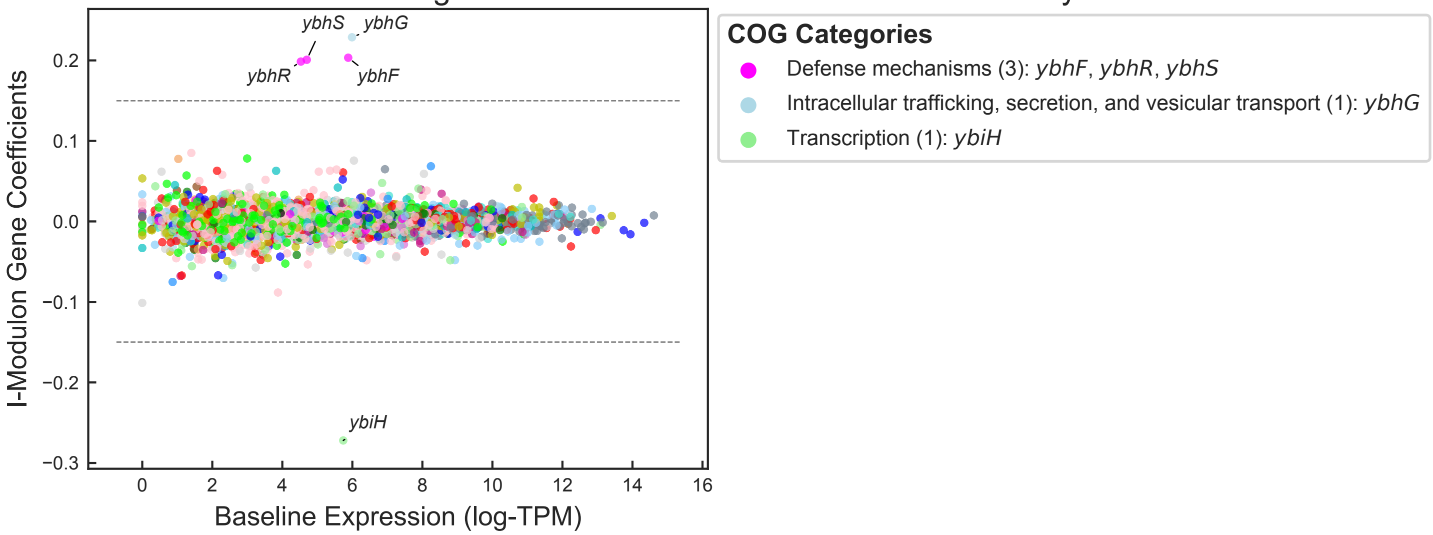

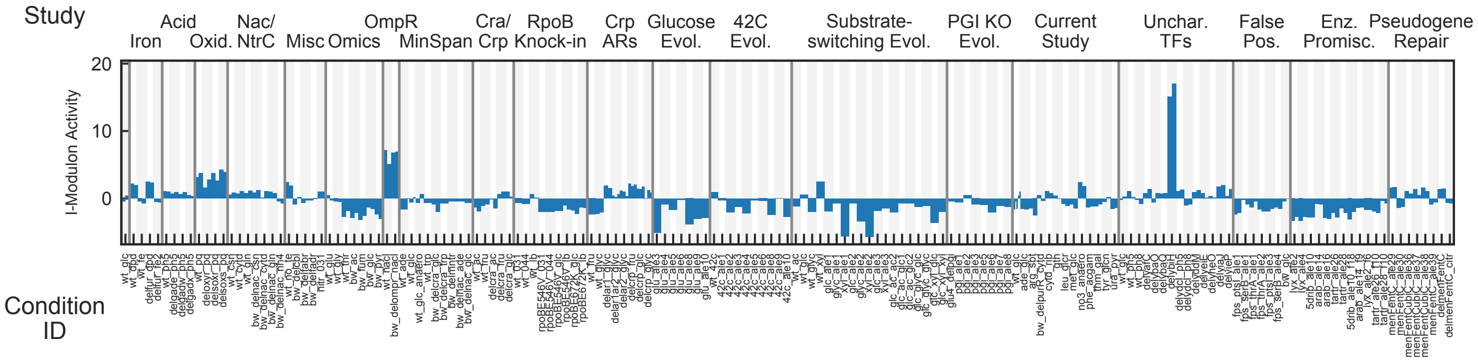

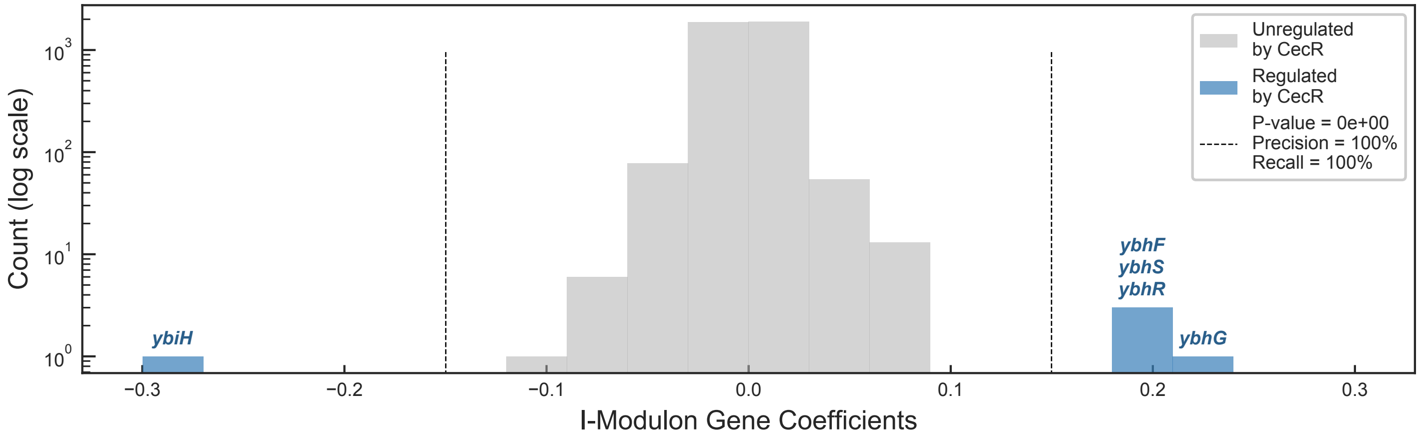

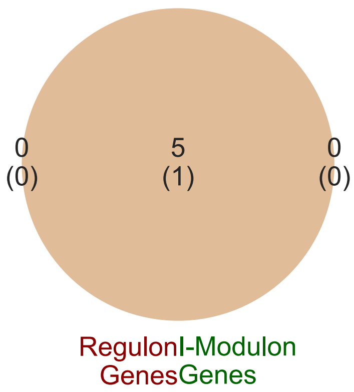

Copper I-Modulon

Regulated by: CusR or HprR or CueR

Biological Function: Copper homeostasis

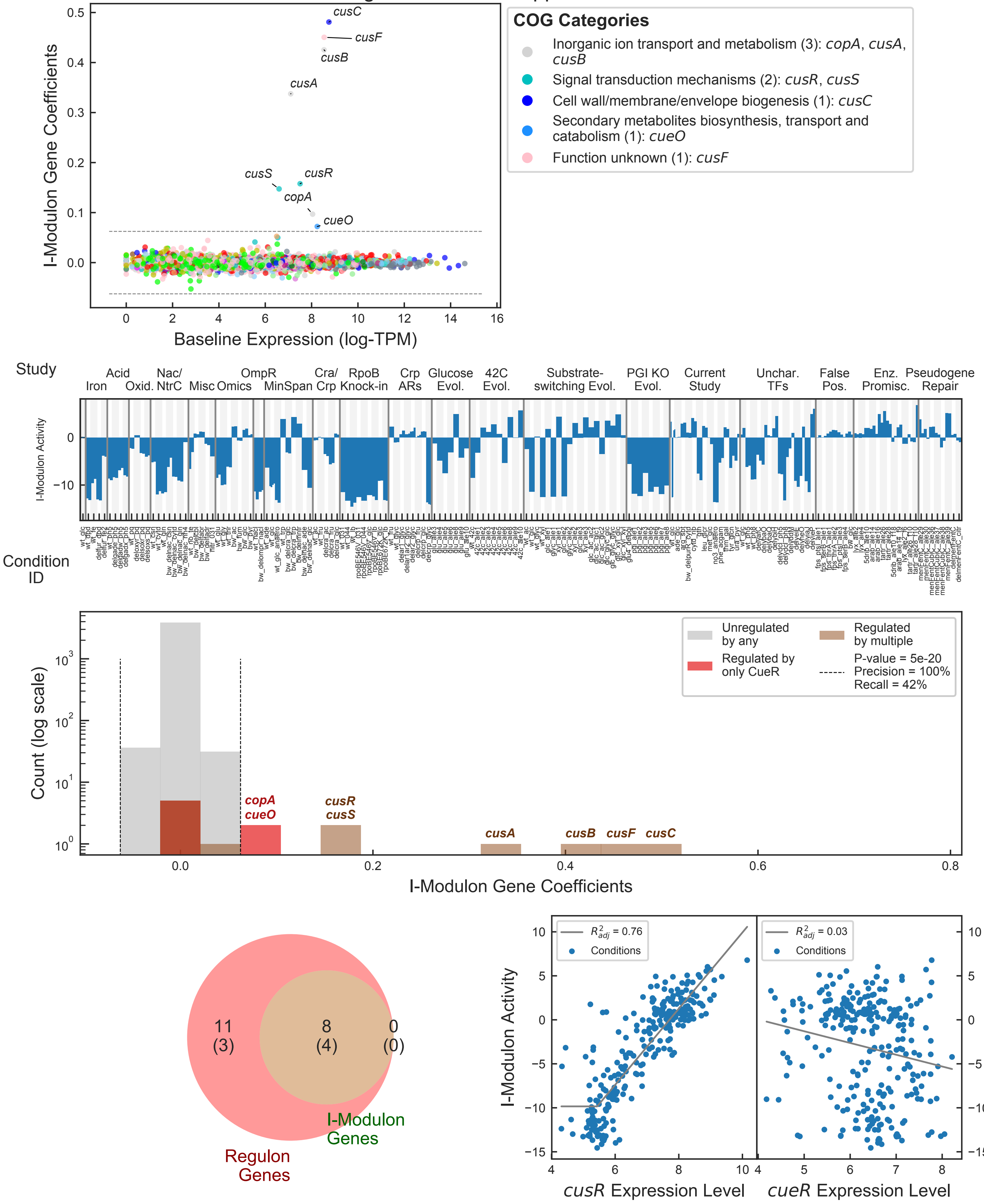

CpxR I-Modulon

Regulated by: CpxR

Biological Function: Various stress responses

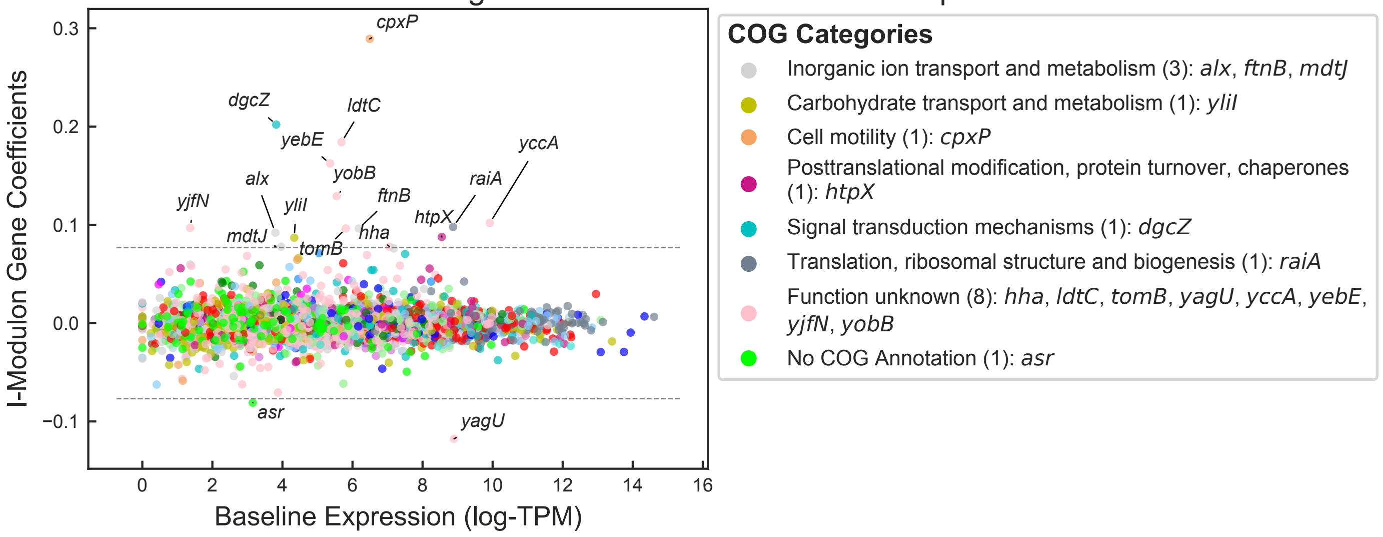

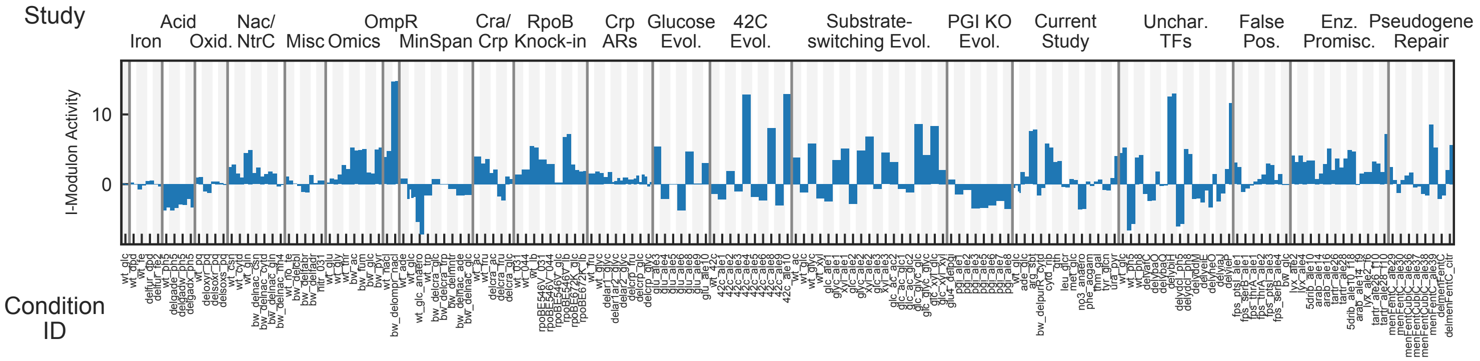

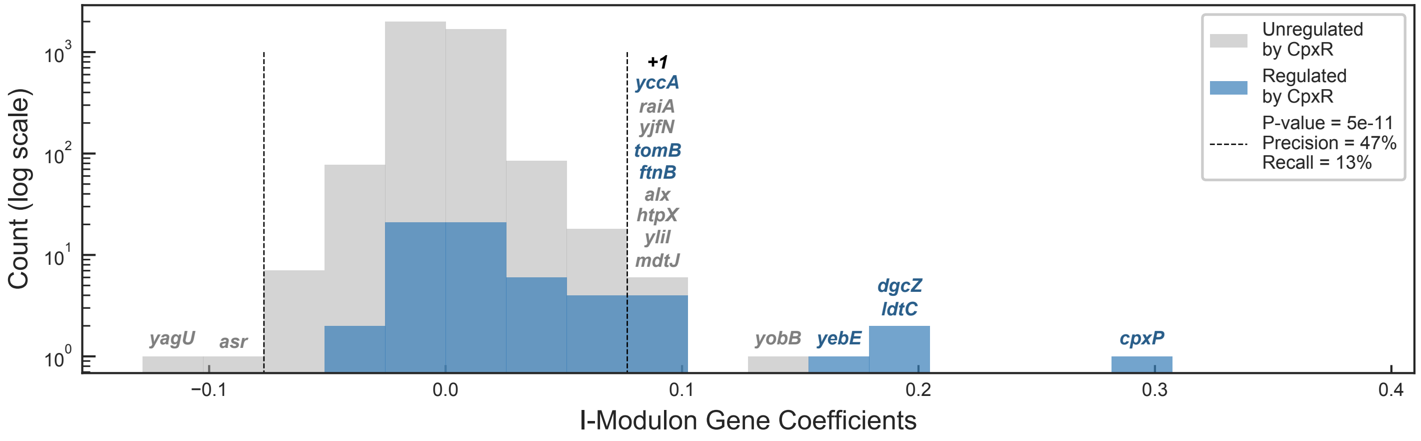

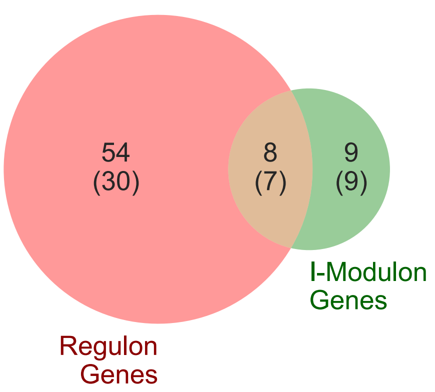

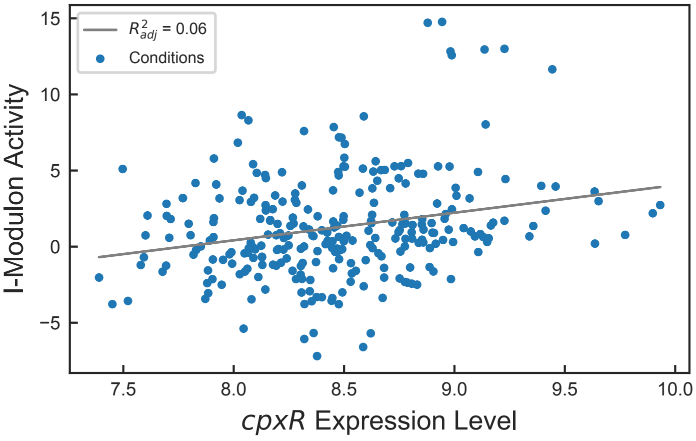

Cra I-Modulon

Regulated by: Cra

Biological Function: Central carbon metabolism

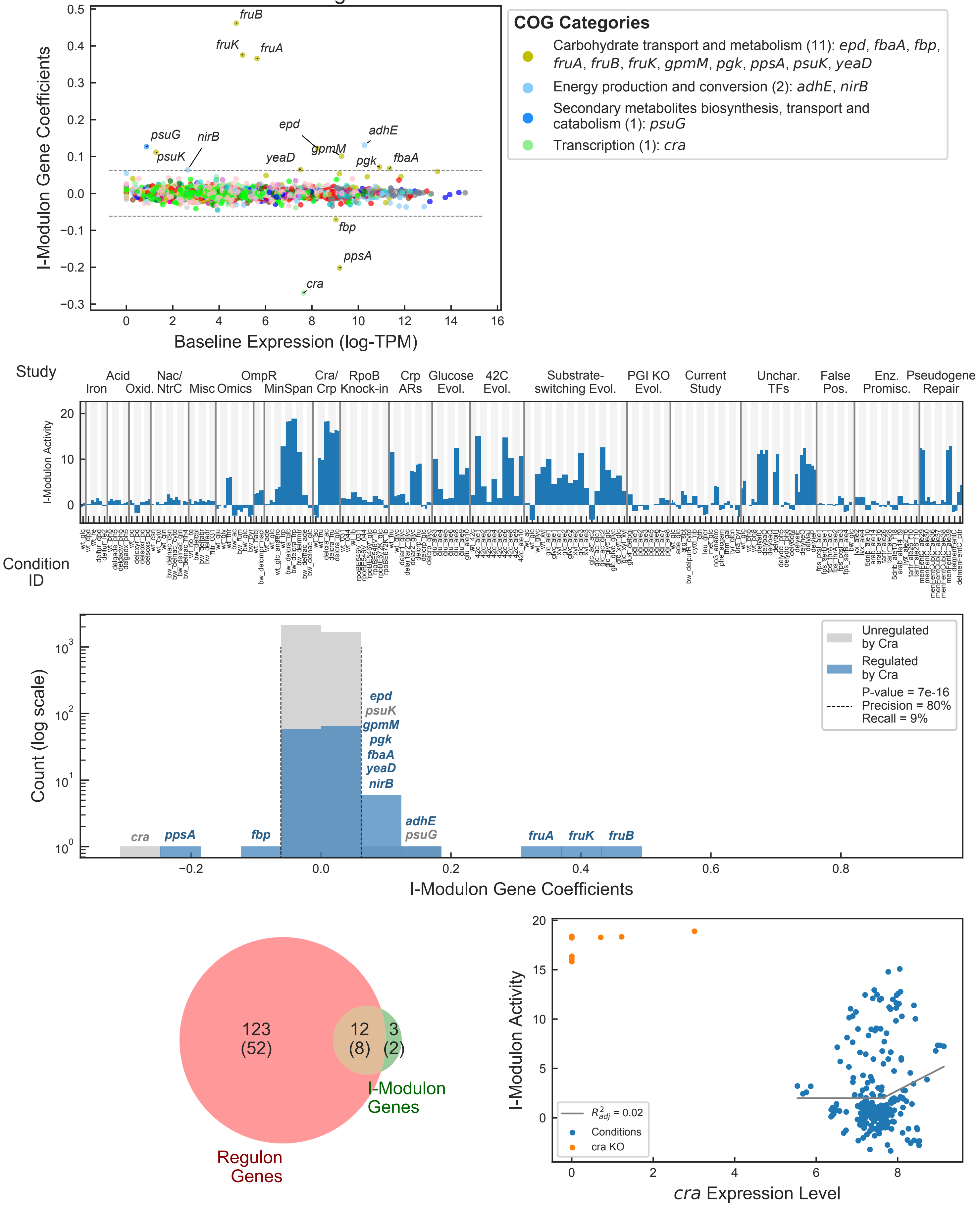

Crp − 1 I-Modulon

Regulated by: Crp

Biological Function: Miscellaneous functions

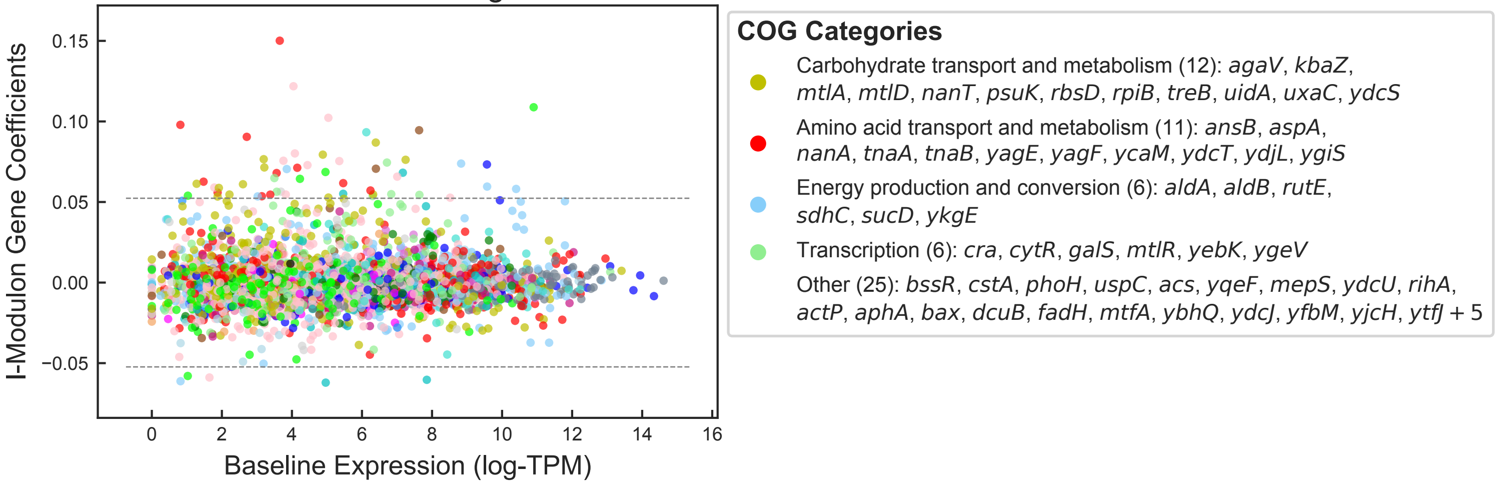

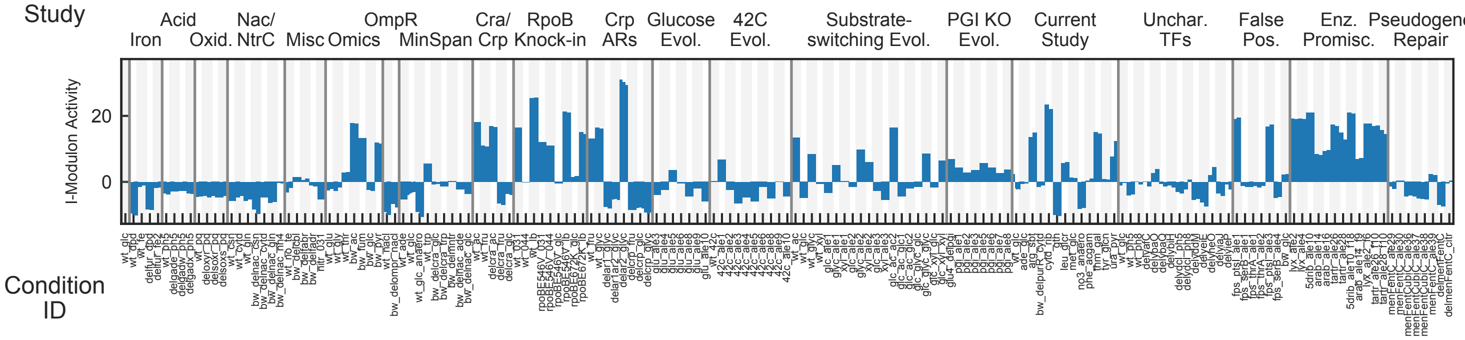

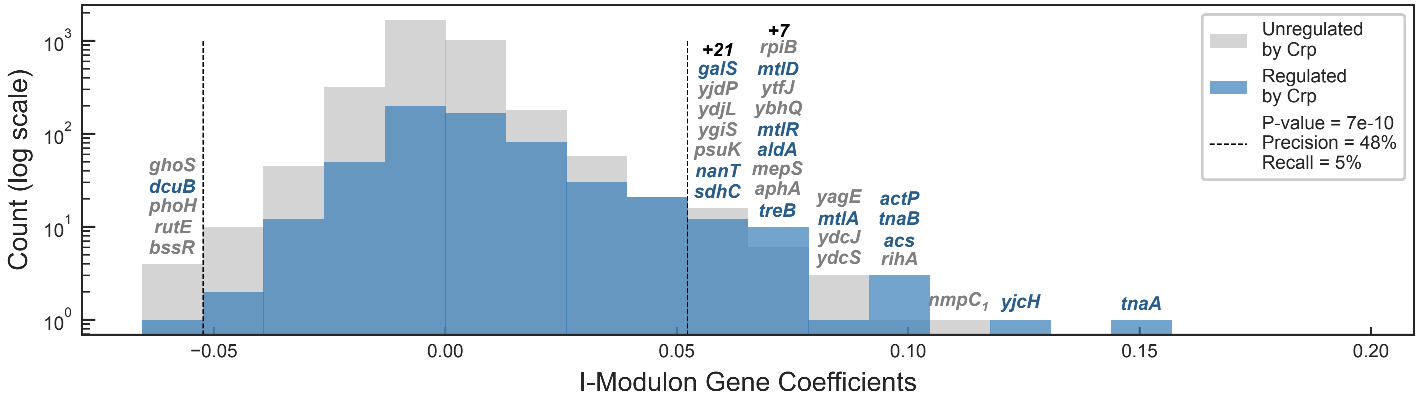

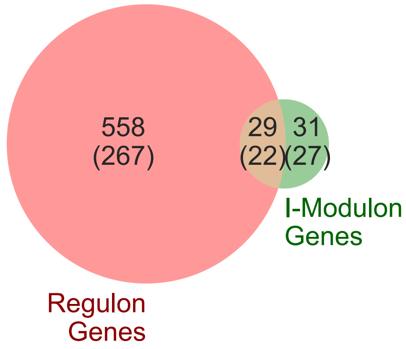

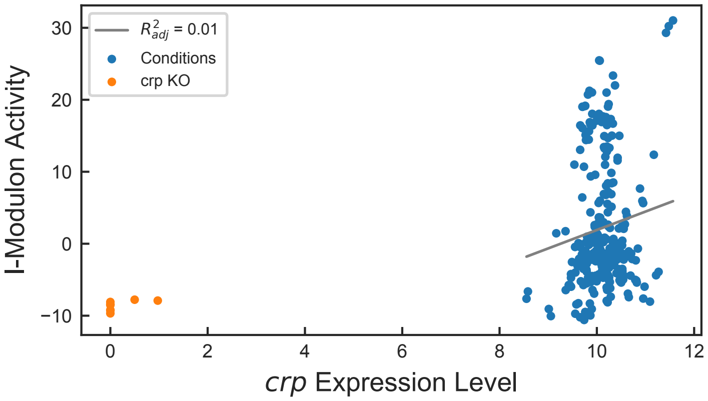

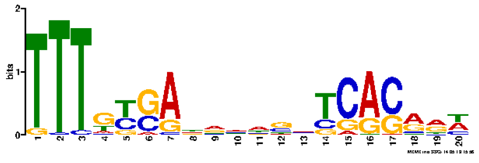

Motif E-value: 5.30e-07

Operons with Upstream Motif: 83%

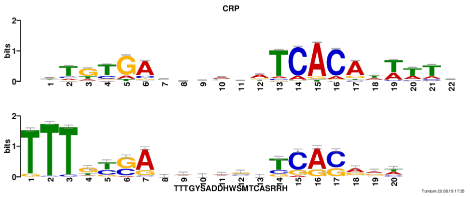

Motif similar to CRP (E-value: 7.82e-09)

Crp−2 I-Modulon

Regulated by: Crp

Biological Function: Various carbon source catabolism

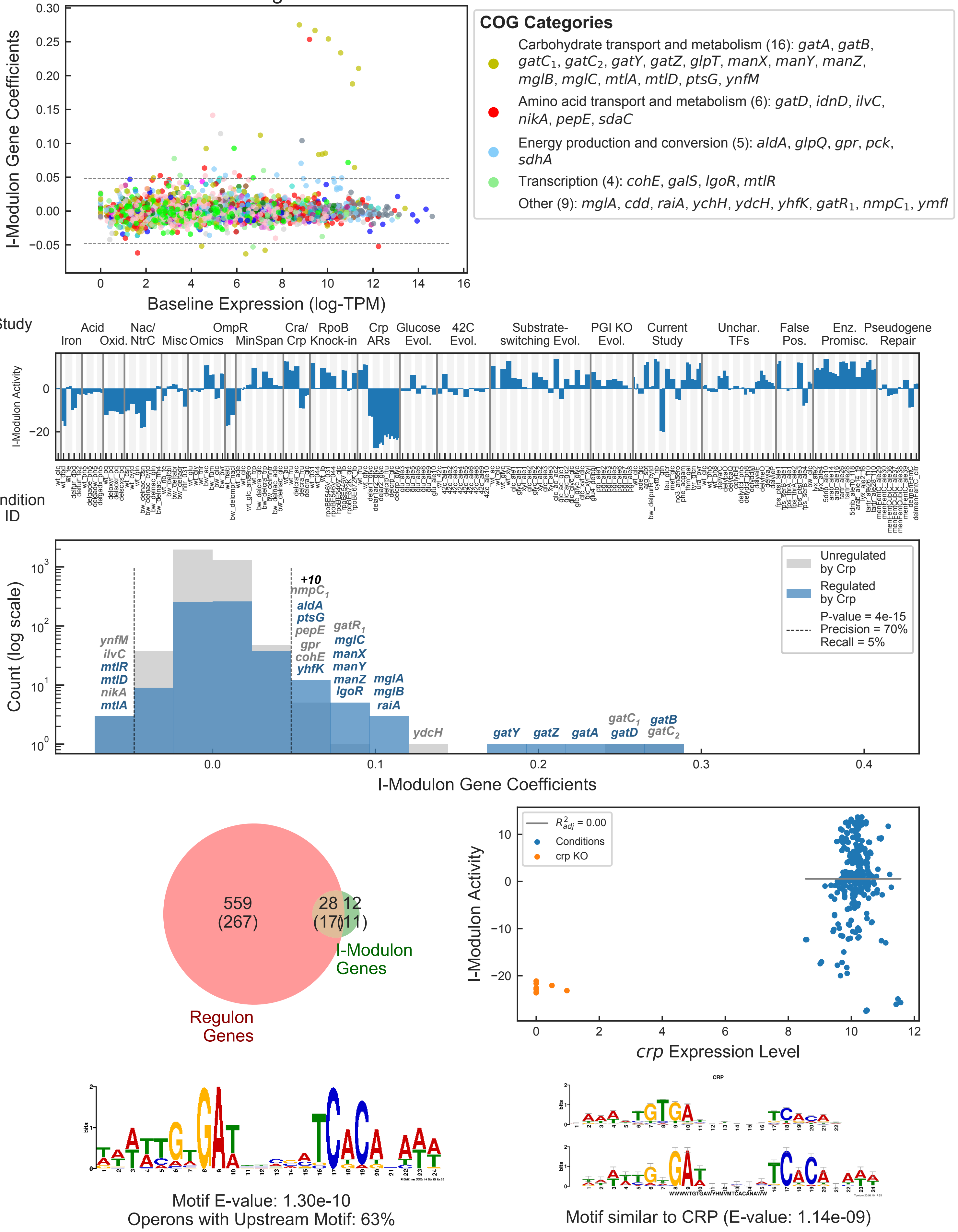

CsqR I-Modulon

Regulated by: CsqR

Biological Function: Sulfoquinovose catabolism

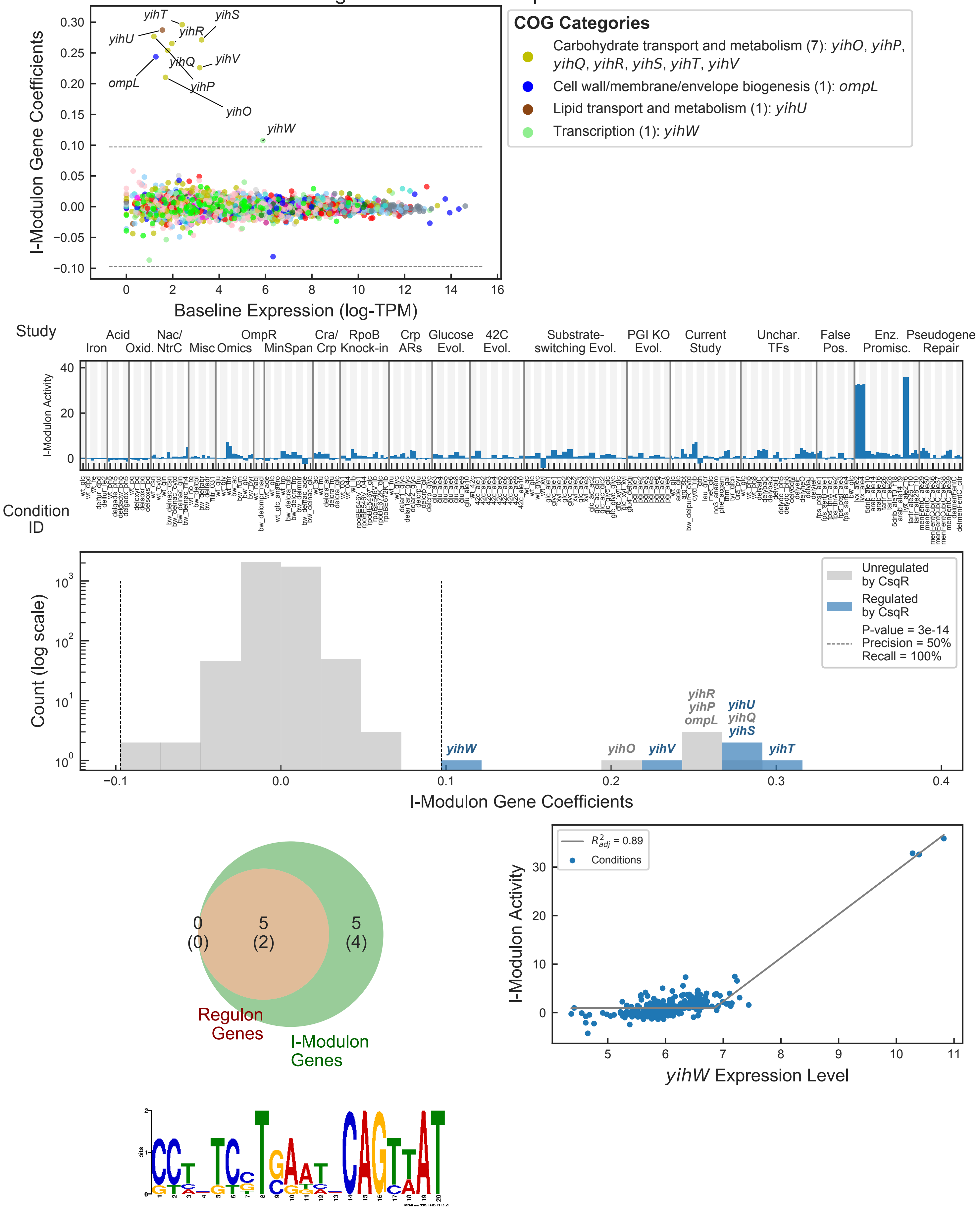

Motif E-value: 4.50e-04

Operons with Upstream Motif: 100%

CysB I-Modulon

Regulated by: CysB

Biological Function: Inorganic sulfate assimilation

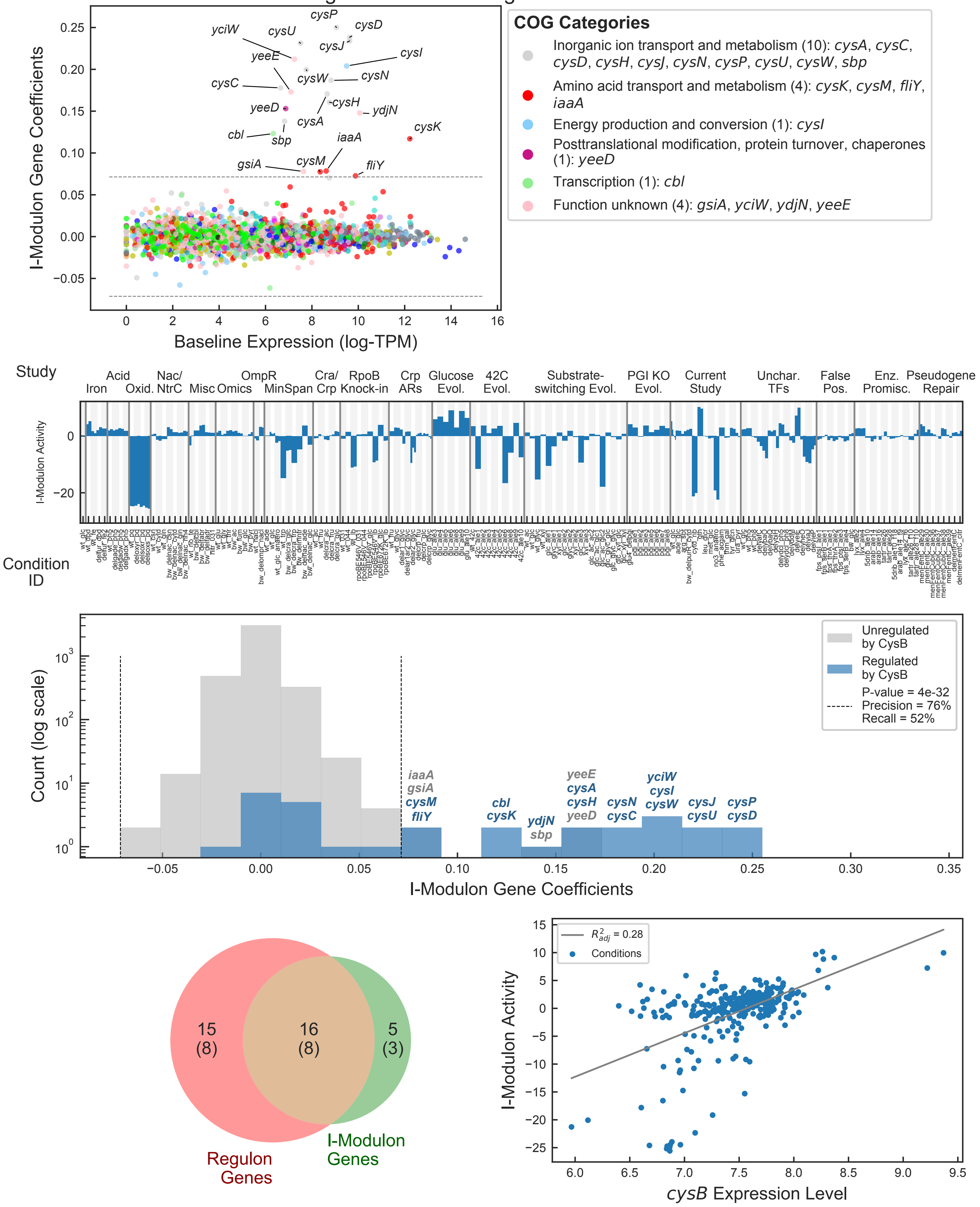

DhaR/Mlc I-Modulon

Regulated by: DhaR or Mlc

Biological Function: Dihydroxyacetone kinase and phosphotransferase systems

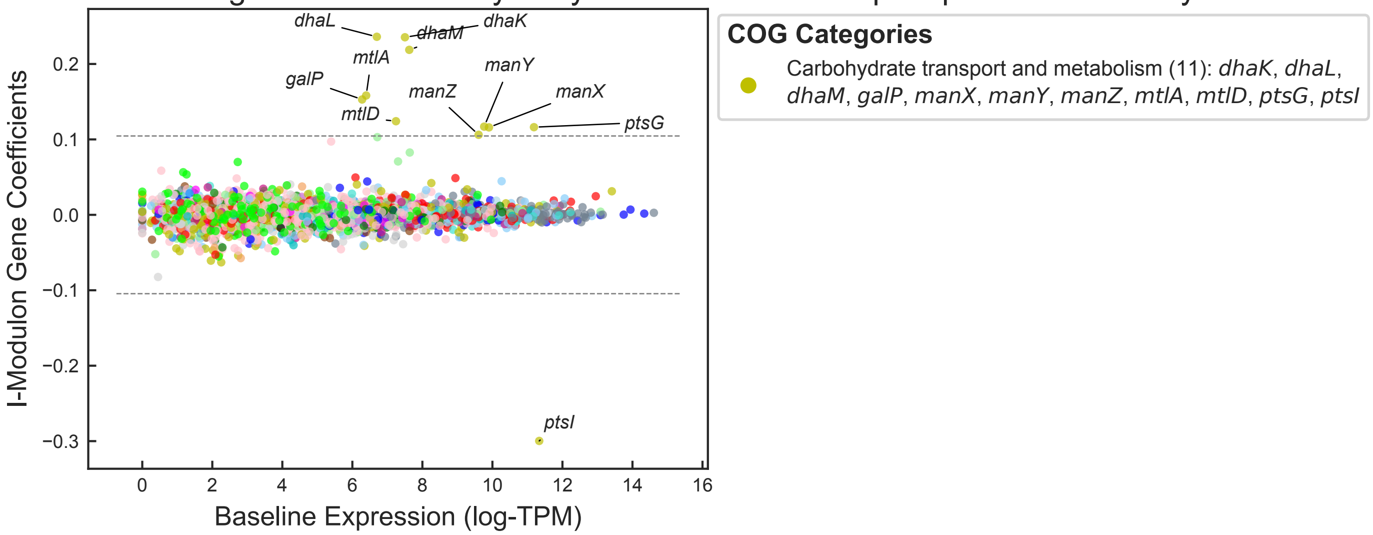

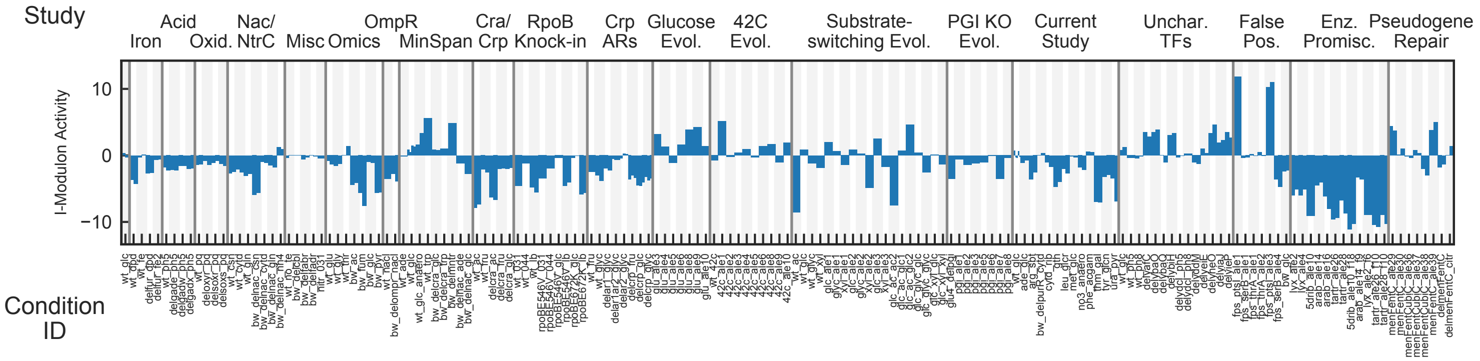

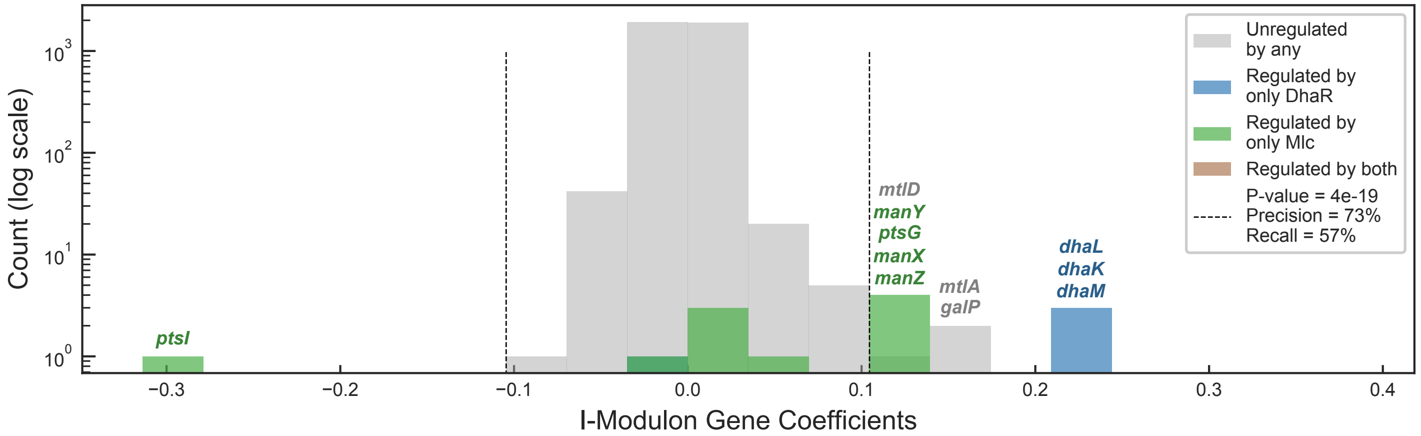

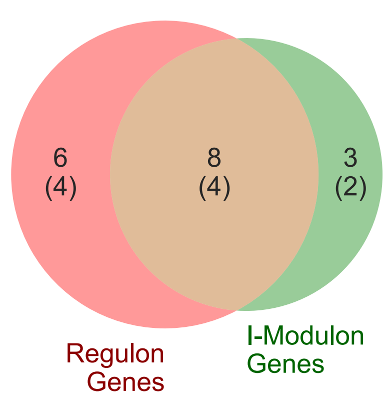

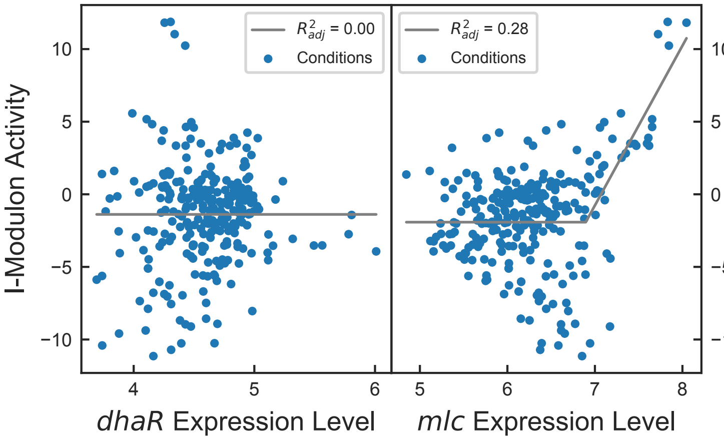

EvgA I-Modulon

Regulated by: EvgA

Biological Function: Acid and osmotic stress response

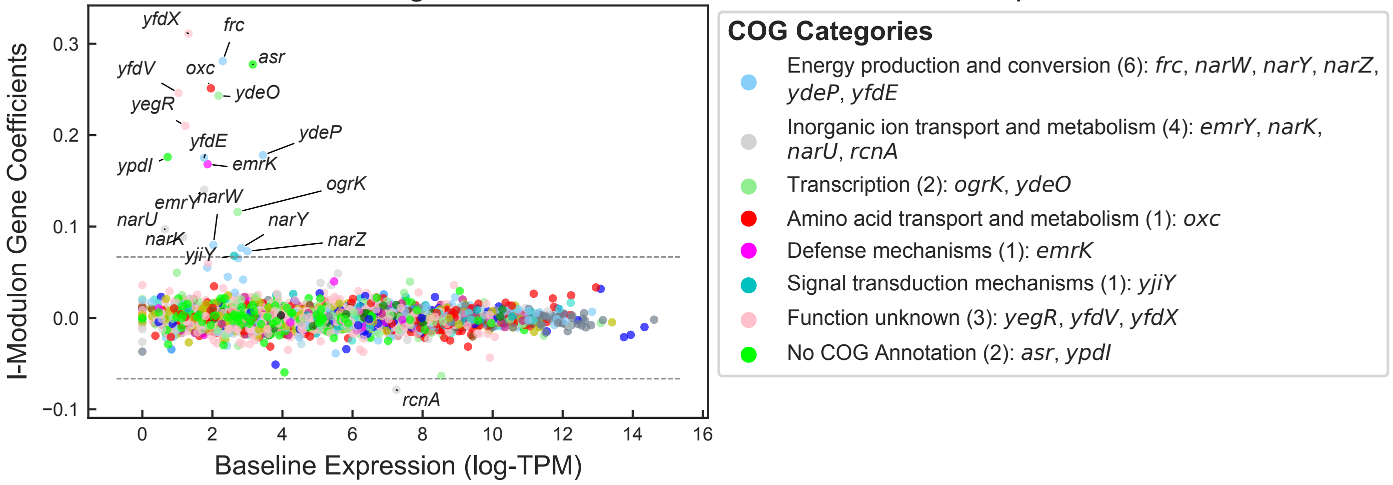

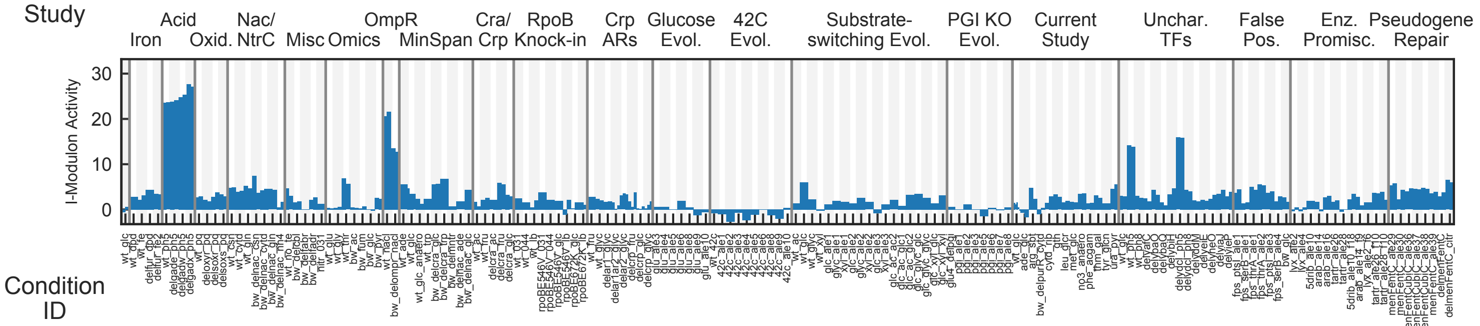

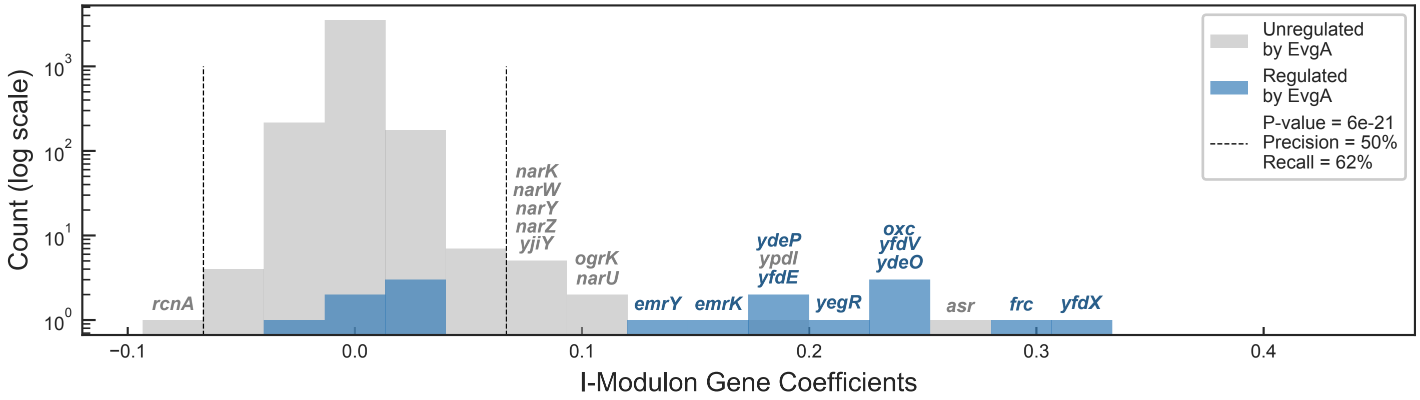

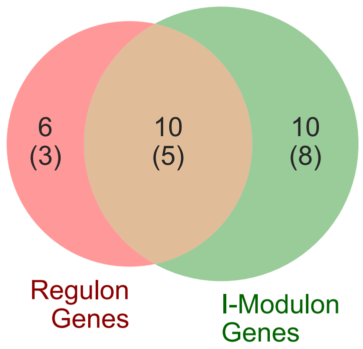

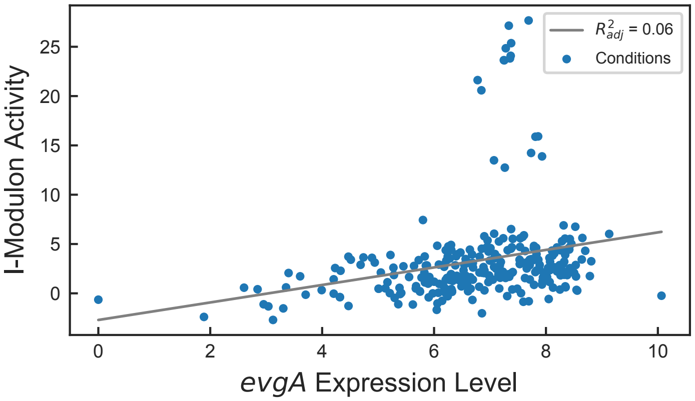

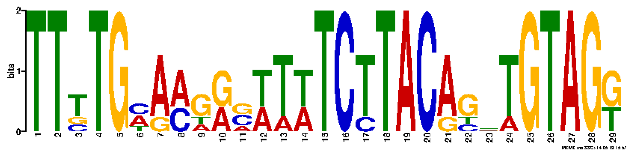

Motif E-value: 2.20e-05

Operons with Upstream Motif: 38%

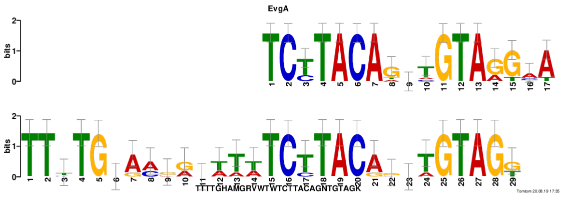

Motif similar to EvgA (E-value: 6.32e-12)

ExuR/FucR I-Modulon

Regulated by: ExuR or FucR

Biological Function: D-galacturonate and L-fucose catabolism

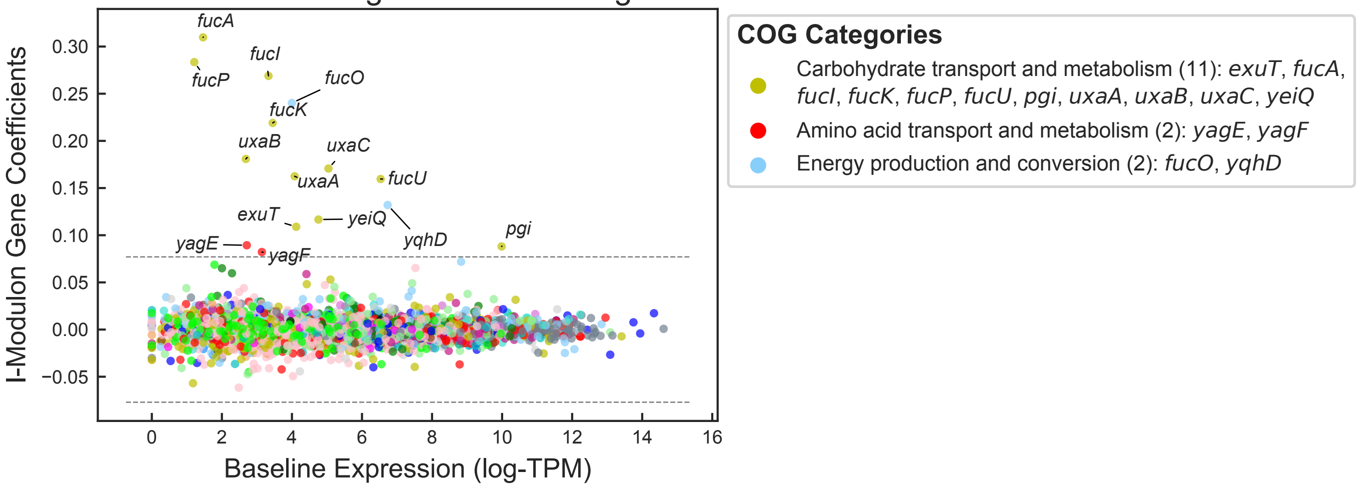

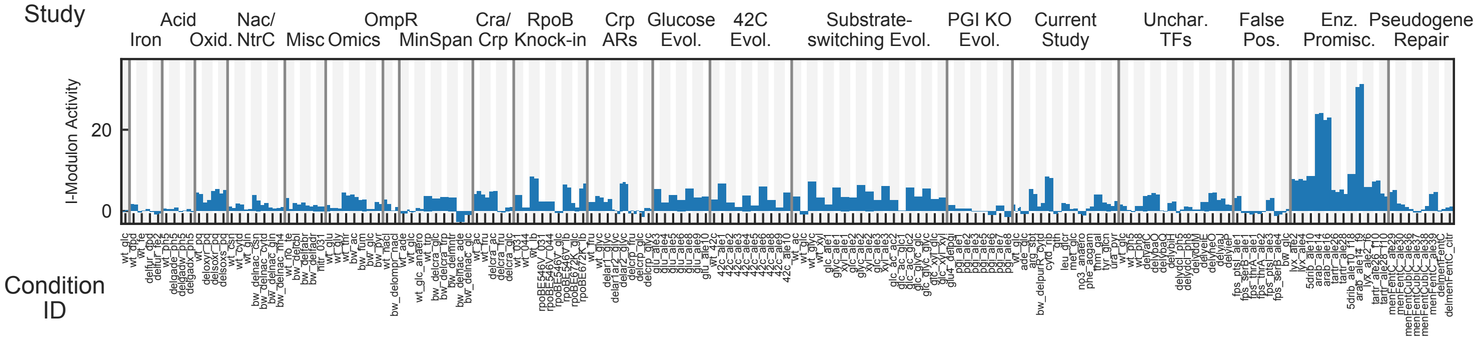

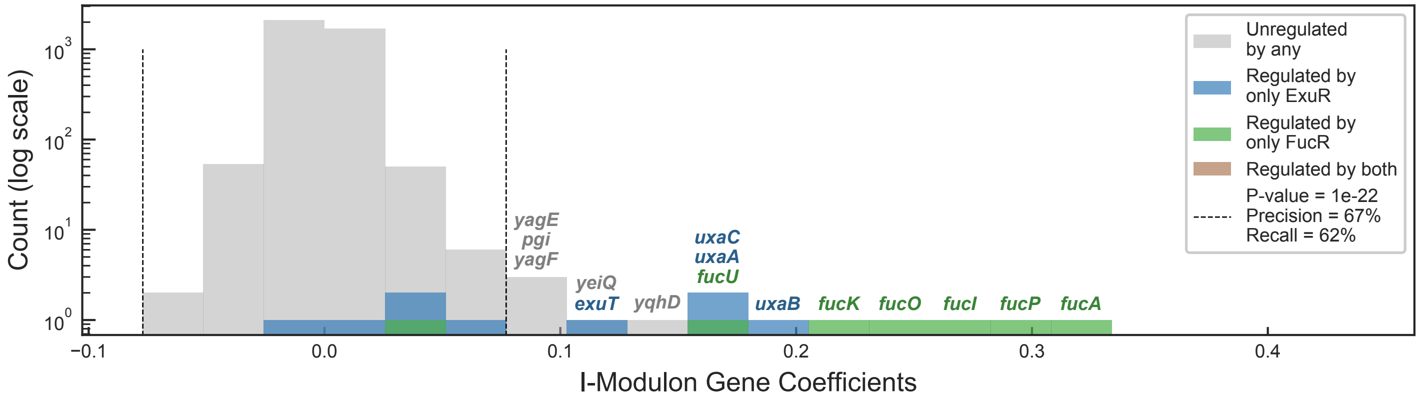

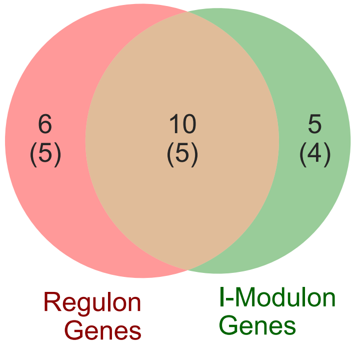

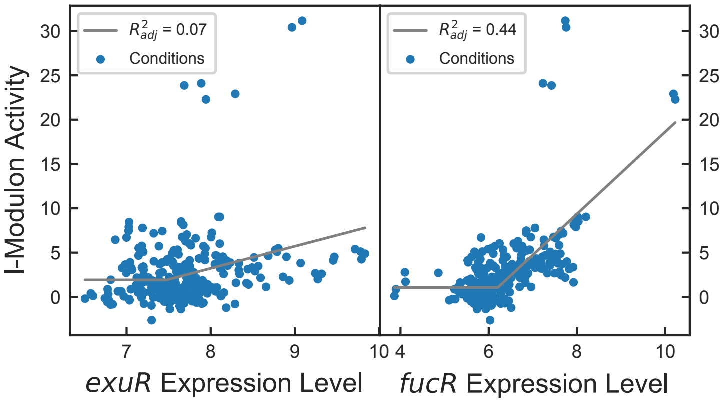

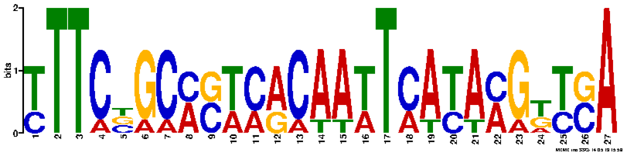

Motif E-value: 1.10e-04

Operons with Upstream Motif: 67%

FadR I-Modulon

Regulated by: FadR or IclR

Biological Function: Fatty acid degradation

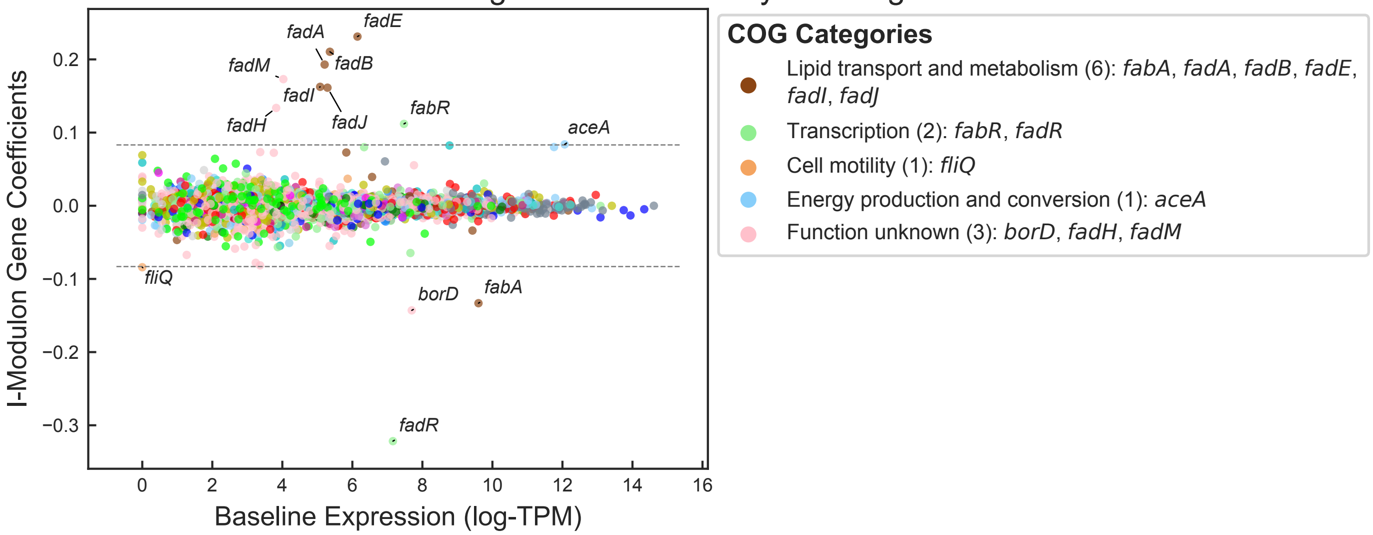

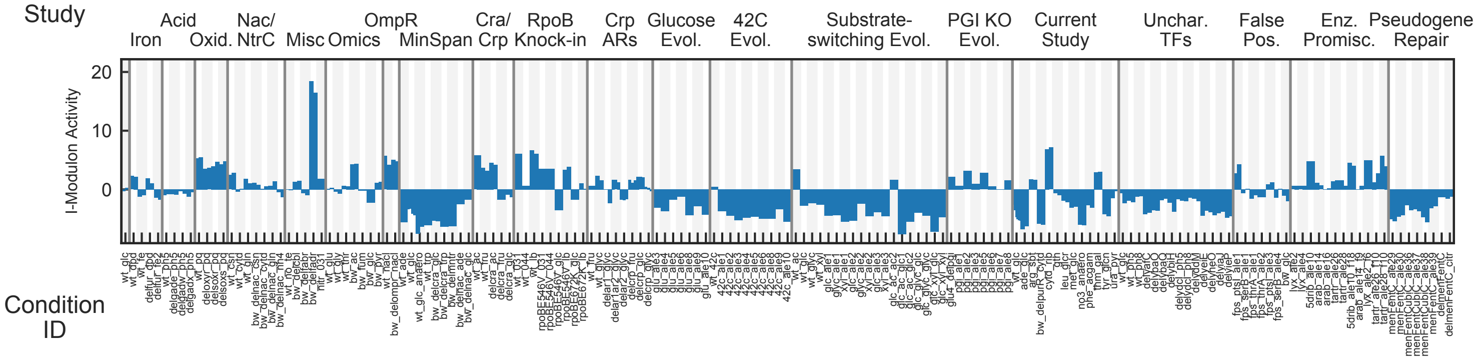

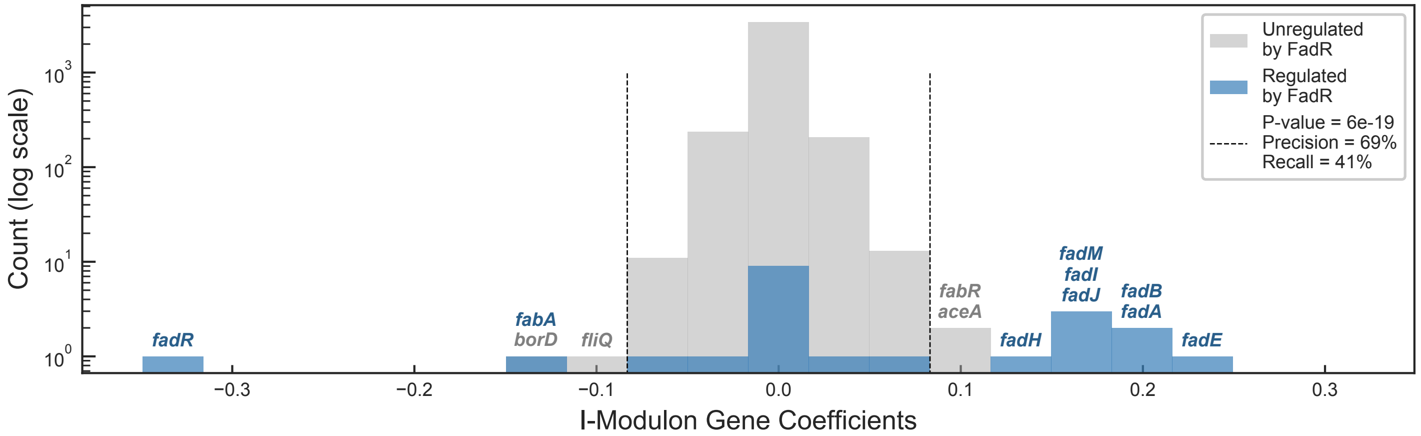

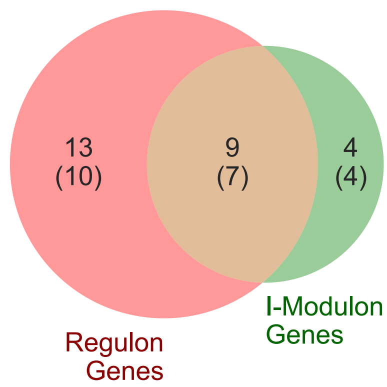

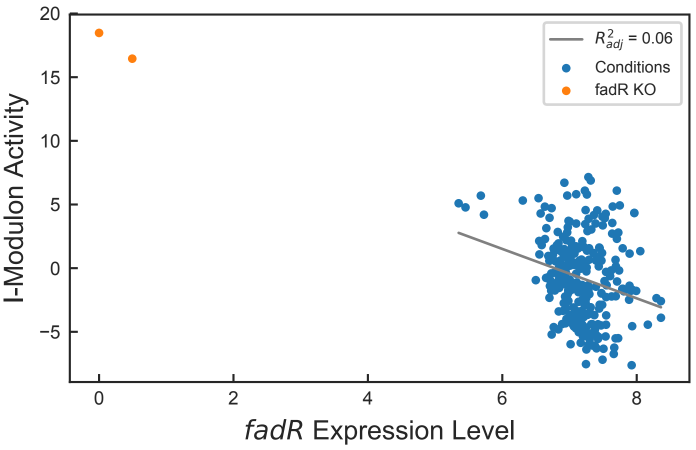

FecI I-Modulon

Regulated by: FecI

Biological Function: Ferric citrate transport

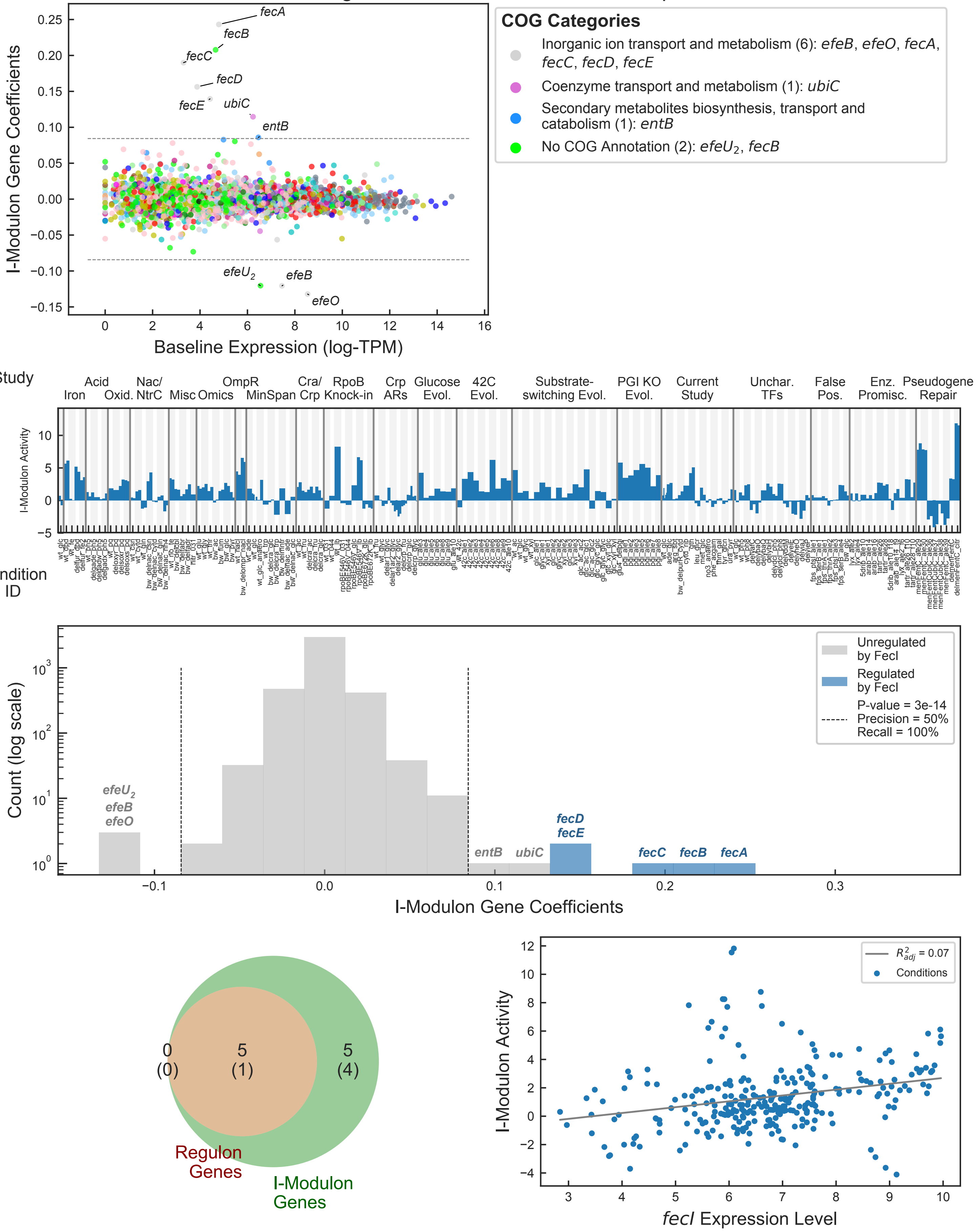

FlhDC I-Modulon

Regulated by: FlhDC
Biological Function: Flagella assembly

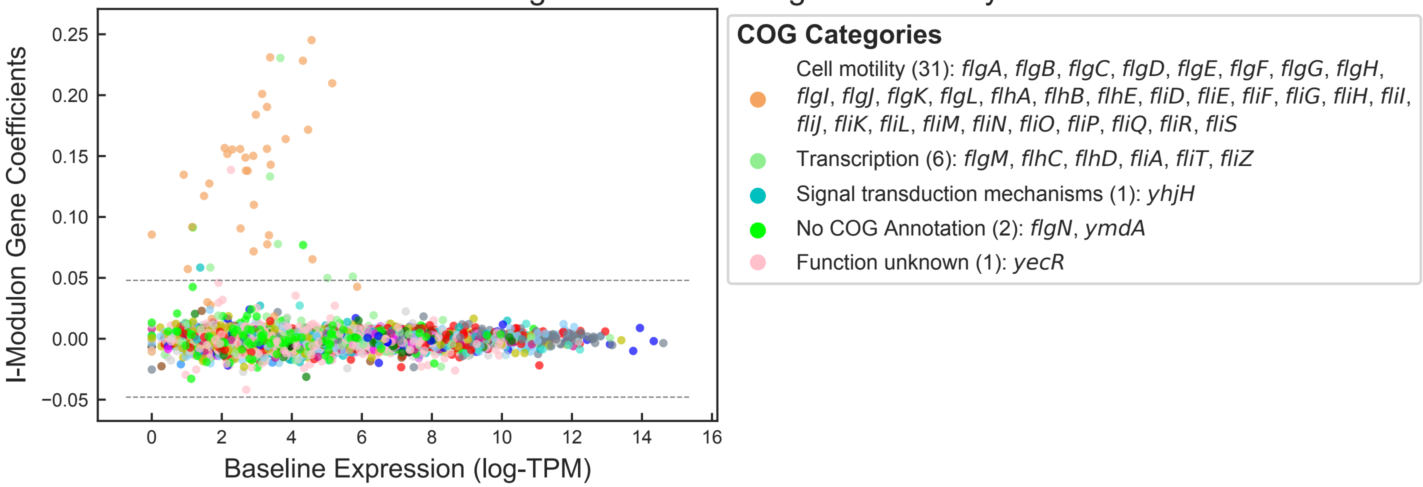

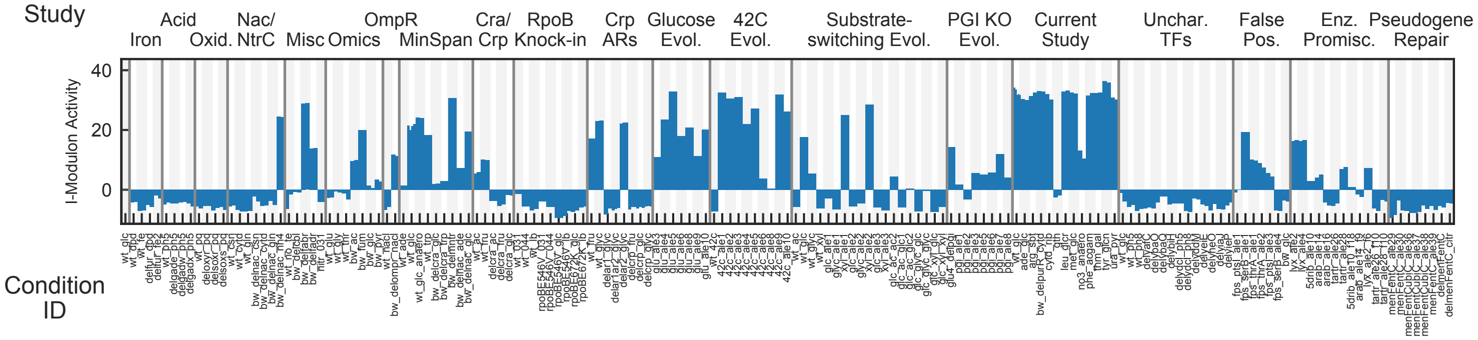

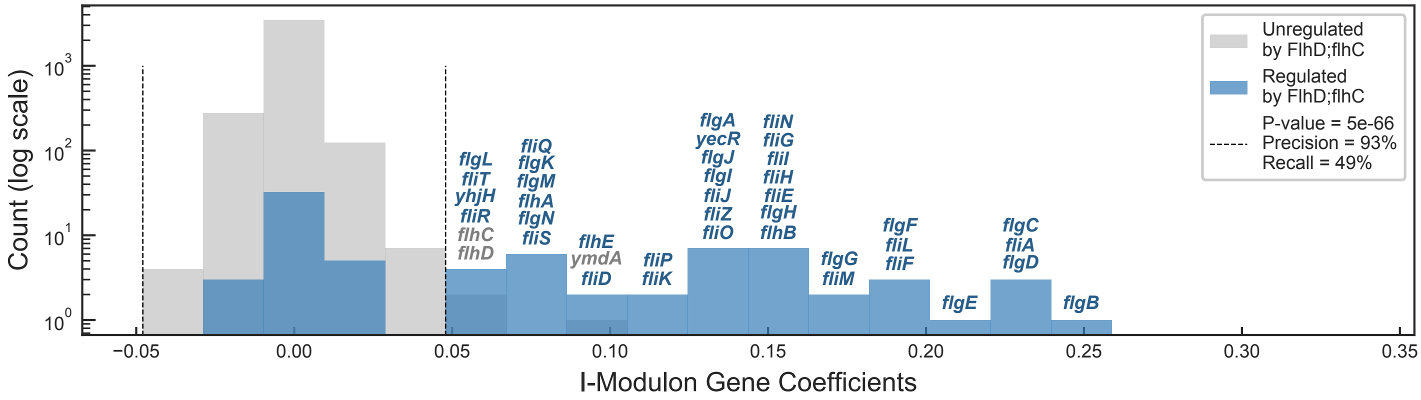

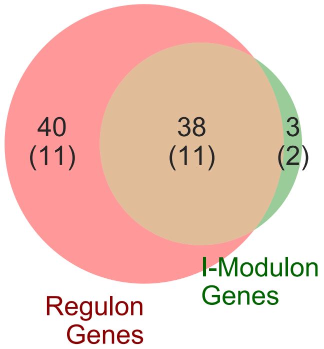

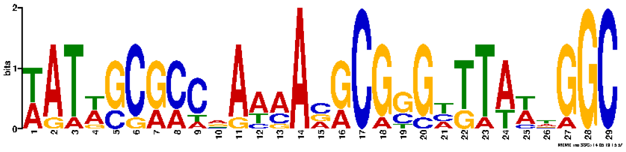

Motif E-value: 7.40e-07
Operons with Upstream Motif: 54%

FliA I-Modulon

Regulated by: FliA
Biological Function: Chemotaxis

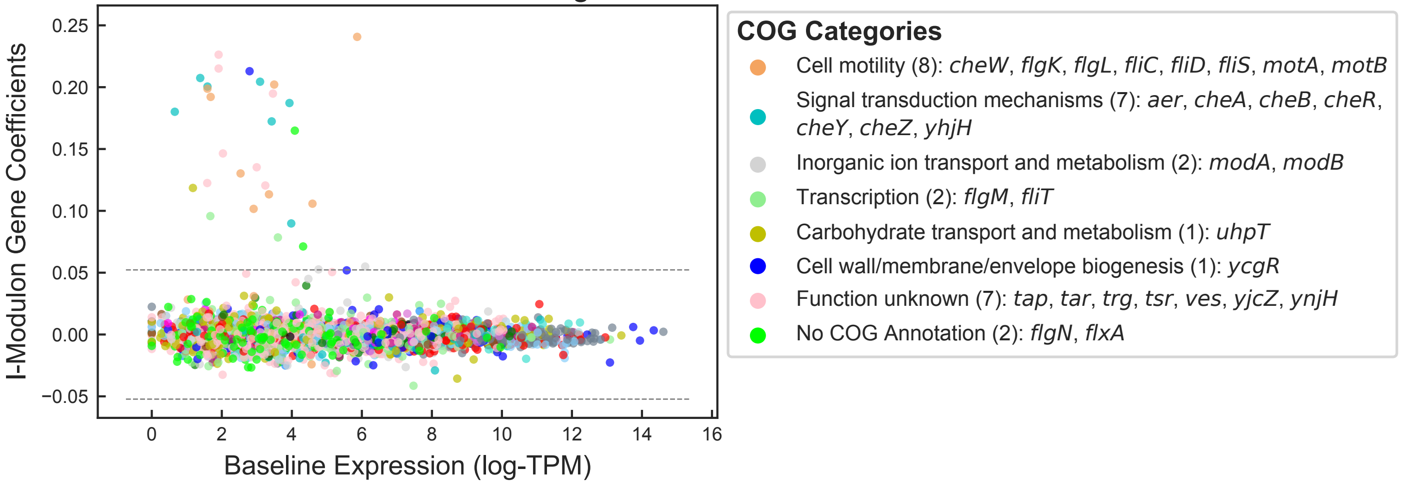

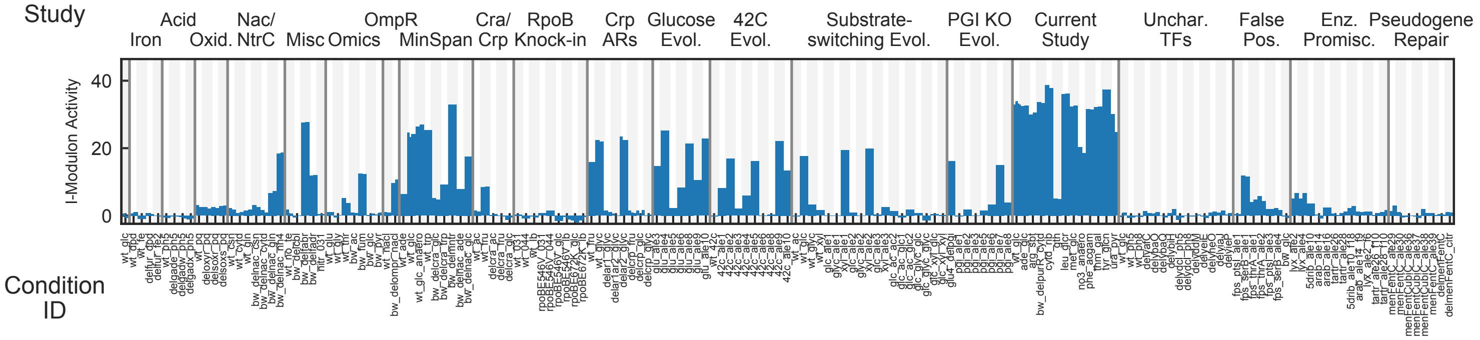

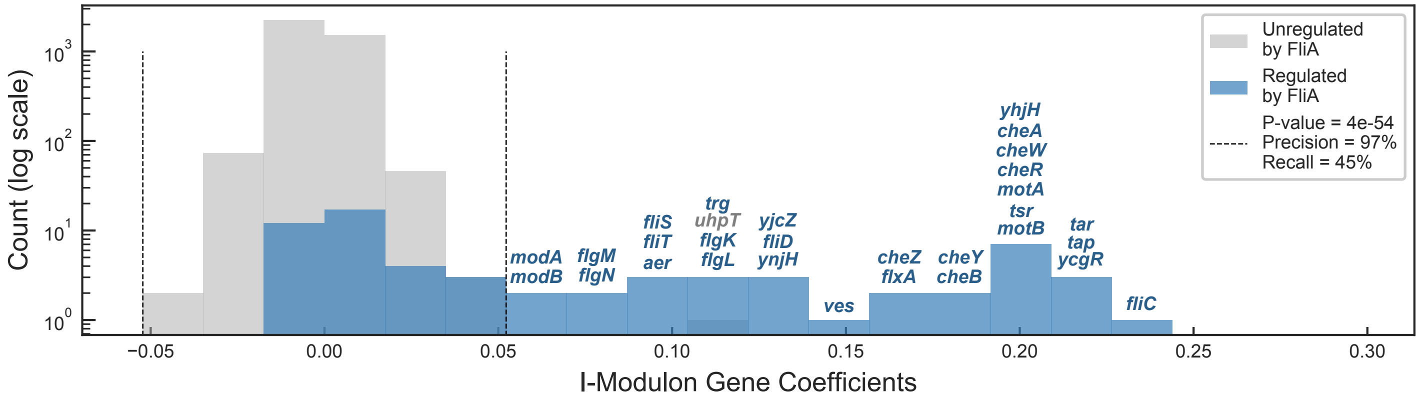

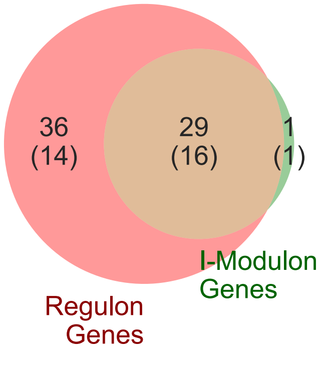

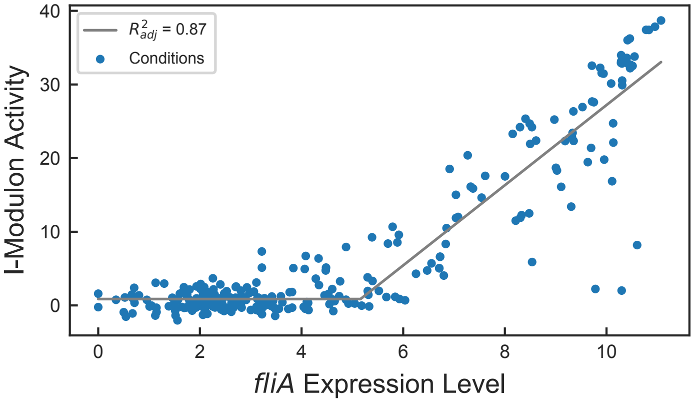

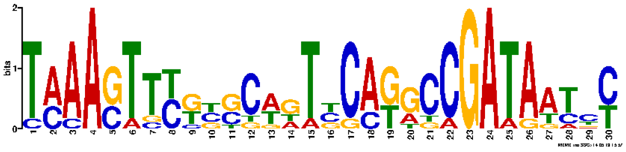

Motif E-value: 2.00e-08
Operons with Upstream Motif: 47%

Fnr I-Modulon

Regulated by: Fnr

Biological Function: Anaerobic response

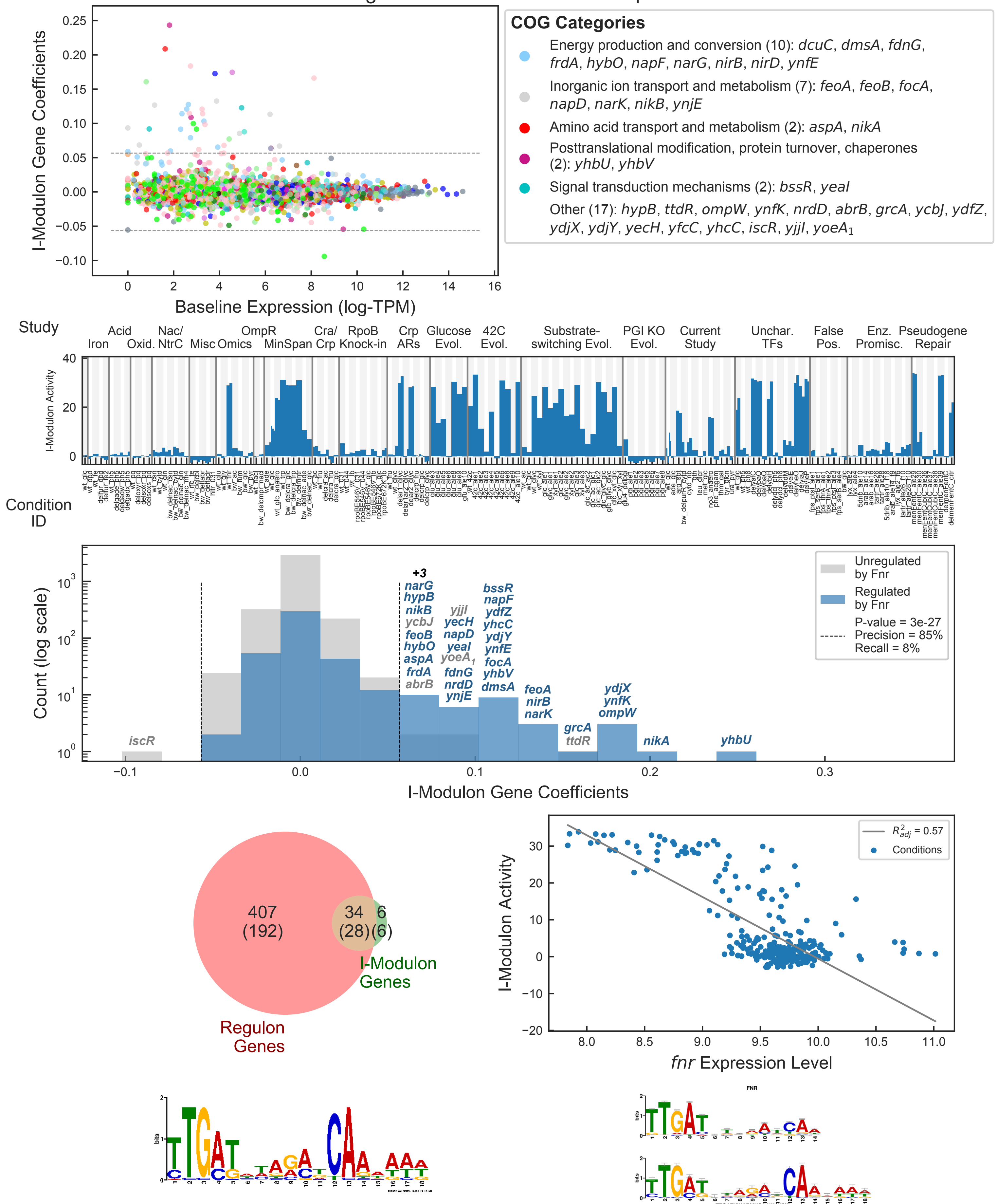

Motif E-value: 4.60e-12

Operons with Upstream Motif: 82%

Motif similar to FNR (E-value: 2.98e-06)

Fur – 1 I-Modulon

Regulated by: Fur

Biological Function: Iron homeostasis

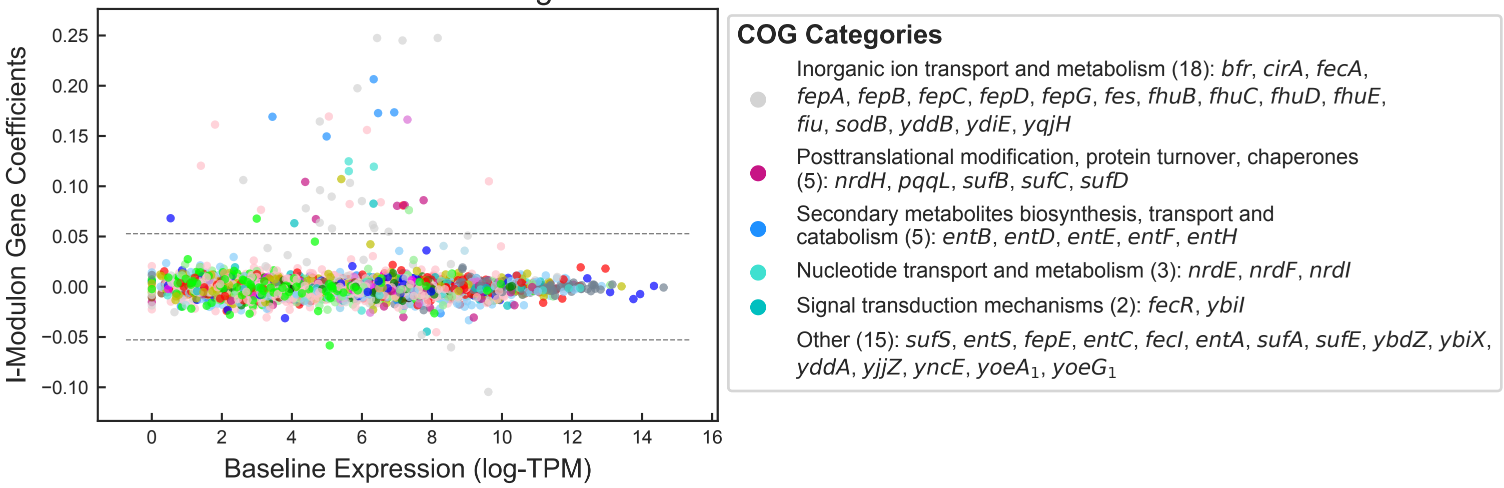

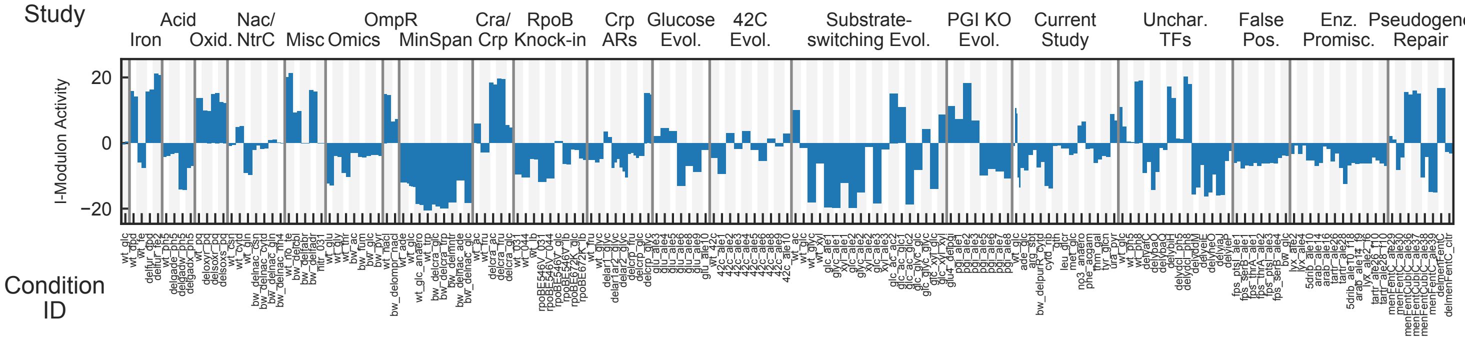

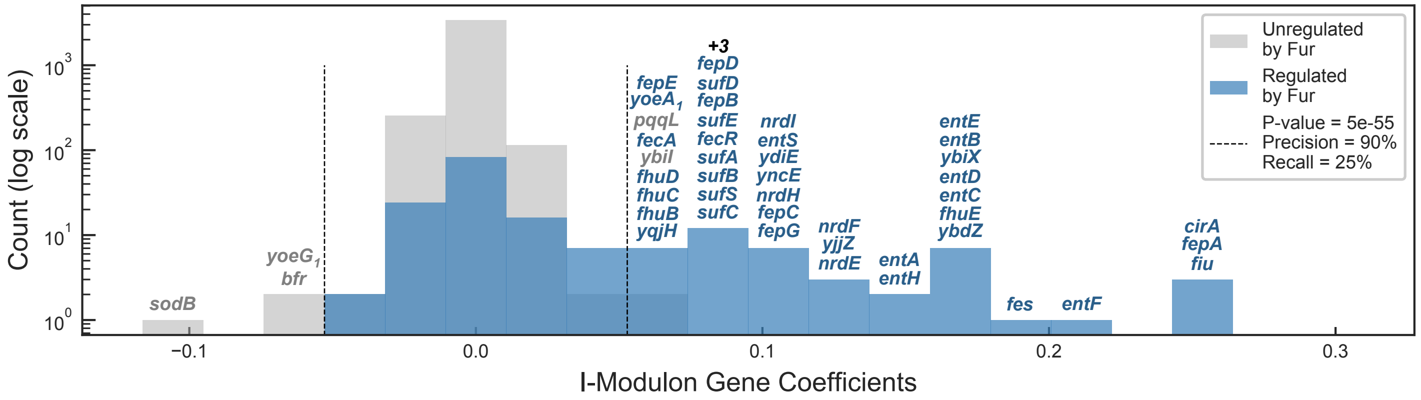

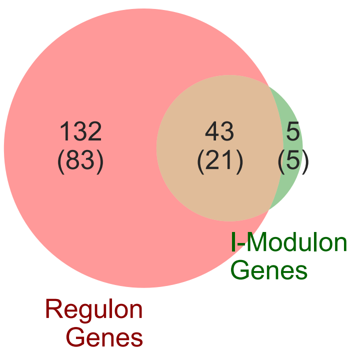

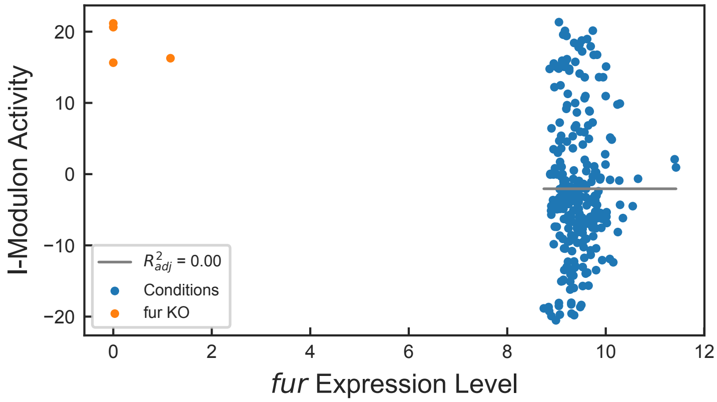

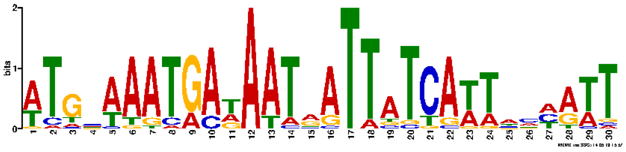

Motif E-value: 9.40e-36

Operons with Upstream Motif: 69%

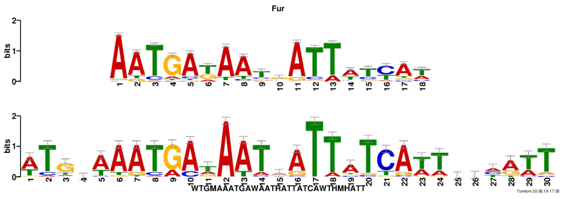

Motif similar to Fur (E-value: 1.75e-10)

Fur – 2 I-Modulon

Regulated by: Fur
Biological Function: Iron homeostasis

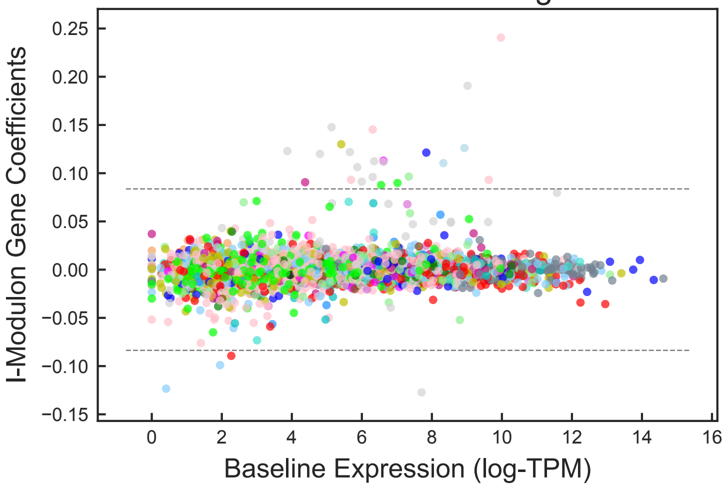

COG Categories

- Inorganic ion transport and metabolism (11): *feoA*, *feoB*, *fepB*, *fepC*, *fepD*, *fepG*, *fes*, *fhuA*, *fhuC*, *fhuD*, *ftnA*
- Energy production and conversion (2): *ydhV*, *ydhY*
- Intracellular trafficking, secretion, and vesicular transport (2): *exbB*, *exbD*
- Amino acid transport and metabolism (1): *preT*
- Carbohydrate transport and metabolism (1): *entS*
- Cell wall/membrane/envelope biogenesis (1): *tonB*
- Other (9): *yojI*, *nrdH*, *fecI*, *bfd*, *fhuF*, *ybaN*, *yncE*, *efeU*$_2$, *gadW*

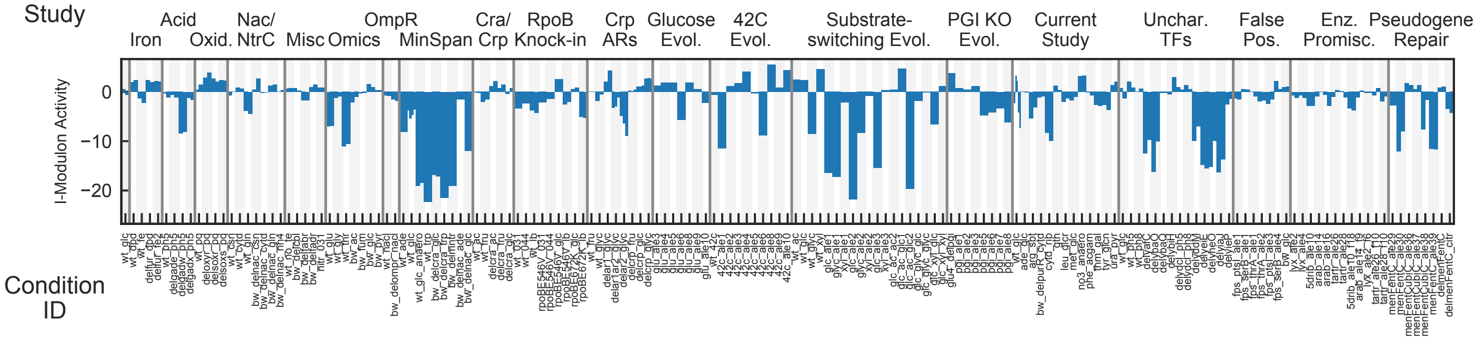

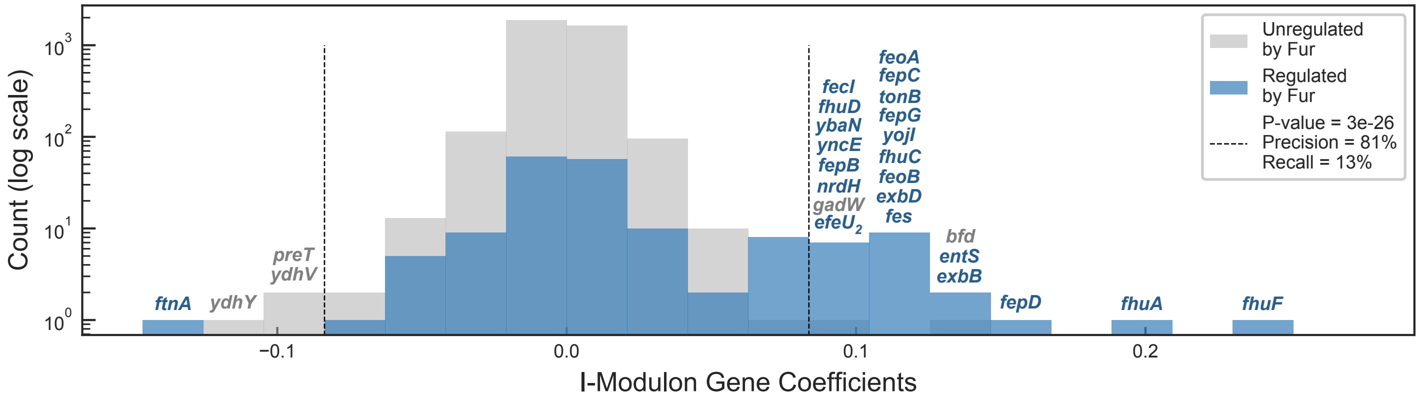

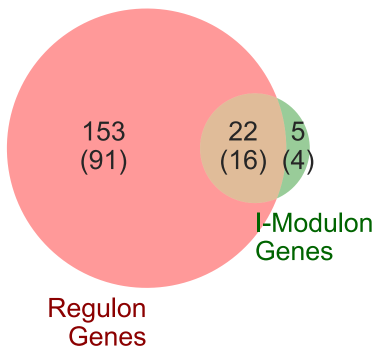

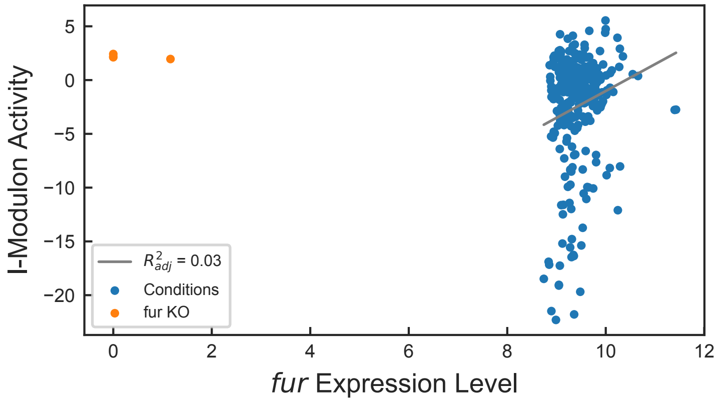

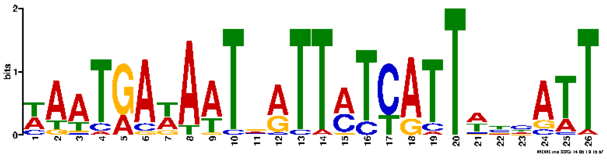

Motif E-value: 3.40e-32
Operons with Upstream Motif: 90%

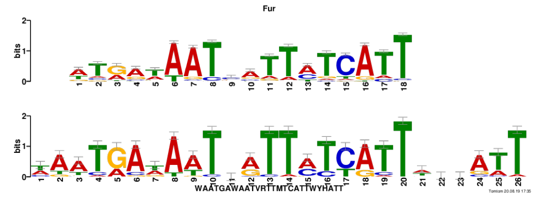

Motif similar to Fur (E-value: 2.42e-11)

GadEWX I-Modulon

Regulated by: GadE and GadW and GadX

Biological Function: Acid stress response

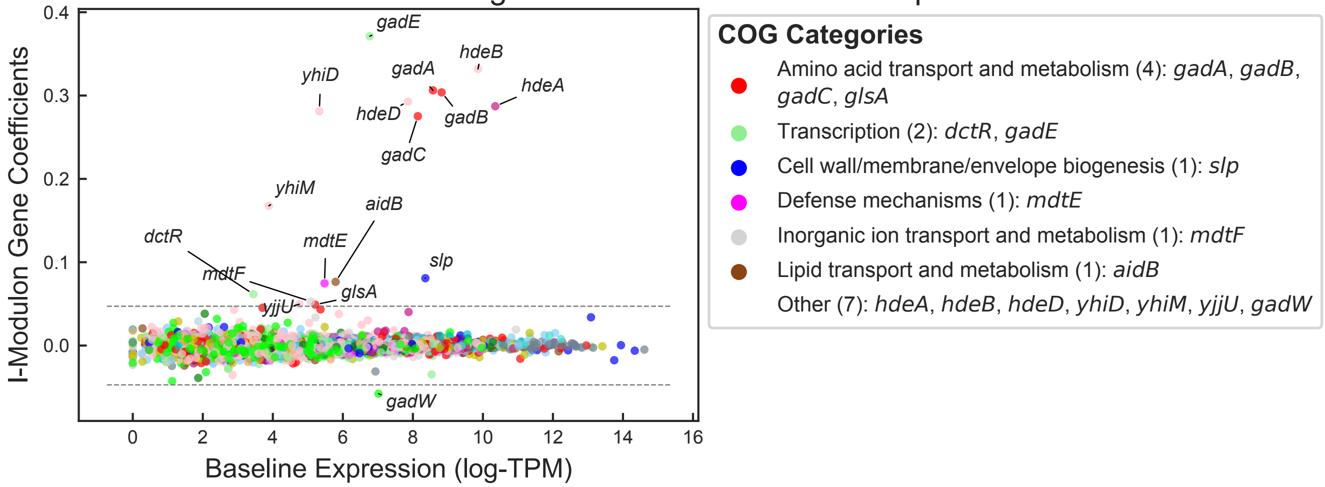

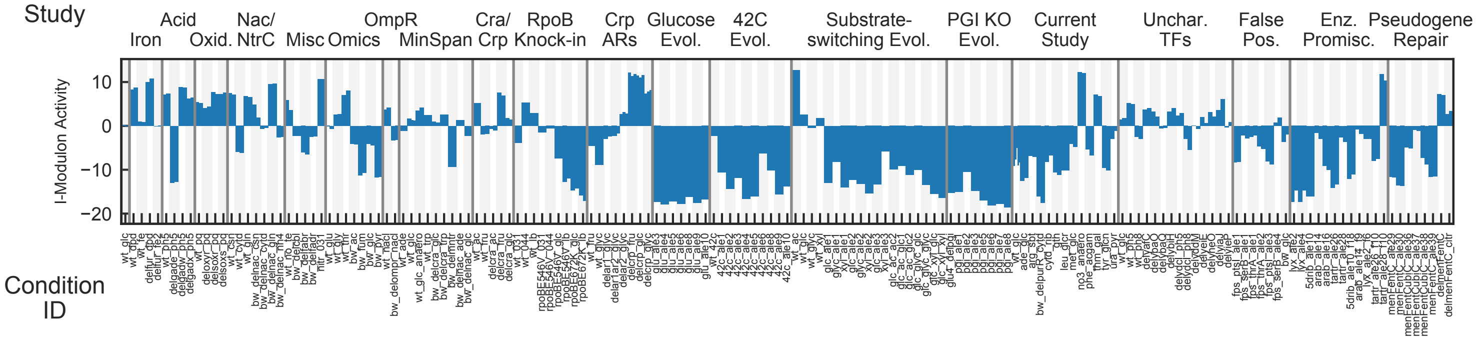

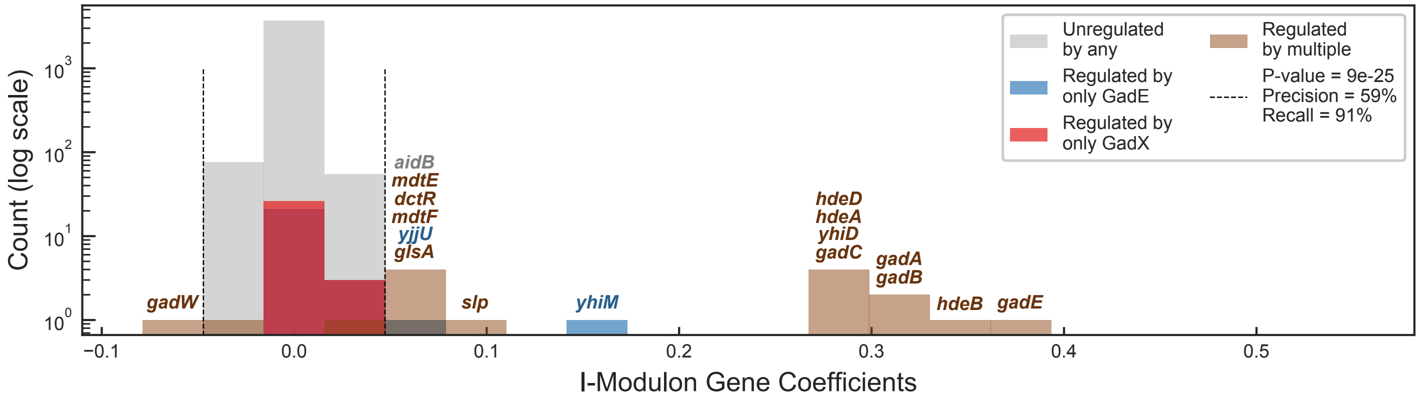

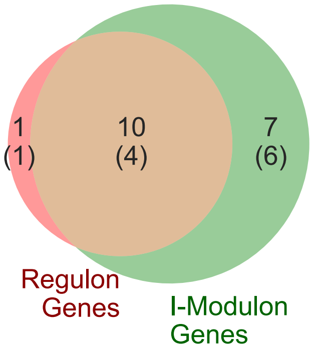

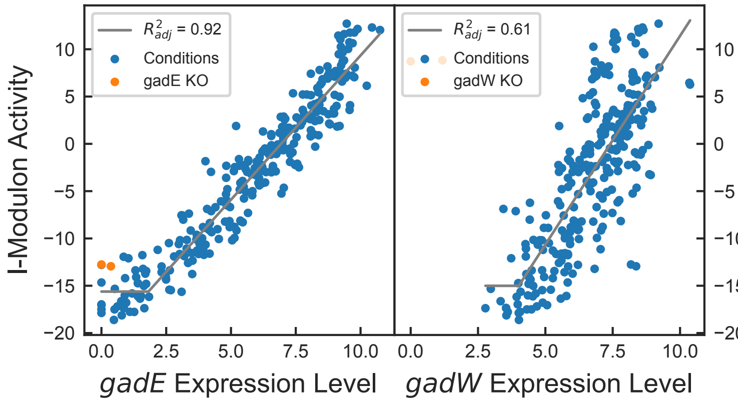

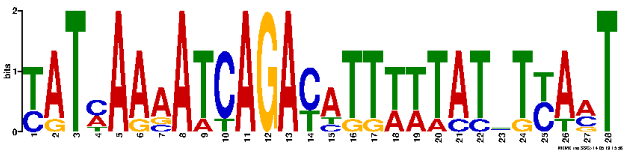

Motif E-value: 8.00e-04

Operons with Upstream Motif: 60%

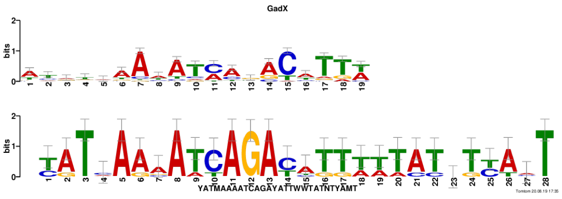

Motif similar to GadX (E-value: 5.32e-04)

GadWX I-Modulon

Regulated by: GadW and GadX

Biological Function: Acid stress response

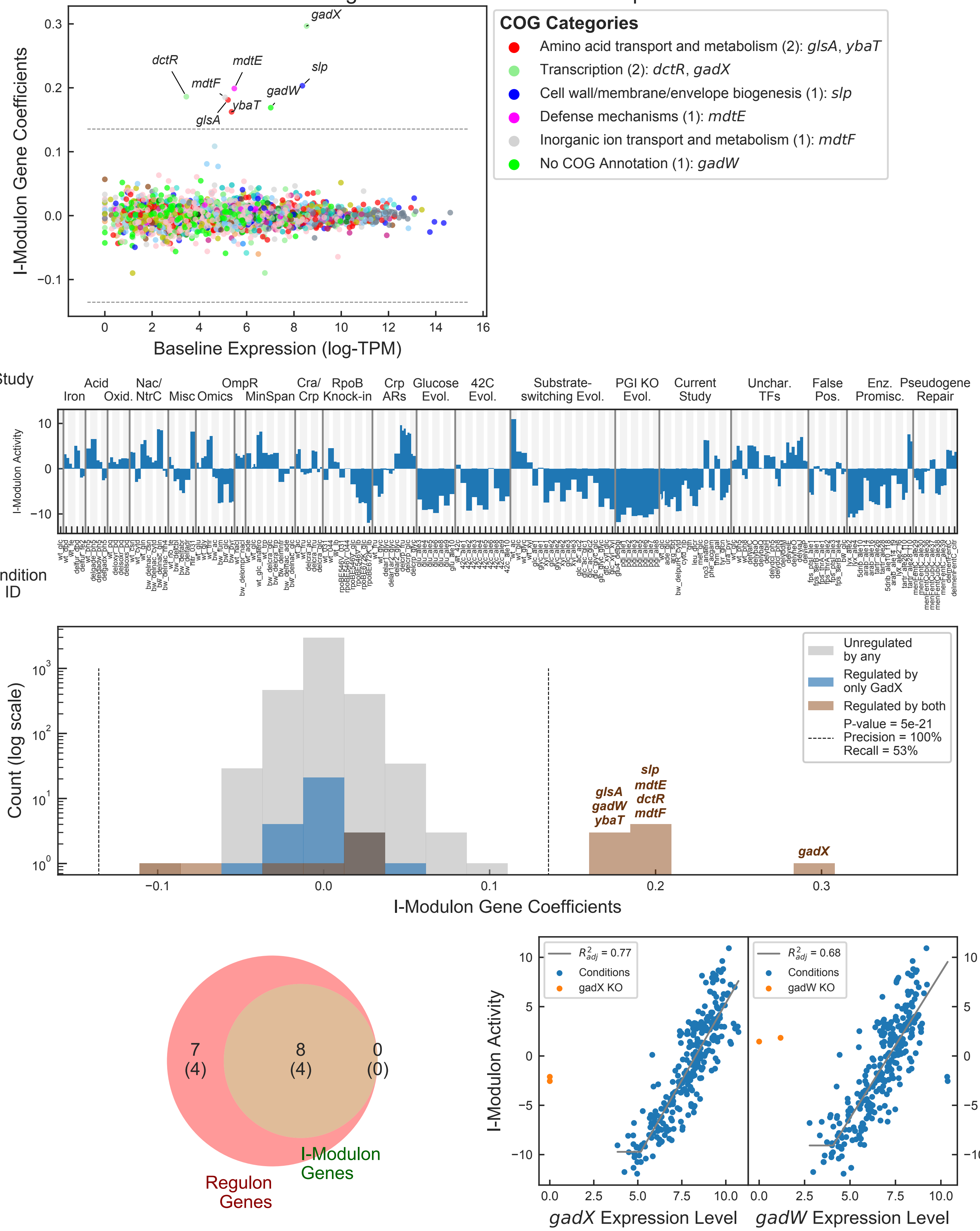

GcvA I-Modulon

Regulated by: GcvA

Biological Function: Glycine cleavage system

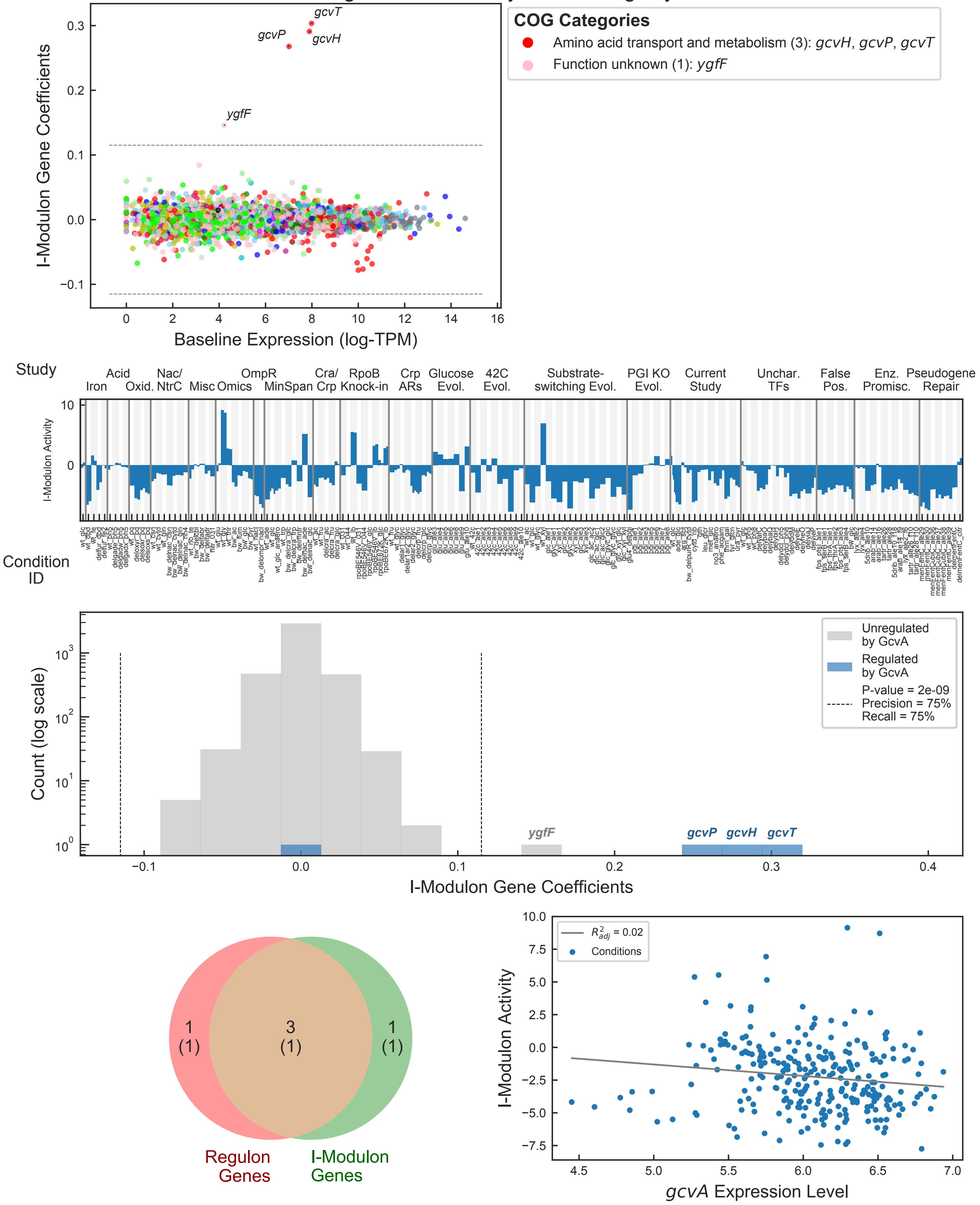

GlcC I-Modulon

Regulated by: GlcC

Biological Function: Glycolate catabolism

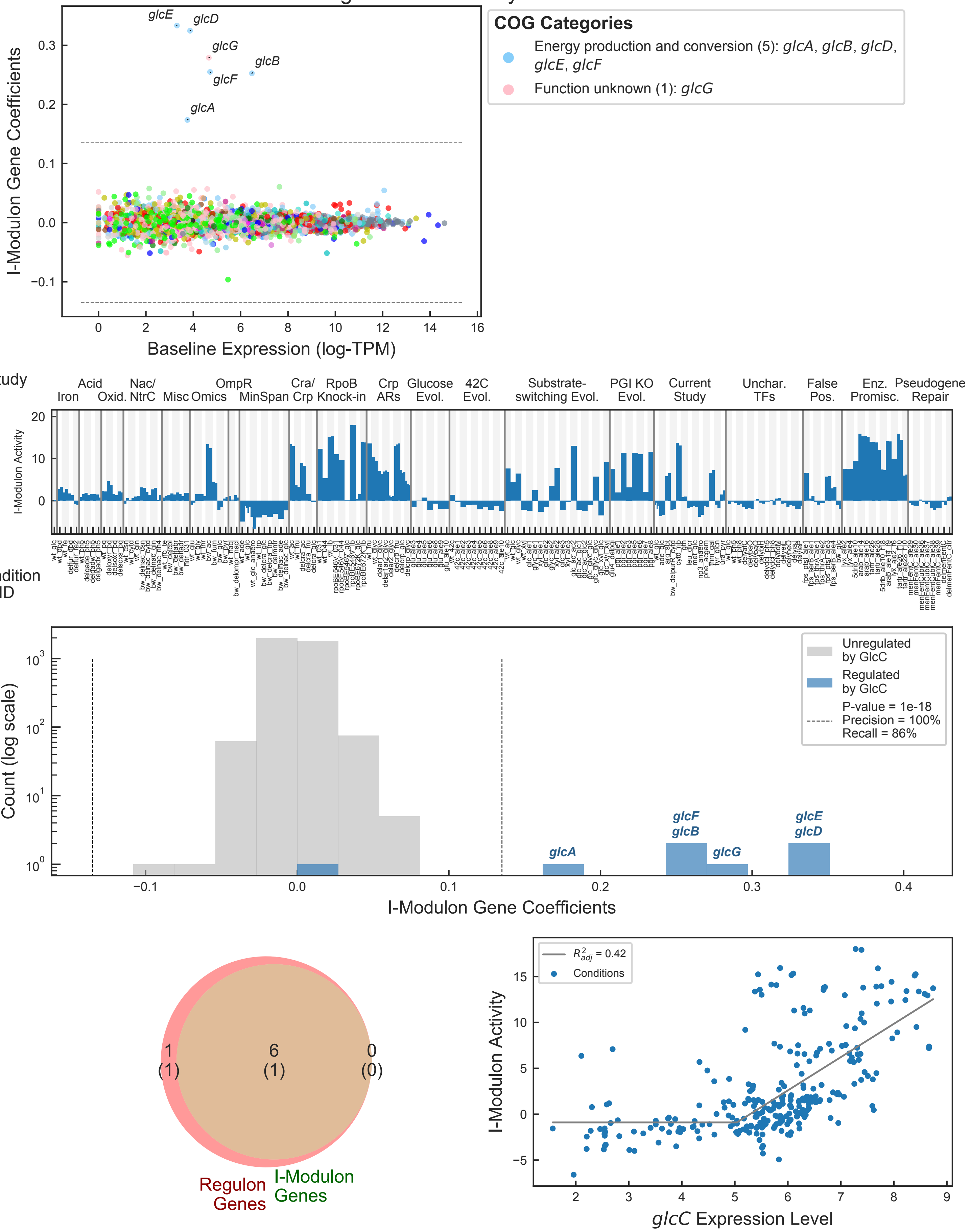

GlpR I-Modulon

Regulated by: GlpR

Biological Function: Glycerol catabolism

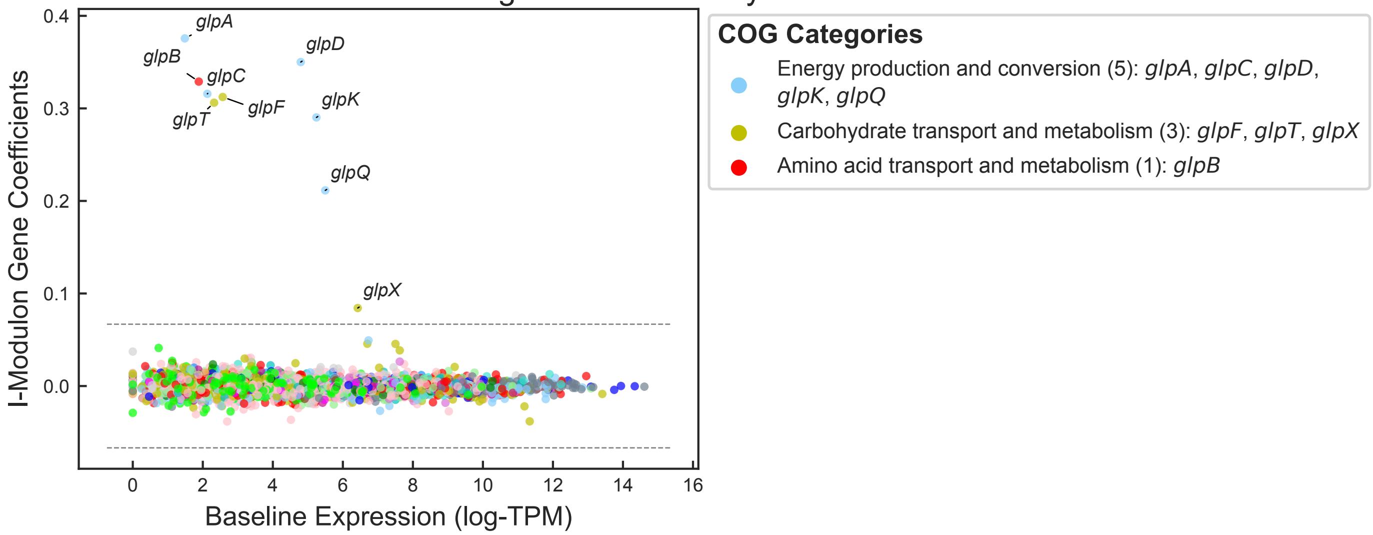

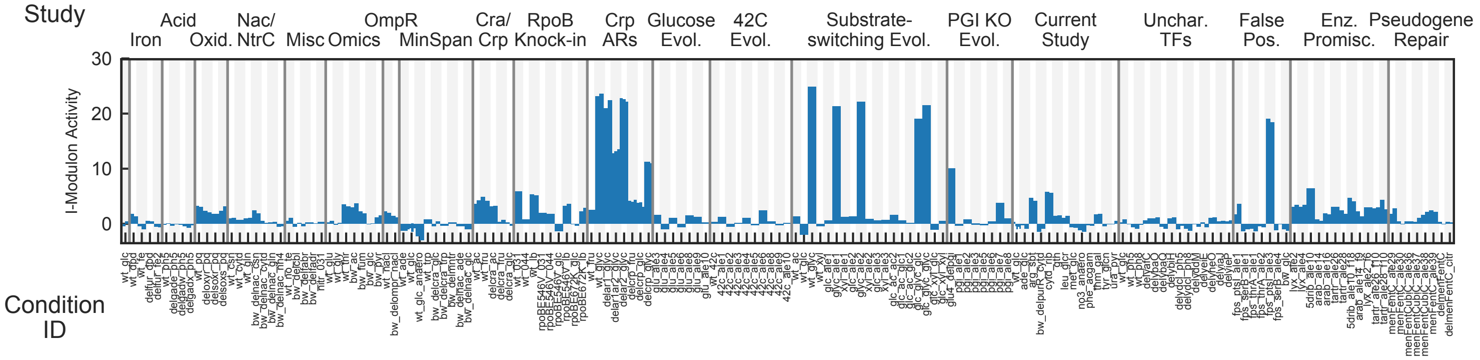

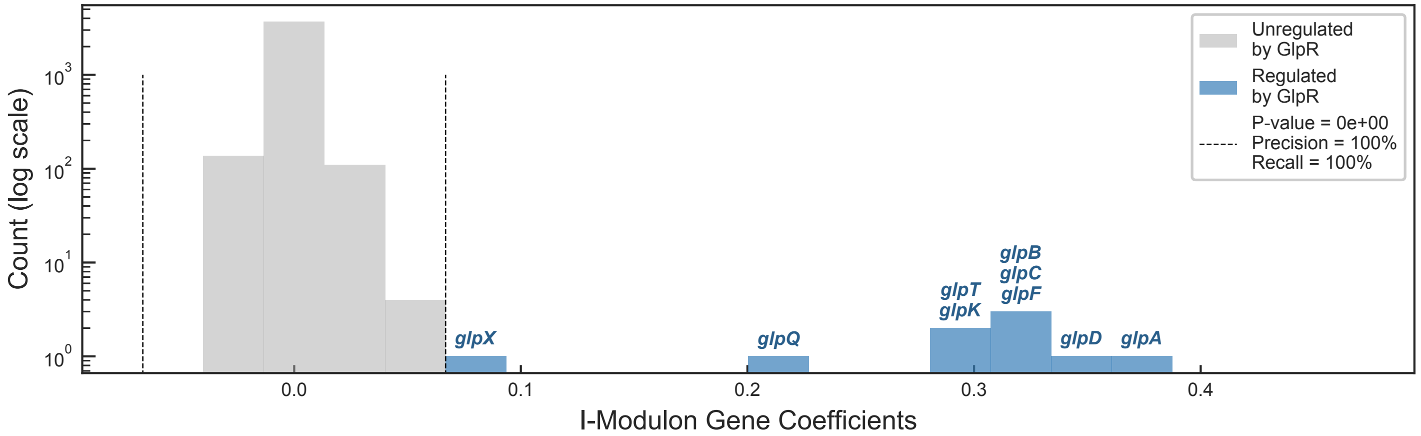

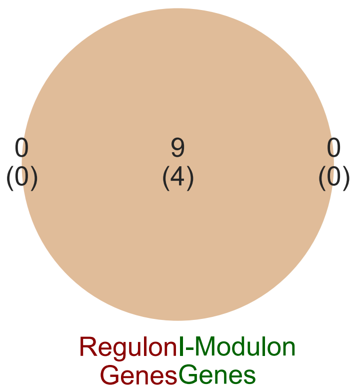

GntR/TyrR I-Modulon

Regulated by: GntR or TyrR

Biological Function: Gluconate catabolism and tyrosine biosynthesis

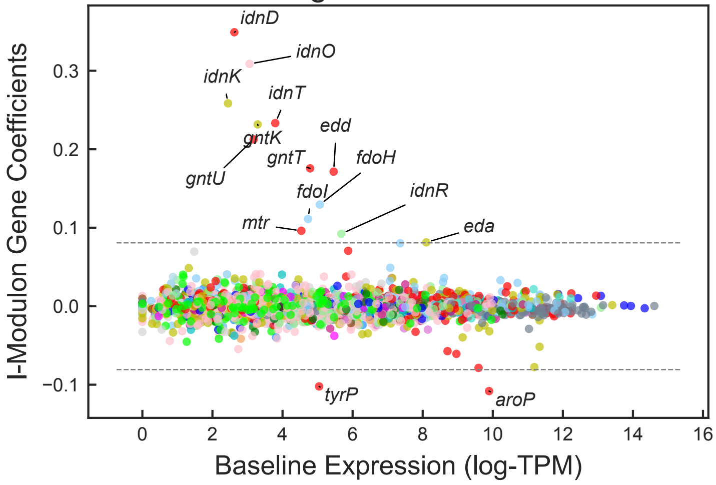

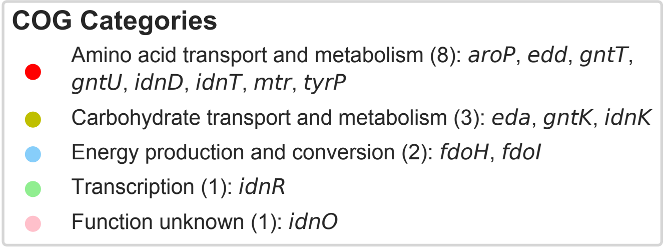

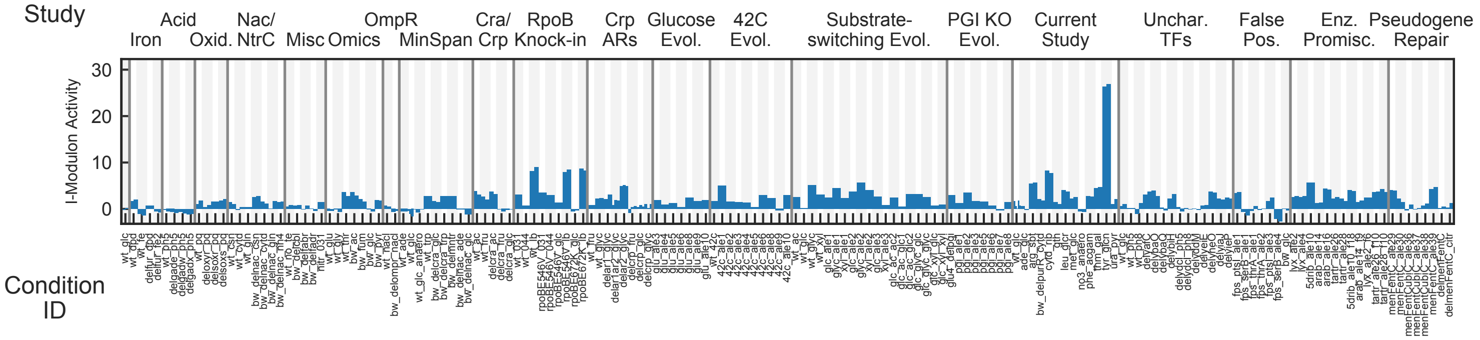

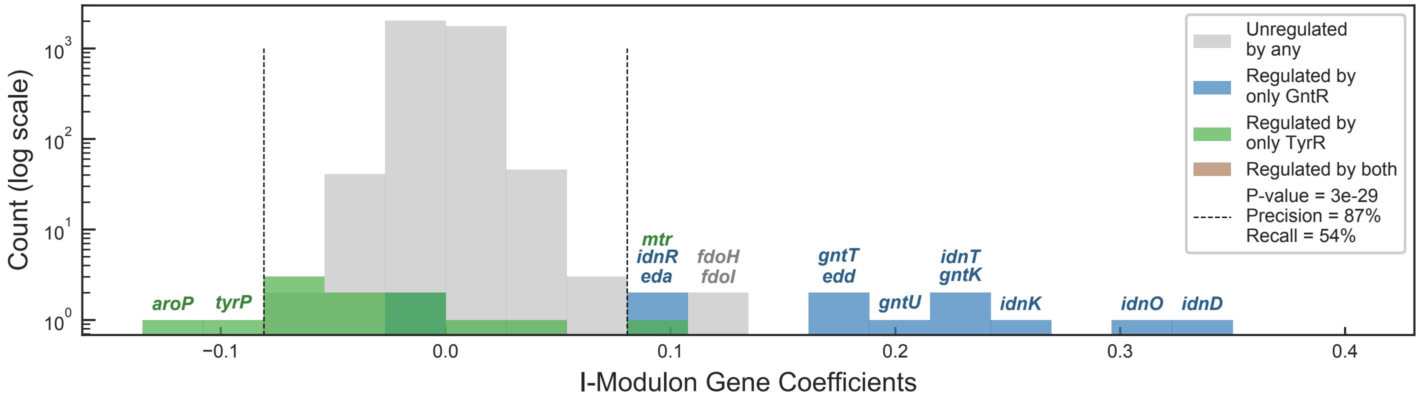

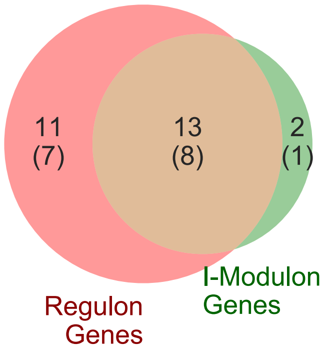

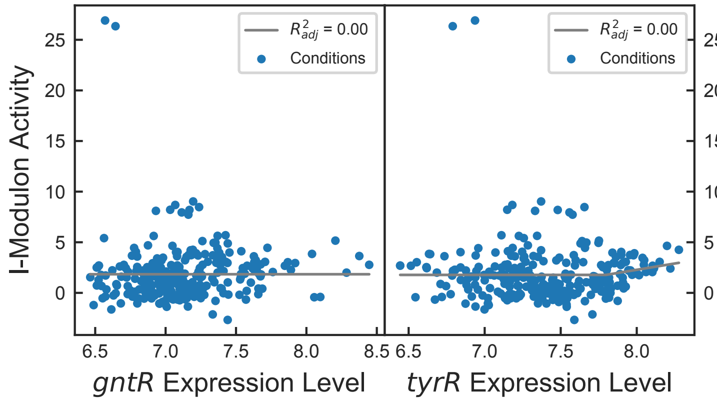

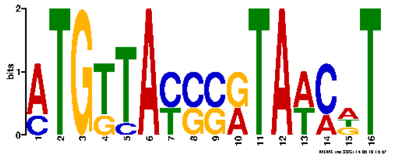

Motif E-value: 4.30e-04

Operons with Upstream Motif: 78%

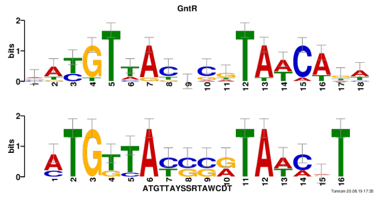

Motif similar to GntR (E-value: 3.25e-06)

His − tRNA I-Modulon

Regulated by: His-tRNA attenuation

Biological Function: Histidine biosynthesis

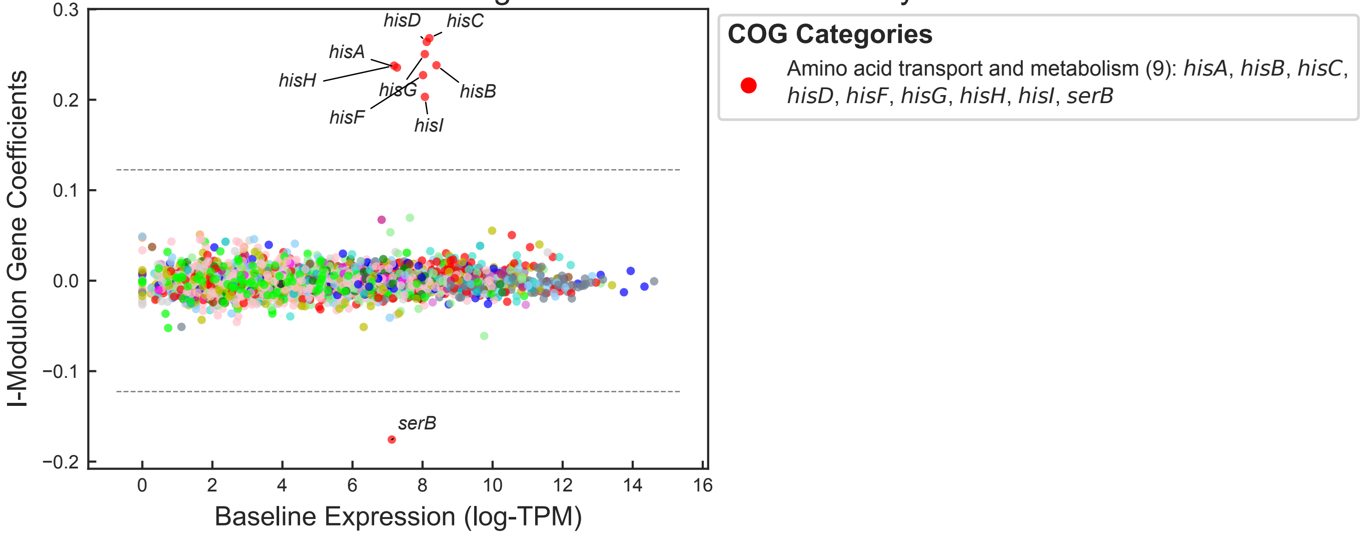

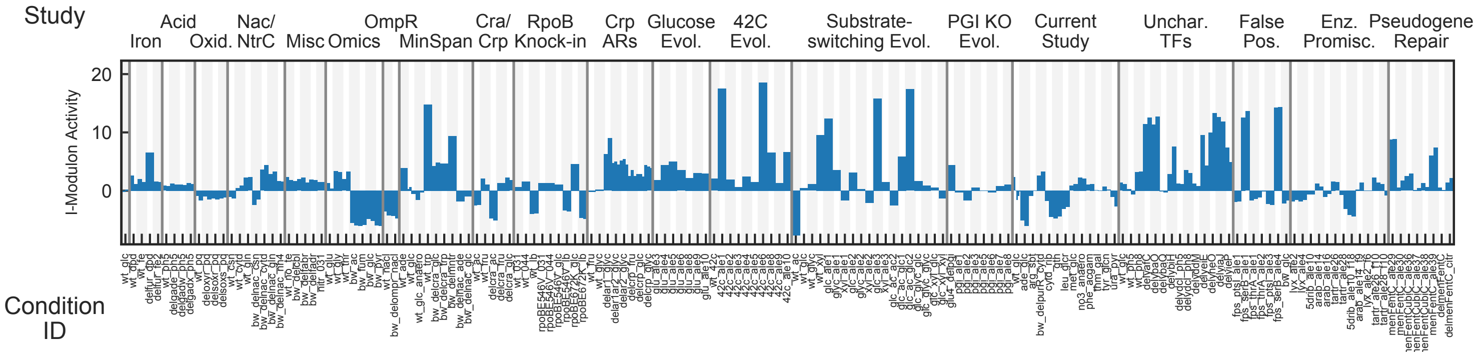

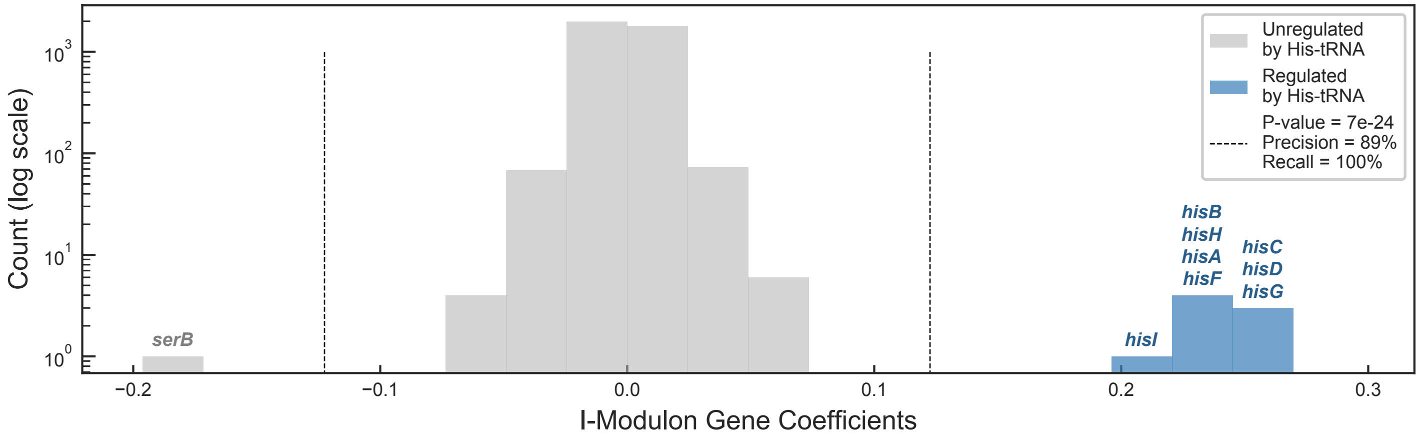

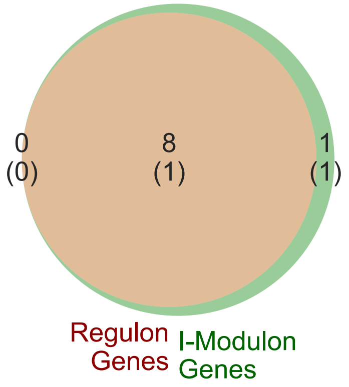

Leu/Ile I-Modulon

Regulated by: IlvY or leu-tRNA attenuation or ile-tRNA attenuation
Biological Function: Branched-chain amino acid biosynthesis

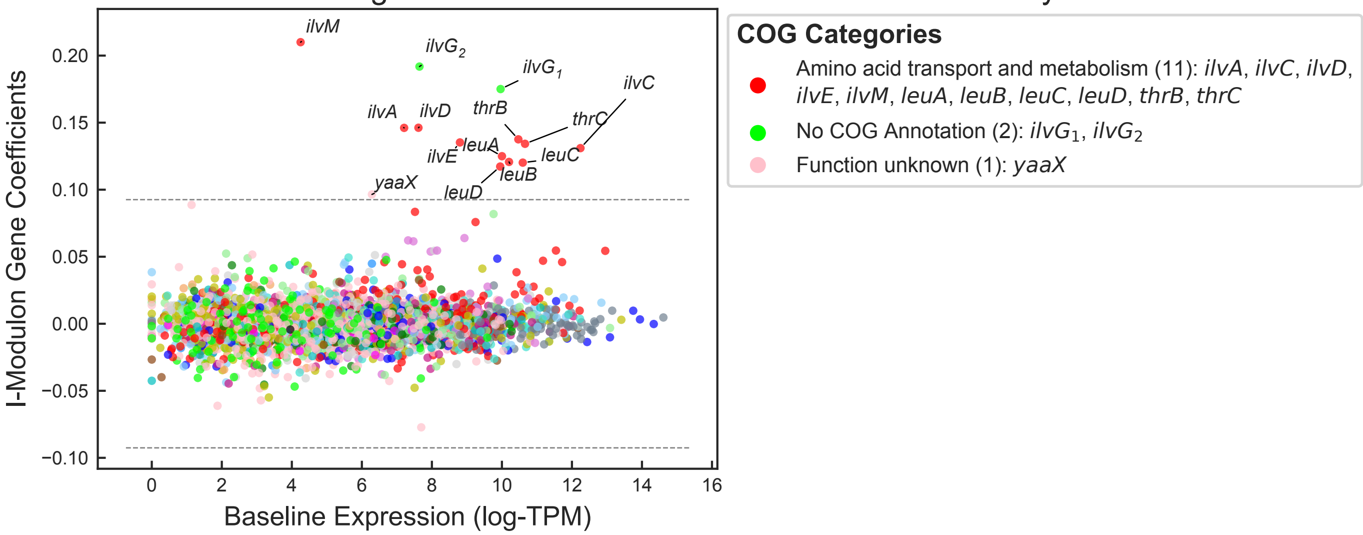

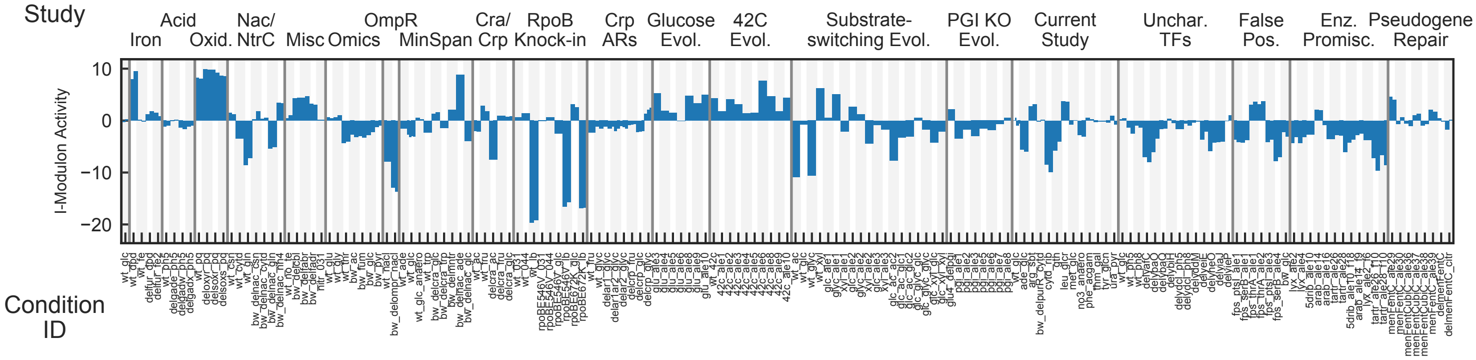

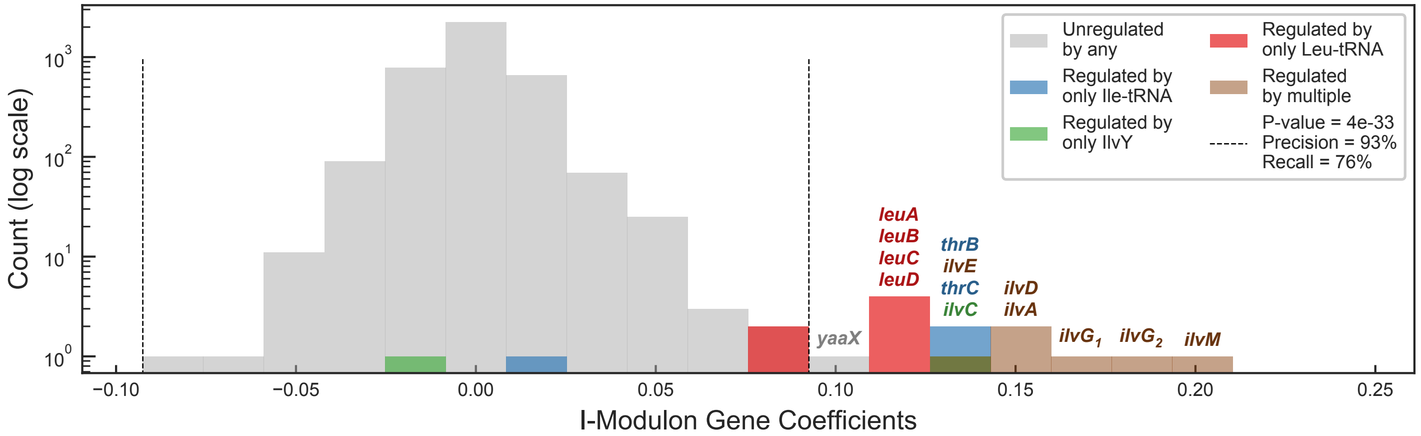

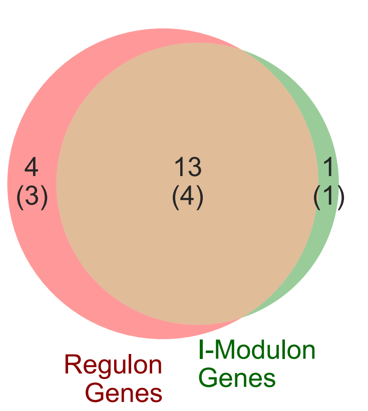

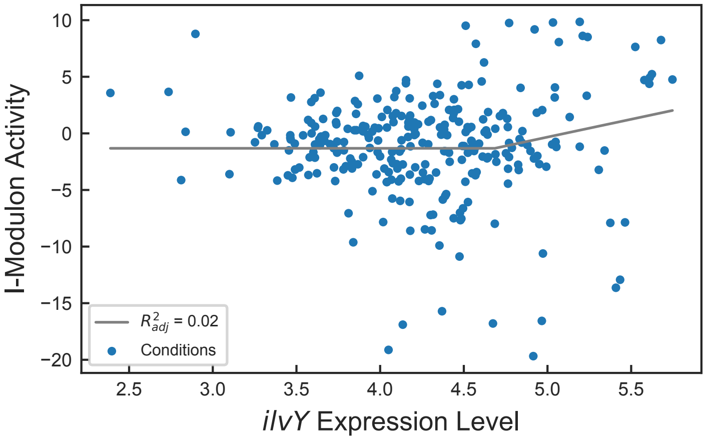

Lrp I-Modulon

Regulated by: Lrp

Biological Function: Amino acid and peptide transport

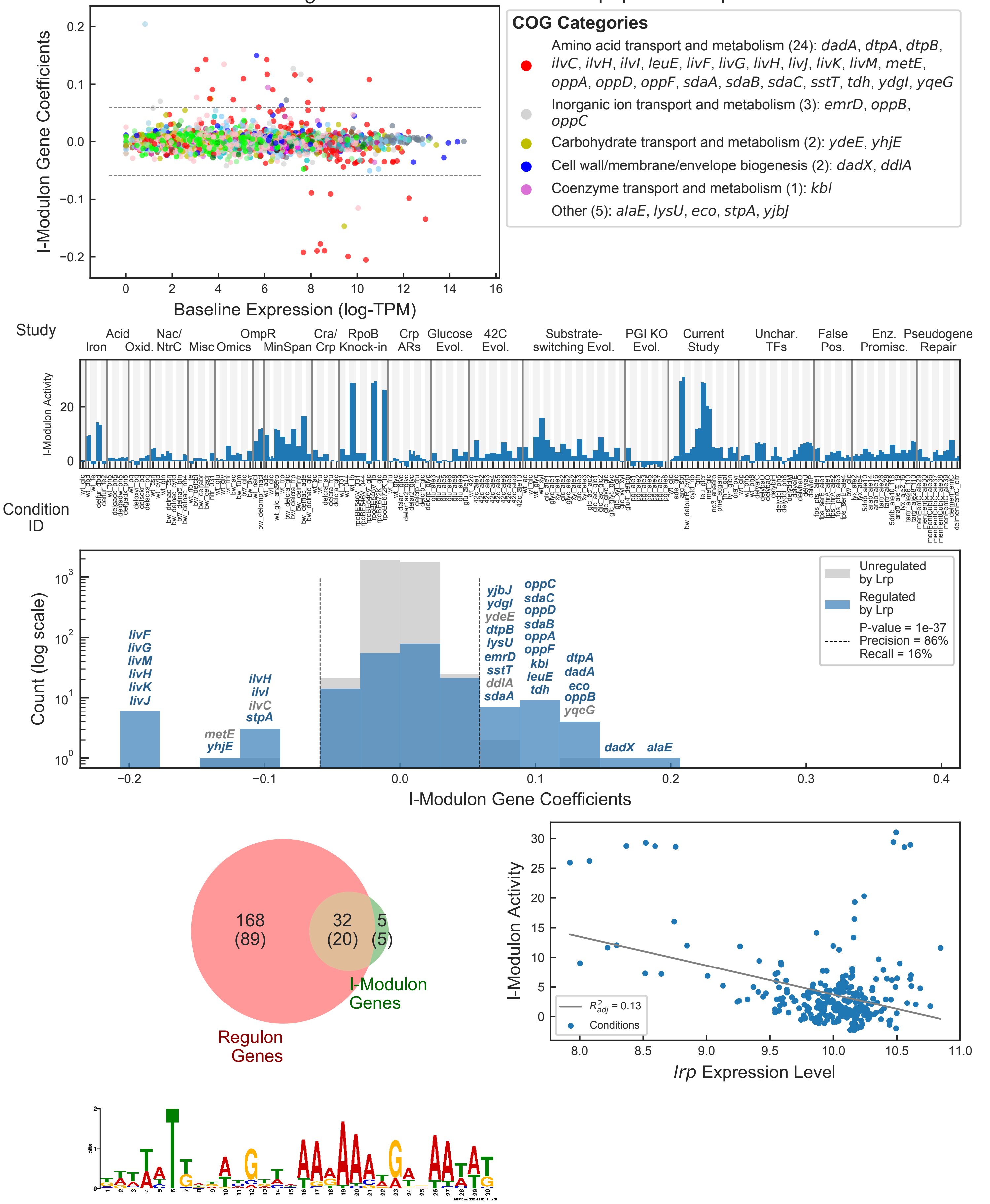

Motif E-value: 1.20e-04

Operons with Upstream Motif: 72%

MalT I-Modulon

Regulated by: MalT

Biological Function: Maltose catabolism

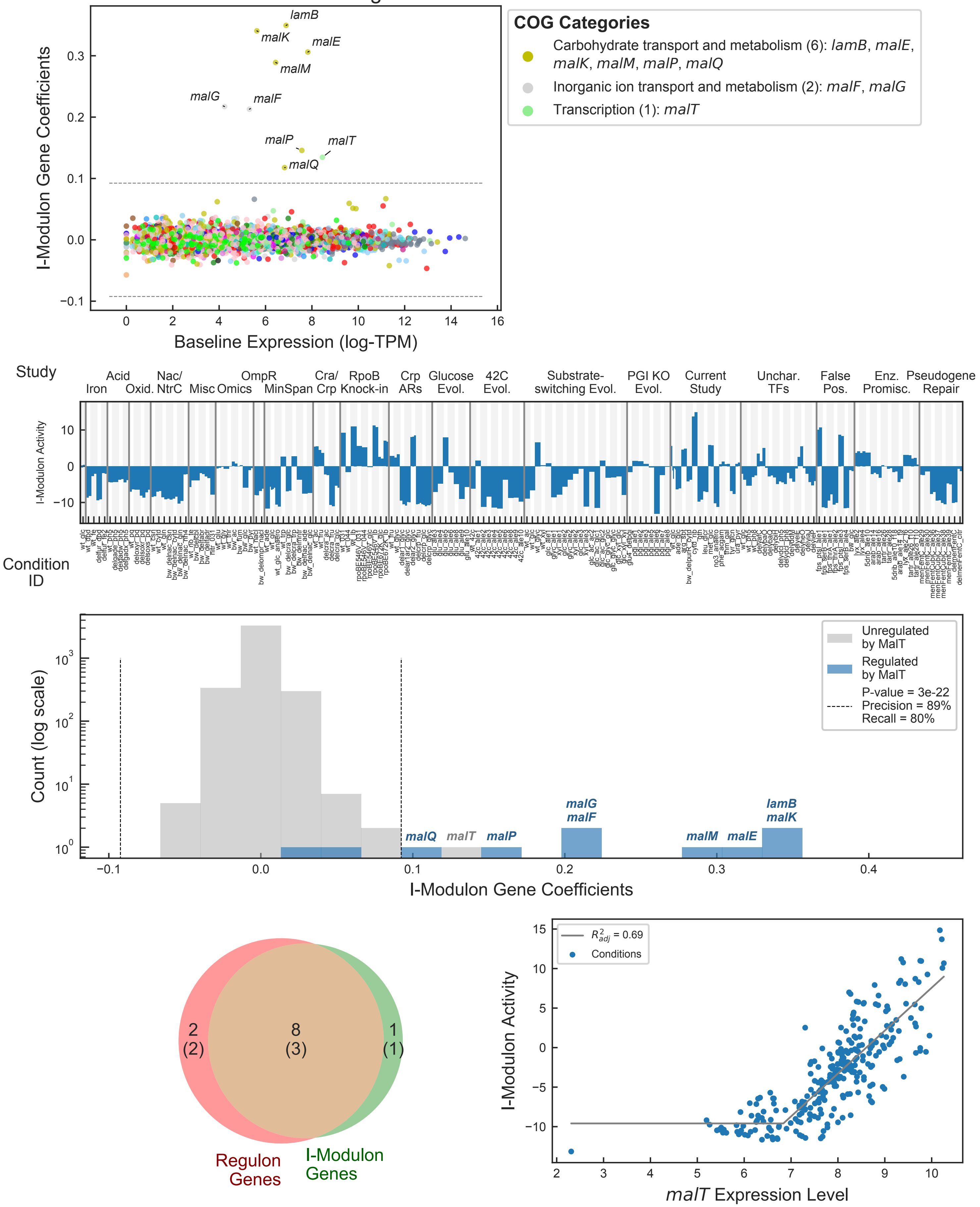

MetJ I-Modulon

Regulated by: MetJ

Biological Function: Methionine biosynthesis

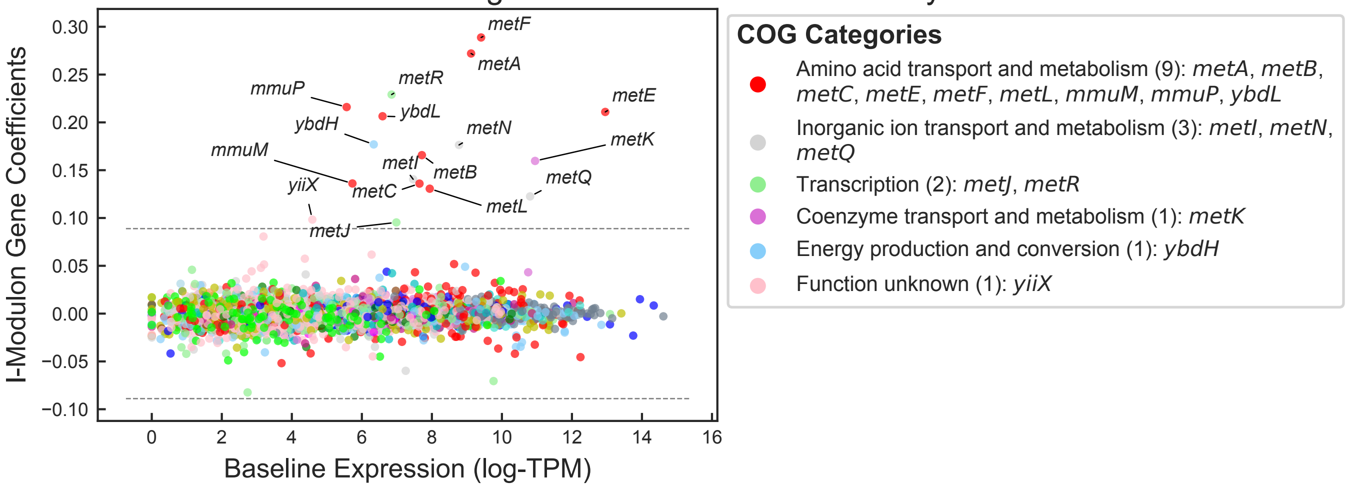

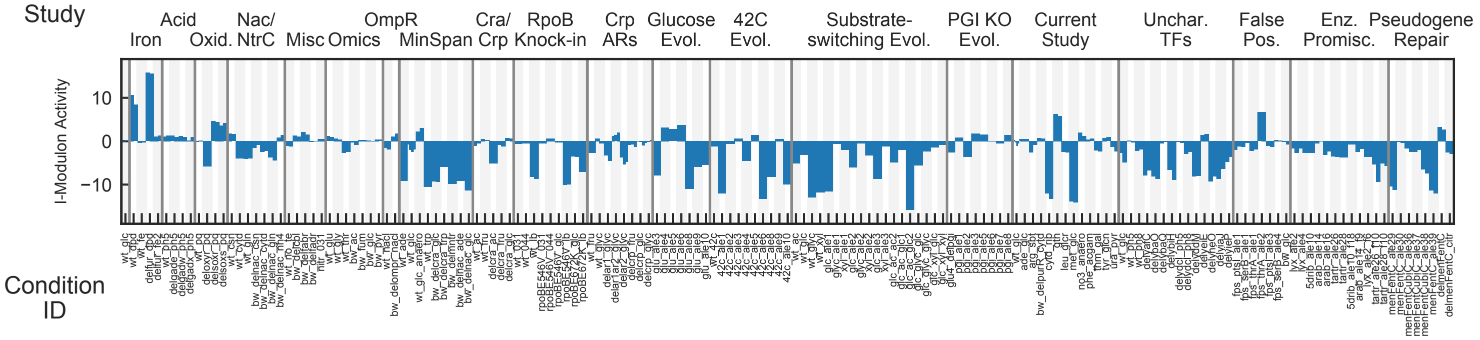

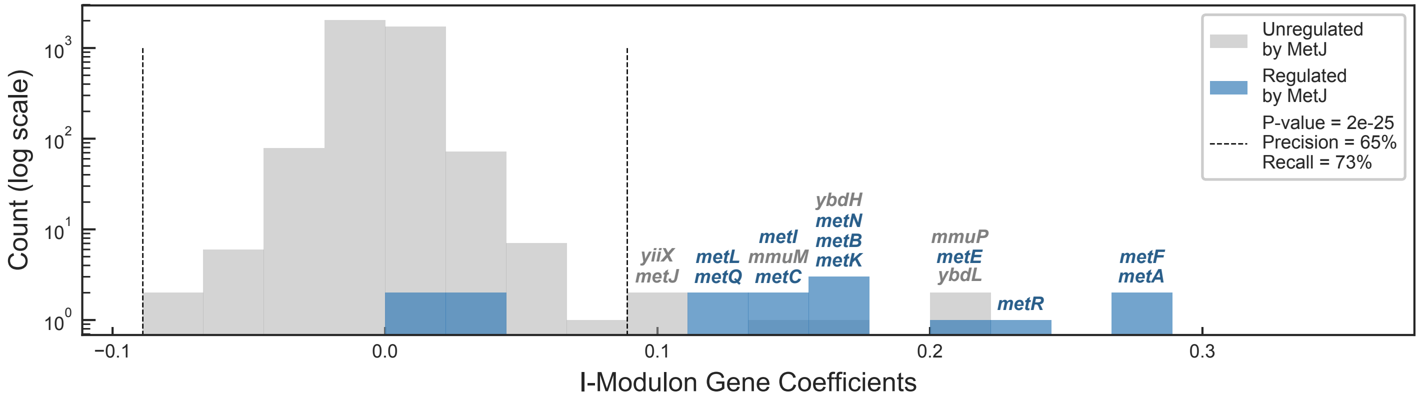

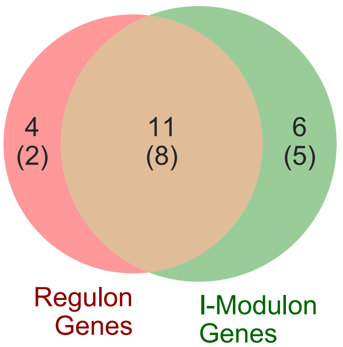

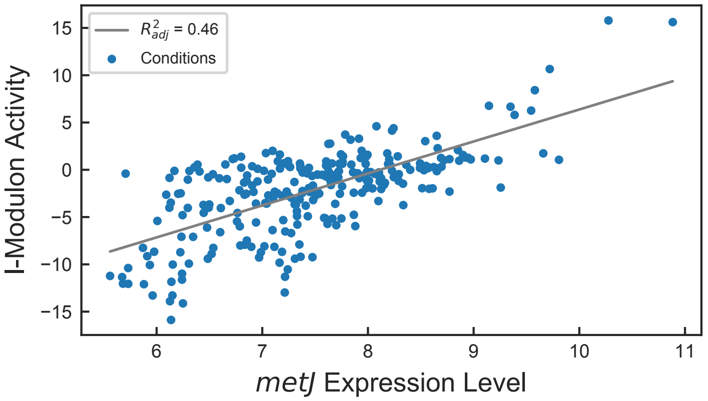

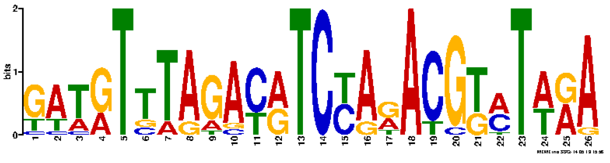

Motif E-value: 1.60e-37

Operons with Upstream Motif: 92%

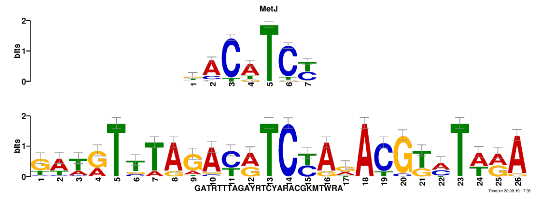

Motif similar to MetJ (E-value: 3.99e-03)

Nac I-Modulon

Regulated by: Nac

Biological Function: Nitrogen starvation response

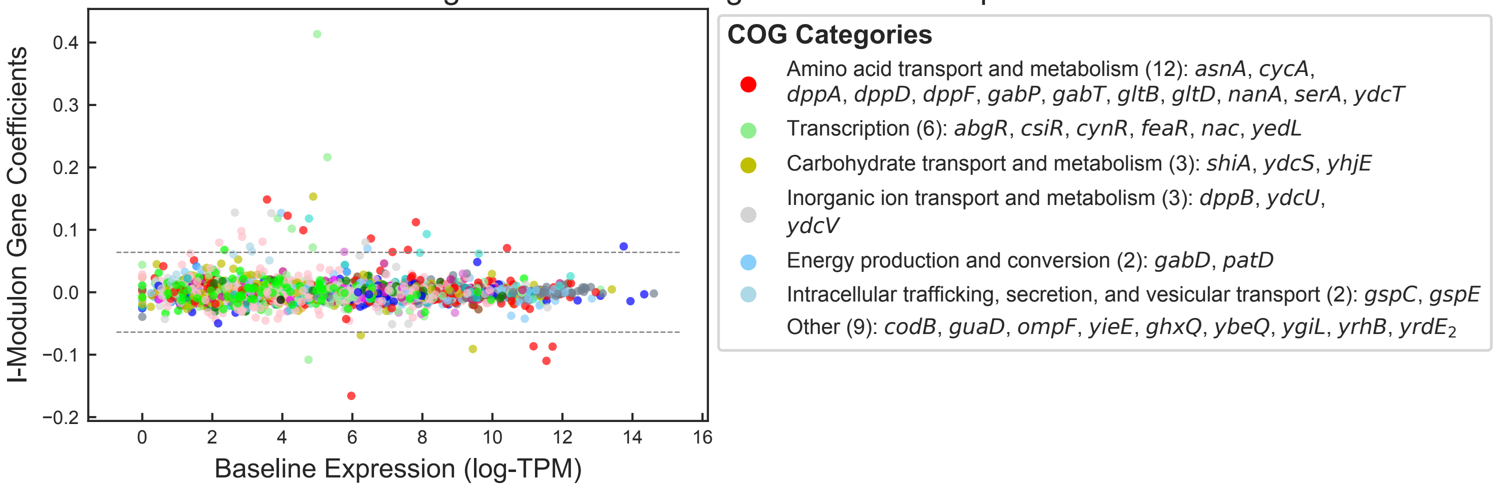

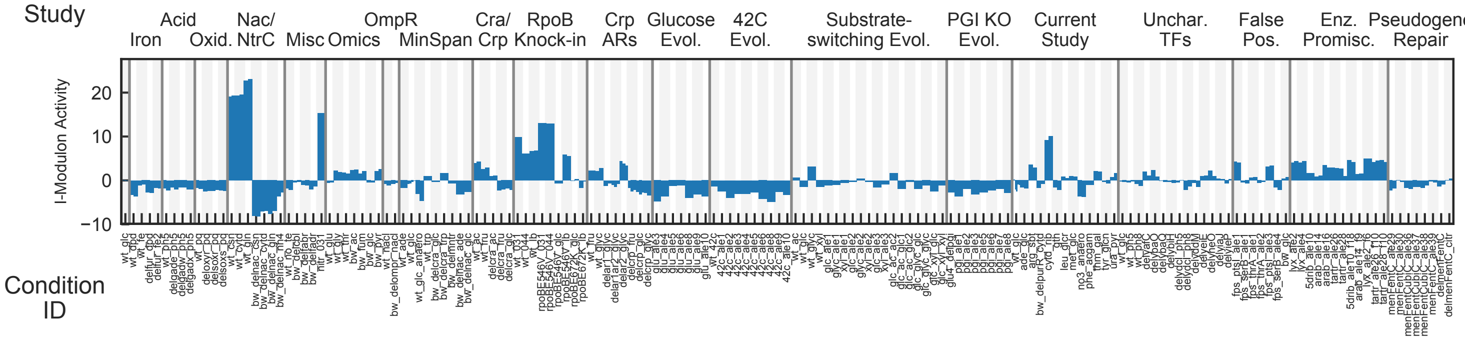

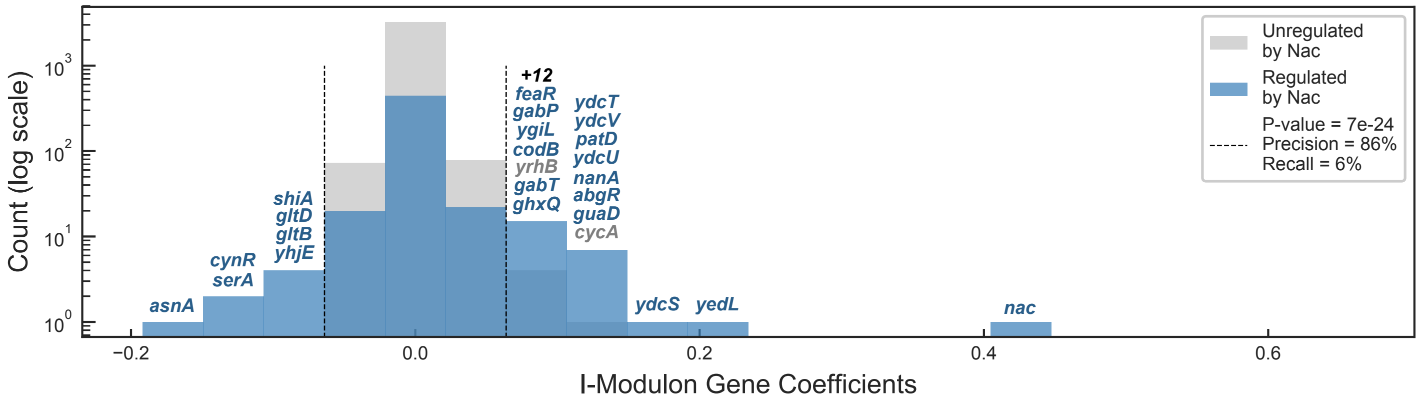

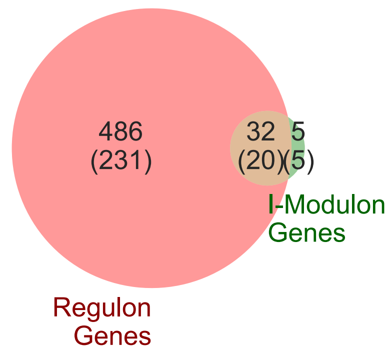

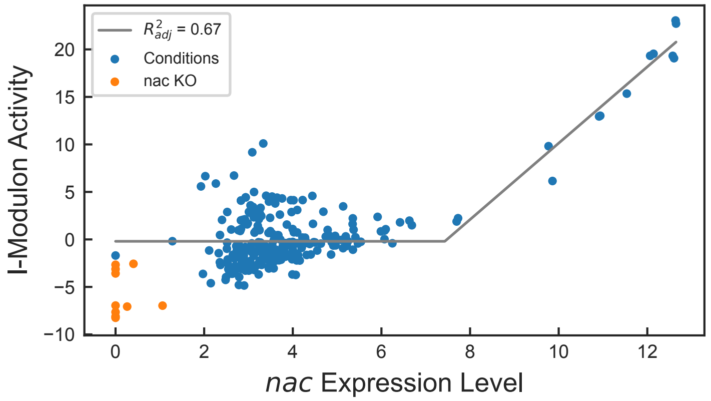

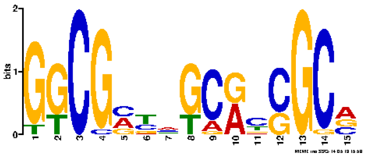

Motif E-value: 2.20e-08

Operons with Upstream Motif: 72%

NagC/TyrR I-Modulon

Regulated by: NagC or TyrR

Biological Function: N-acetylglucosamine catabolism and tyrosine biosynthesis

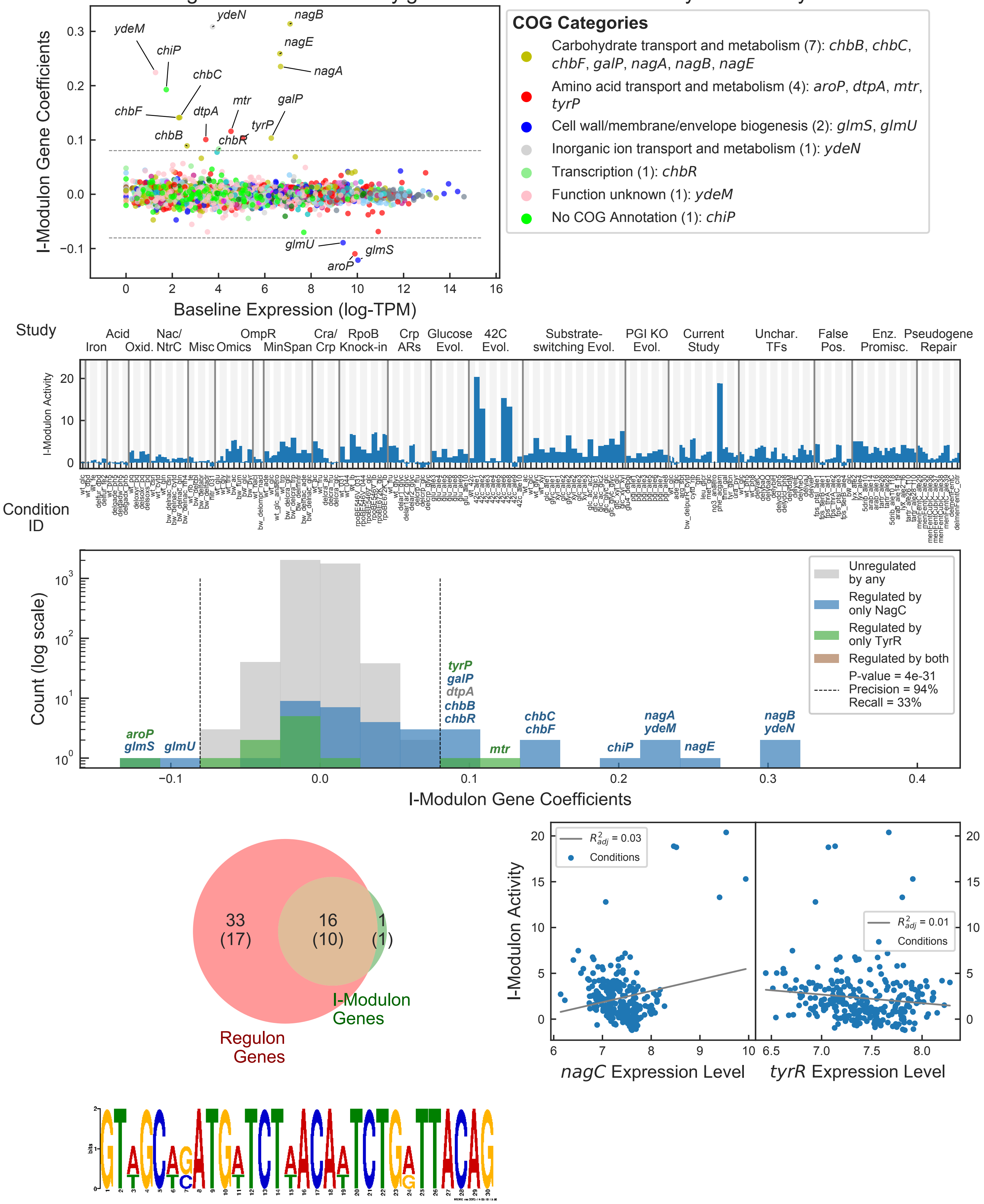

Motif E-value: 1.50e-04

Operons with Upstream Motif: 27%

NarL I-Modulon

Regulated by: NarL

Biological Function: Nitrate respiration

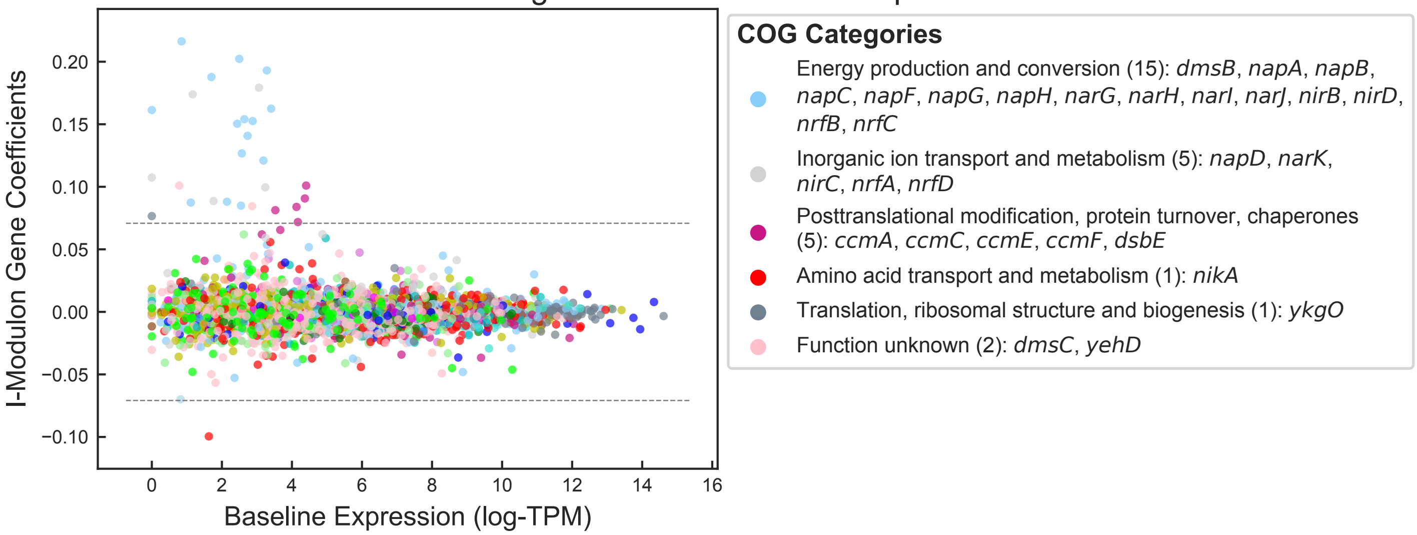

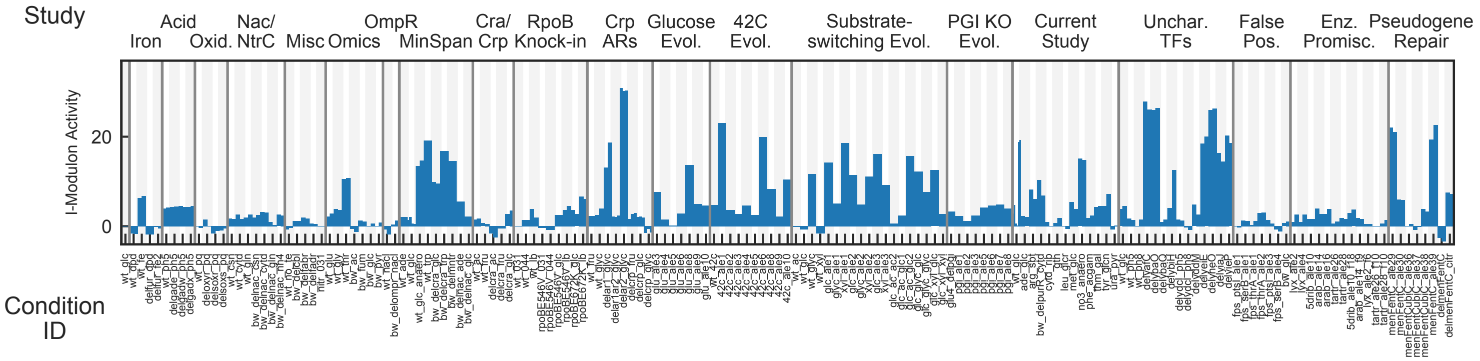

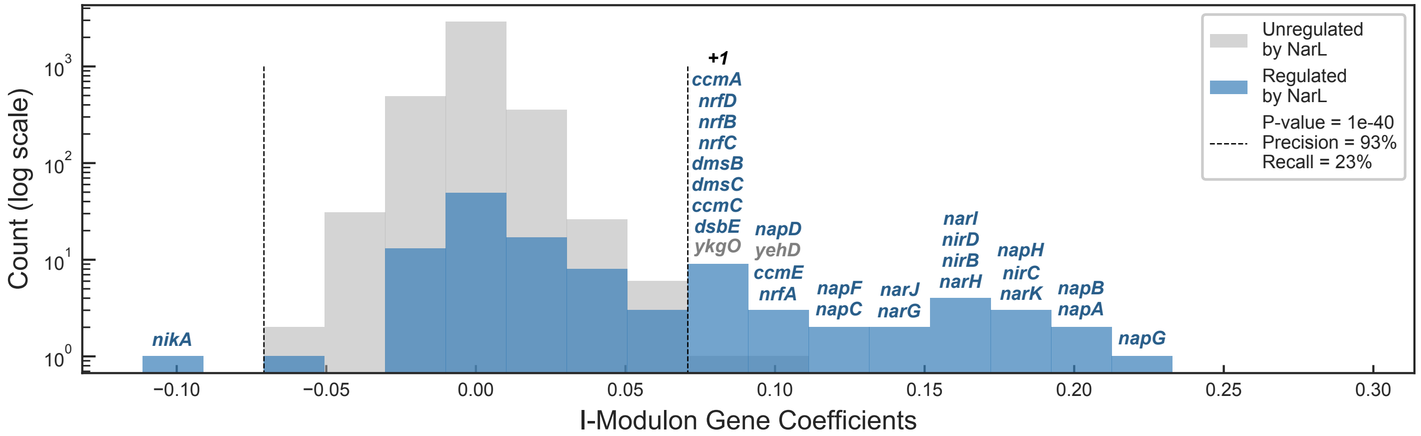

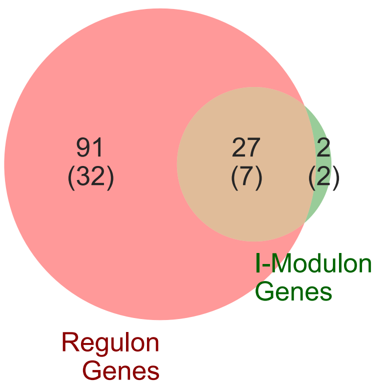

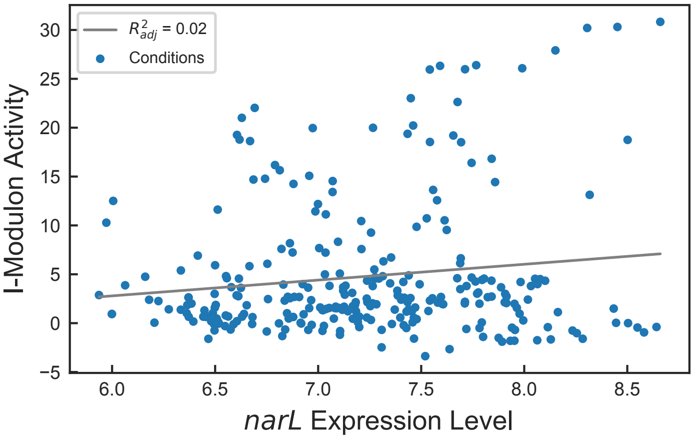

NikR I-Modulon

Regulated by: NikR

Biological Function: Nickel homeostasis

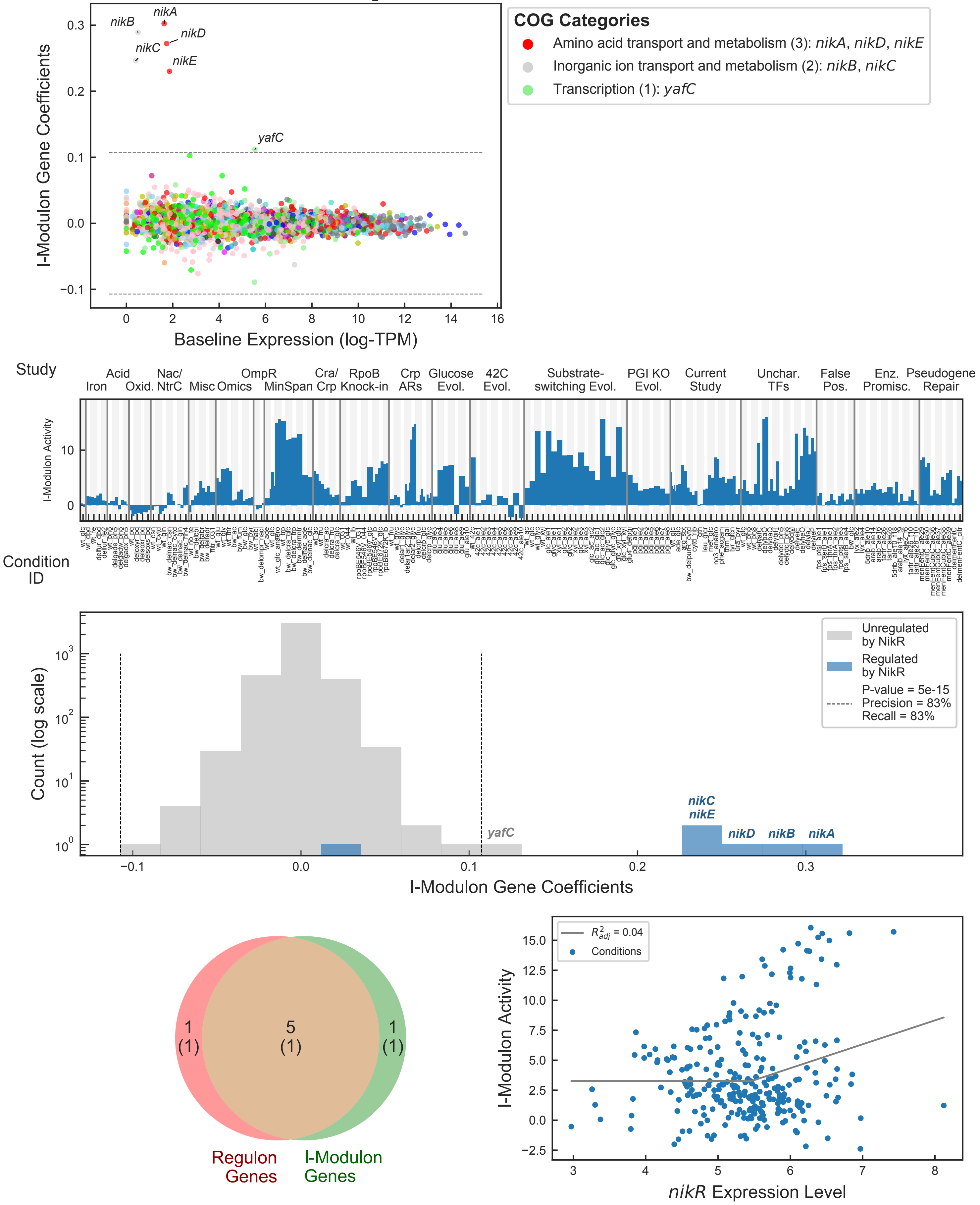

NtrC + RpoN I-Modulon

Regulated by: NtrC and RpoN

Biological Function: Nitrogen starvation response

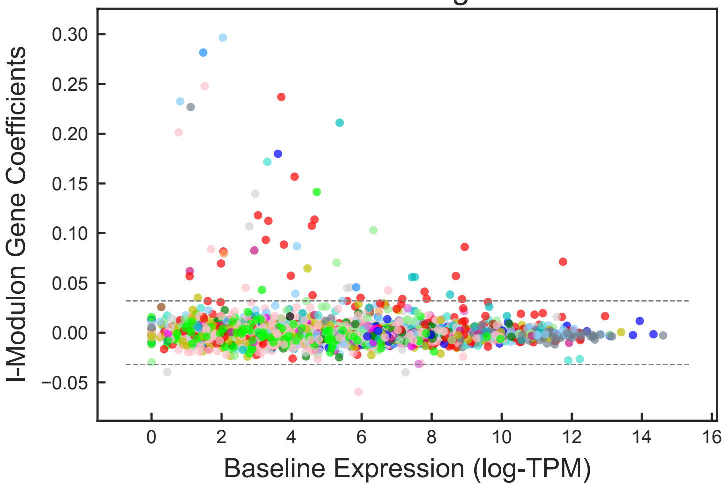

COG Categories

- Amino acid transport and metabolism (21): *argT*, *asnA*, *asnB*, *astA*, *astB*, *astC*, *astE*, *ddpA*, *ddpD*, *ddpF*, *gabP*, *glnA*, *glnK*, *gltI*, *hisP*, *patA*, *potF*, *ygeW*, *yhdX*, *yhdY*, *yhdZ*
- Inorganic ion transport and metabolism (6): *chaC*, *ddpB*, *ddpC*, *mgtA*, *rcnA*, *yeaR*
- Energy production and conversion (5): *astD*, *fdhF*, *lhgO*, *rutA*, *rutE*
- Signal transduction mechanisms (4): *amtB*, *glnG*, *glnL*, *yeaG*
- Carbohydrate transport and metabolism (2): *prpB*, *ynfM*
- Other (18): *hypA*, *yqeB*, *csiD*, *rutB*, *cbl*, *yedL*, *zraP*, *ddpX*, *rutG*, *rutC*, *mgrB*, *rutD*, *rutF*, *yeaE*, *yeaH*, *yqeC*, *asr*, *yhdW₂*

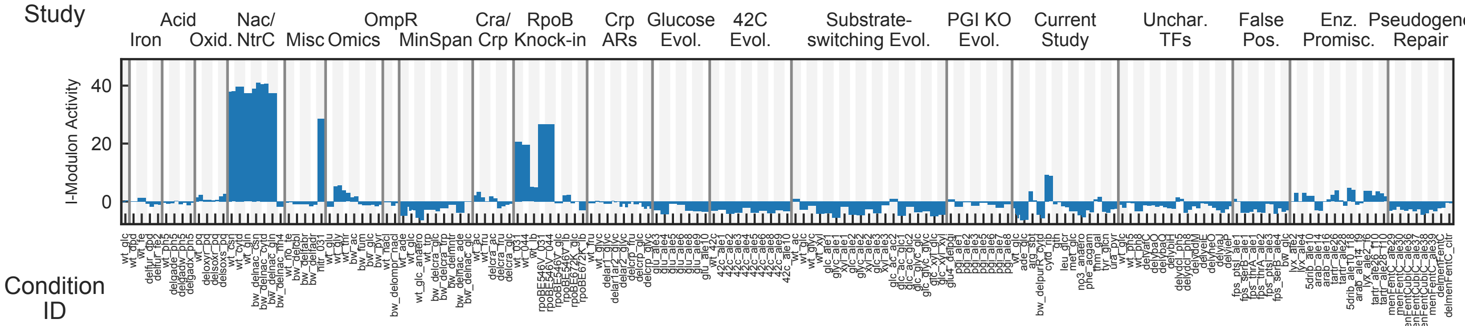

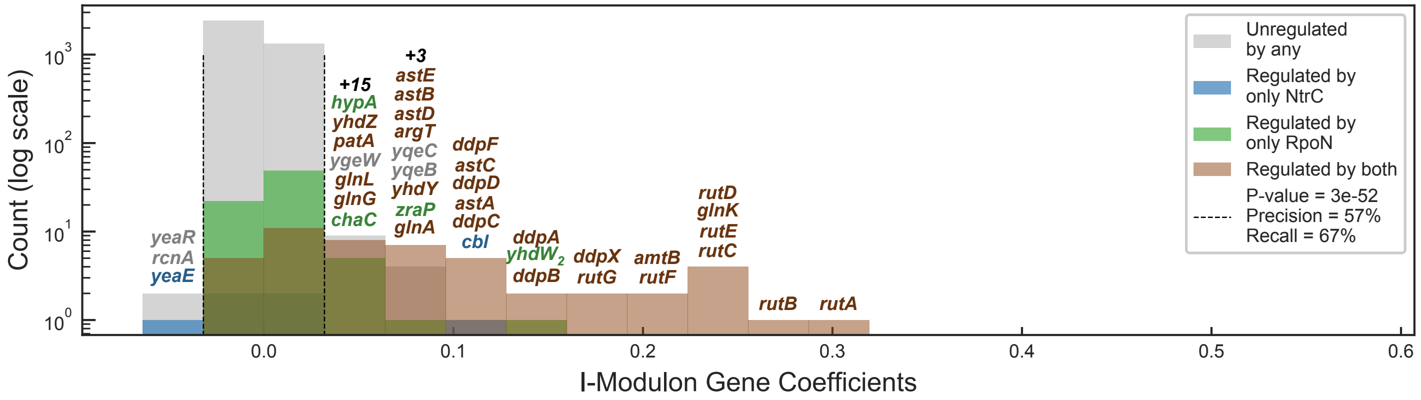

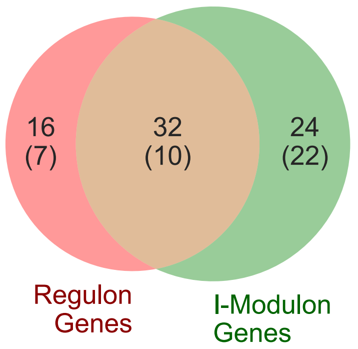

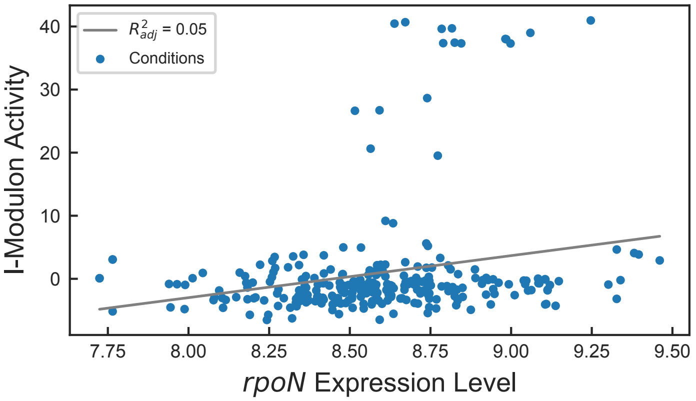

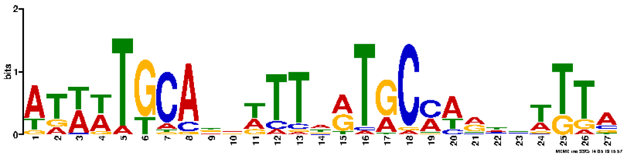

Motif E-value: 1.30e-06

Operons with Upstream Motif: 68%

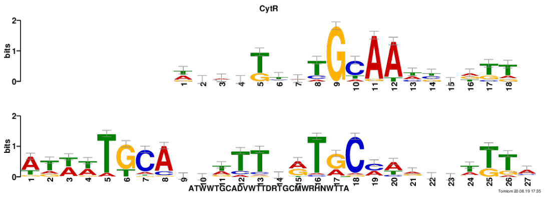

Motif similar to CytR (E-value: 2.29e-03)

OxyR I-Modulon

Regulated by: OxyR

Biological Function: Peroxide reductases

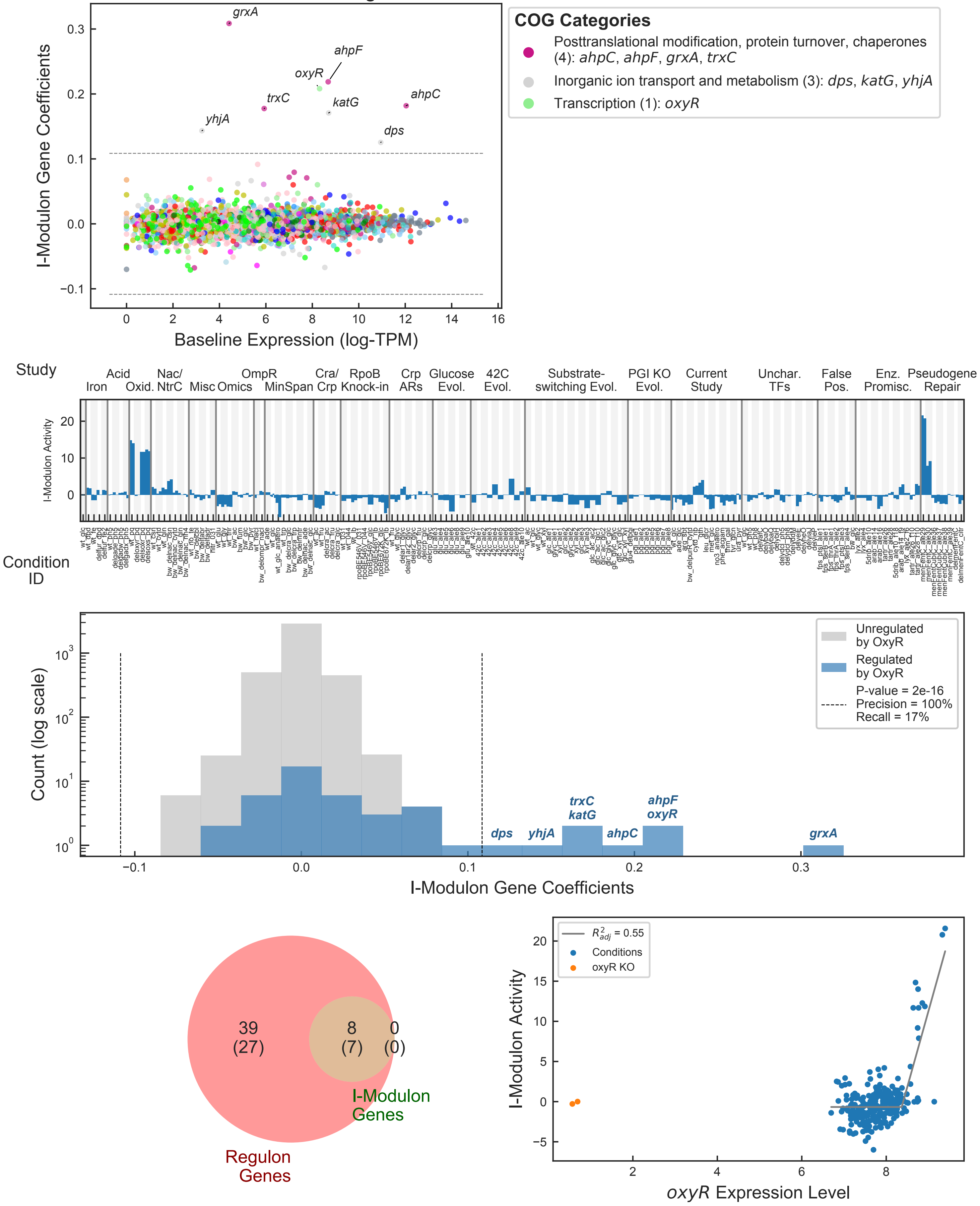

PrpR I-Modulon

Regulated by: PrpR

Biological Function: Propionate catabolism

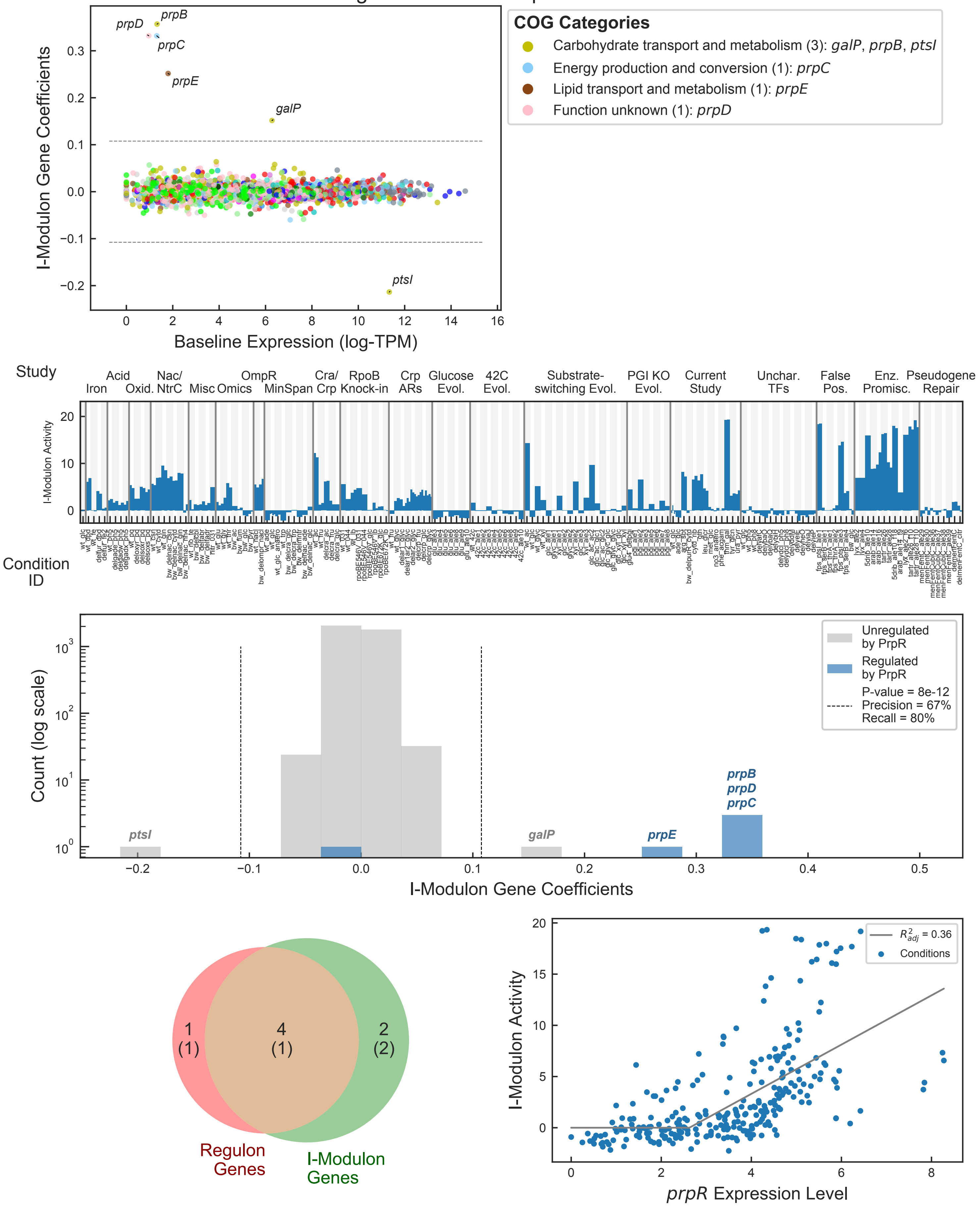

PurR−1 I-Modulon

Regulated by: PurR
Biological Function: Purine Biosynthesis

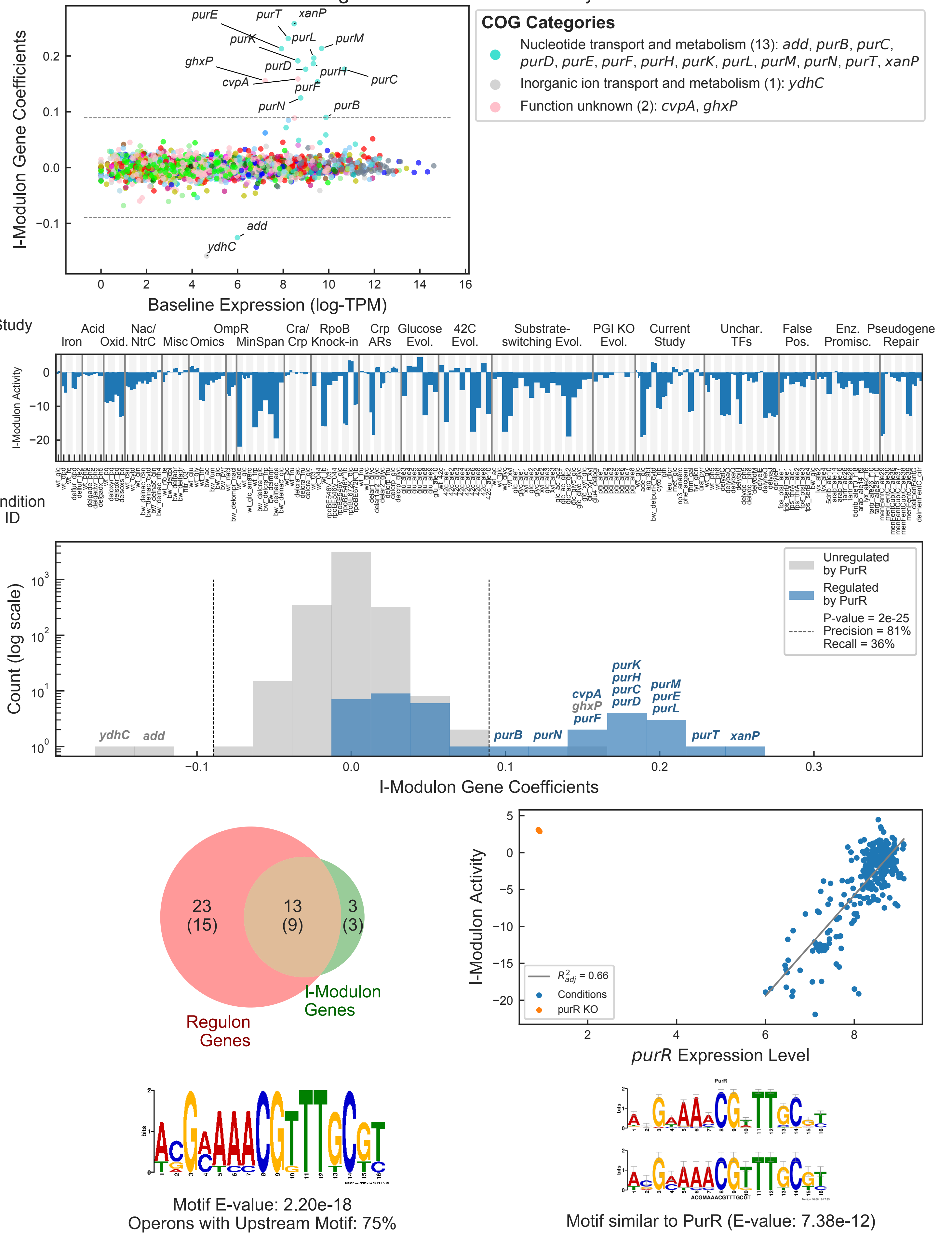

PurR−2 I-Modulon

Regulated by: PurR

Biological Function: Pyrimidine biosynthesis

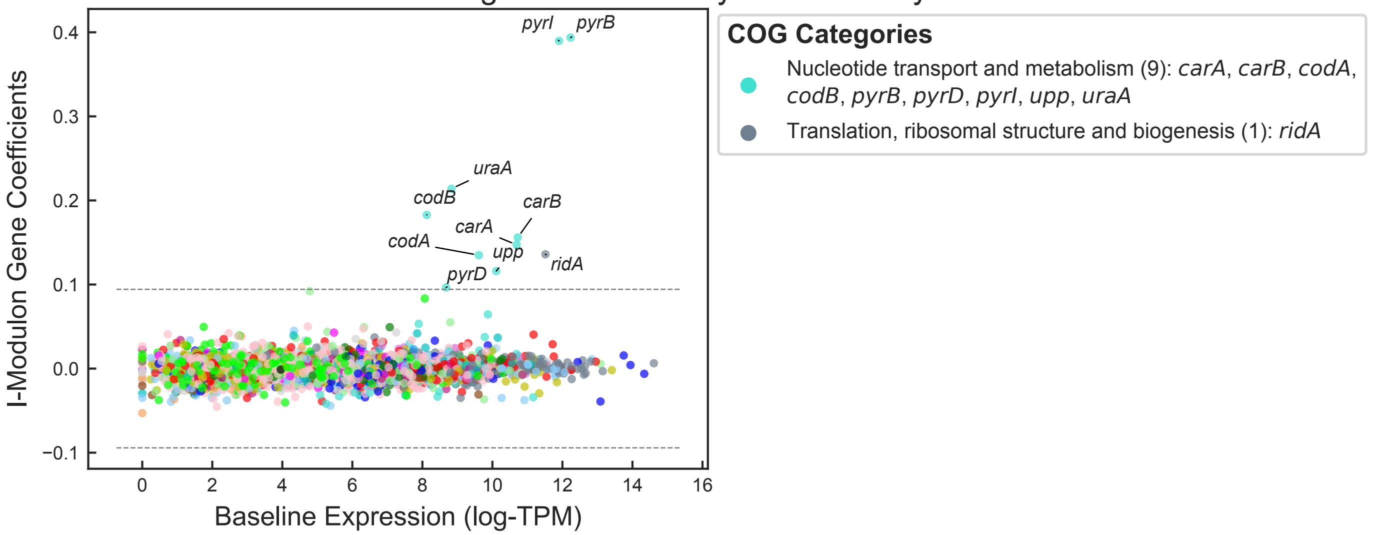

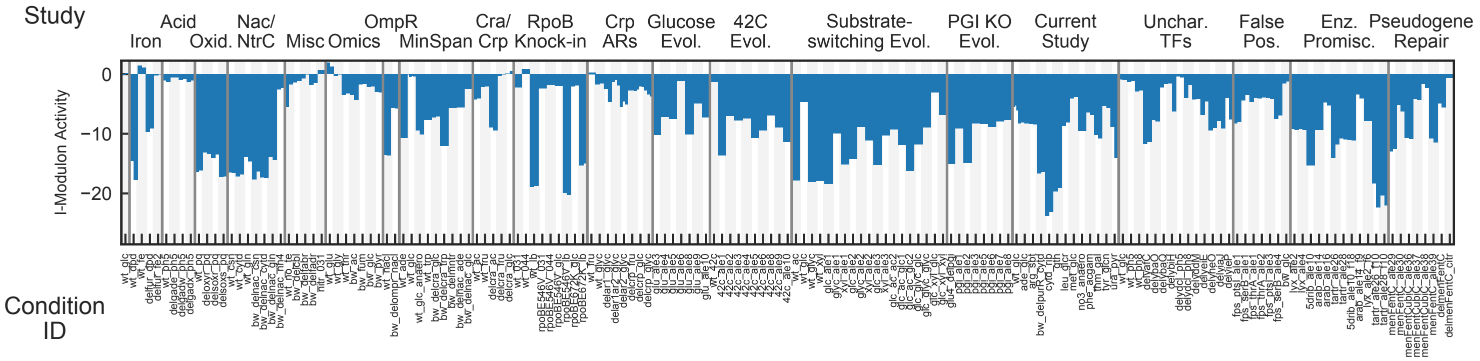

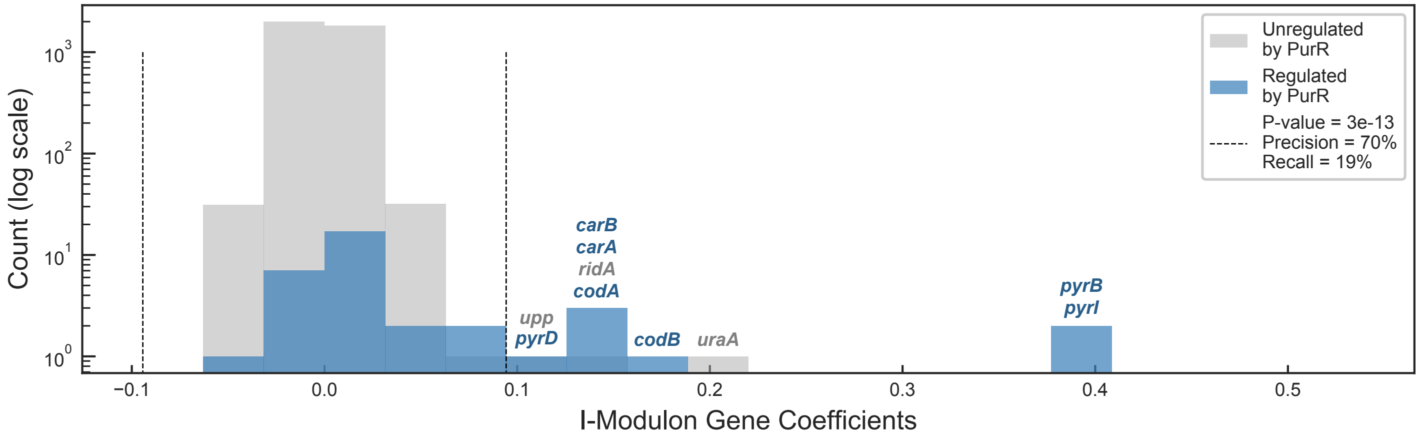

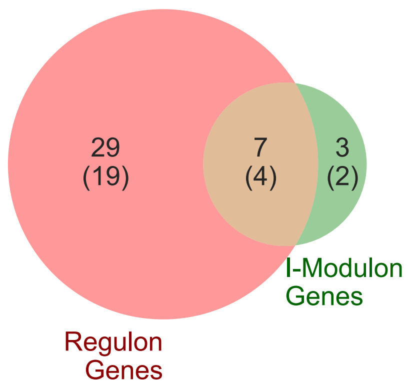

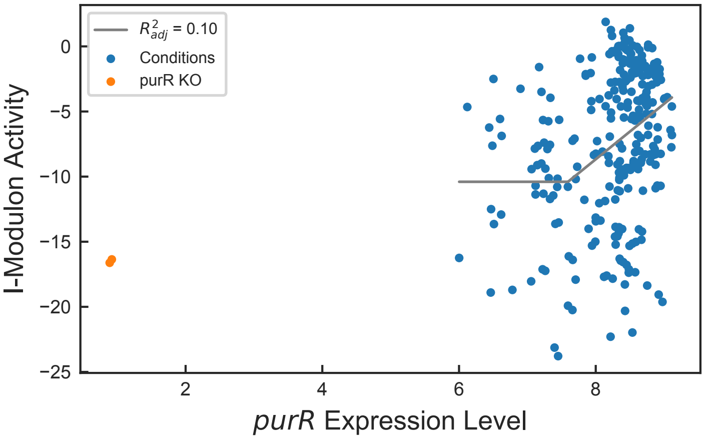

PuuR I-Modulon

Regulated by: PuuR

Biological Function: Putrescine catabolism

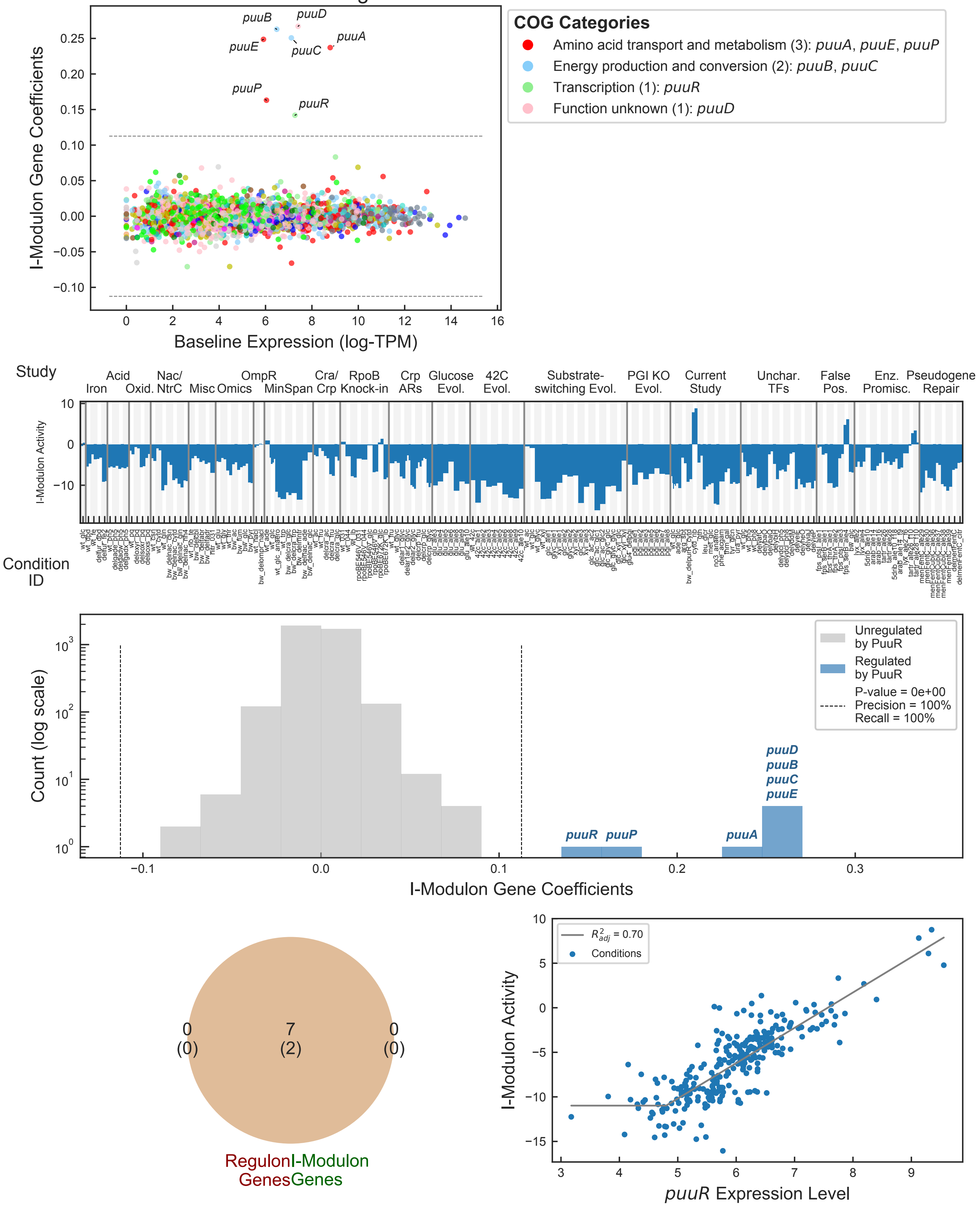

Pyruvate I-Modulon

Regulated by: BtsR or YpdB or PdhR

Biological Function: Pyruvate transport and metabolism

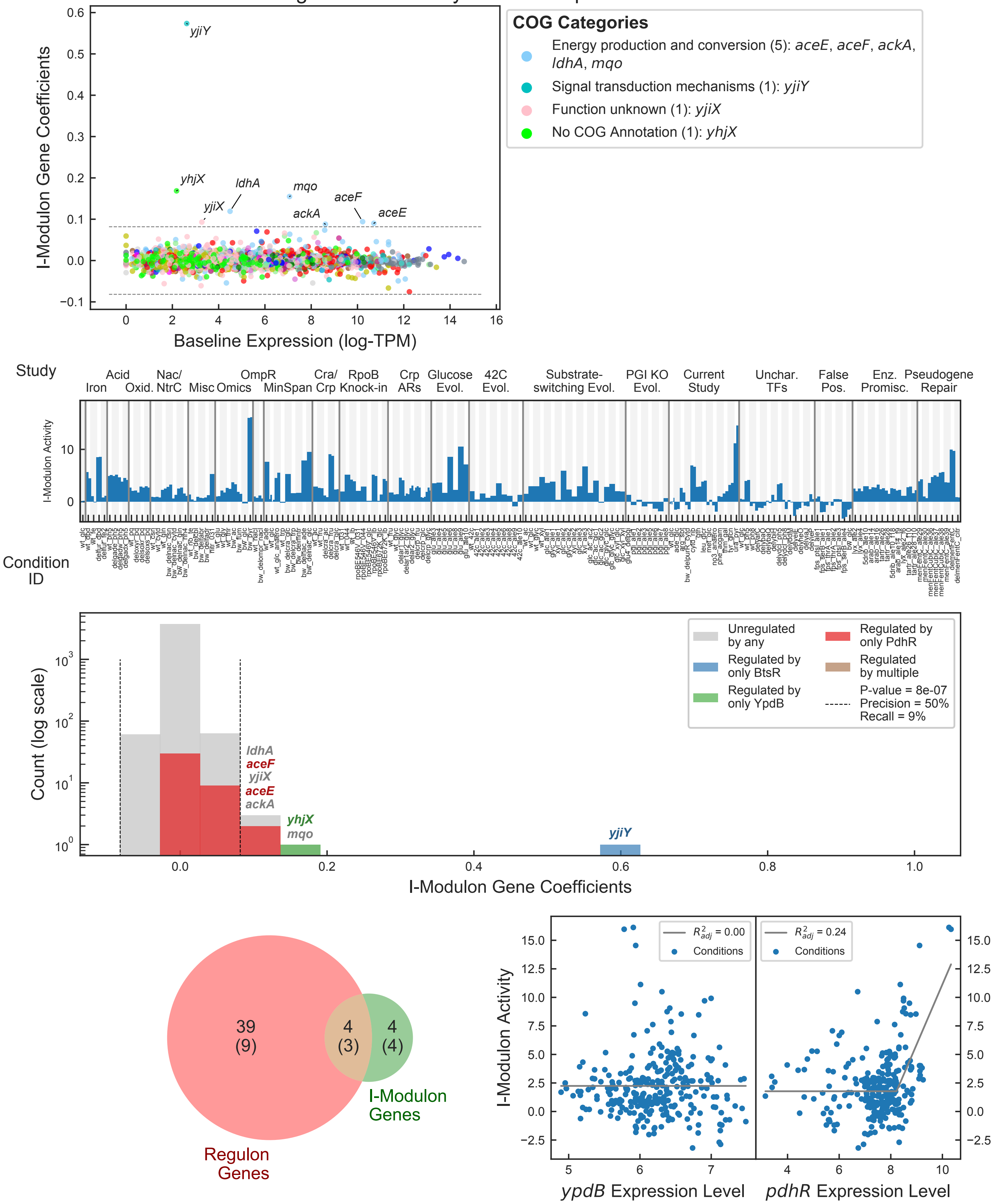

RbsR I-Modulon

Regulated by: RbsR

Biological Function: D-ribose catabolism

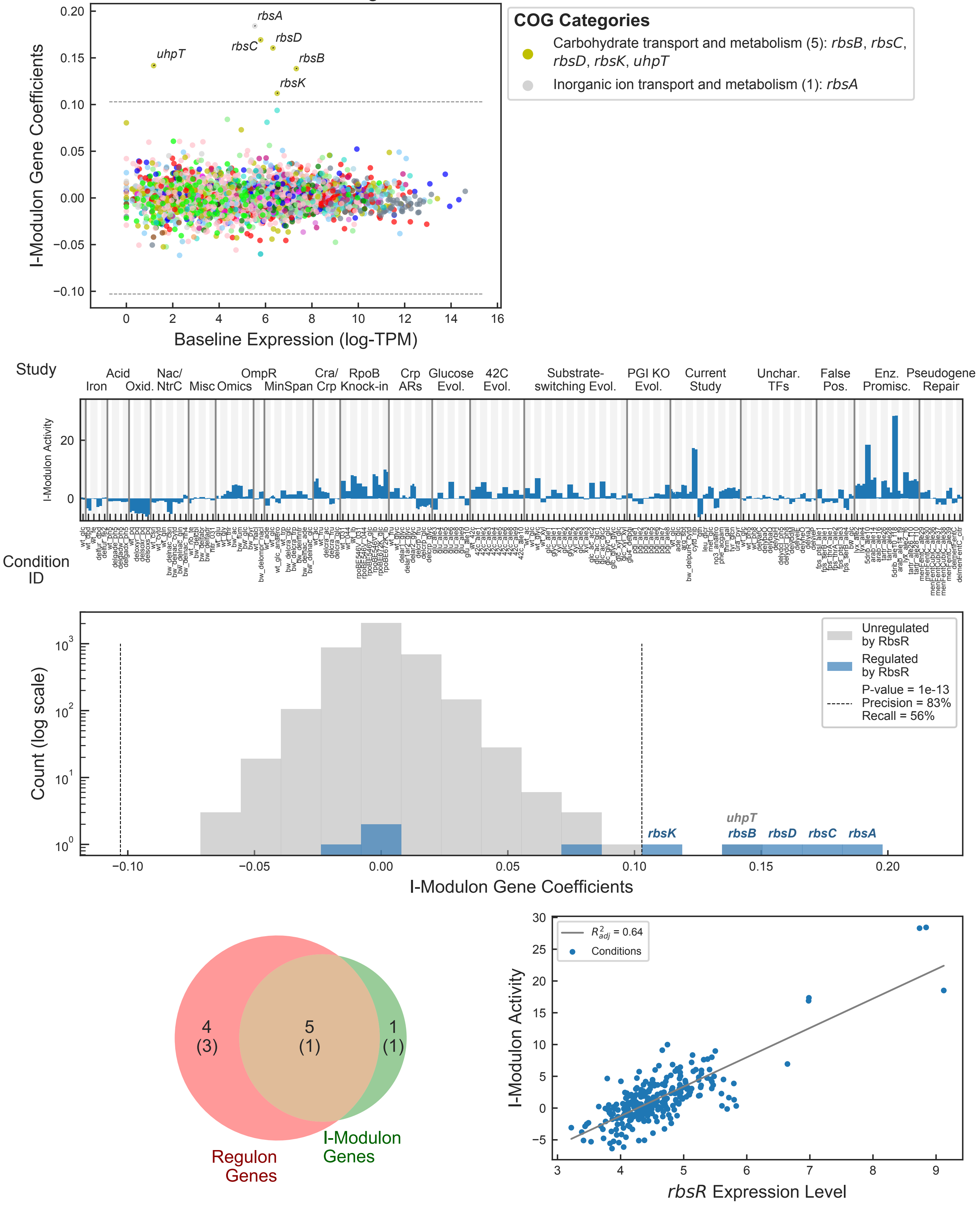

RcsAB I-Modulon

Regulated by: RcsAB

Biological Function: Colanic acid capsule formation

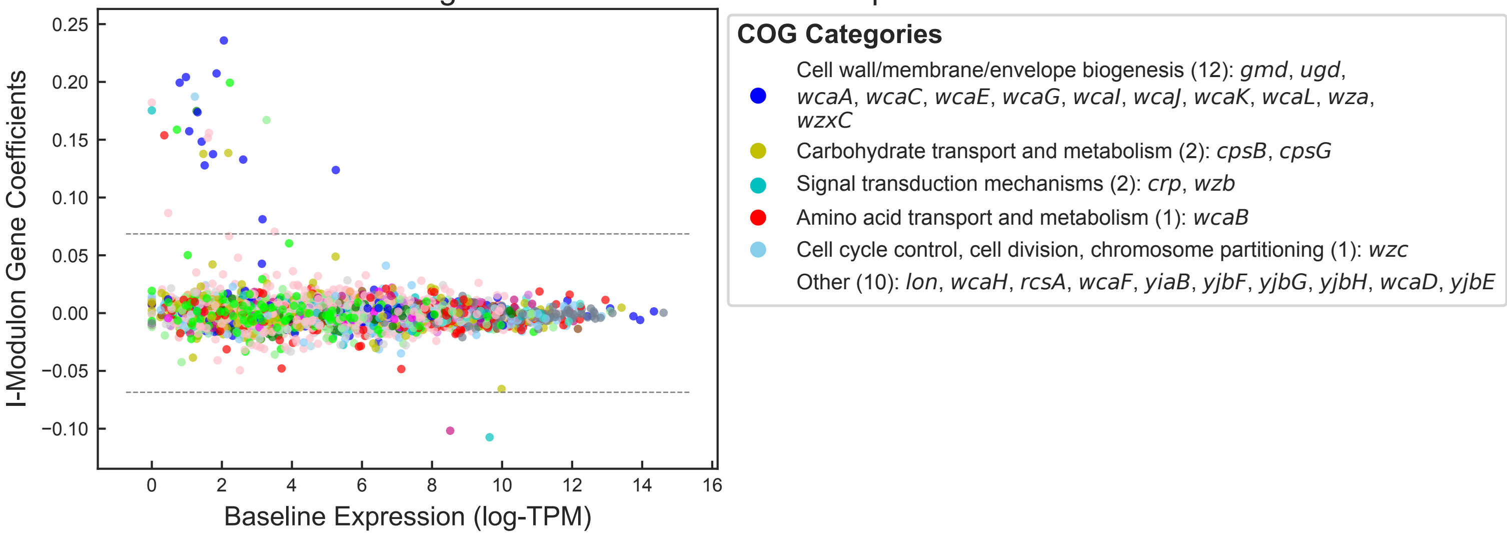

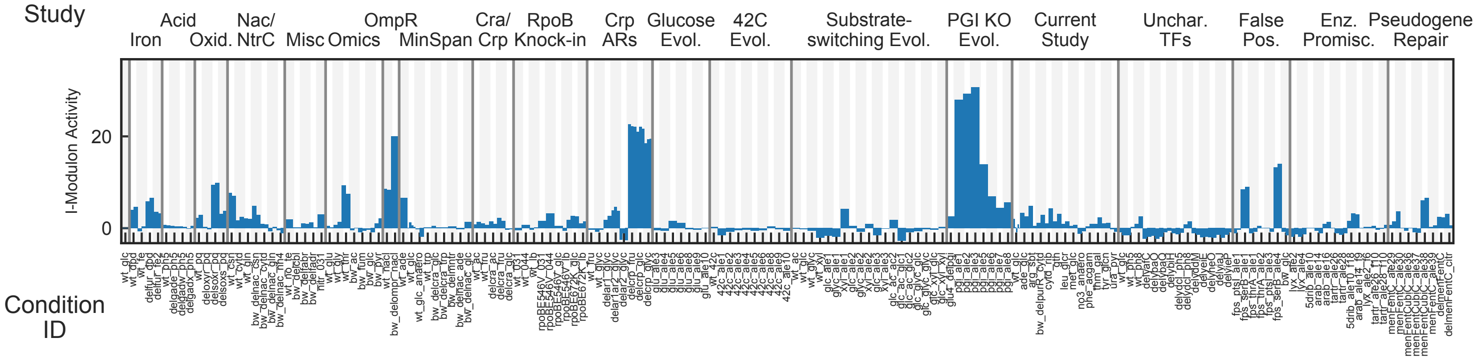

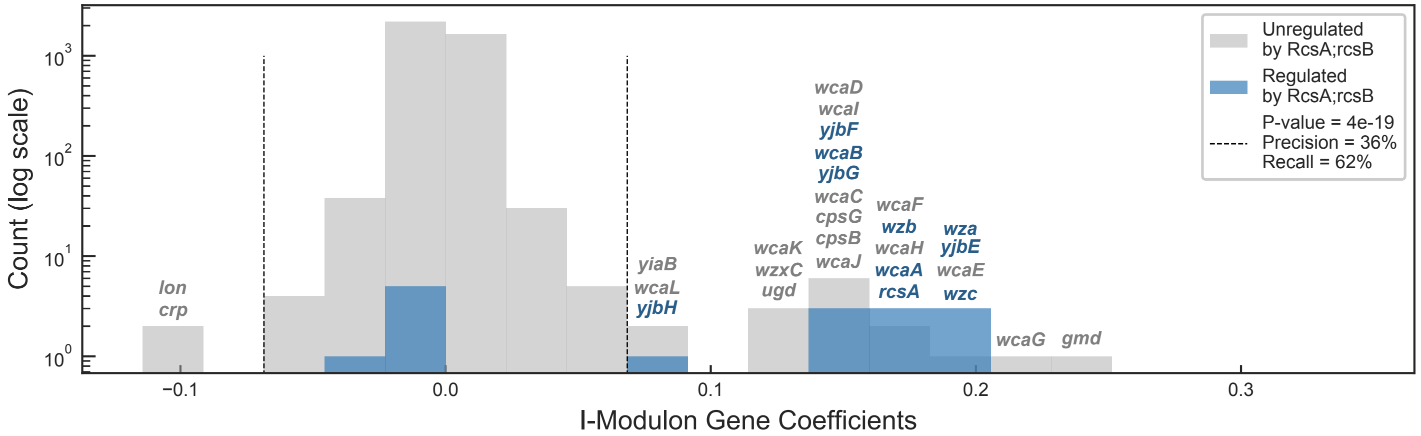

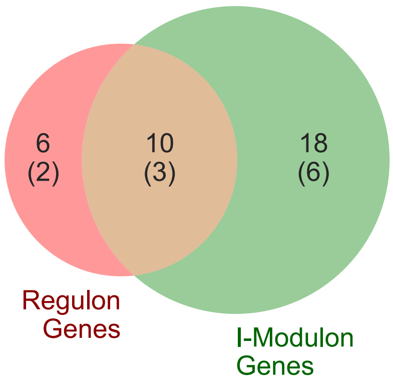

RpoH I-Modulon

Regulated by: RpoH

Biological Function: Heat shock response

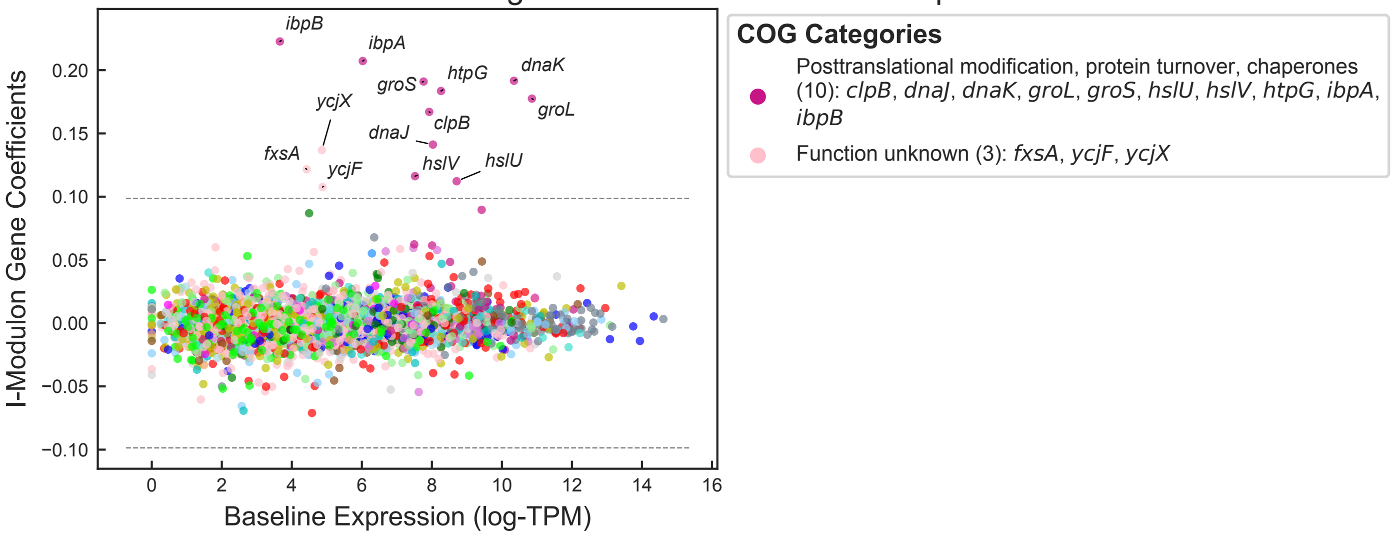

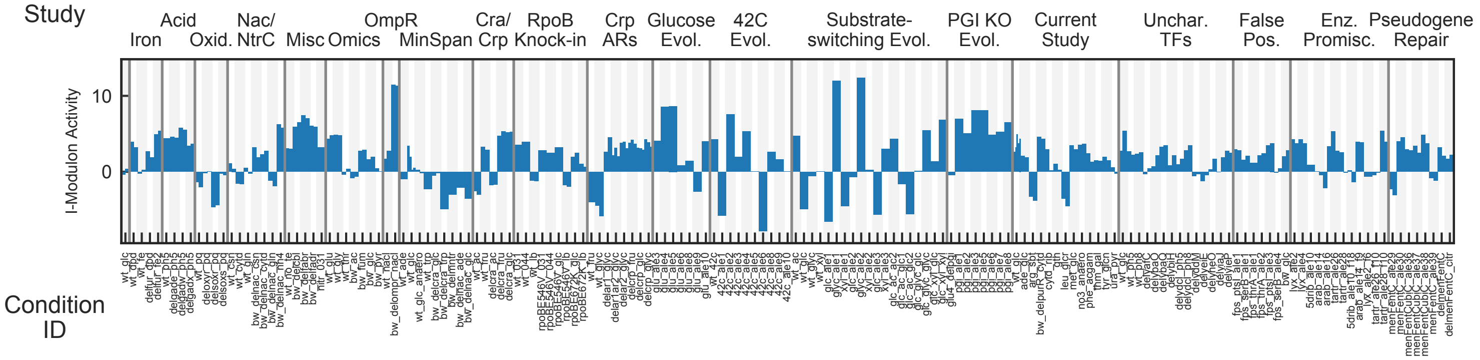

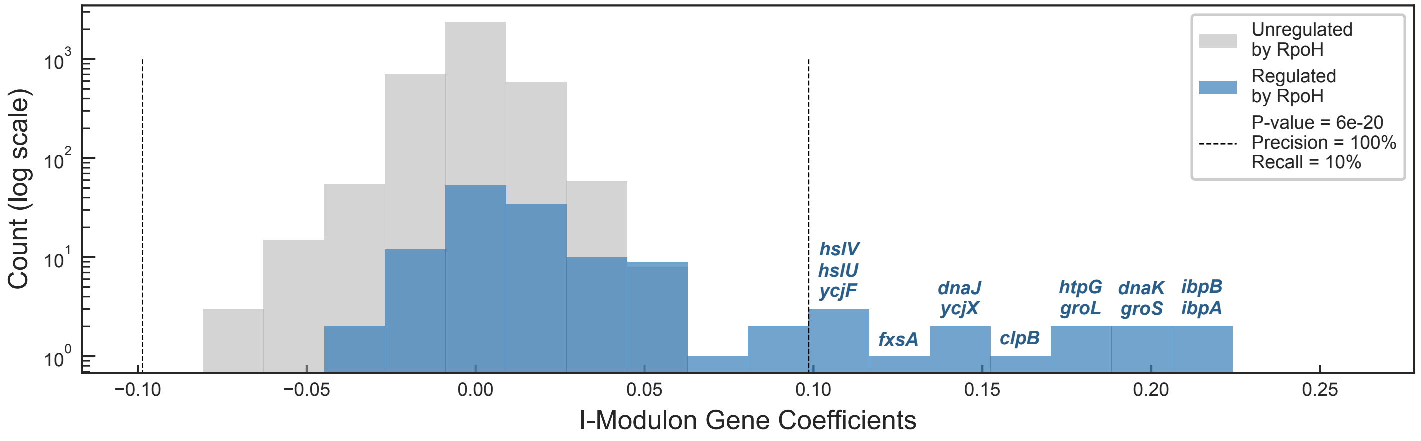

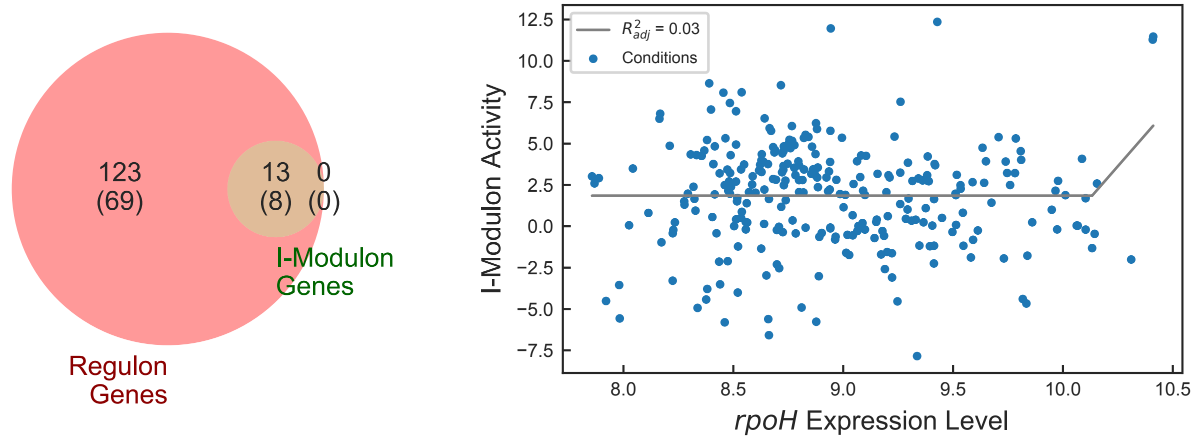

RpoS I-Modulon

Regulated by: RpoS

Biological Function: General stress response

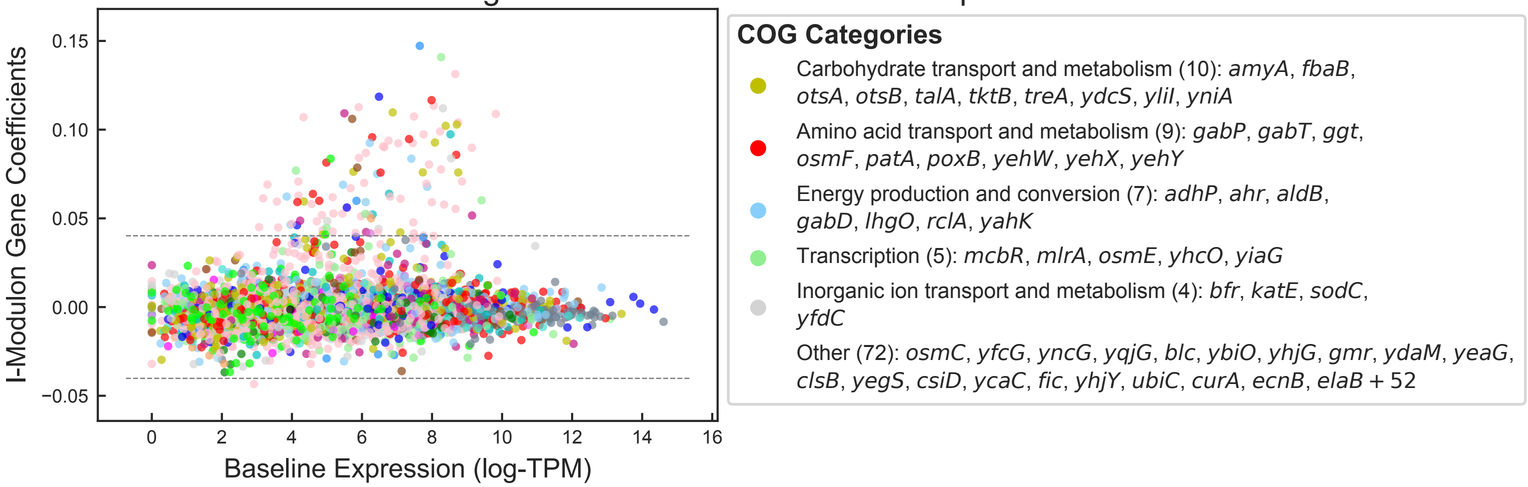

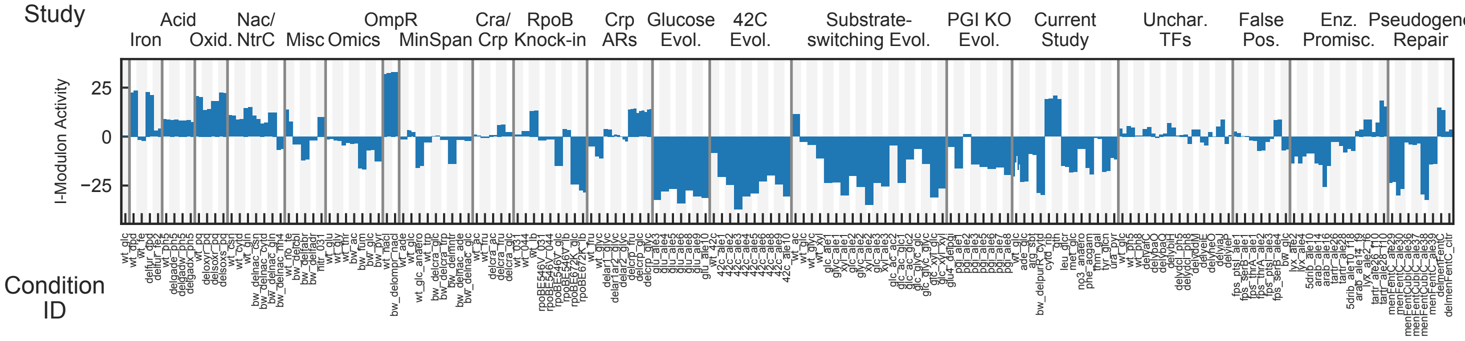

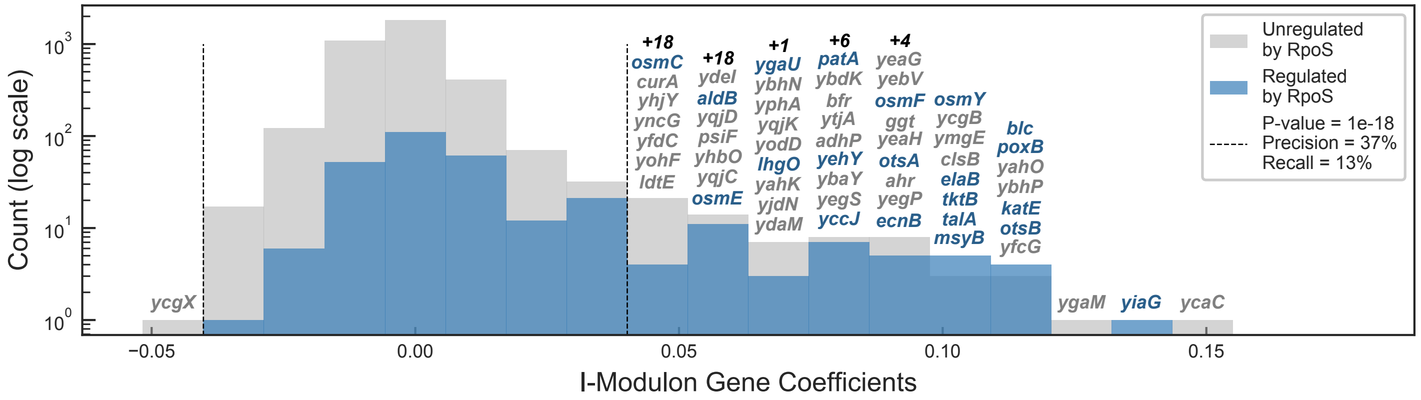

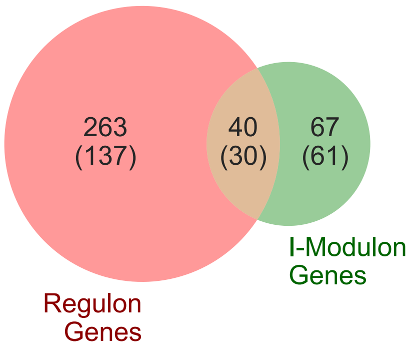

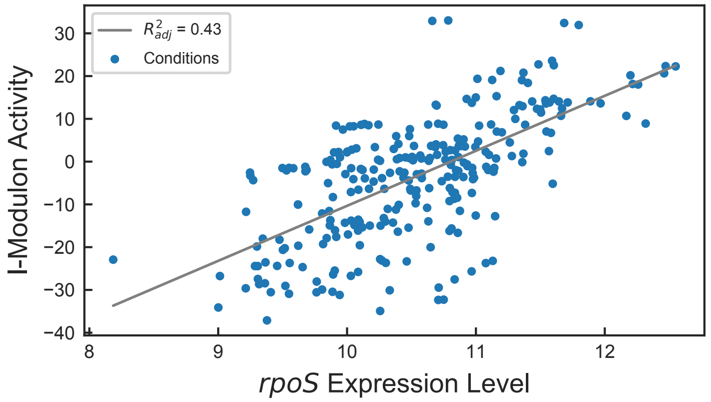

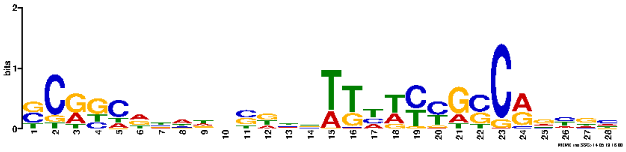

Motif E-value: 3.00e-05

Operons with Upstream Motif: 98%

SoxS I-Modulon

Regulated by: SoxS

Biological Function: Oxidative stress response

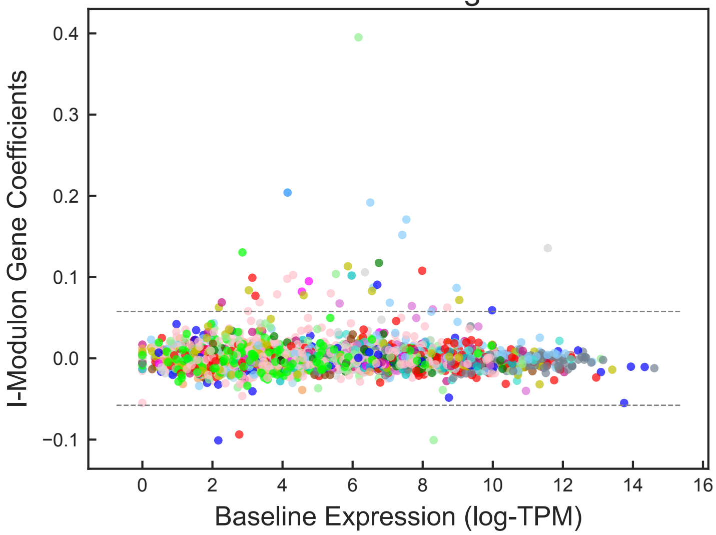

COG Categories

- Carbohydrate transport and metabolism (6): *cpsG*, *mdtG*, *nepI*, *treF*, *yeiI*, *zwf*
- Energy production and conversion (6): *fldA*, *fldB*, *fpr*, *fumC*, *nfsA*, *pfo*
- Amino acid transport and metabolism (4): *aegA*, *frvX*, *poxB*, *rspB*
- Cell wall/membrane/envelope biogenesis (3): *lpxC*, *mltC*, *rspA*
- Other (22): *lipA*, *ribA*, *rimK*, *oxyR*, *soxS*, *ybaO*, *mdlA*, *mdlB*, *sodA*, *yggX*, *yjjW*, *nfo*, *nhoA*, *inaA*, *ariR*, *yaiA*, *ybjC*, *ygfZ*, *yhcN*, *ymgA* + 2

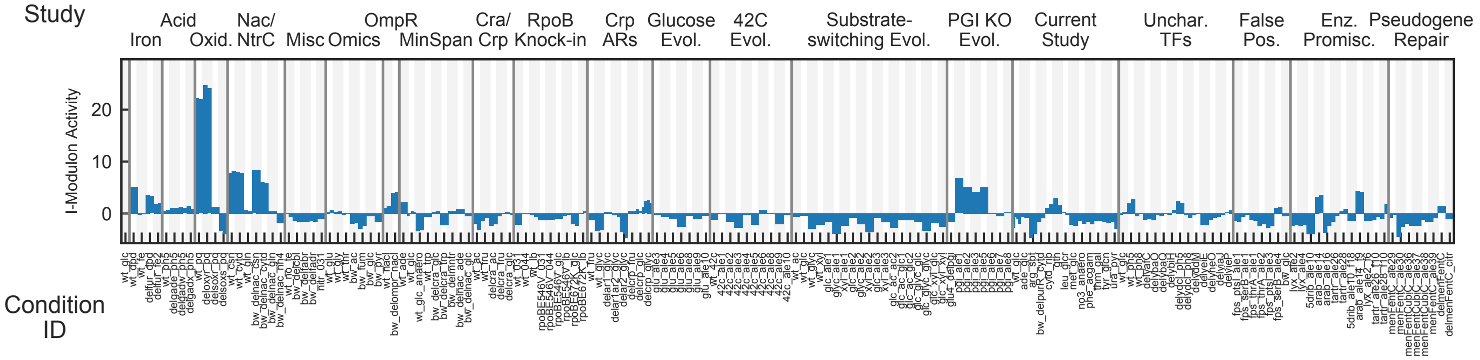

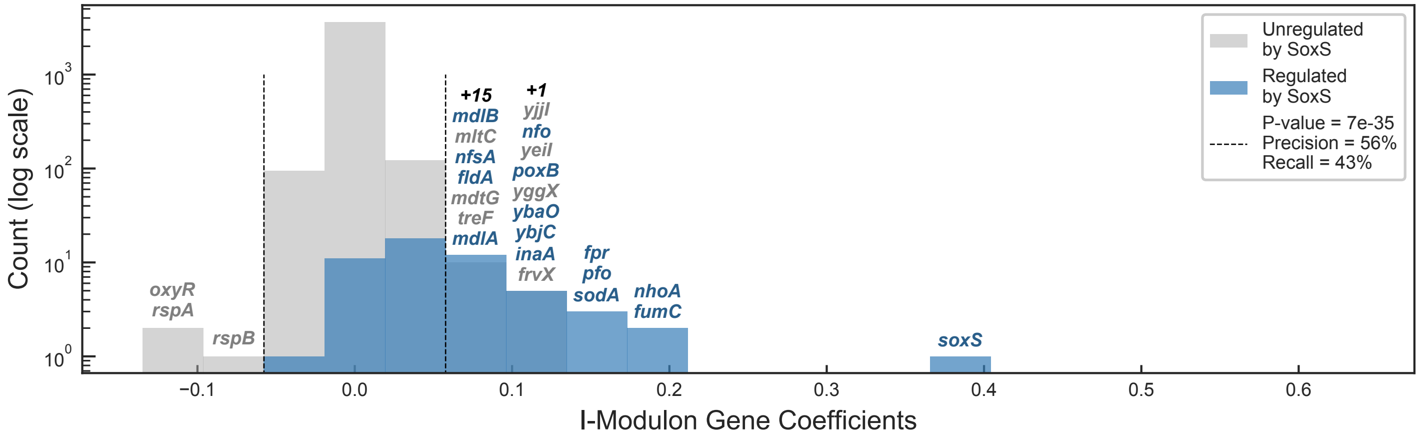

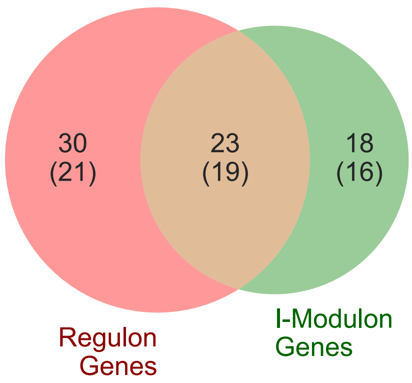

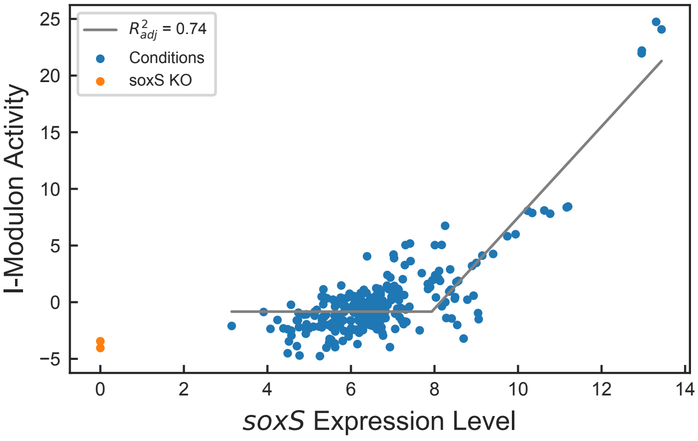

SrlR + GutM I-Modulon

Regulated by: SrlR and GutM

Biological Function: Sorbitol catabolism

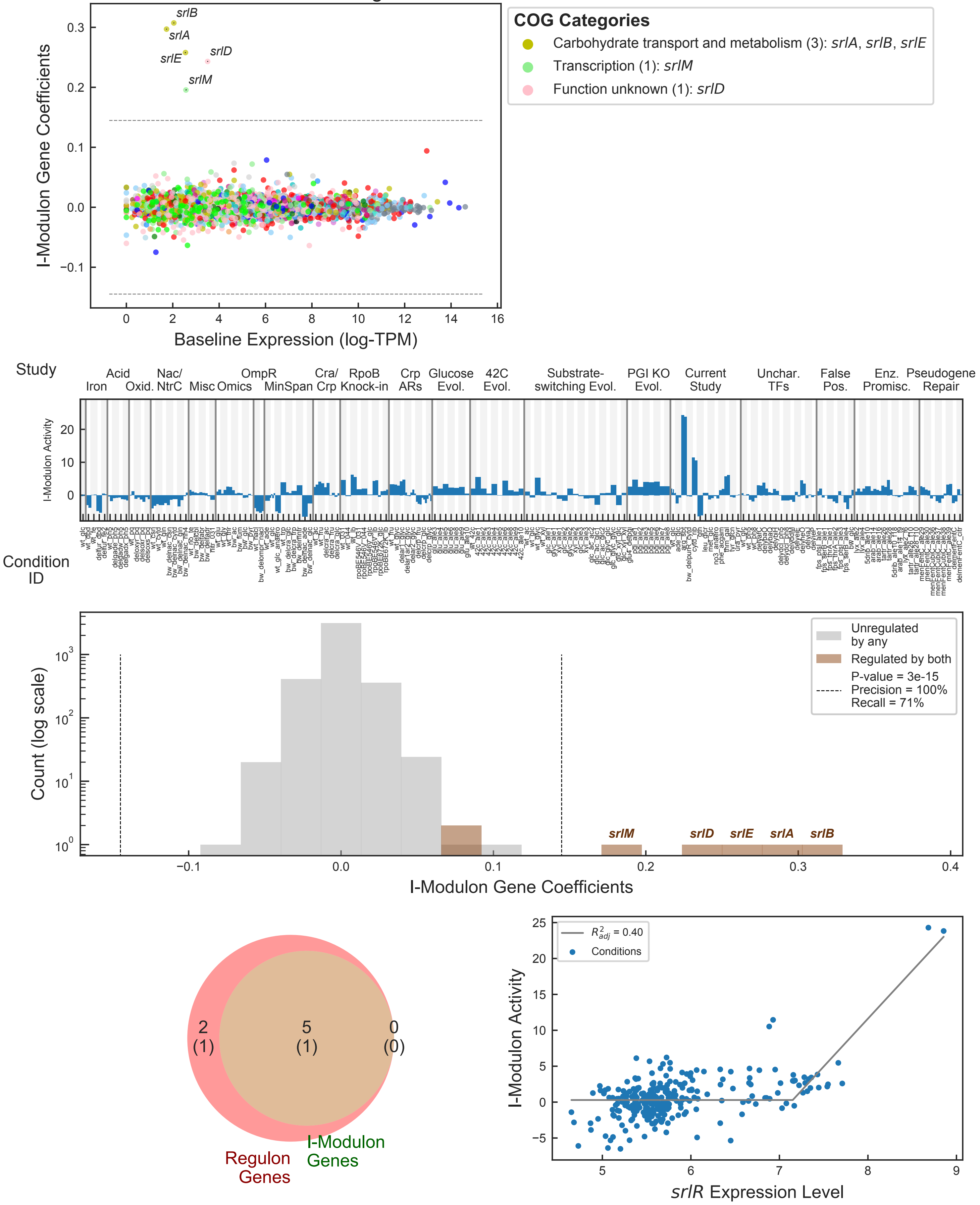

Thiamine I-Modulon

Regulated by: Thiamine

Biological Function: Thiamine biosynthesis

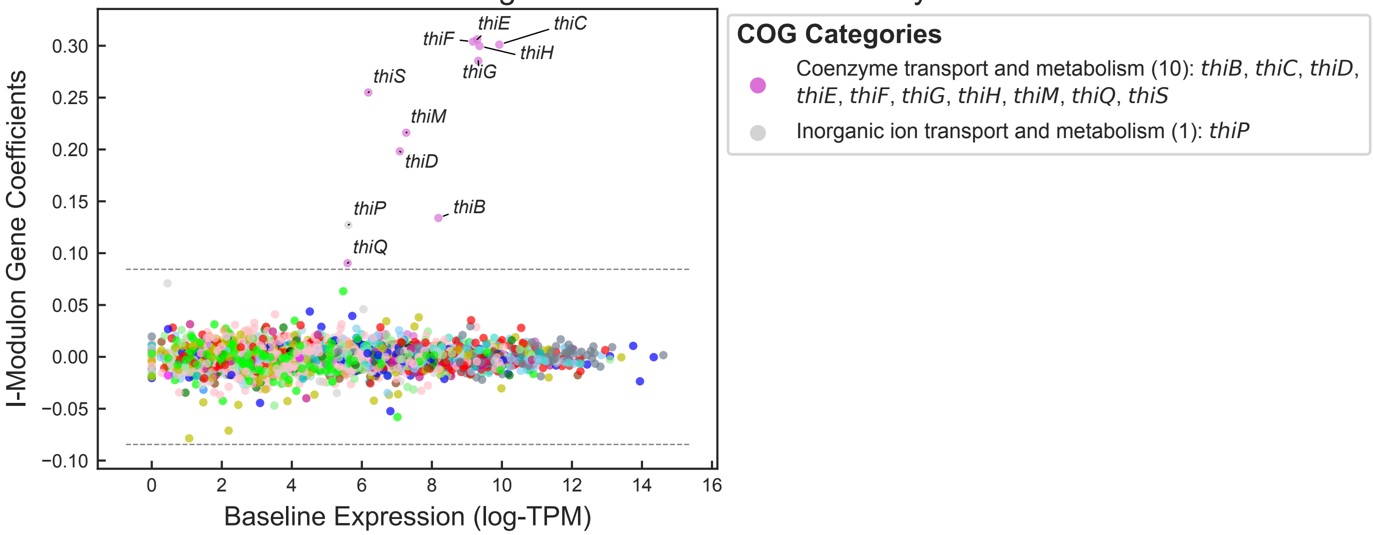

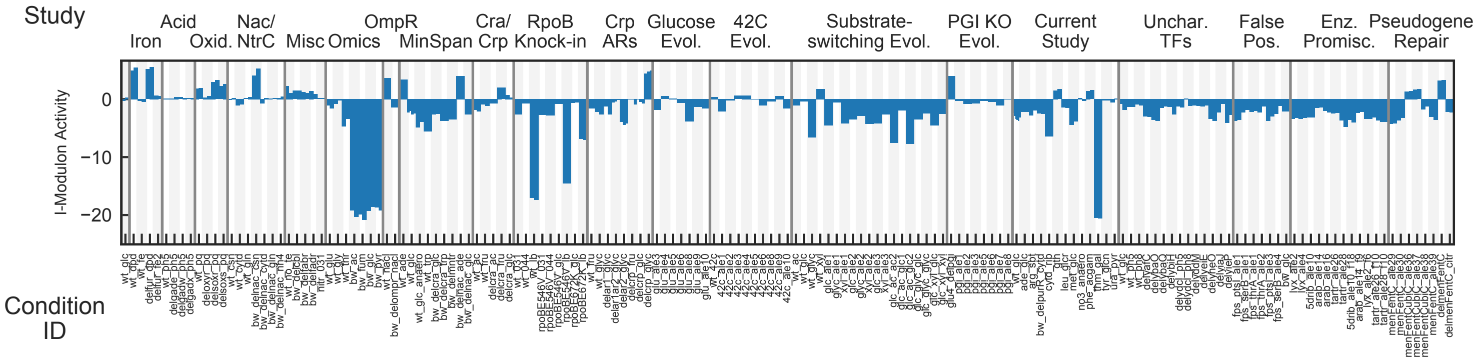

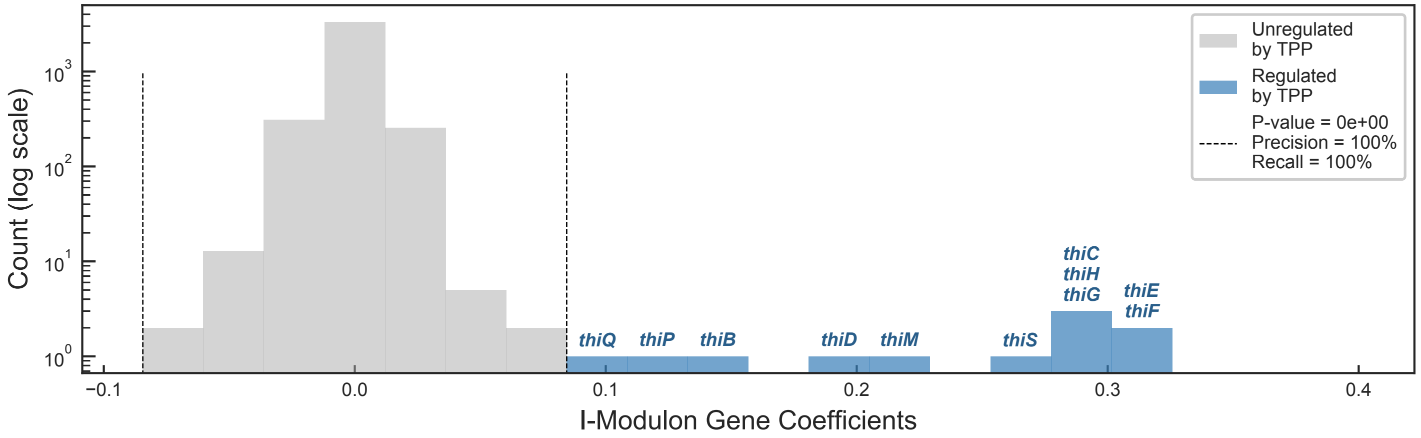

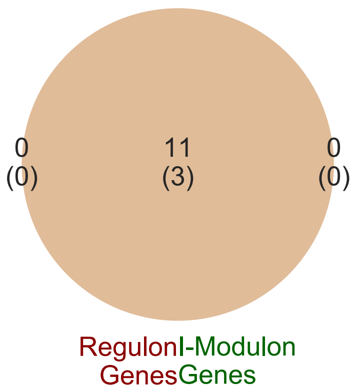

Tryptophan I-Modulon

Regulated by: TrpR or trp-tRNA attenuation or Tryptophan attenuation
Biological Function: Tryptophan Biosynthesis

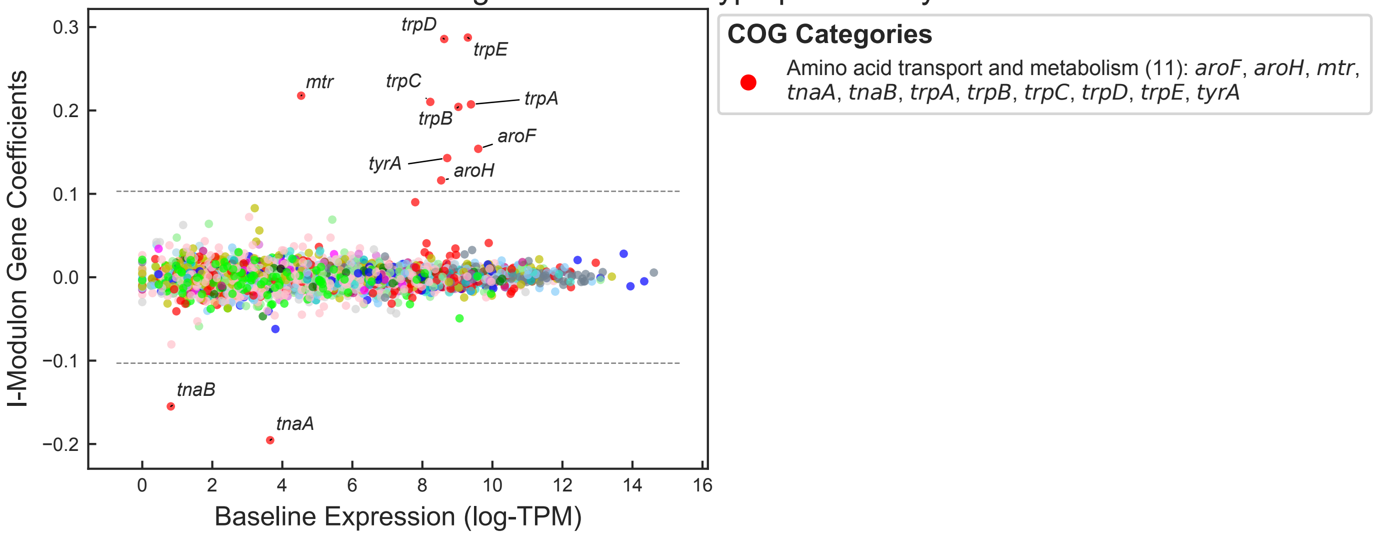

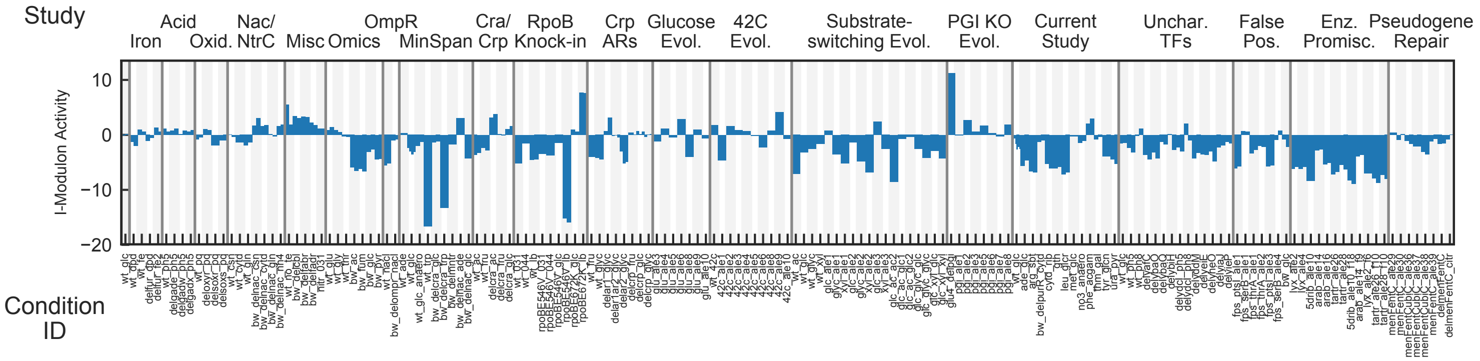

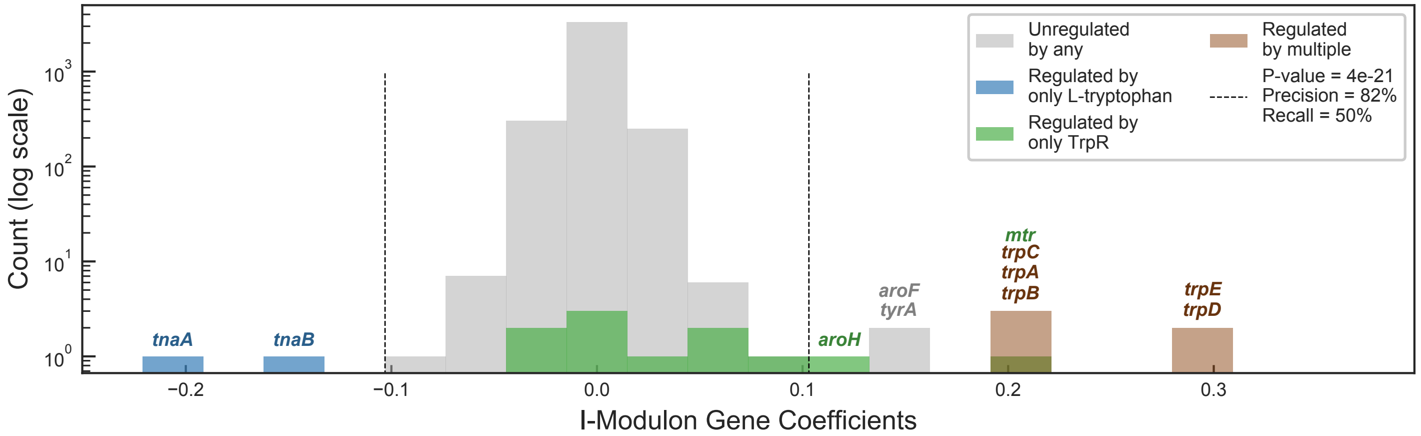

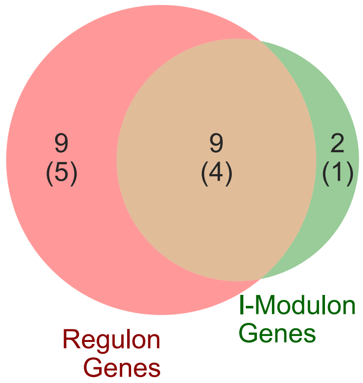

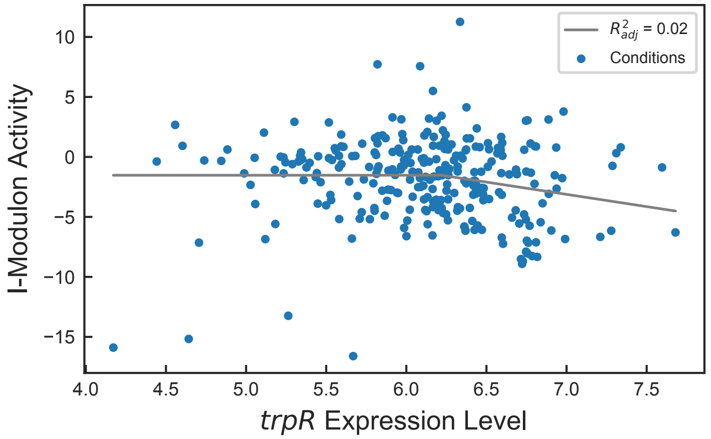

XylR I-Modulon

Regulated by: XylR
Biological Function: Xylose catabolism

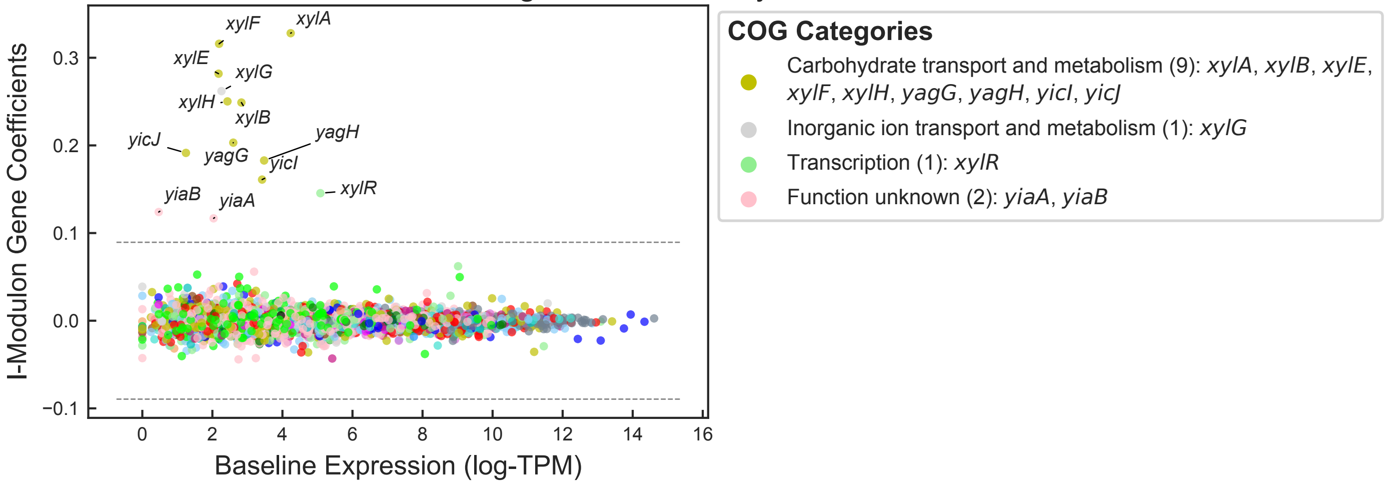

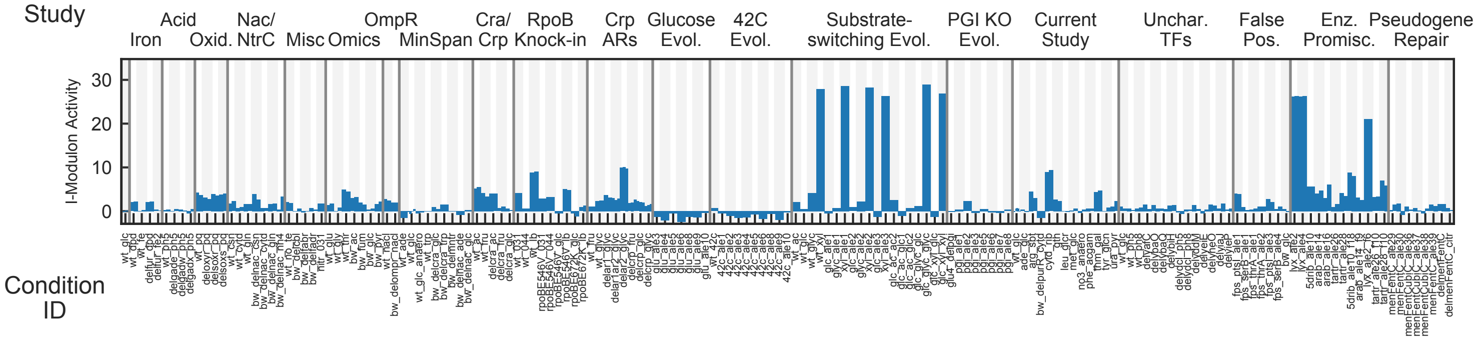

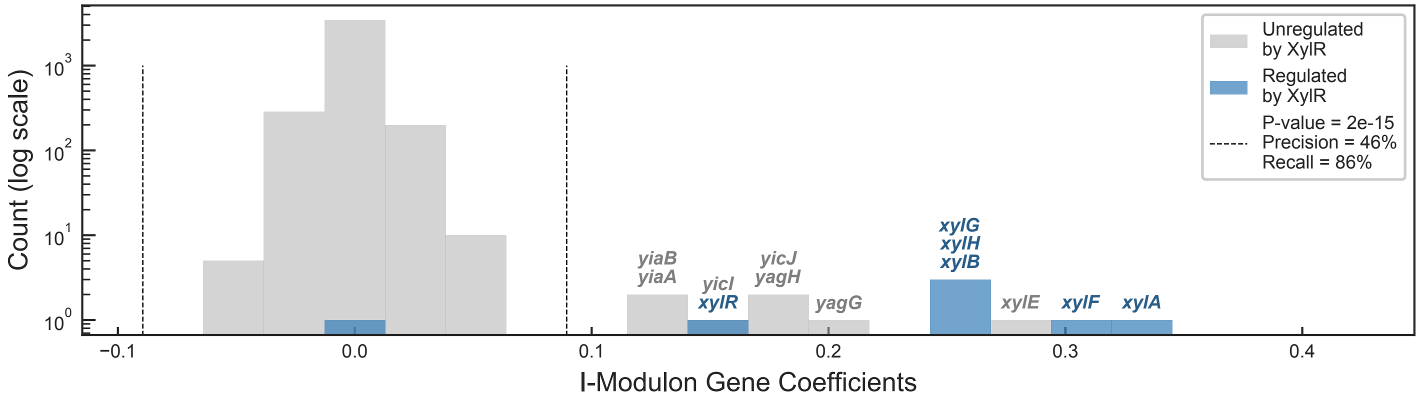

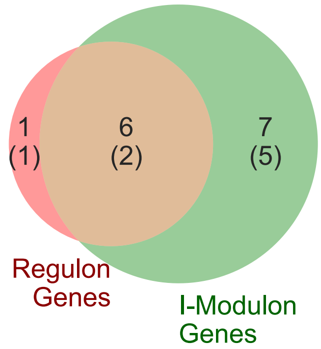

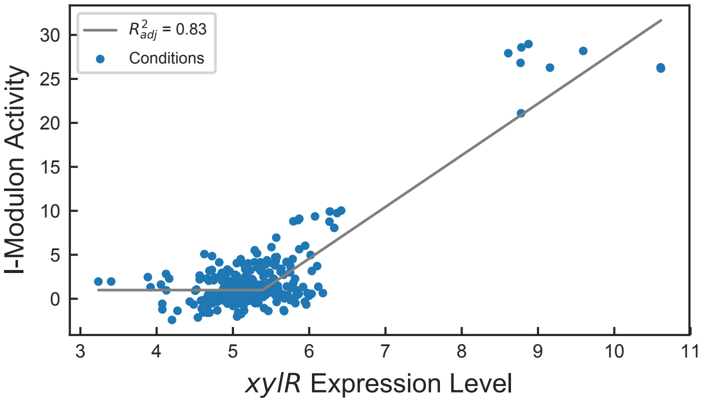

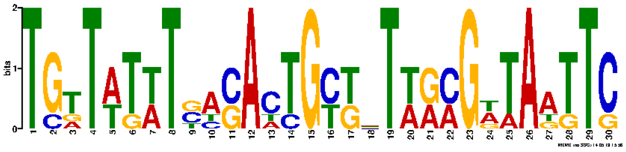

Motif E-value: 3.70e-04
Operons with Upstream Motif: 71%

YgbI I-Modulon

Regulated by: YgbI

Biological Function: Unknown Function

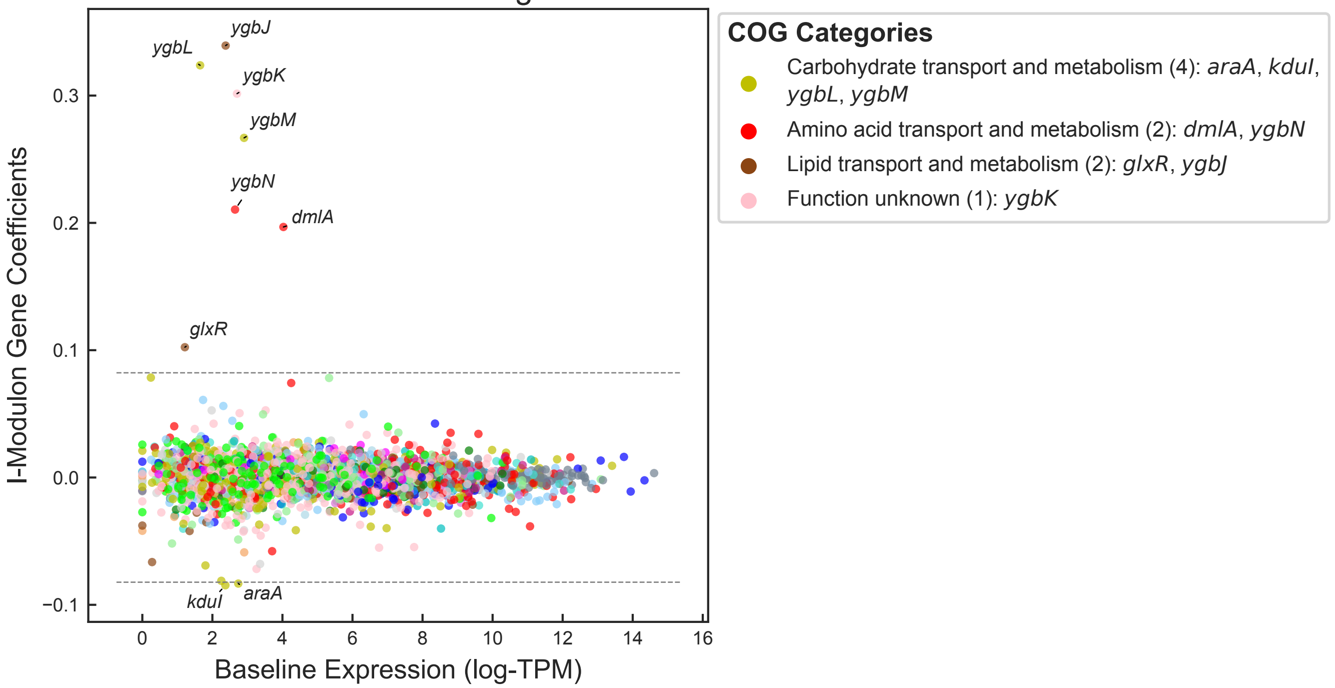

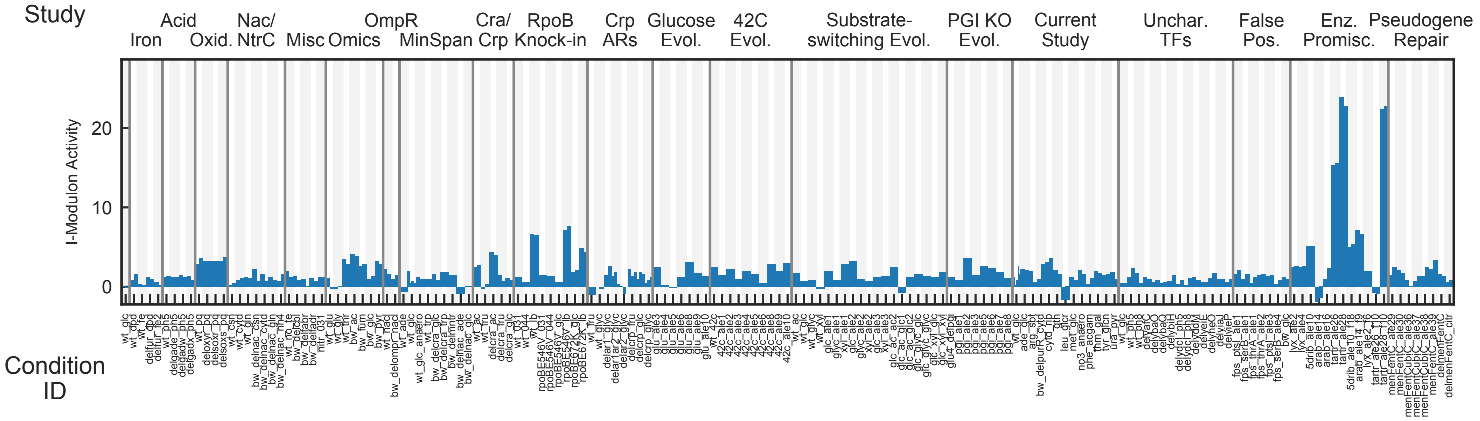

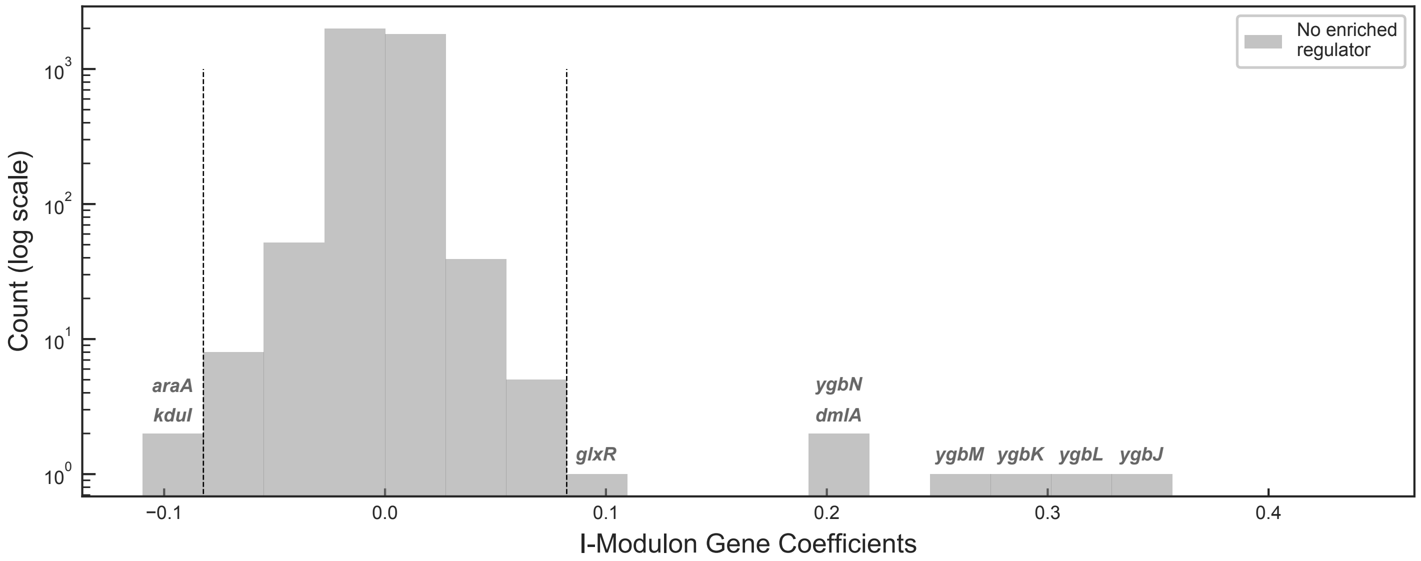

YiaJ I-Modulon

Regulated by: YiaJ

Biological Function: Ascorbate utilization

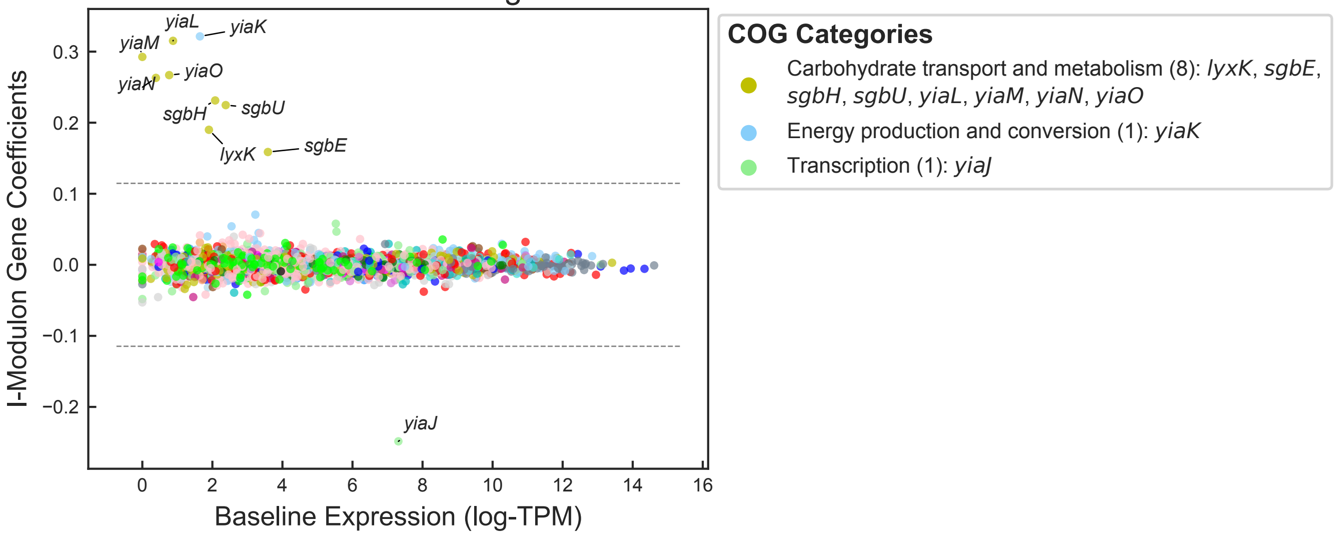

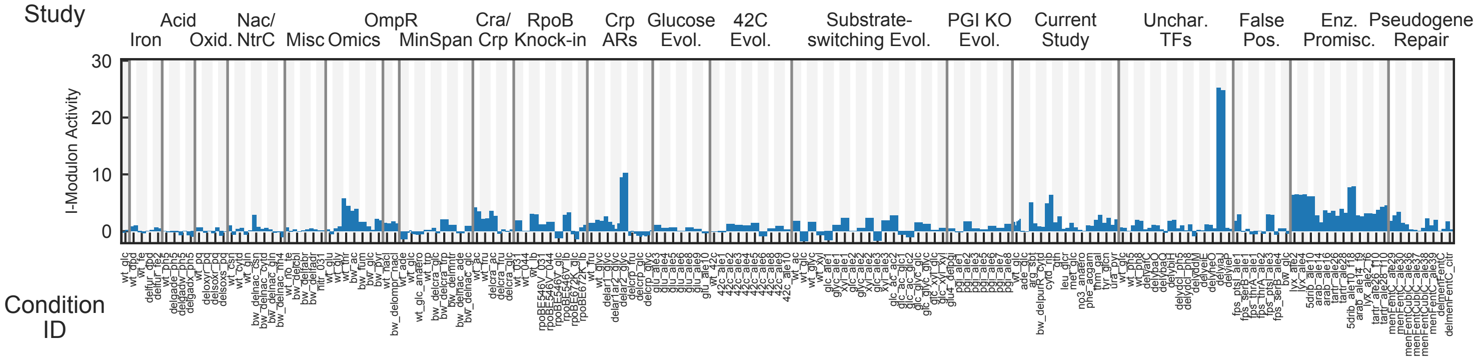

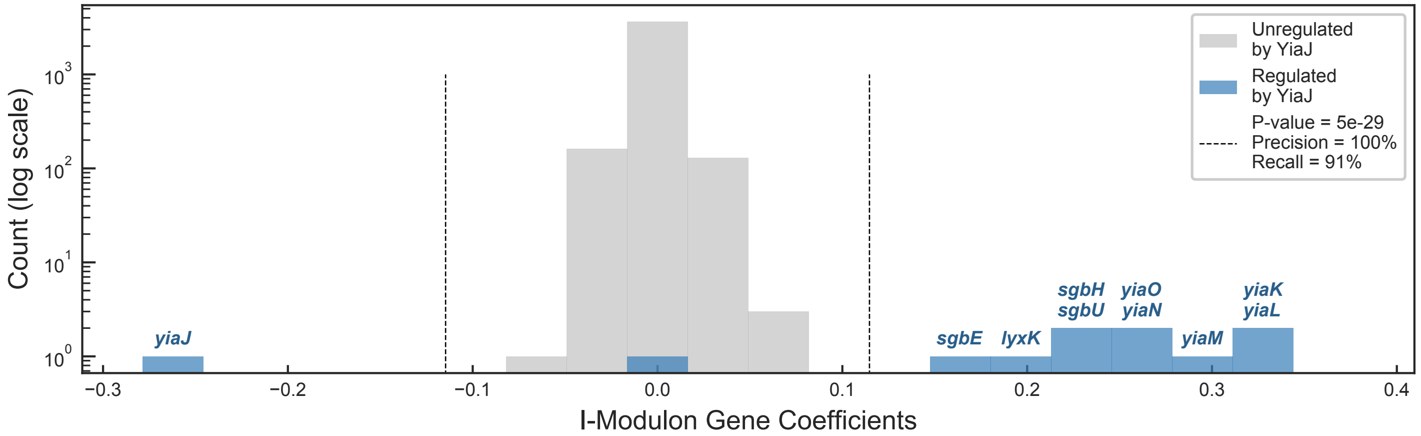

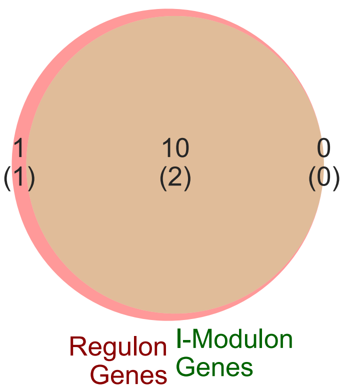

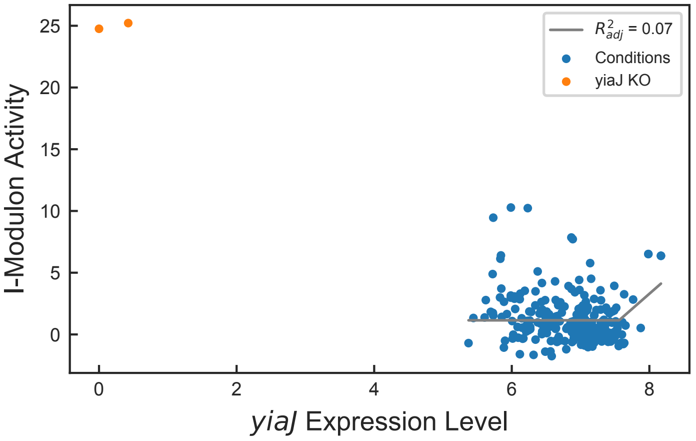

YieP I-Modulon

Regulated by: YieP

Biological Function: Unknown

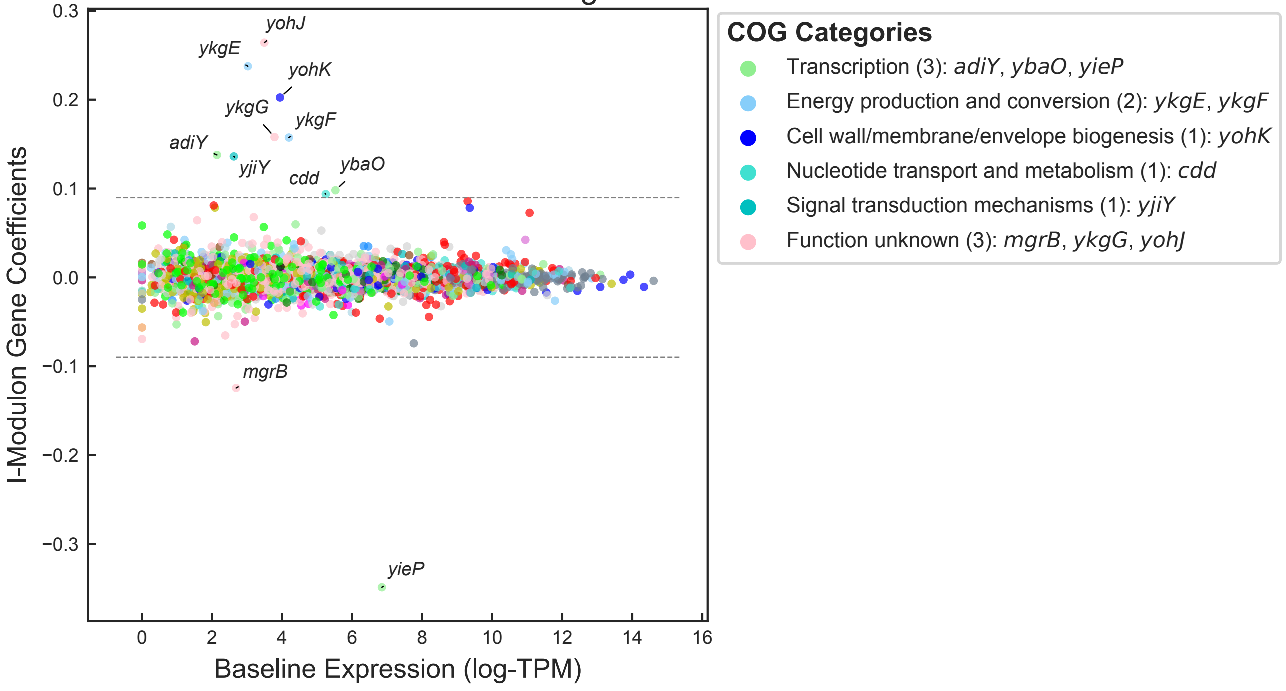

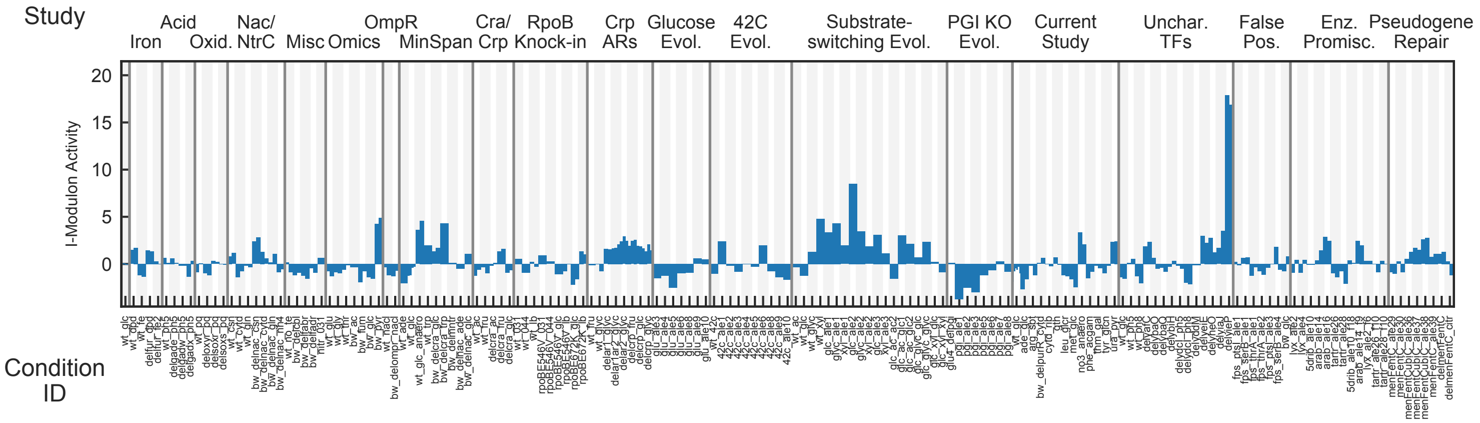

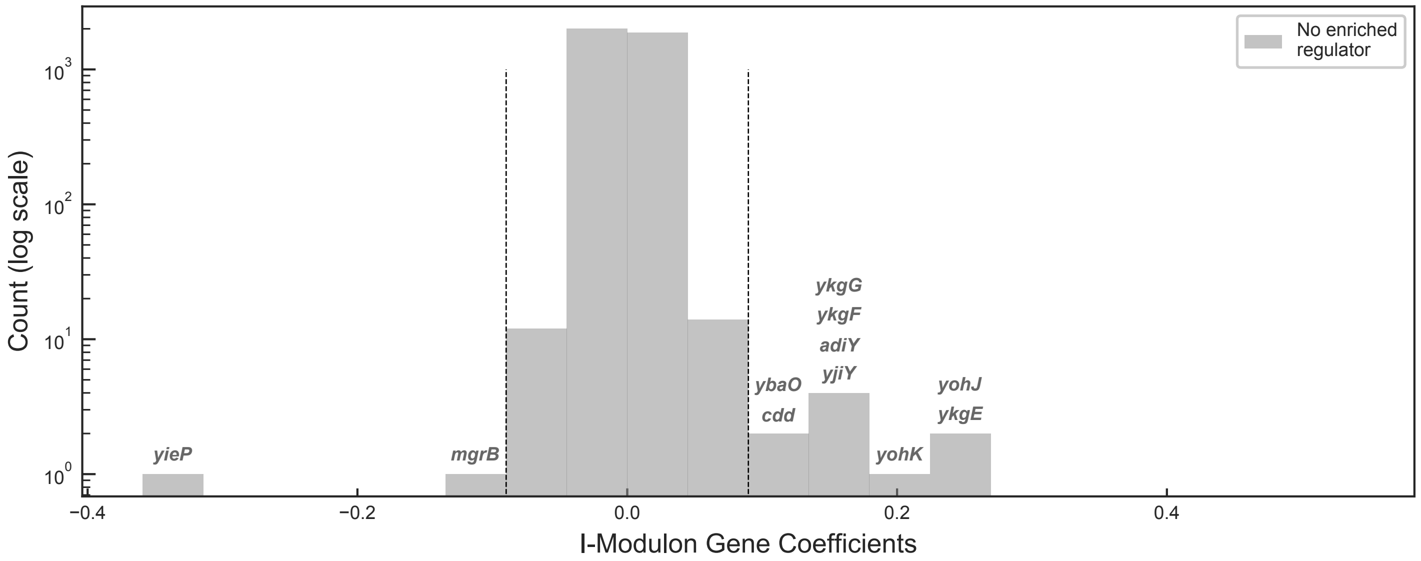

YneJ I-Modulon

Regulated by: YneJ

Biological Function: Unknown Function

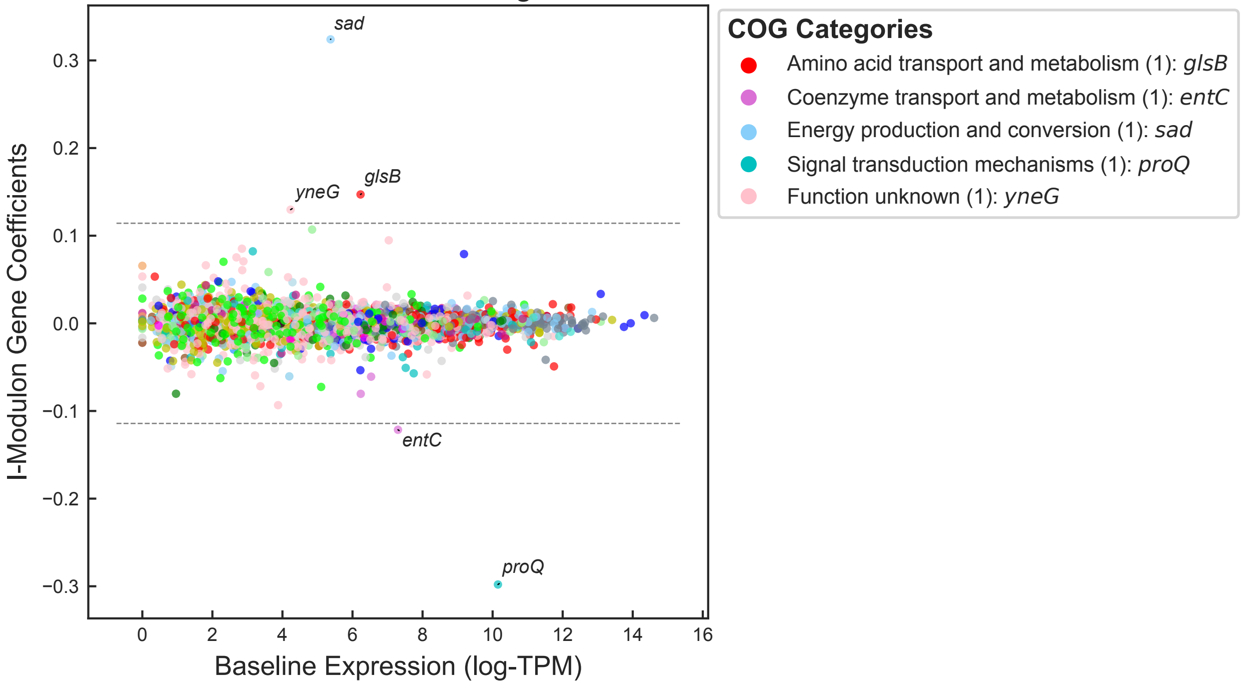

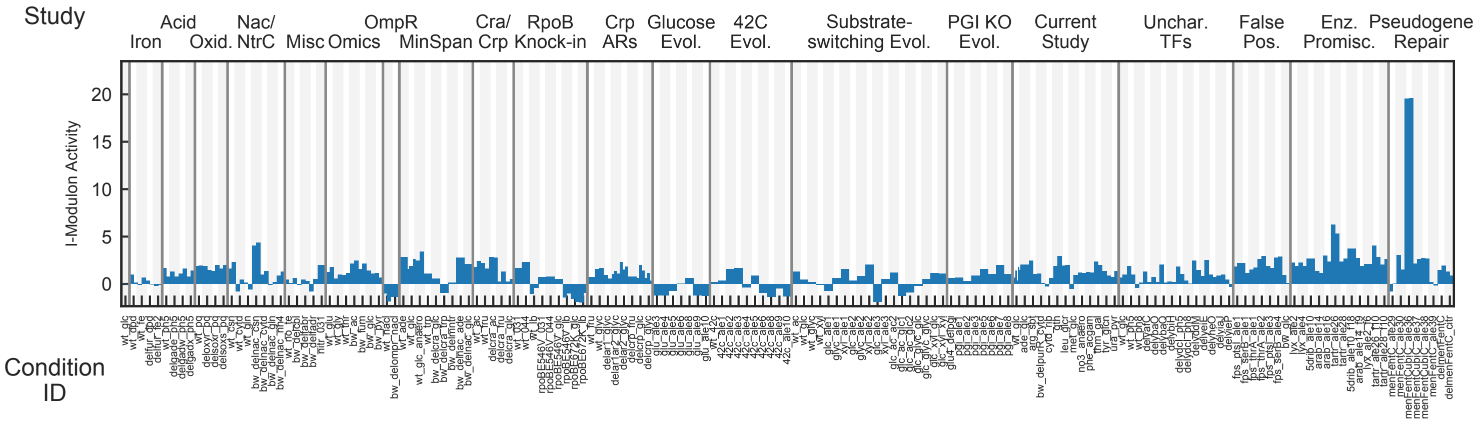

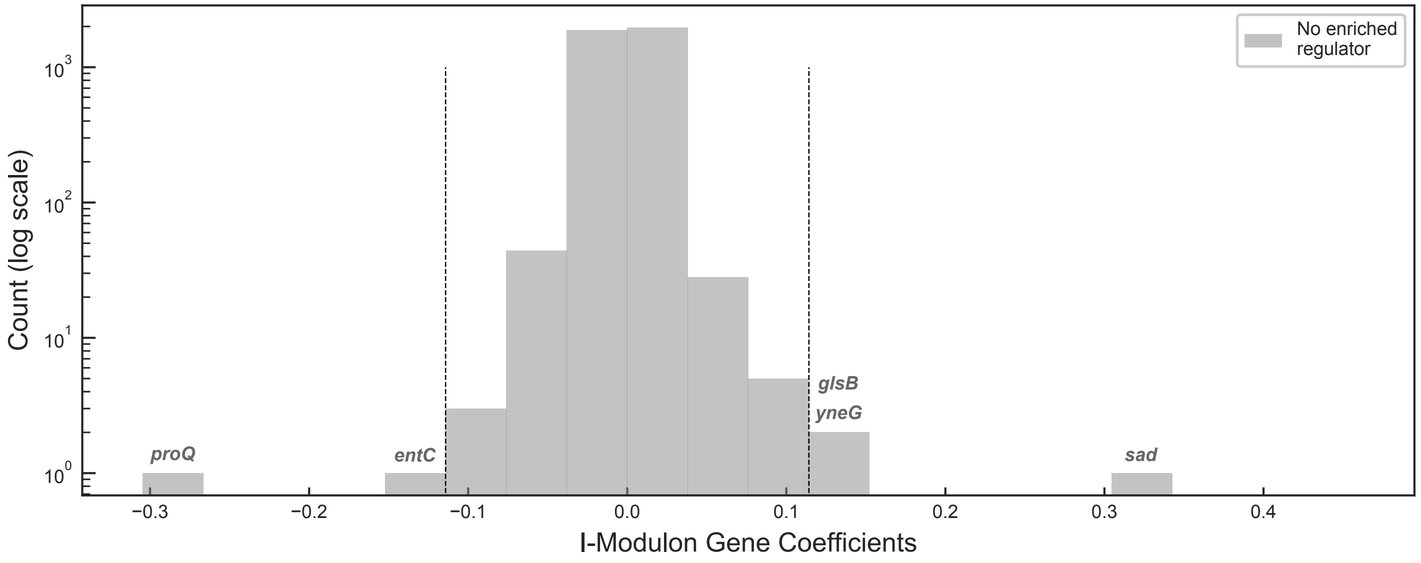

Zinc I-Modulon

Regulated by: ZntR or Zur

Biological Function: Zinc homeostasis

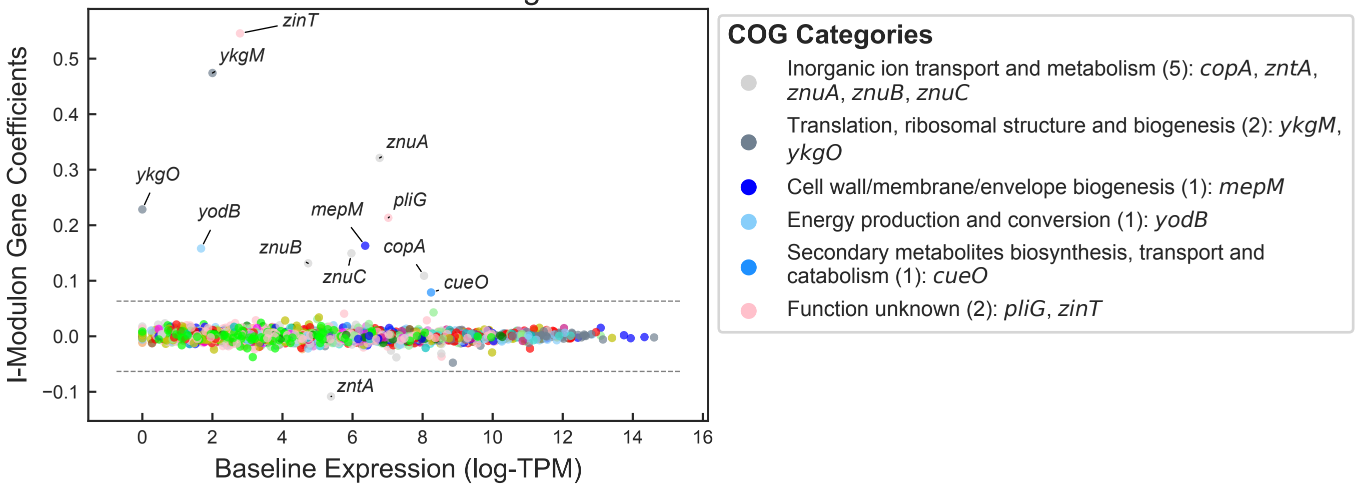

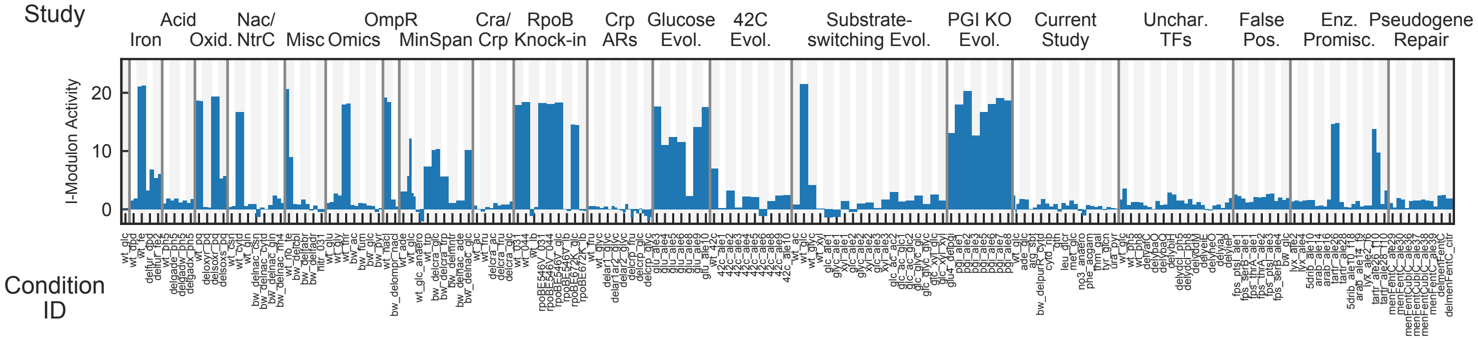

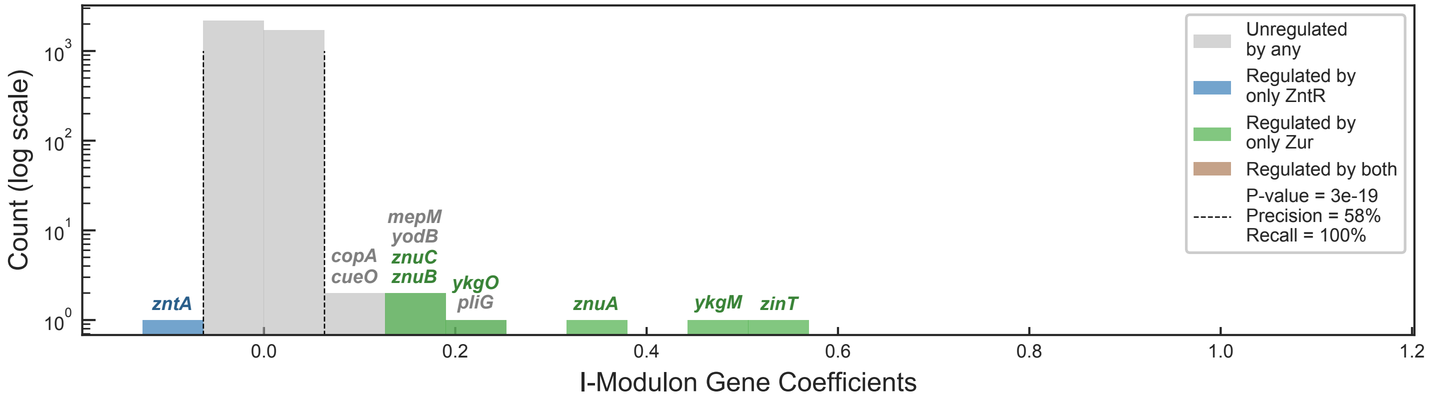

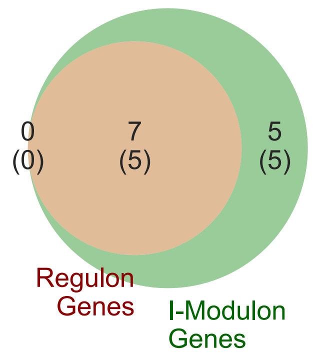

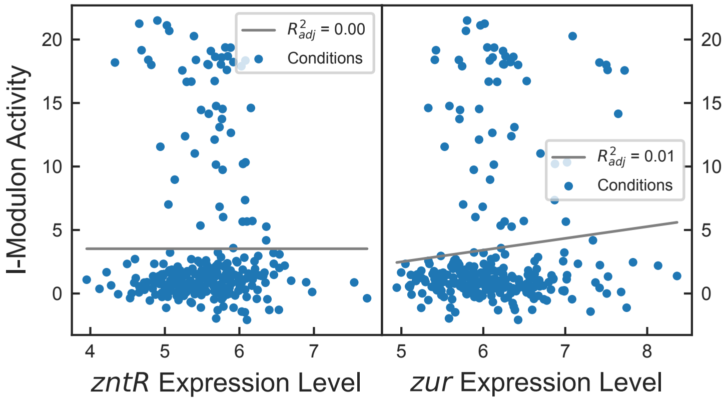

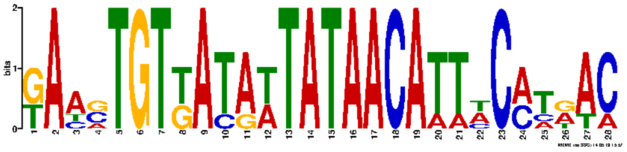

Motif E-value: 4.50e-06

Operons with Upstream Motif: 50%

crp – KO I-Modulon

Biological Function: Accounts for crp knock-out

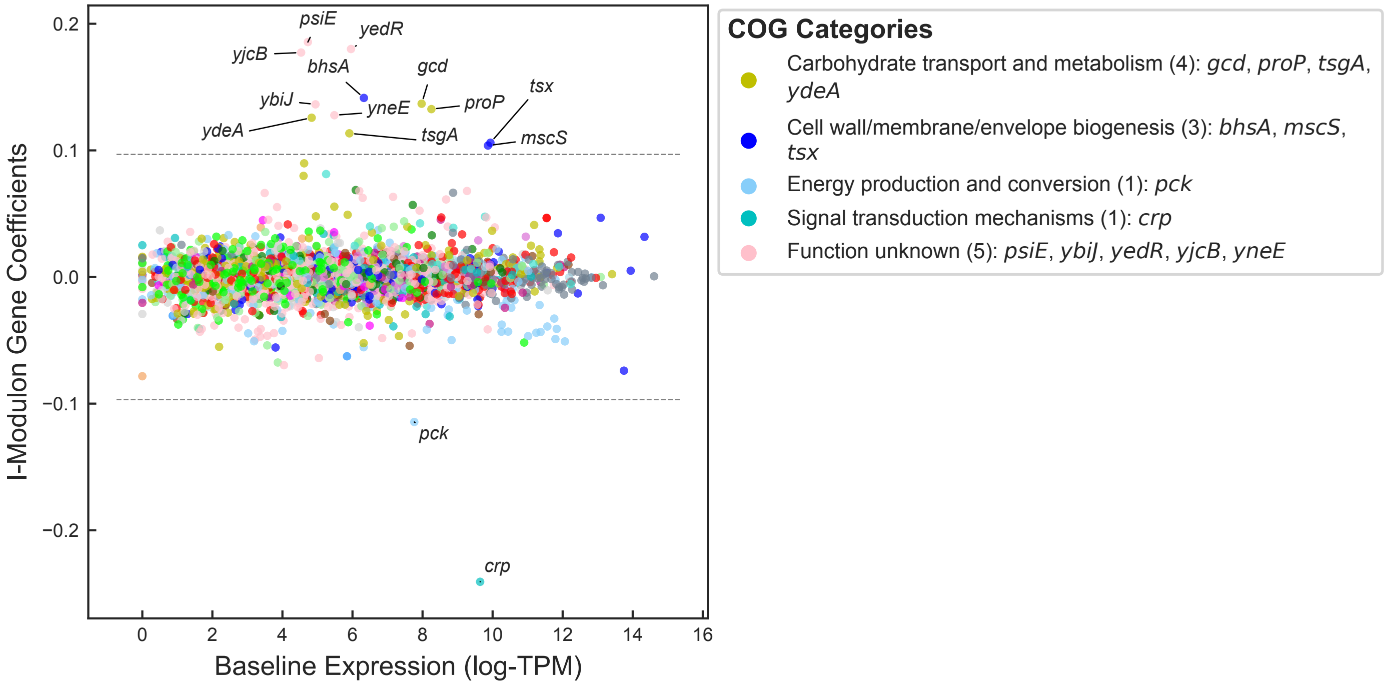

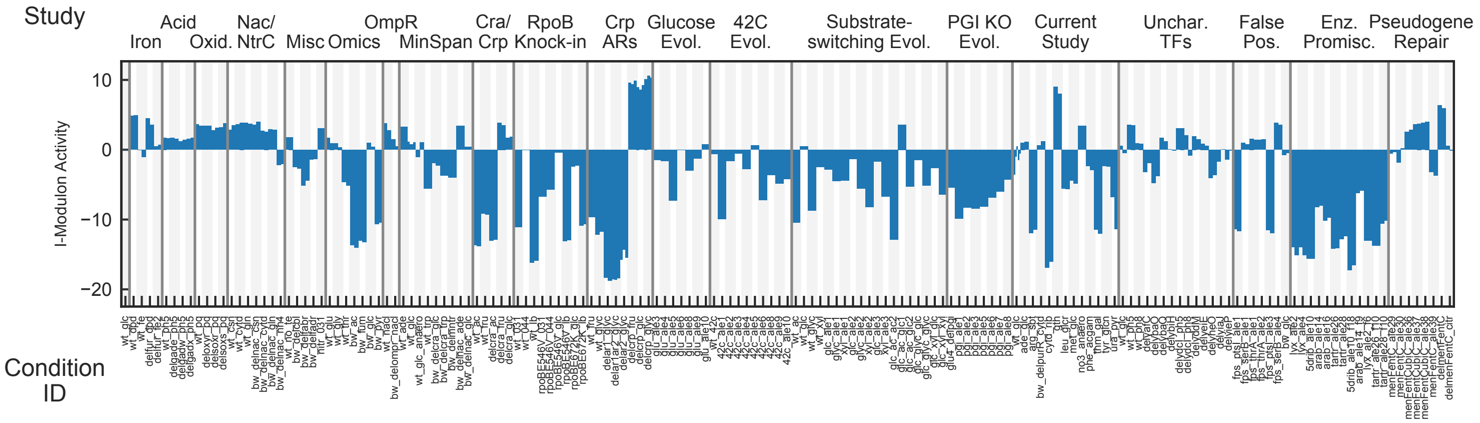

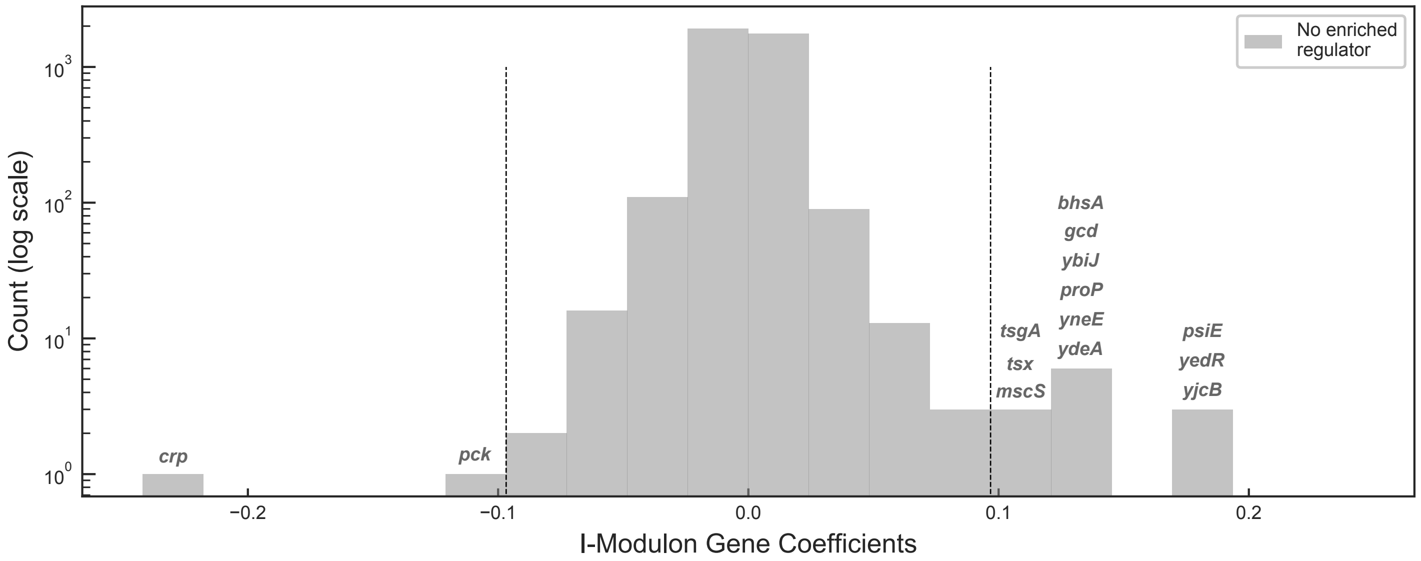

curli I-Modulon

Biological Function: Curli assembly

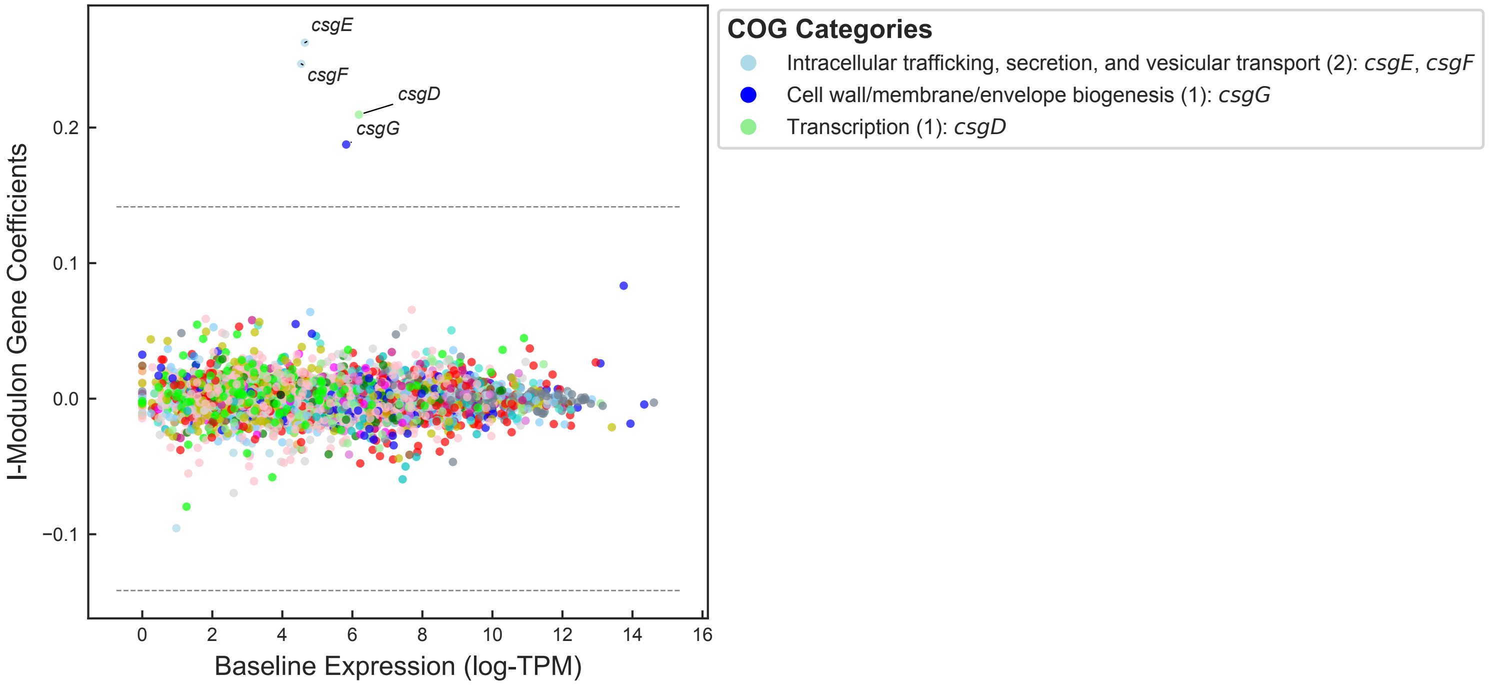

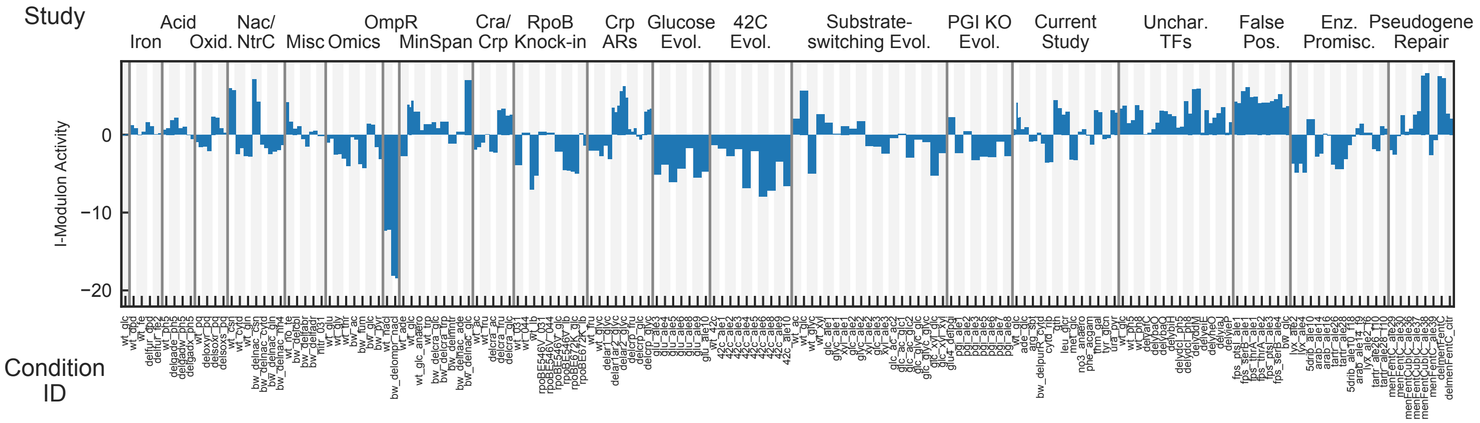

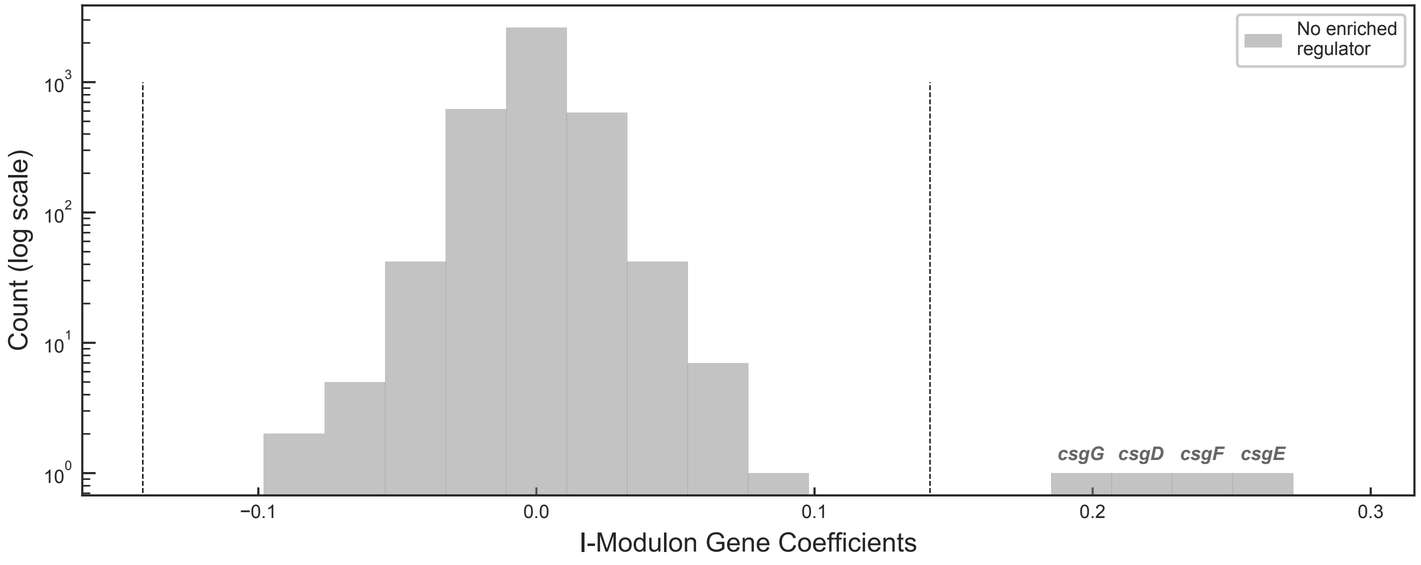

deletion − 1 I-Modulon

Biological Function: Large deletion of 39 genes during evolution

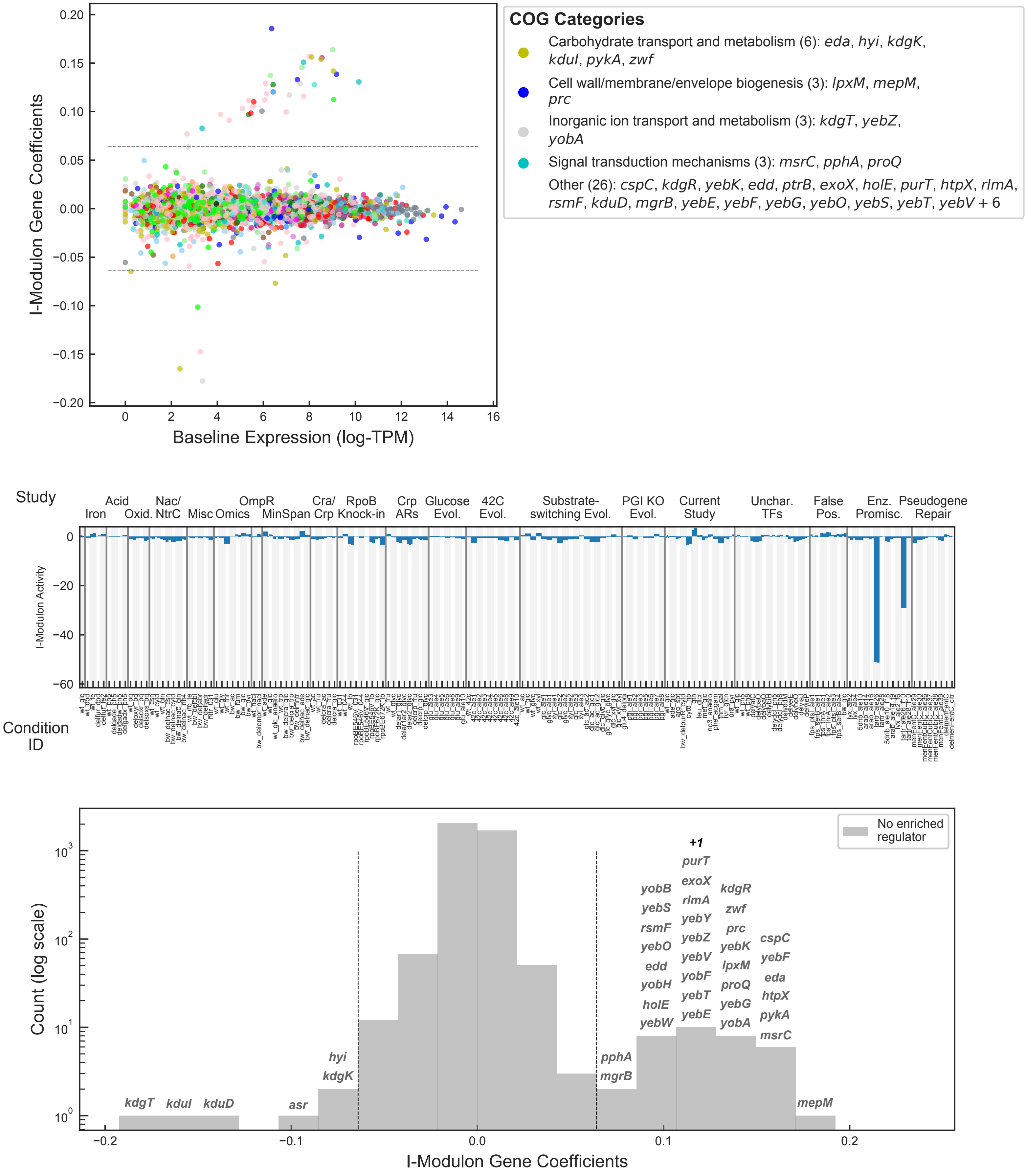

deletion − 2 I-Modulon

Biological Function: Large deletion of 171 genes during evolution

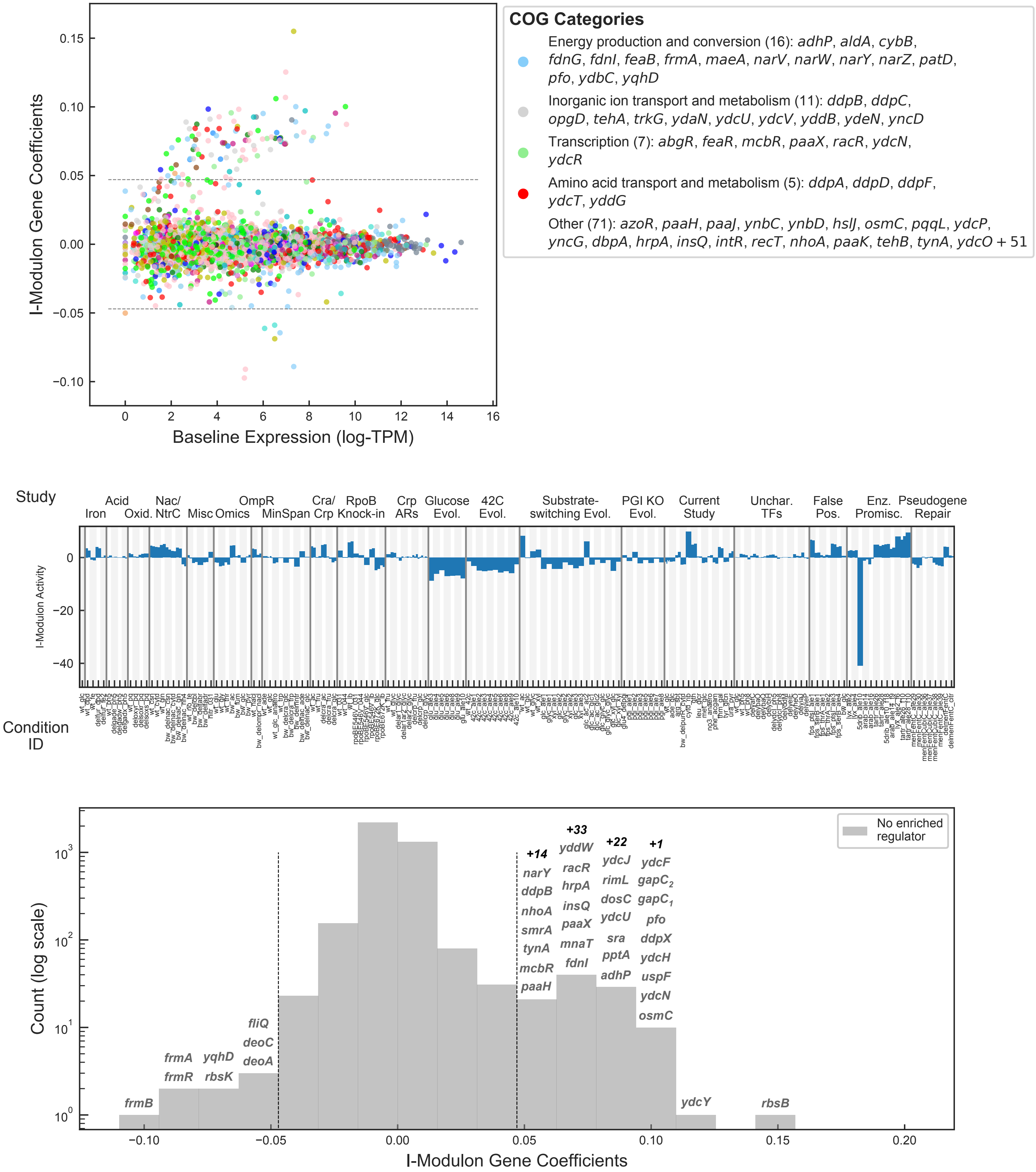

duplication − 1 I-Modulon

Biological Function: Large duplication of 129 genes during evolution

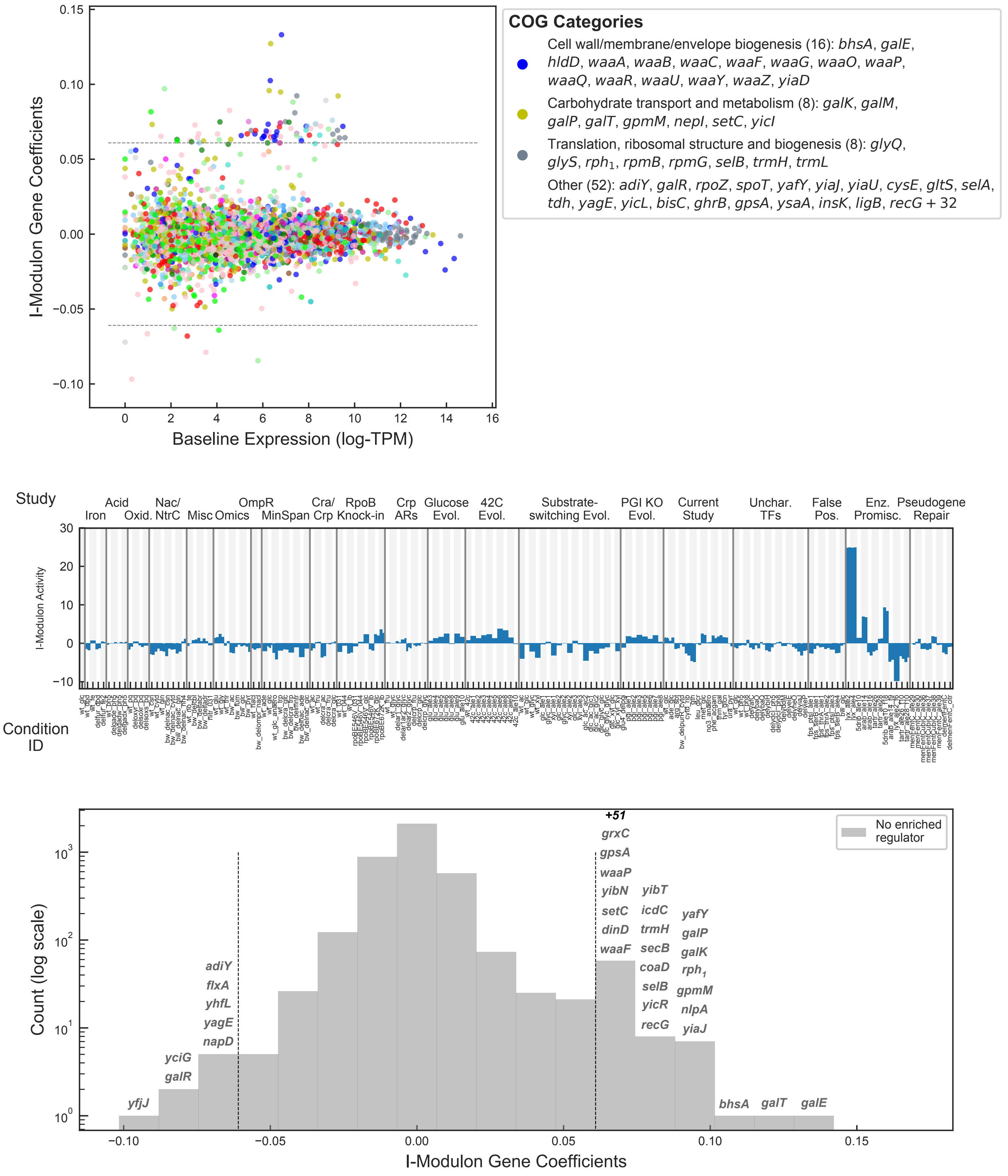

e14 – deletion I-Modulon

Biological Function: Removal of e14 prophage

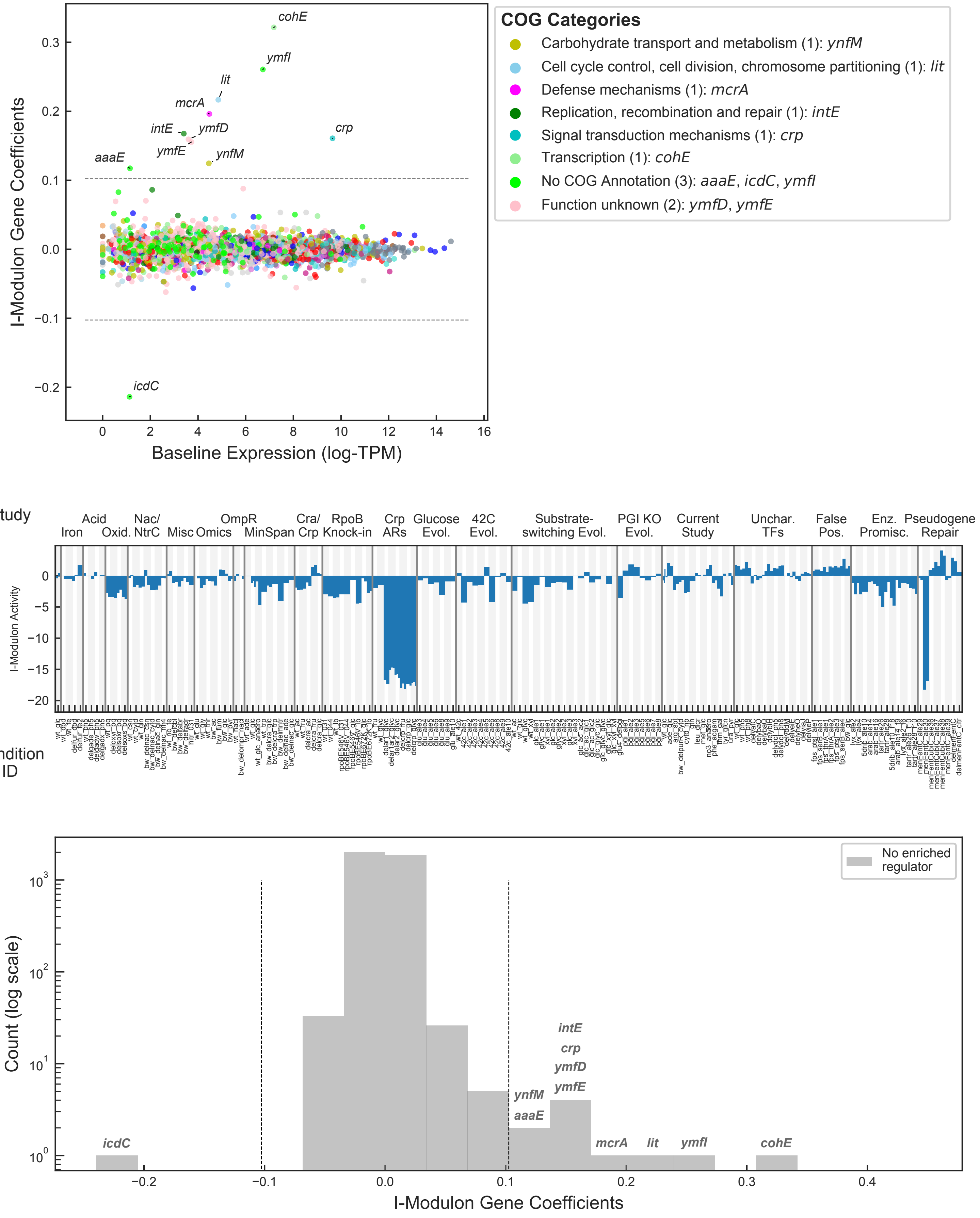

efeU – repair I-Modulon

Biological Function: Accounts for repair and expression of efeU operon

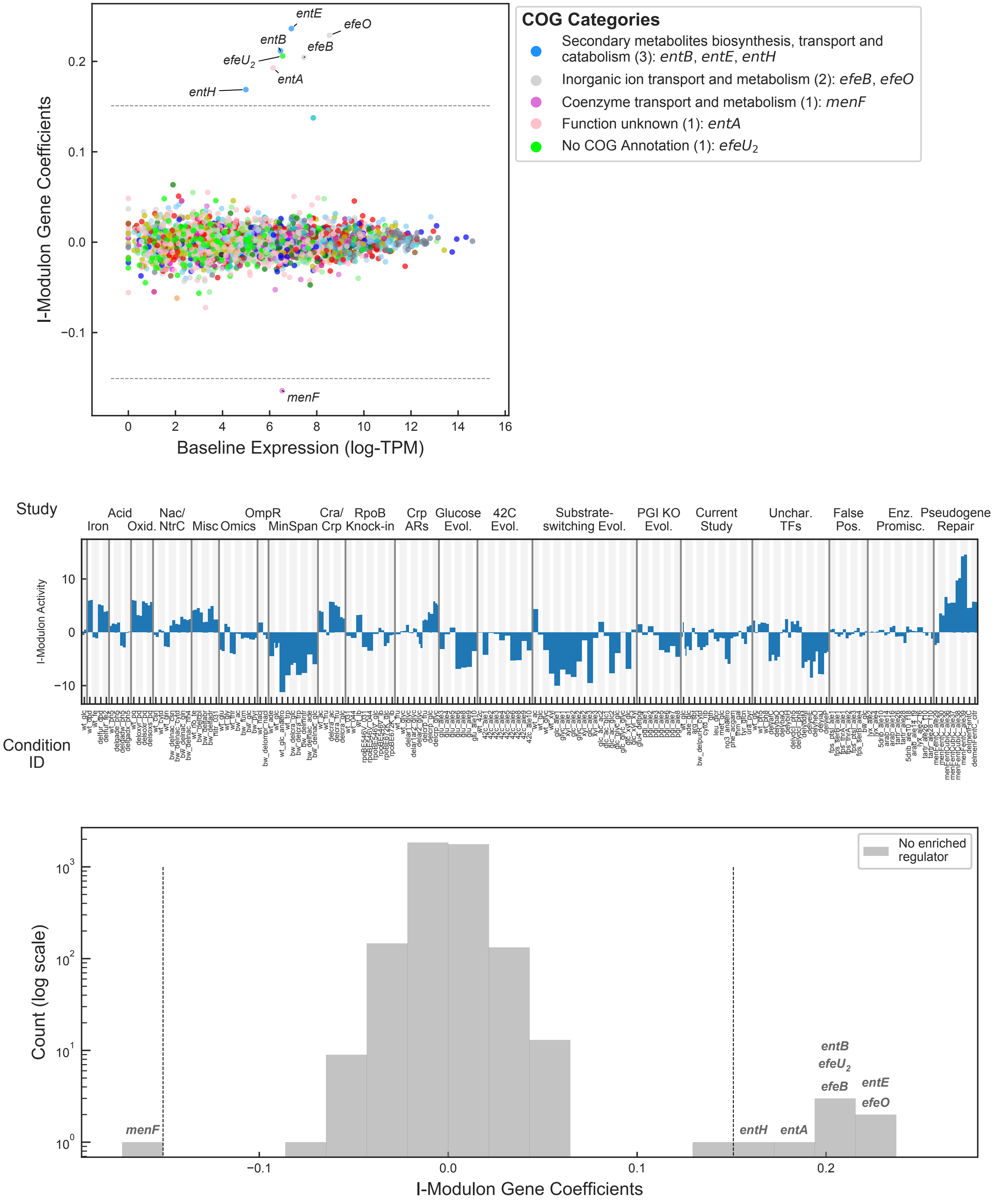

entC – menF – KO I-Modulon

Biological Function: Accounts for entC and menF knock-outs

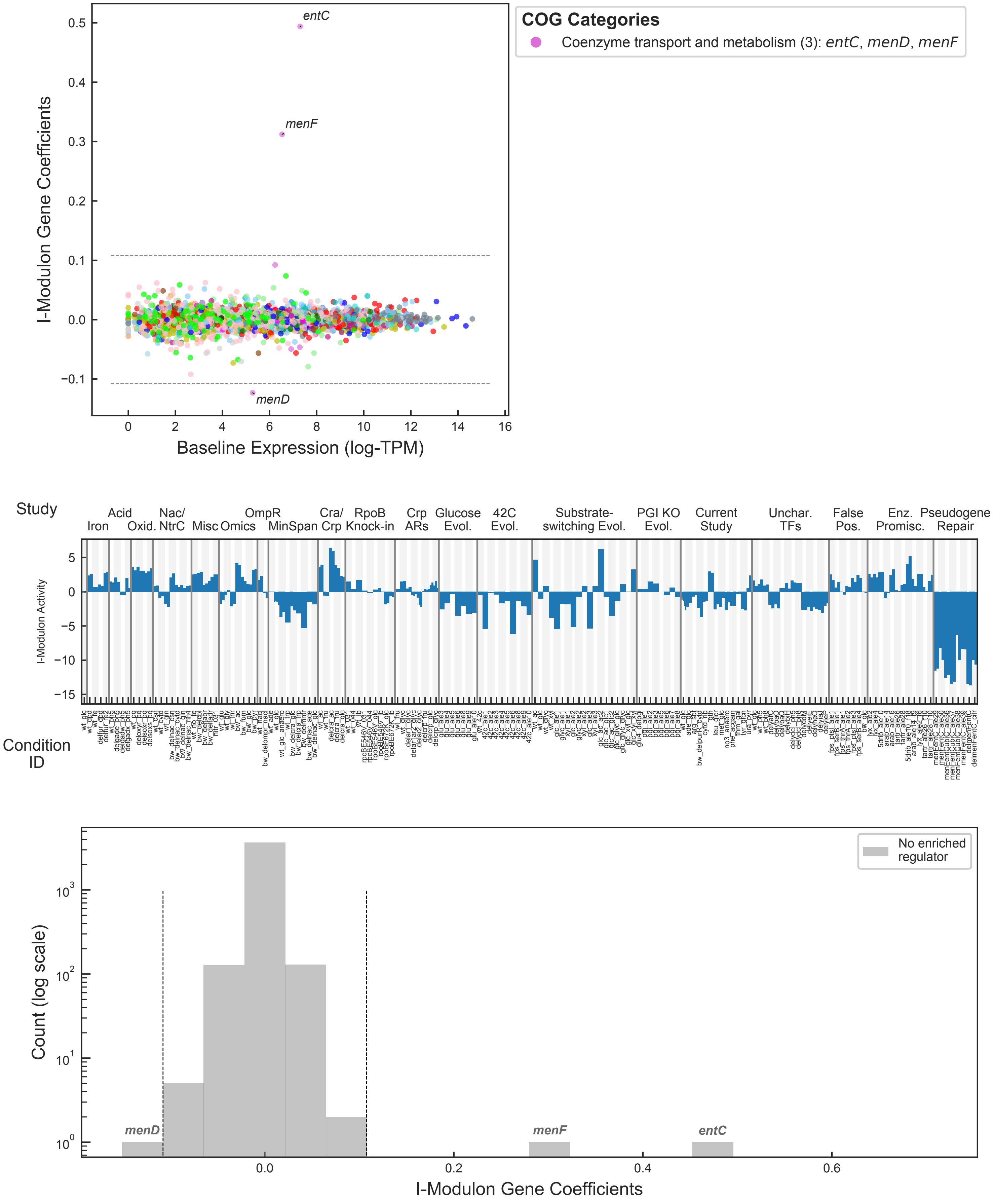

fimbriae I-Modulon

Biological Function: Fimbriae assembly

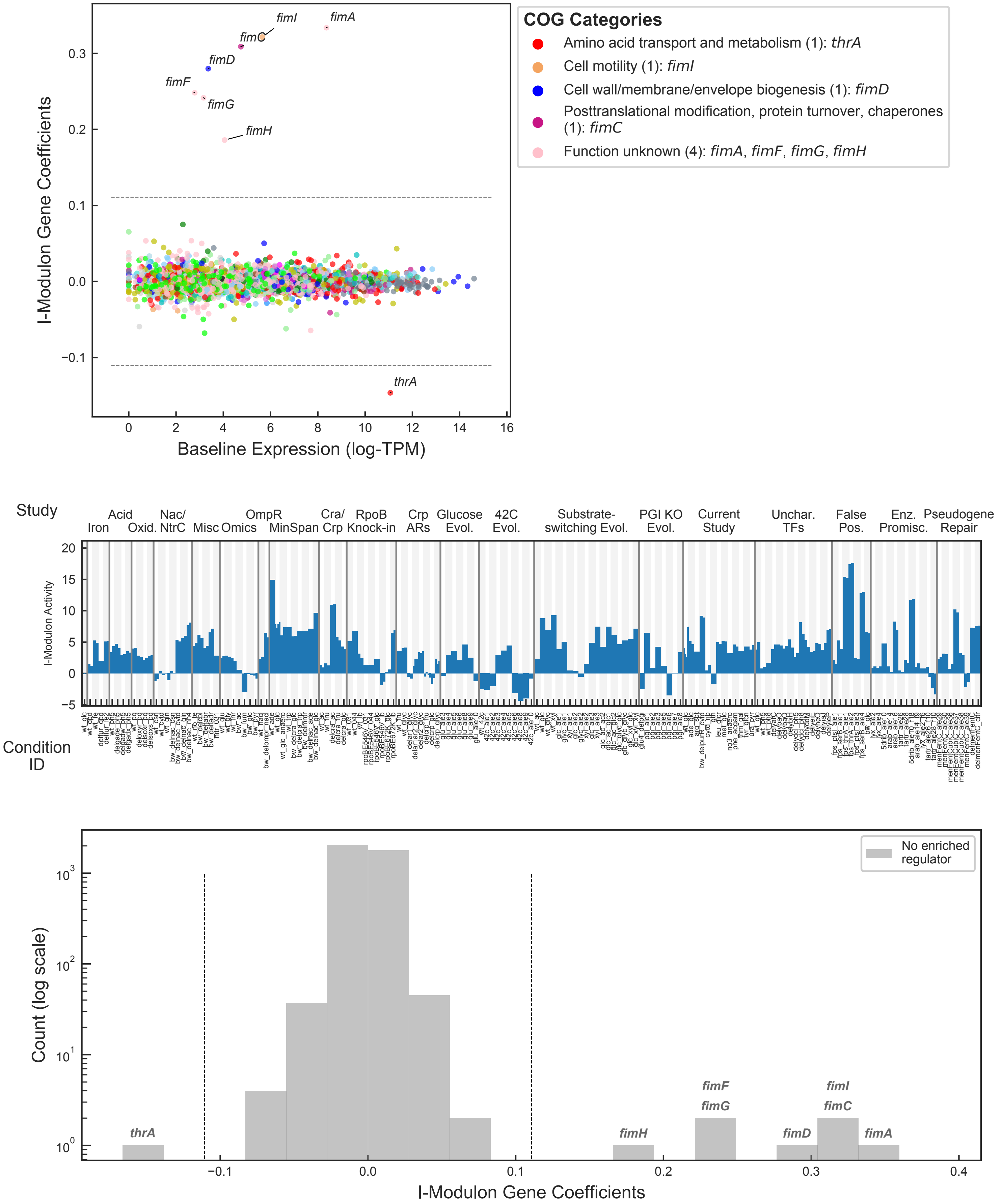

flu – yeeRS I-Modulon

Biological Function: Genes in CP4-44 prophage

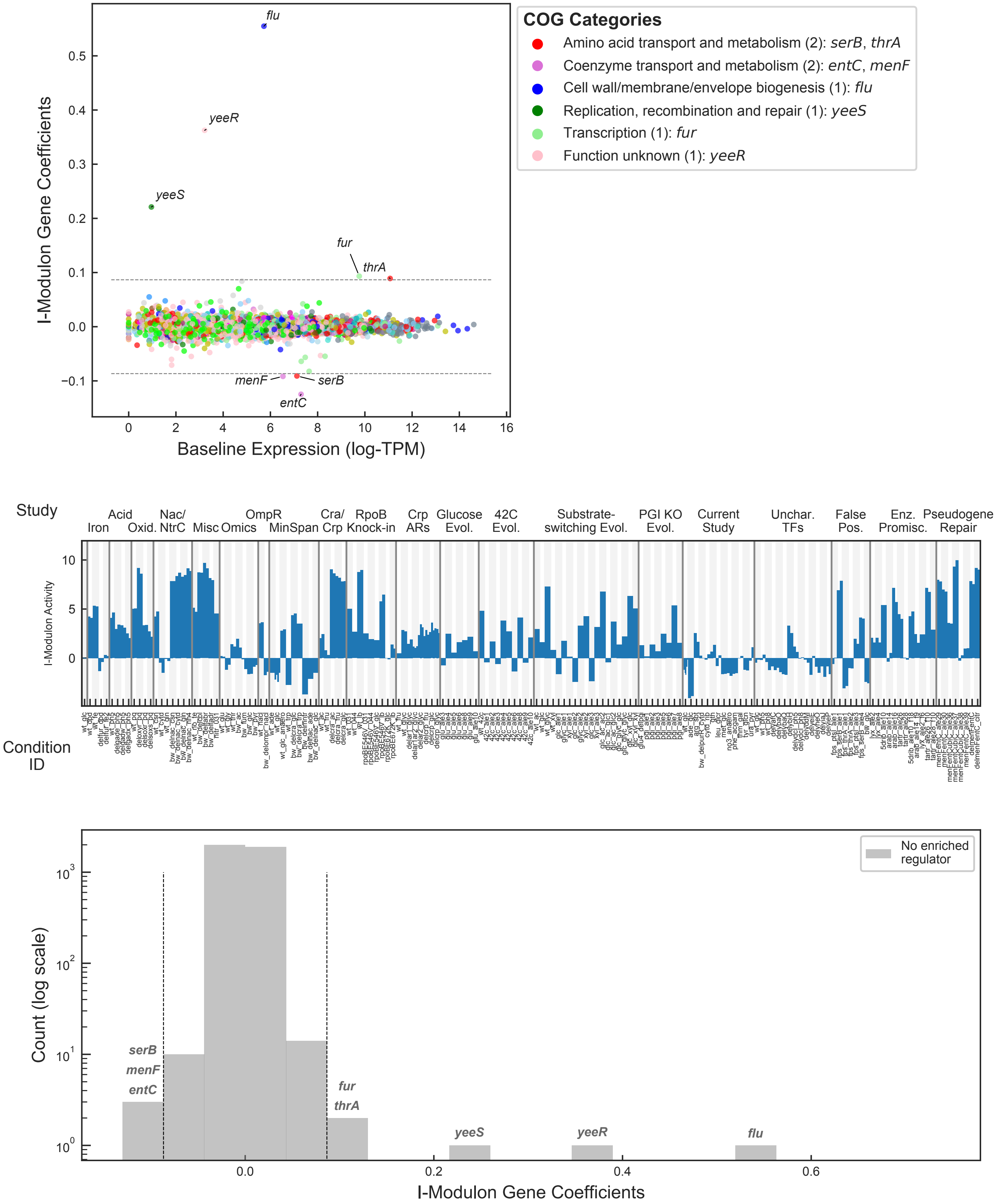

fur – KO I-Modulon

Biological Function: Accounts for fur knock-out

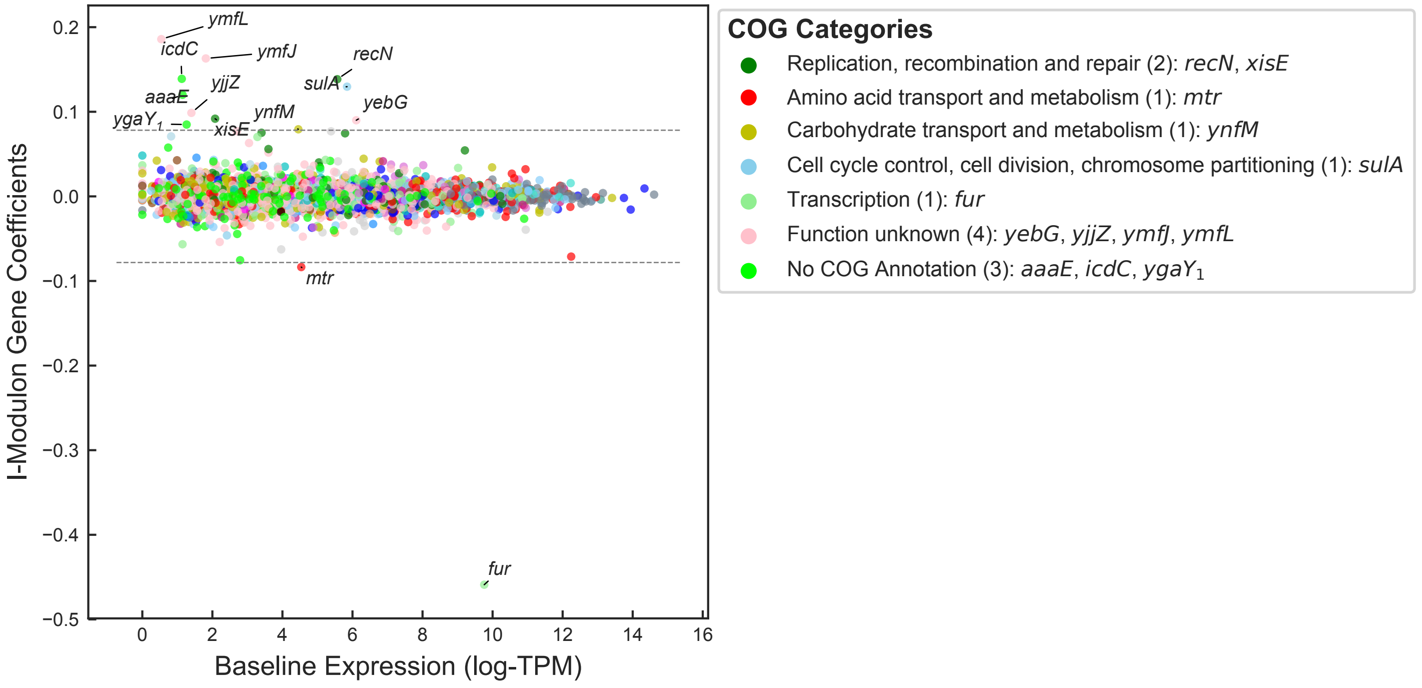

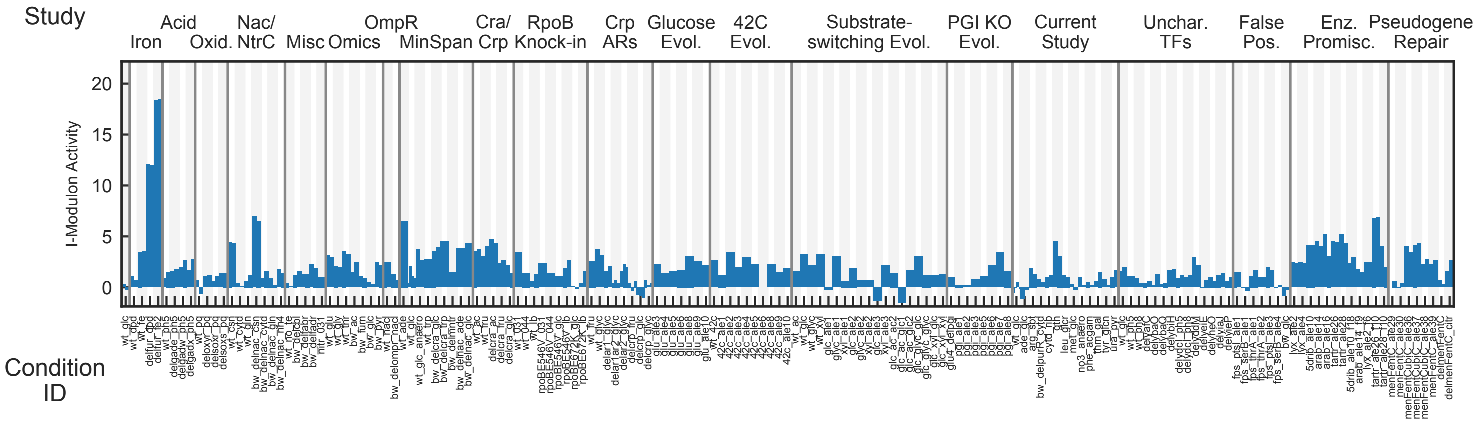

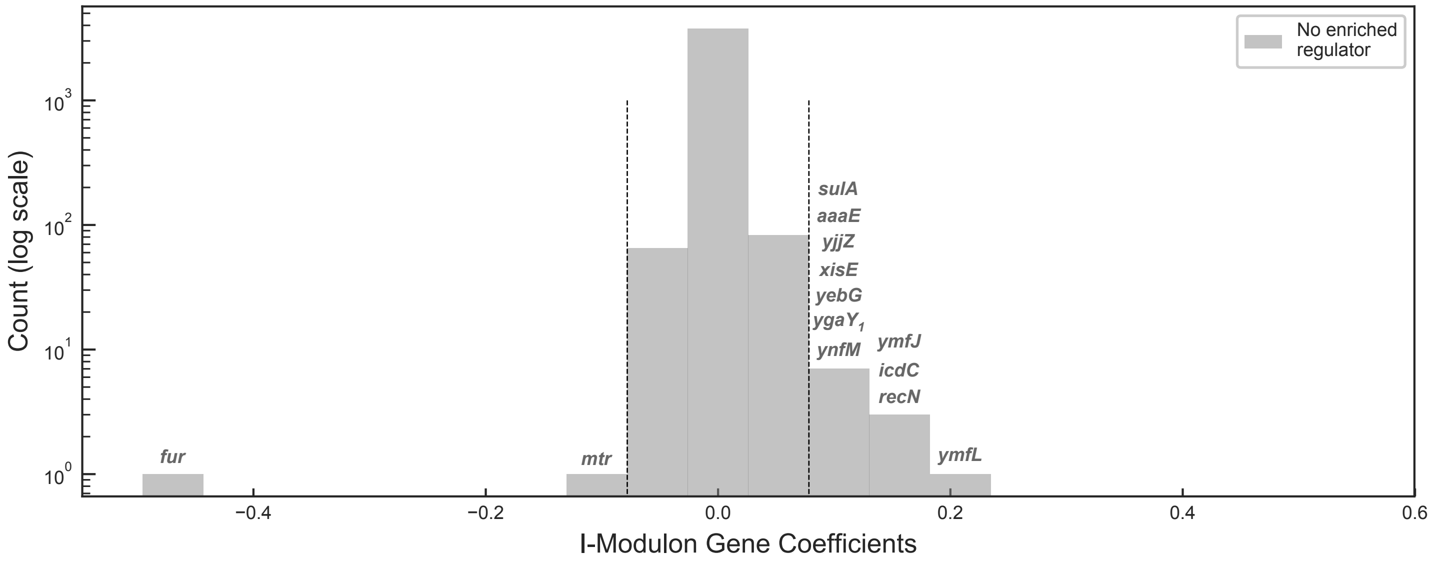

gadWX – KO I-Modulon

Biological Function: Accounts for gadW and gadX knock-outs

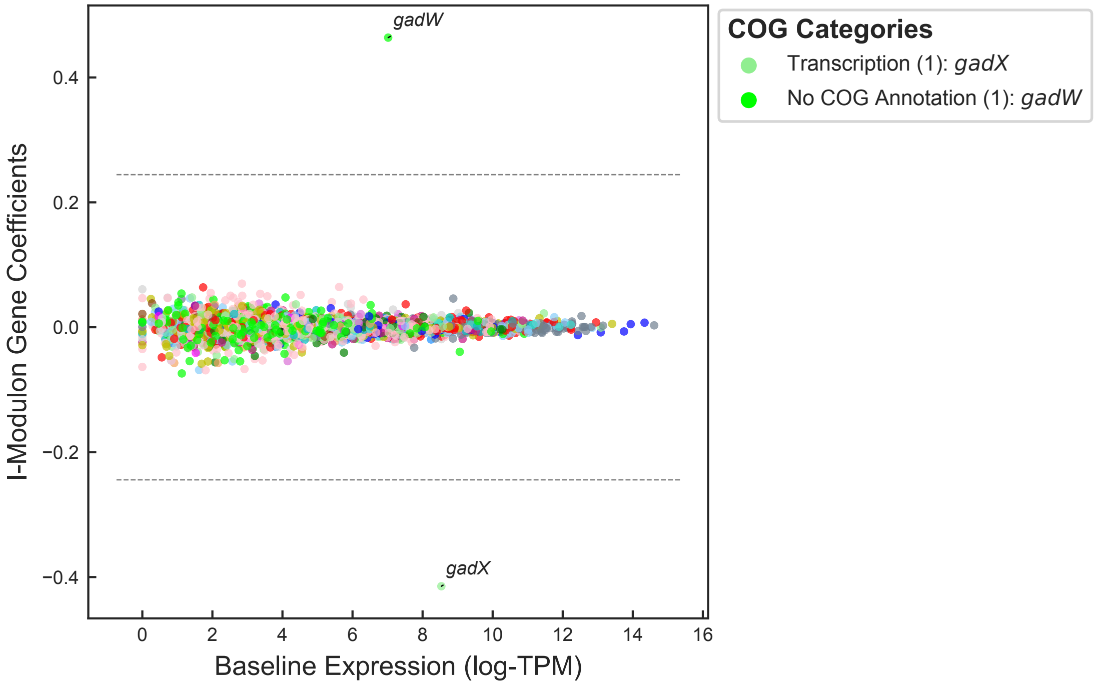

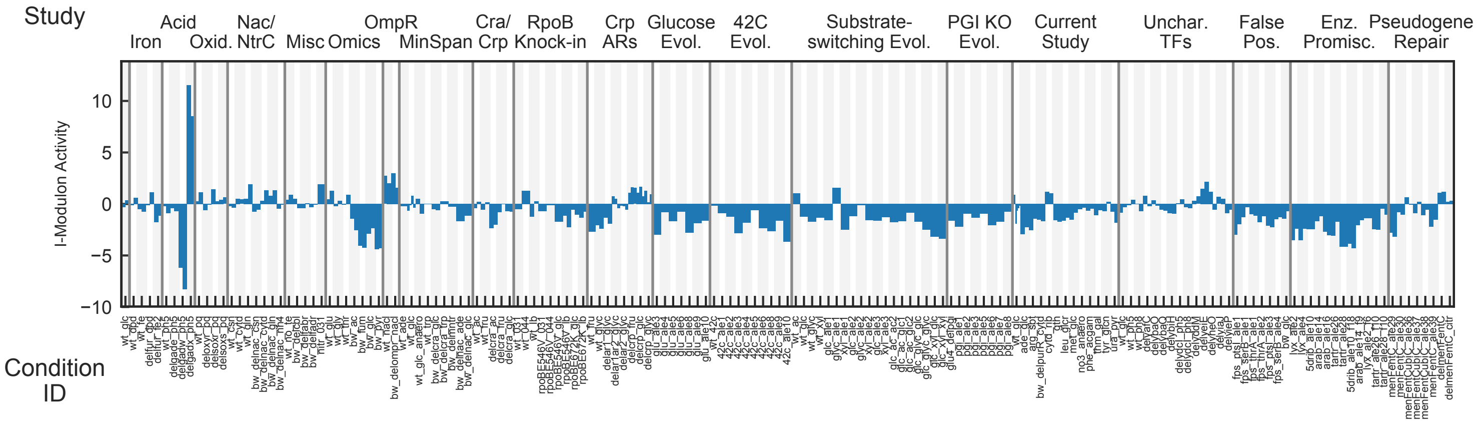

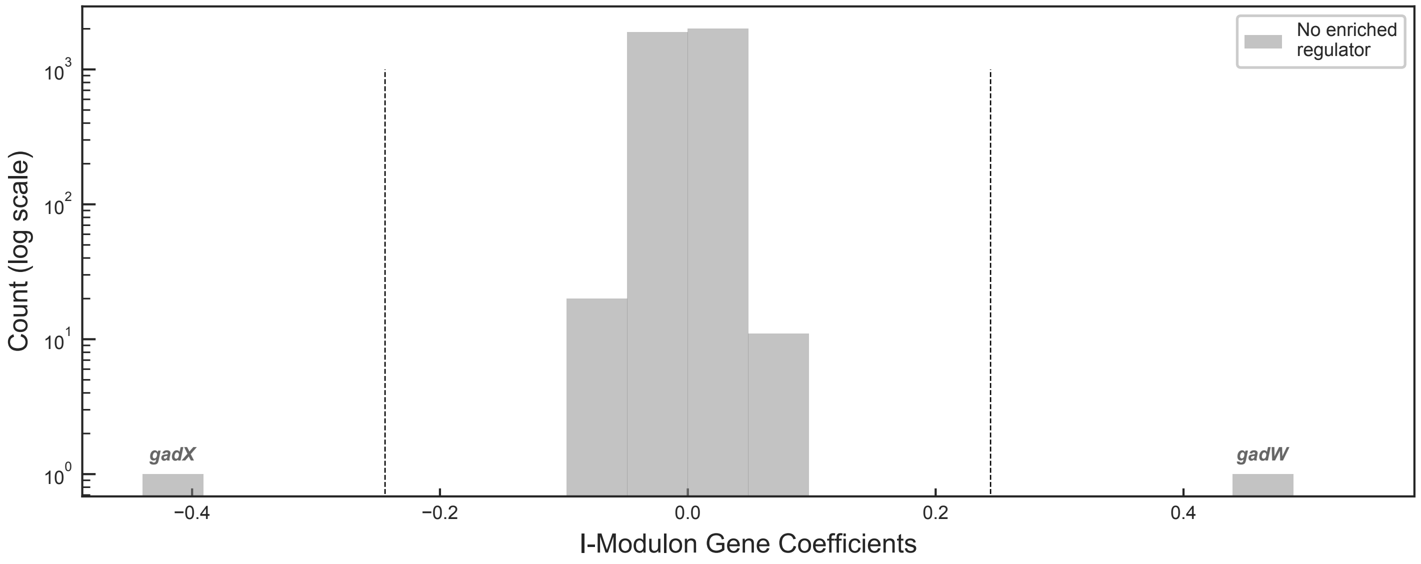

insertion I-Modulon

Biological Function: IS2 insertion element after laboratory evolution

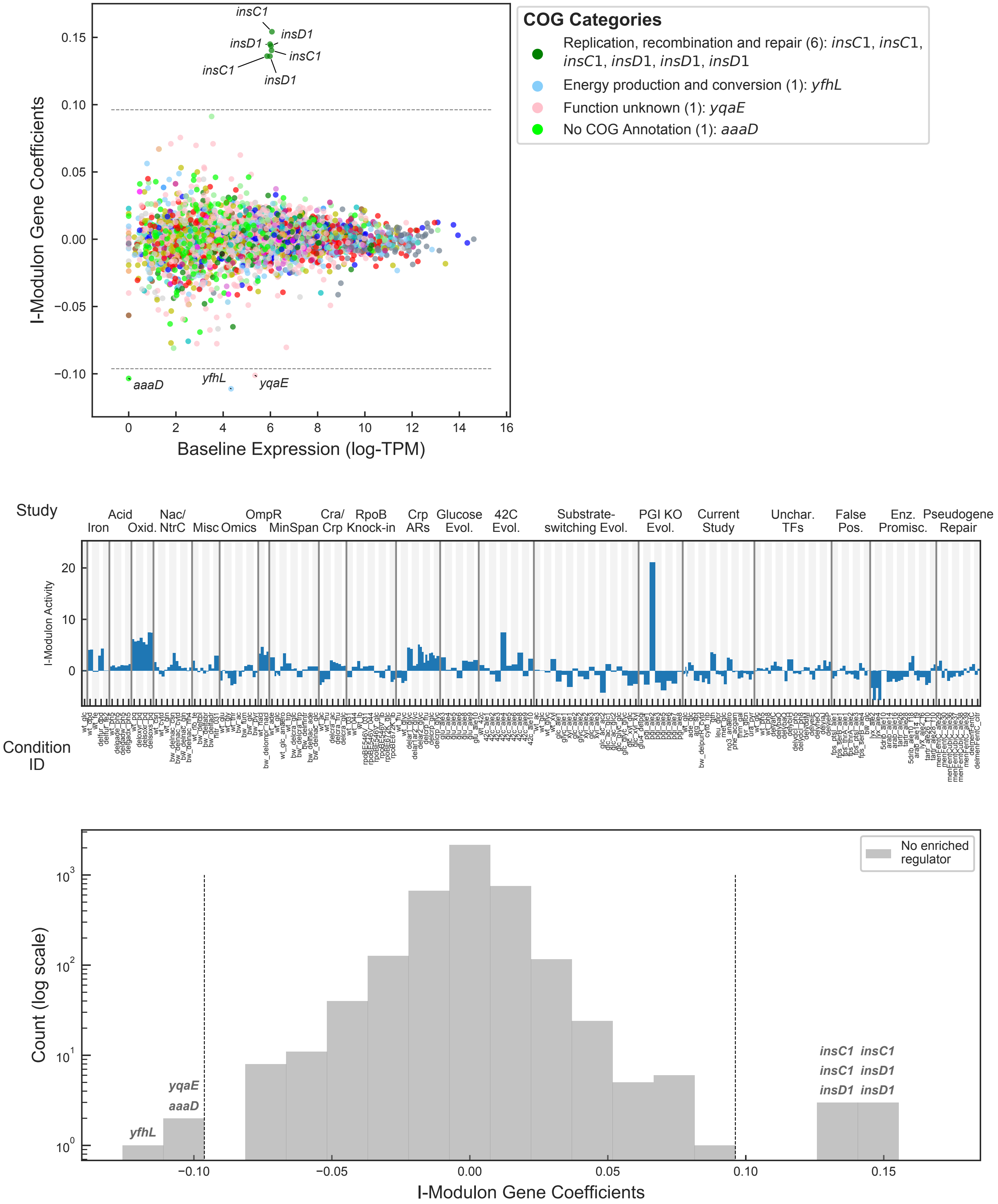

iron − related I-Modulon

Biological Function: Related to iron metabolism

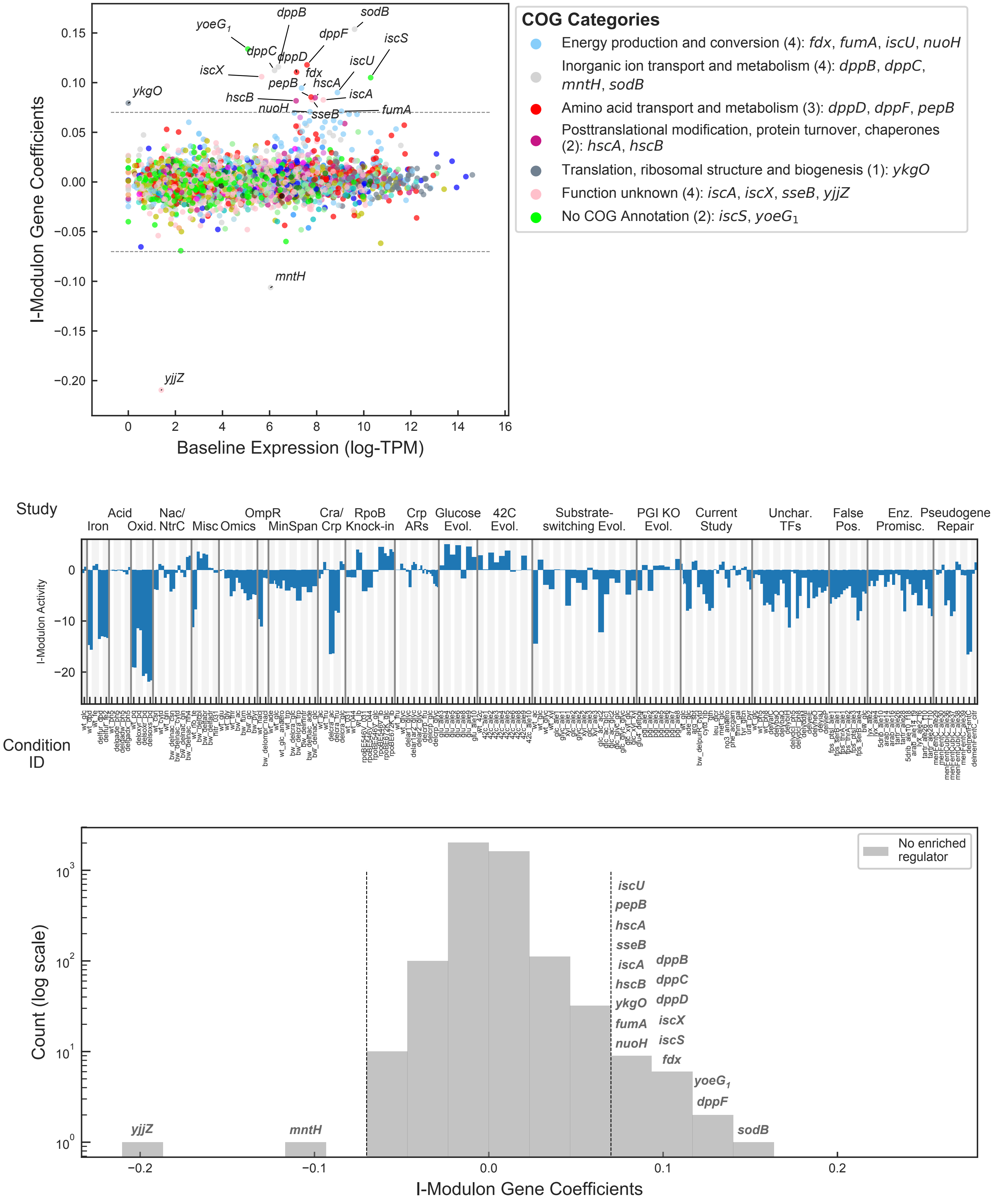

lipopolysaccharide I-Modulon

Biological Function: Lipopolysaccharide biosynthesis

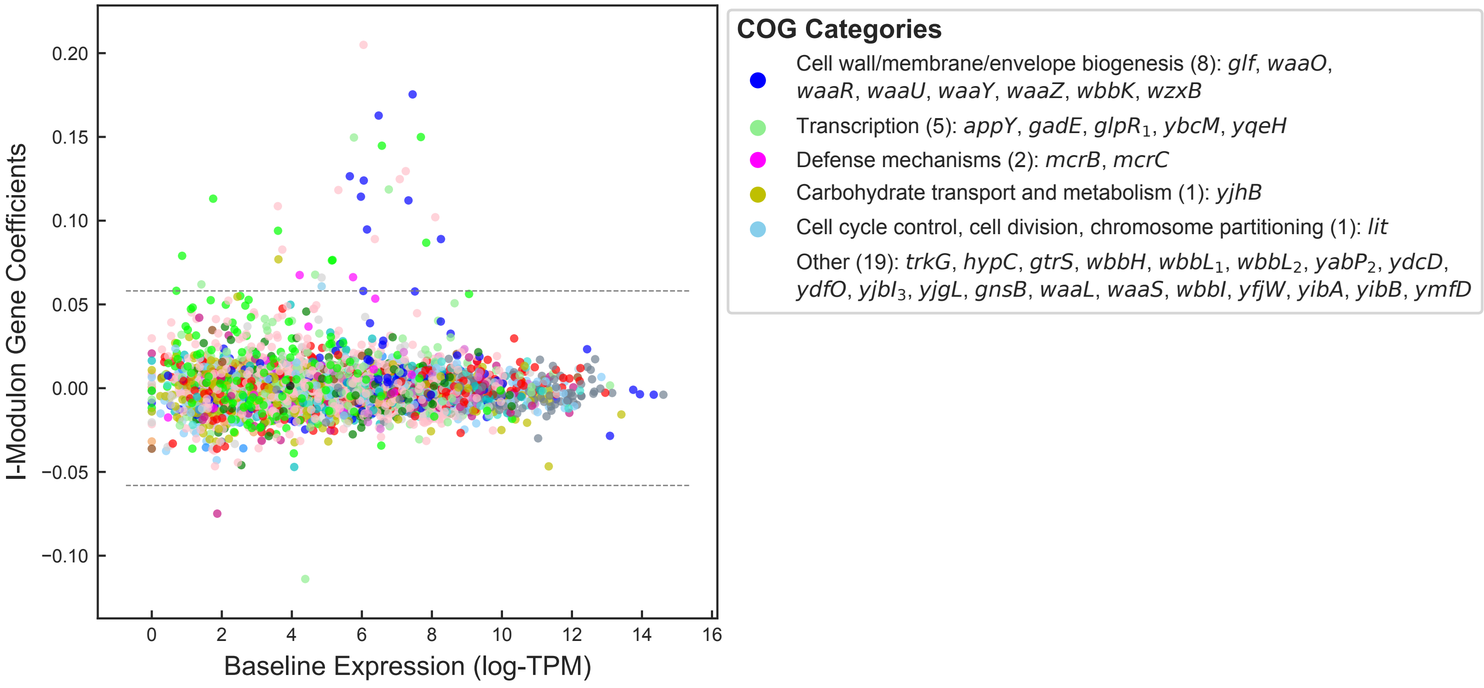

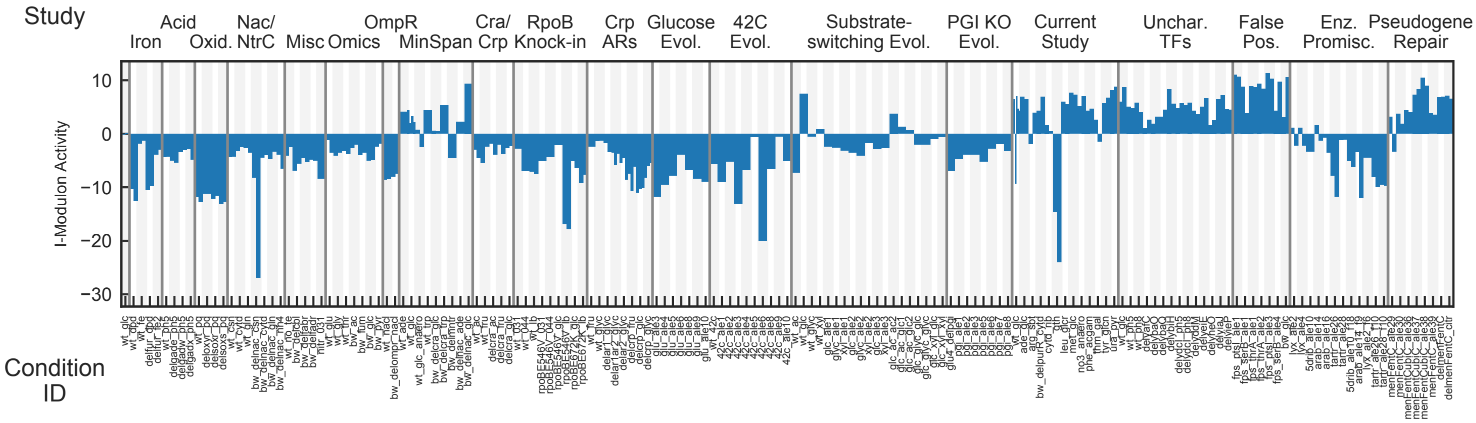

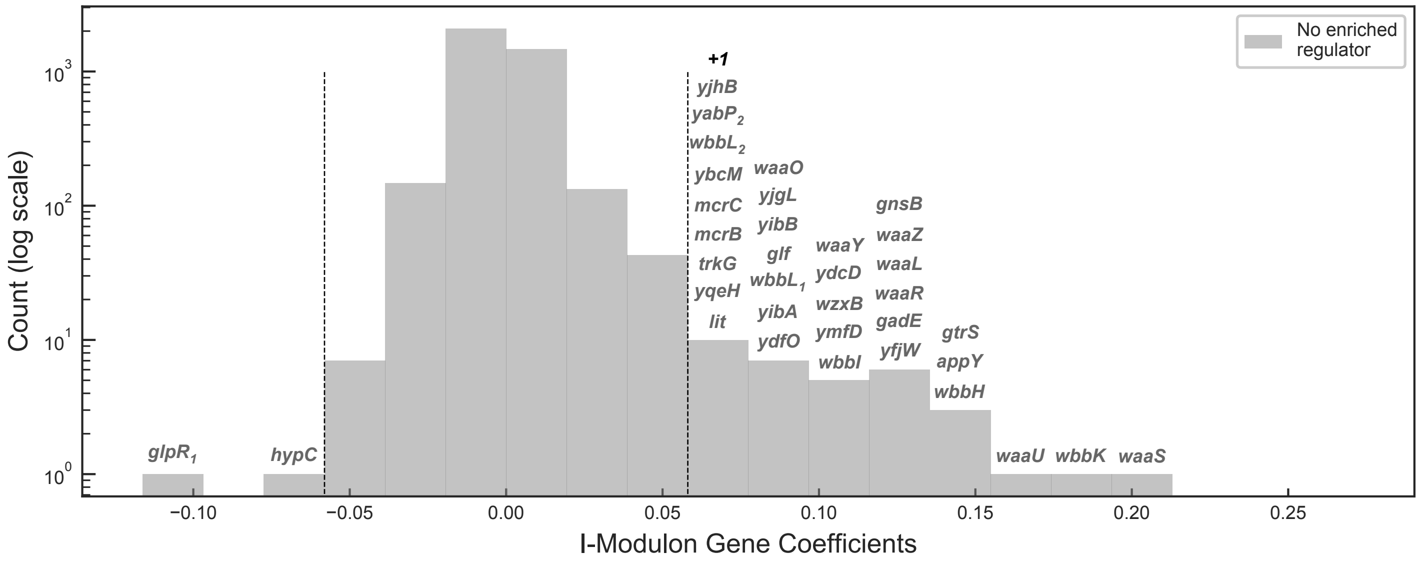

membrane I-Modulon

Biological Function: Enriched in membrane-bound proteins

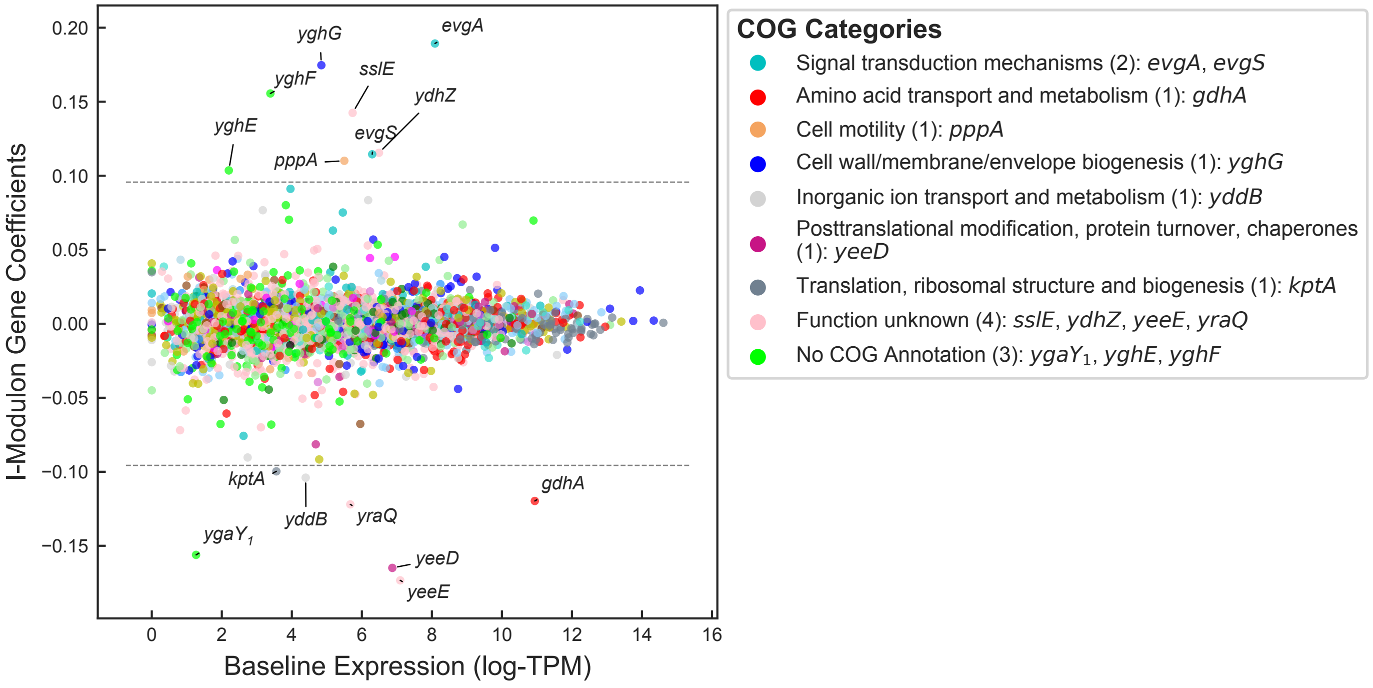

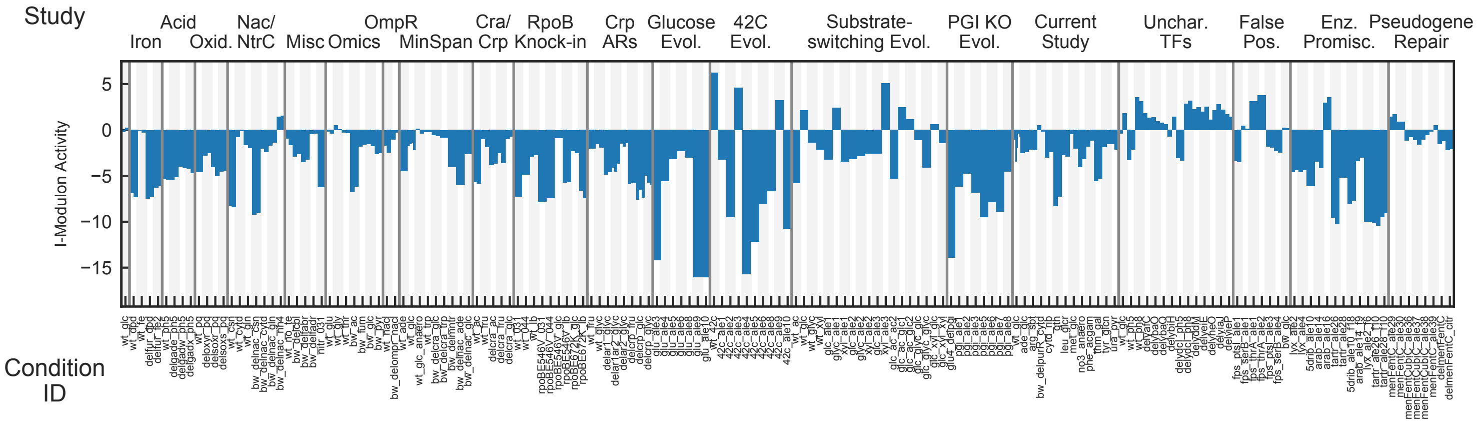

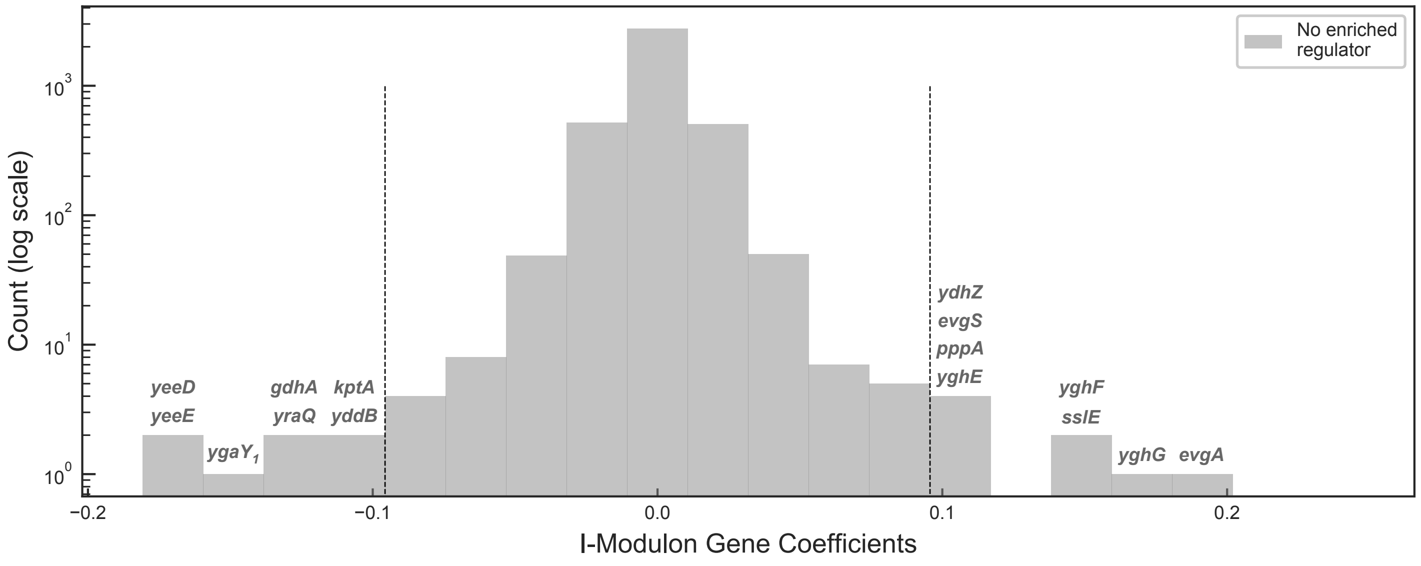

nitrate – related I-Modulon

Biological Function: Nitric oxide response

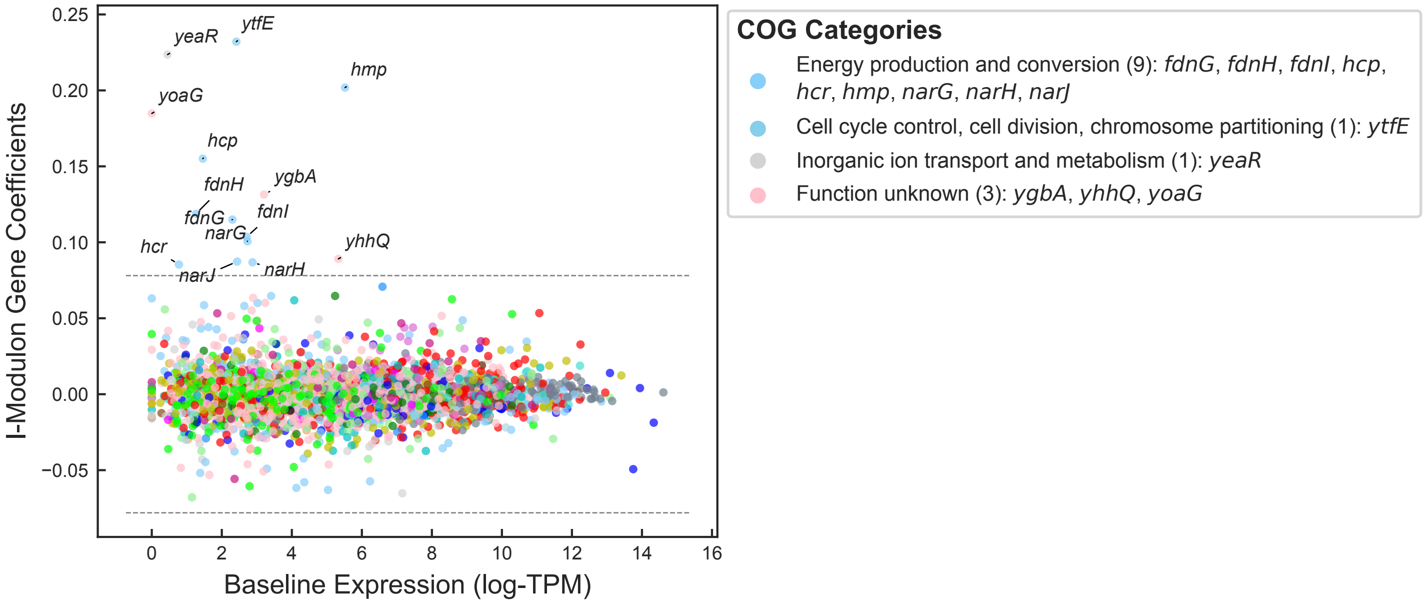

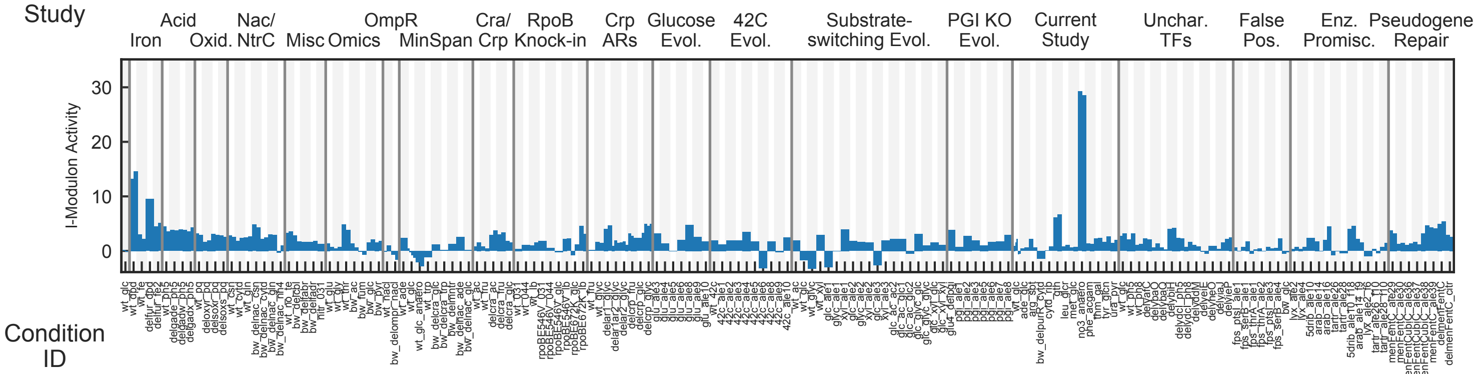

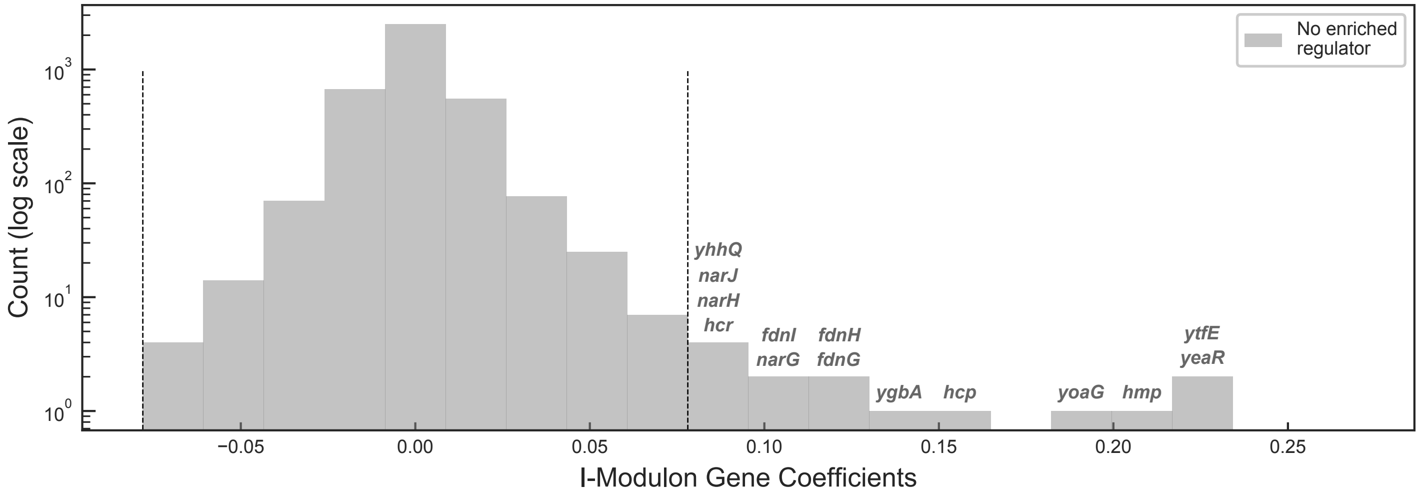

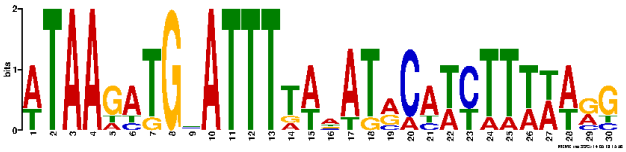

Motif E-value: 5.30e-05
Operons with Upstream Motif: 75%

proVWX I-Modulon

Biological Function: Glycine betaine transport

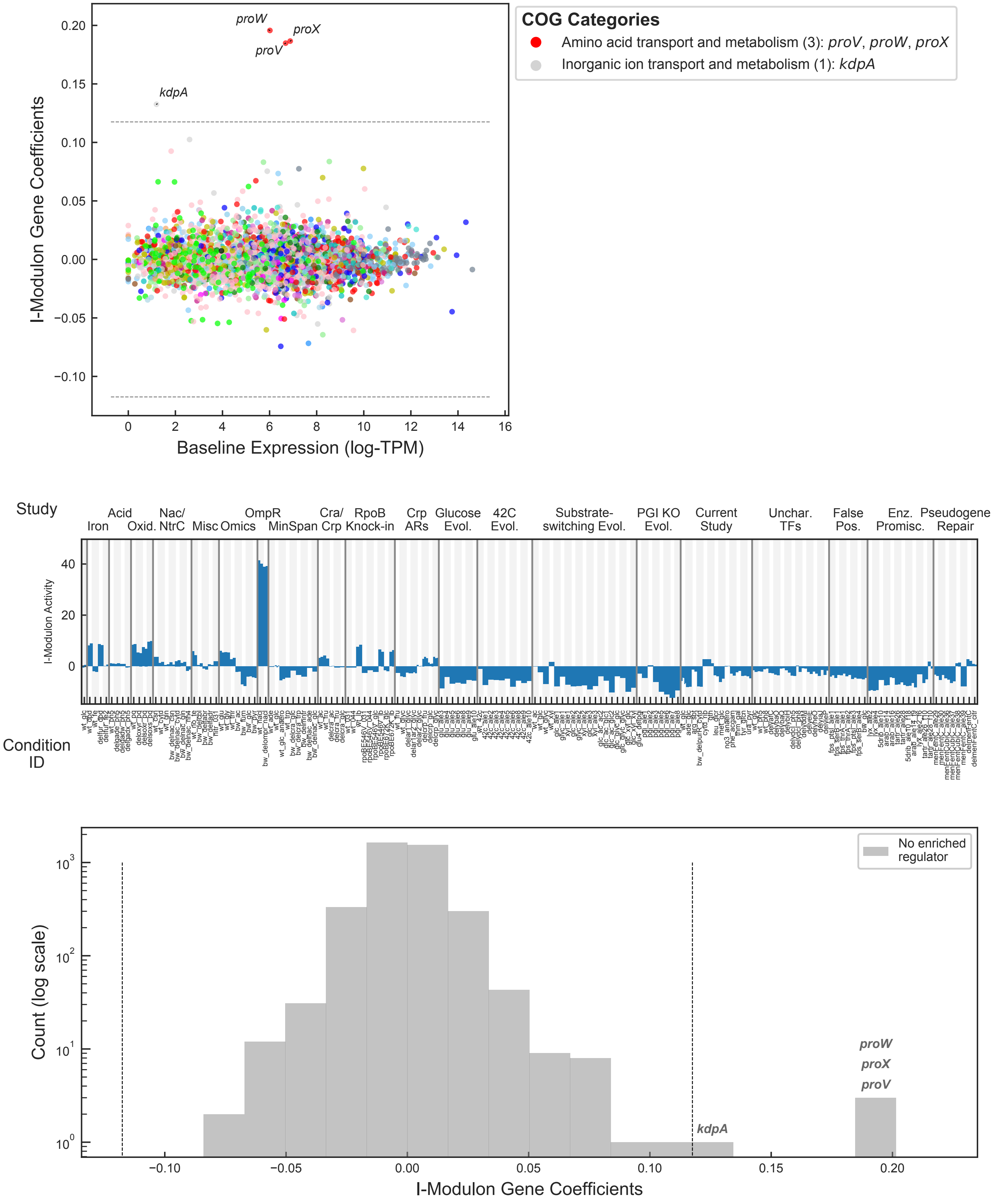

purR − KO I-Modulon

Biological Function: Accounts for purR knock-out

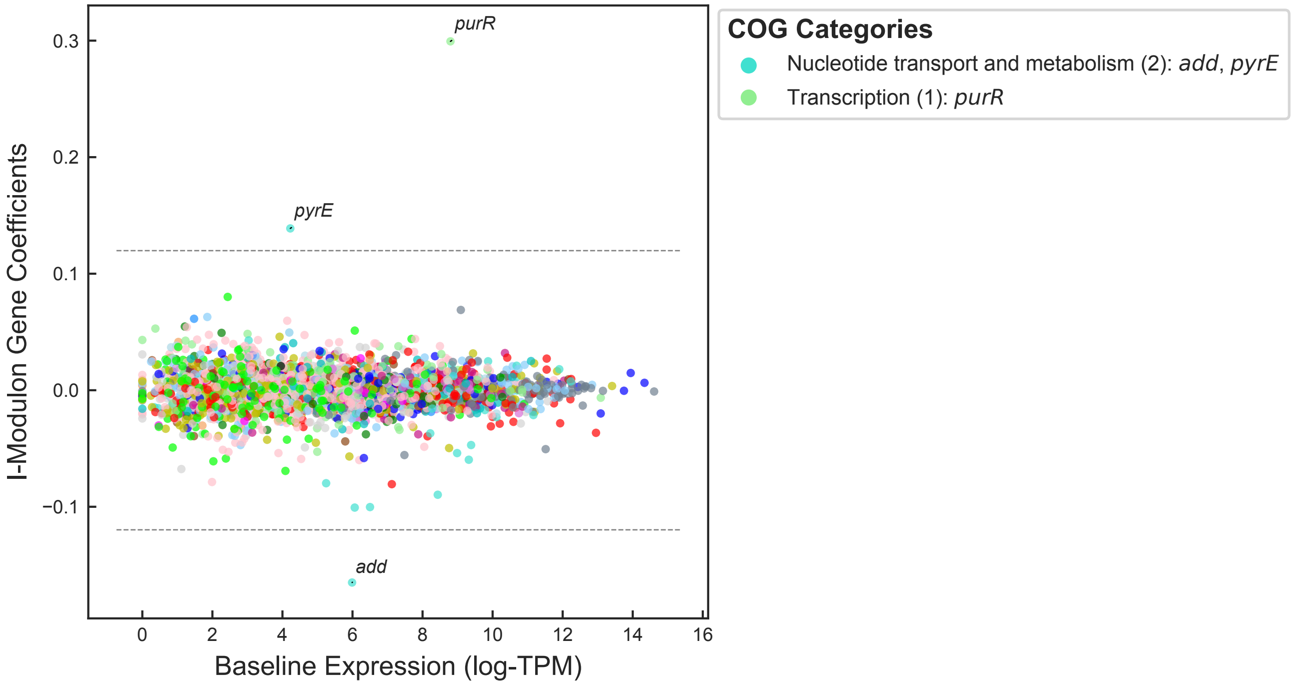

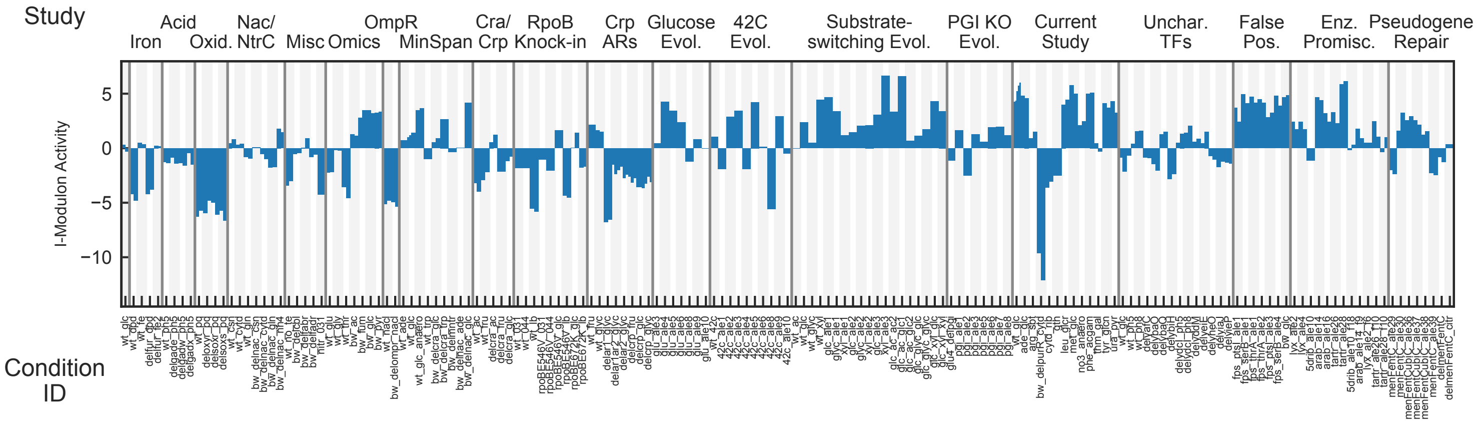

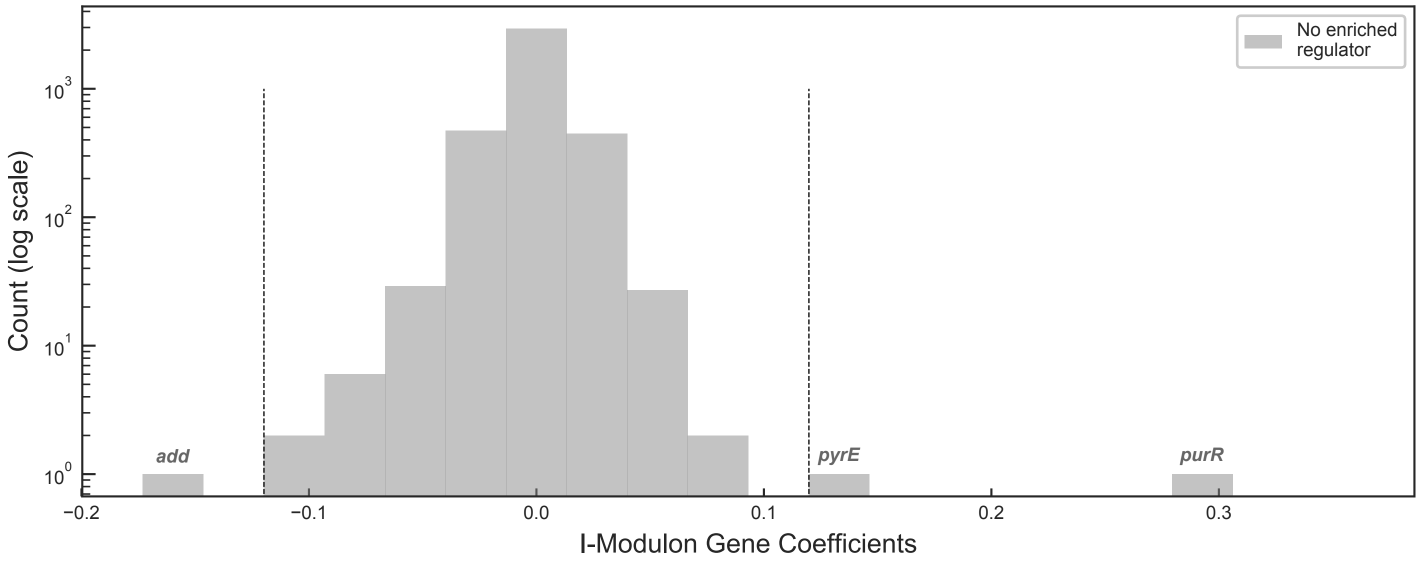

sgrT I-Modulon

Biological Function: Contains single dominating gene: sgrT

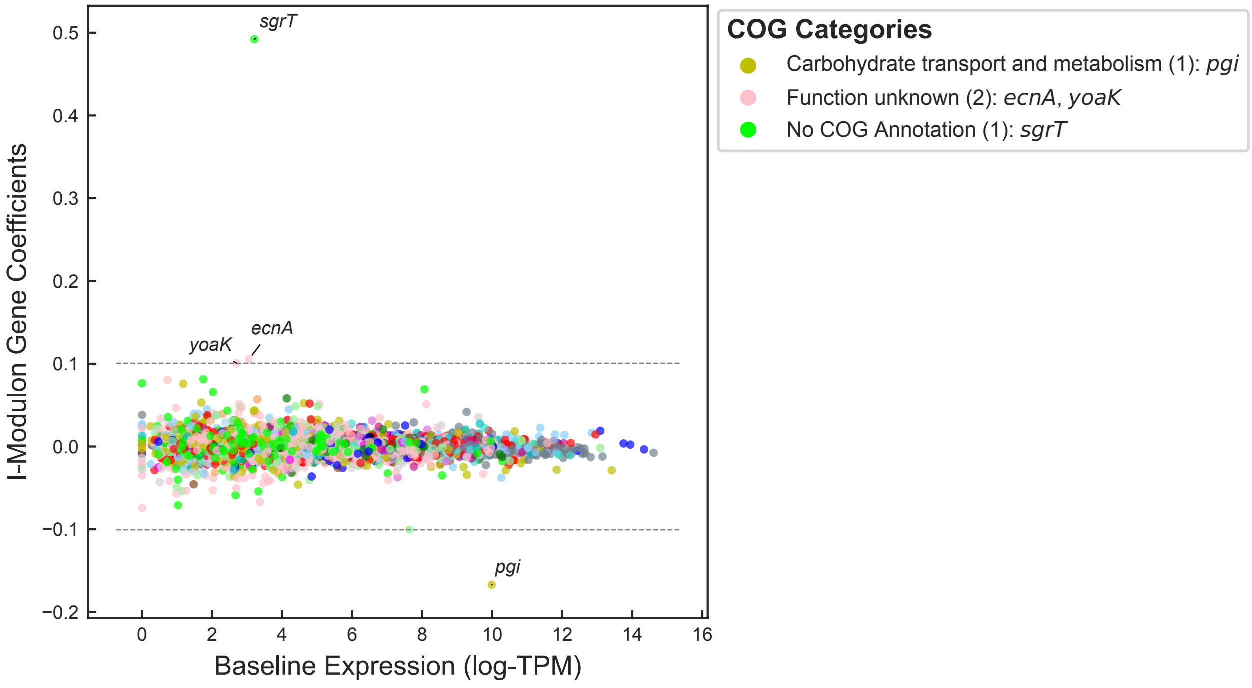

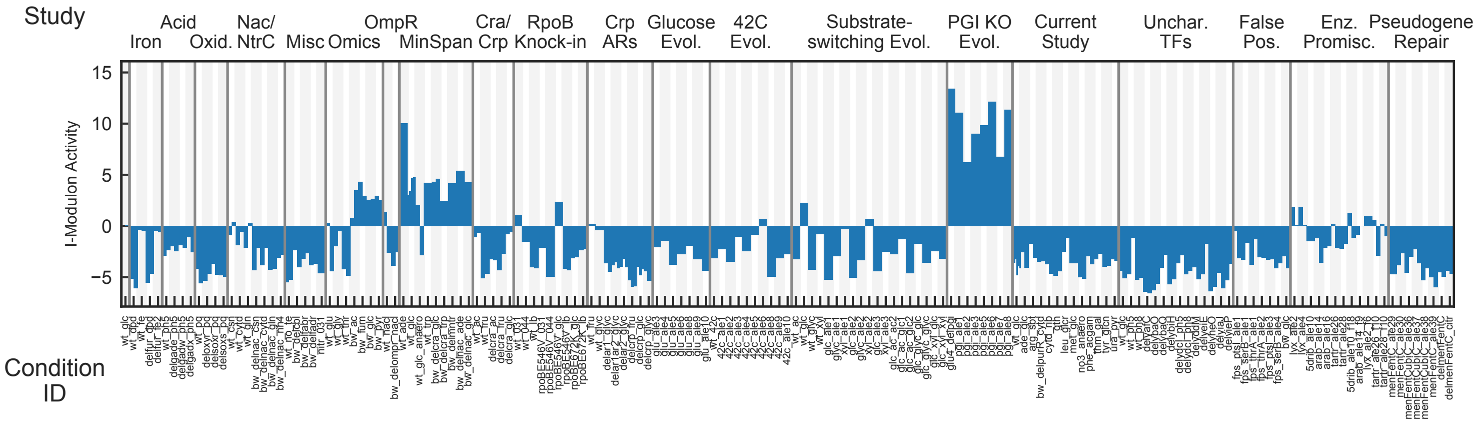

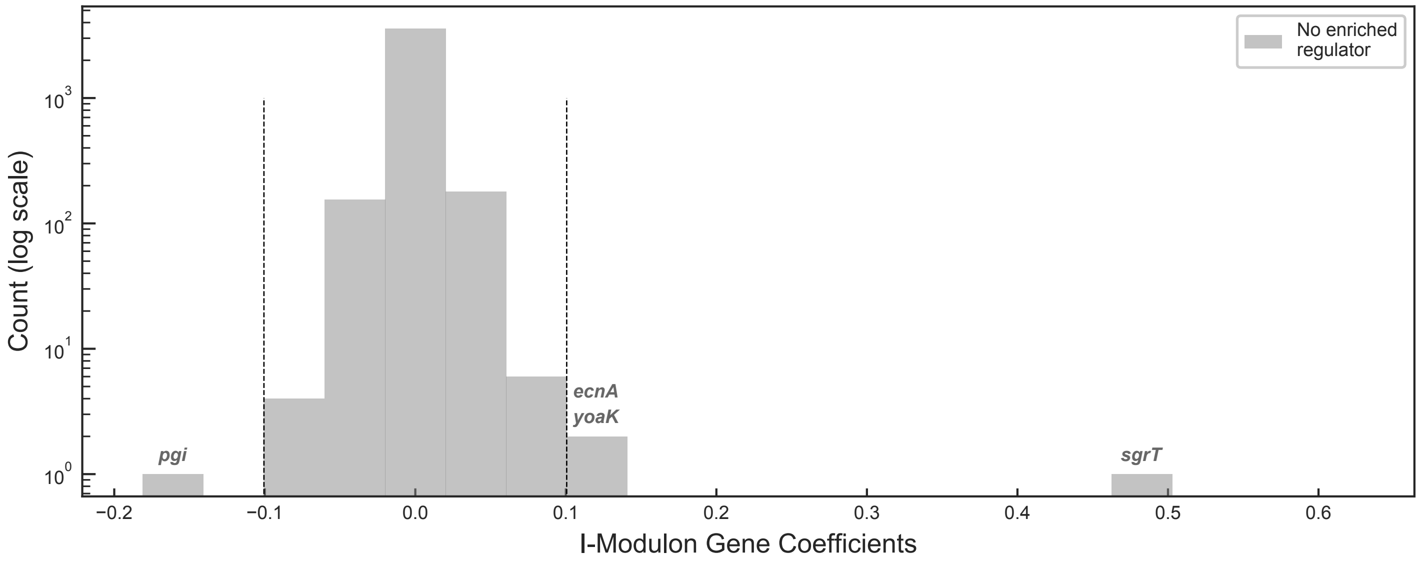

thrA − KO I-Modulon

Biological Function: Accounts for thrA knock-out

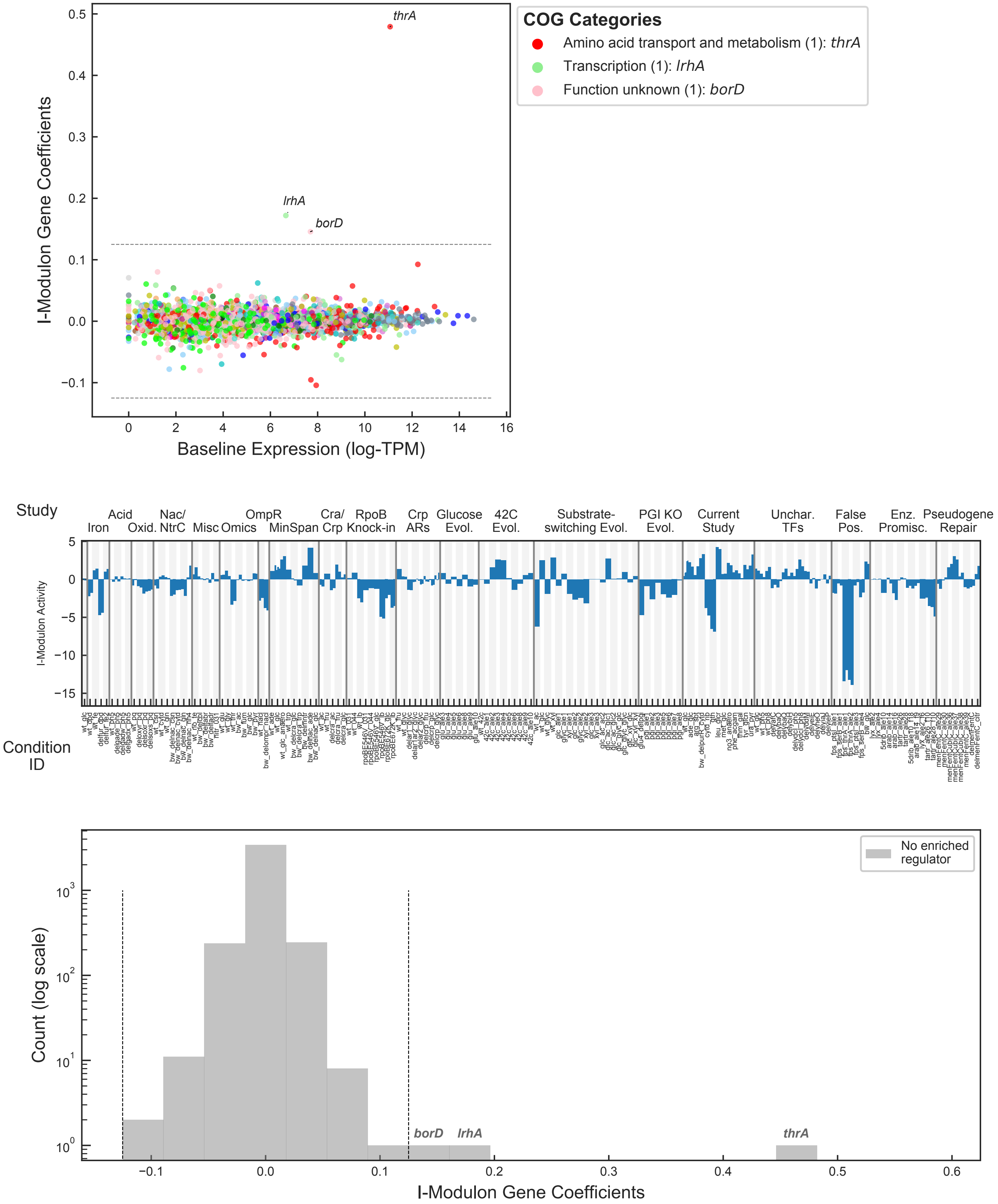

translation I-Modulon

Biological Function: Enriched in translation machinery

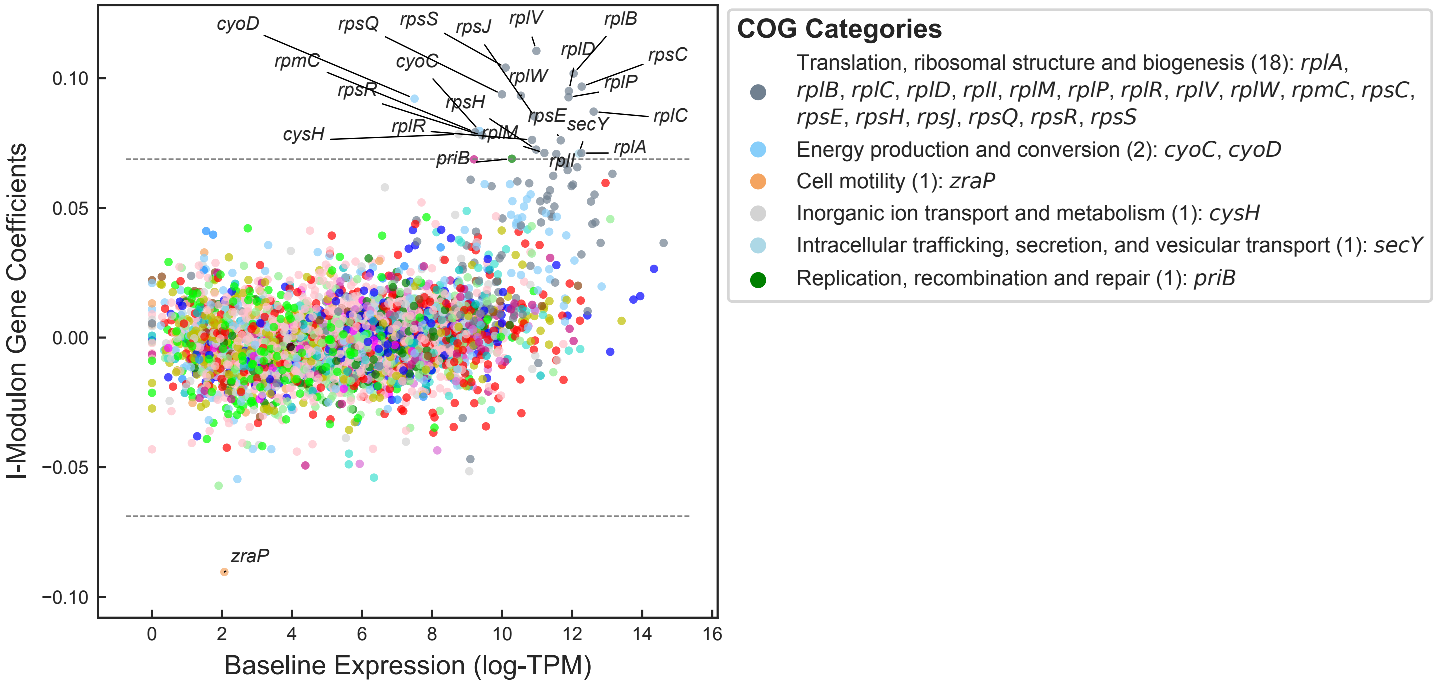

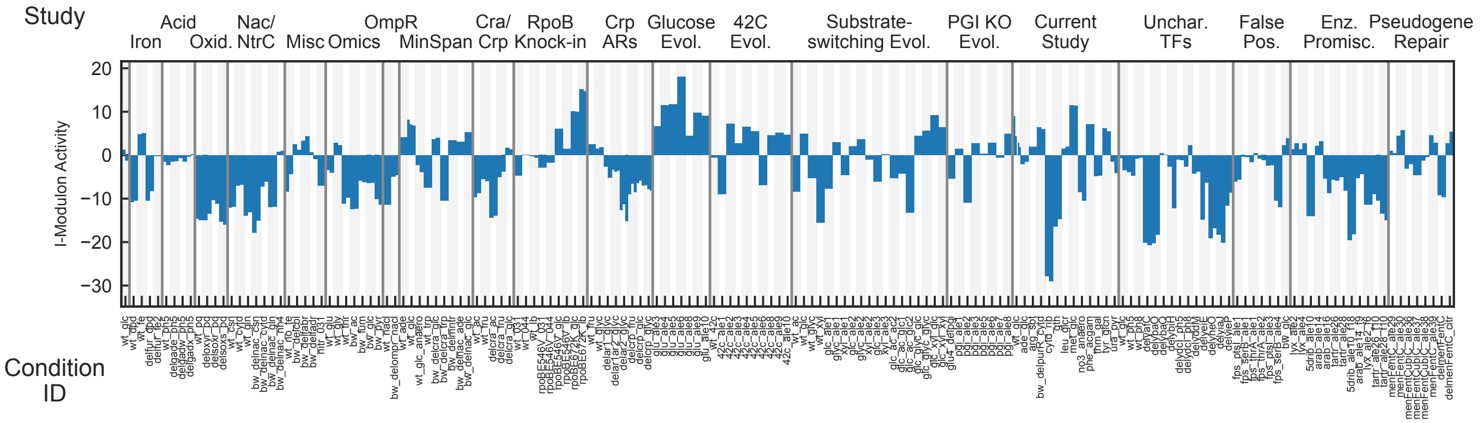

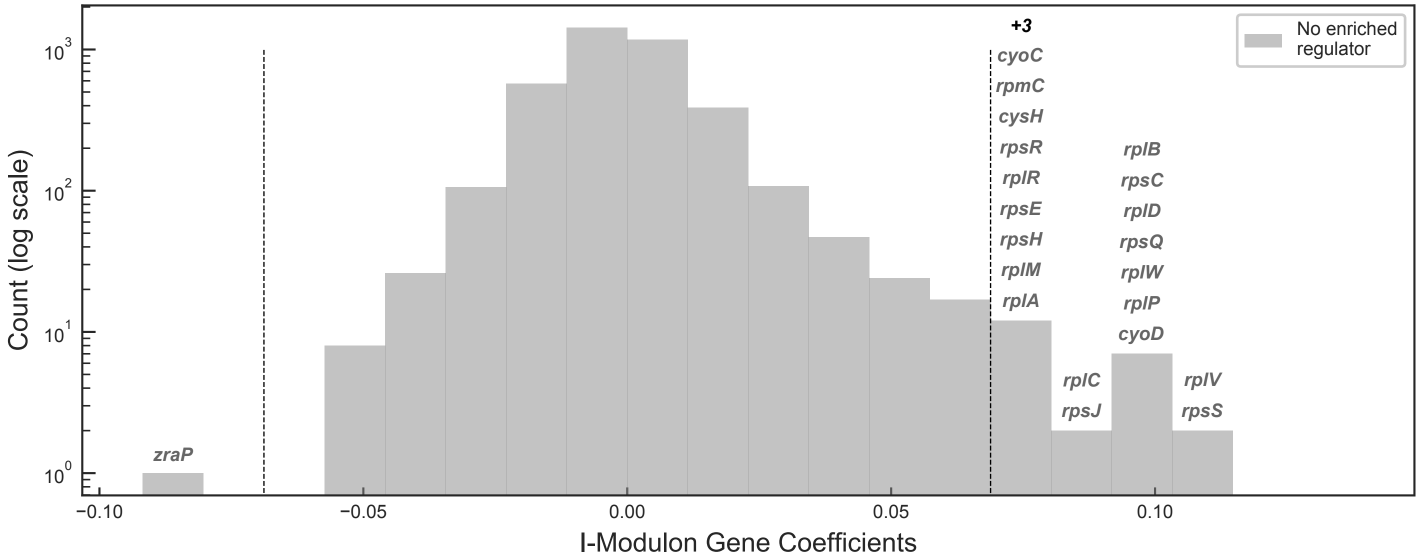

ydcI − KO I-Modulon

Biological Function: Accounts for ydcI knock-out

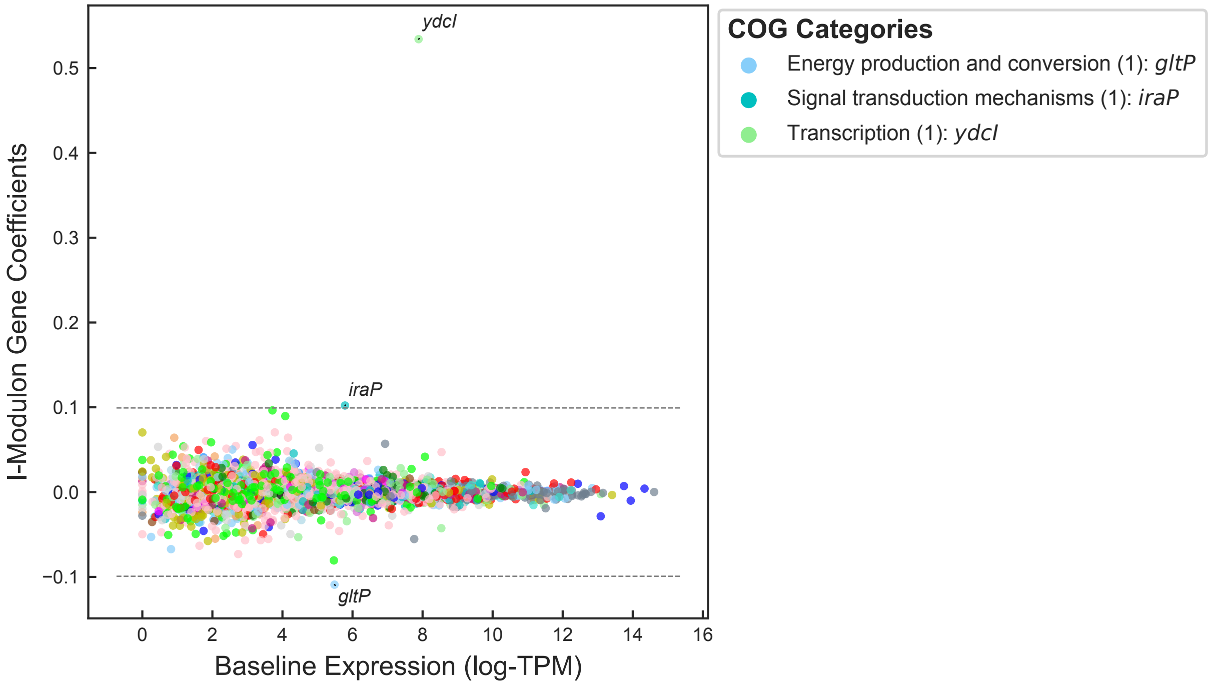

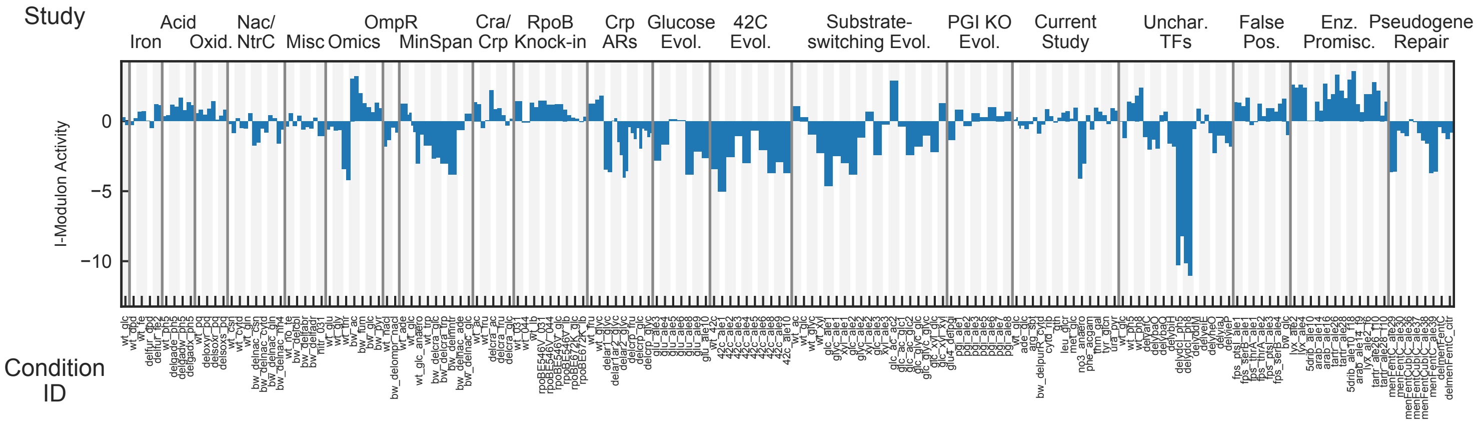

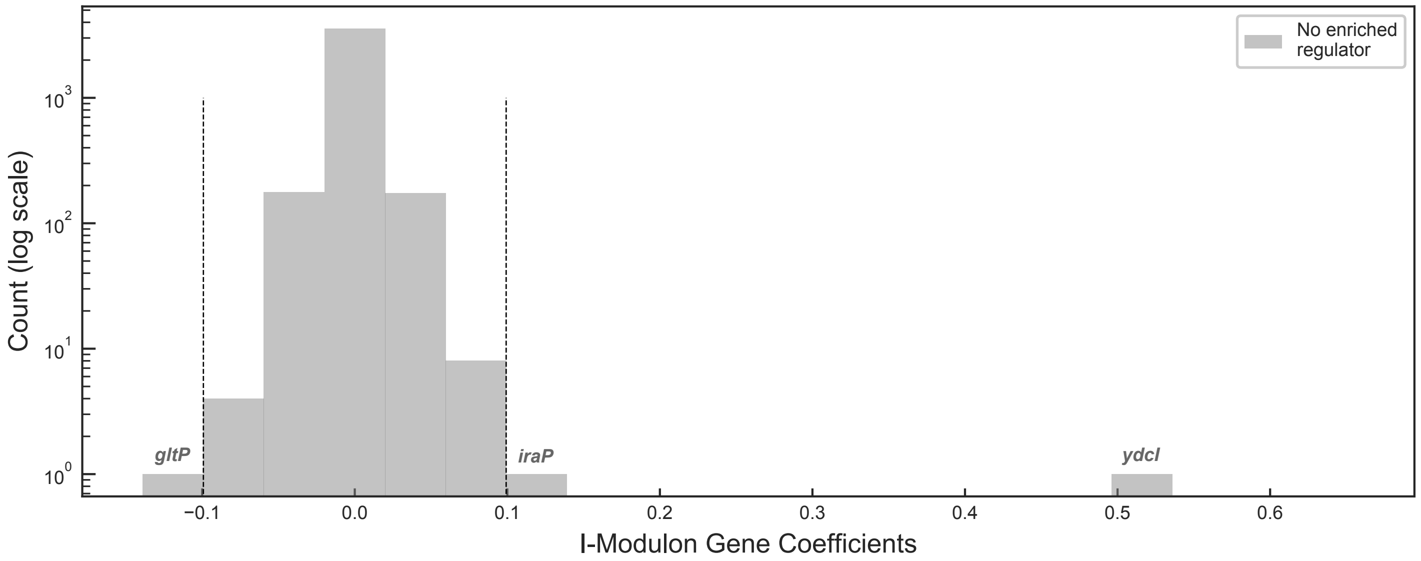

yheO − KO I-Modulon

Biological Function: Accounts for yheO knock-out

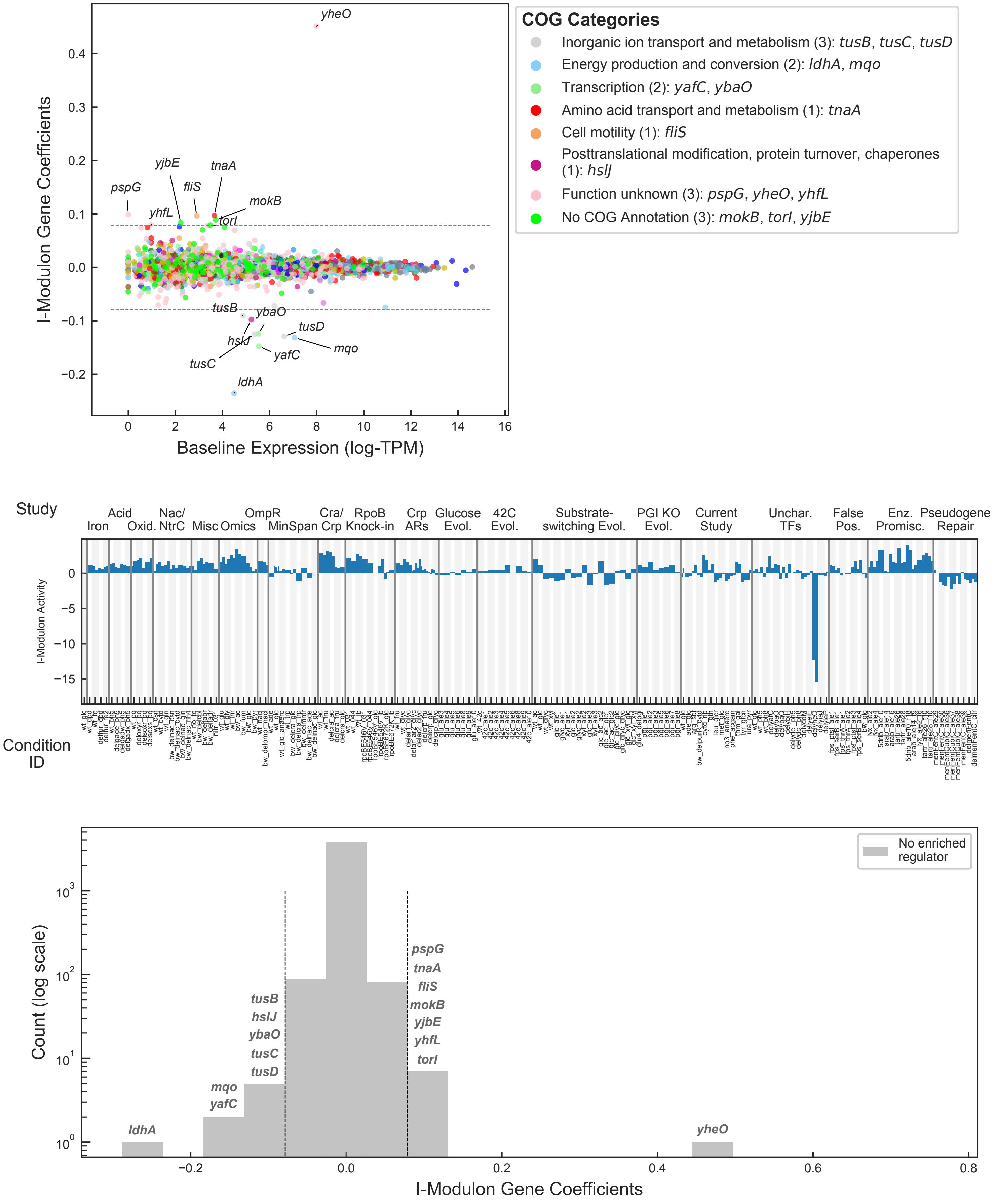

Uncharacterized I-Modulon #1

Biological Function: Unknown Function

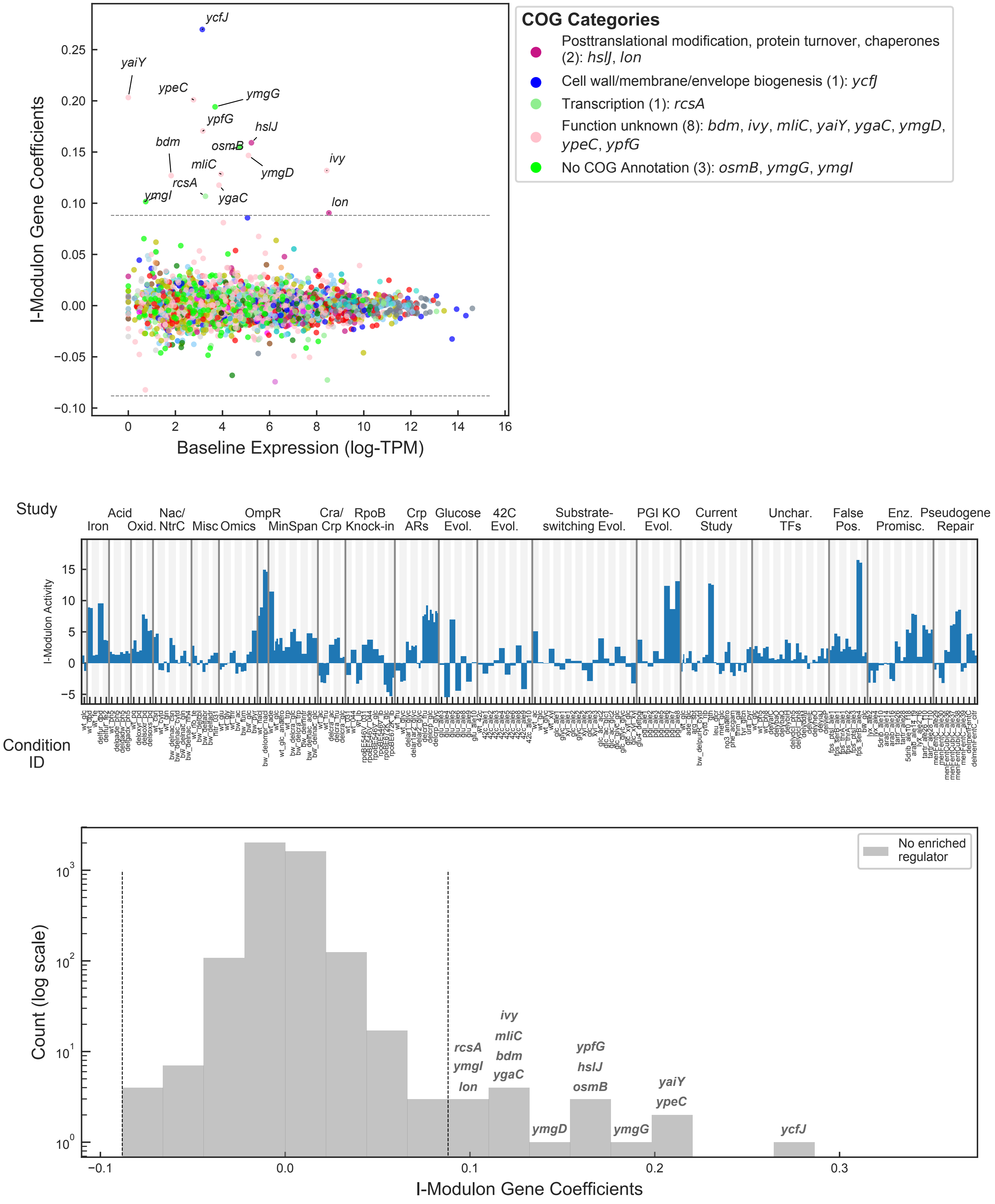

Uncharacterized I-Modulon #2

Biological Function: Unknown Function

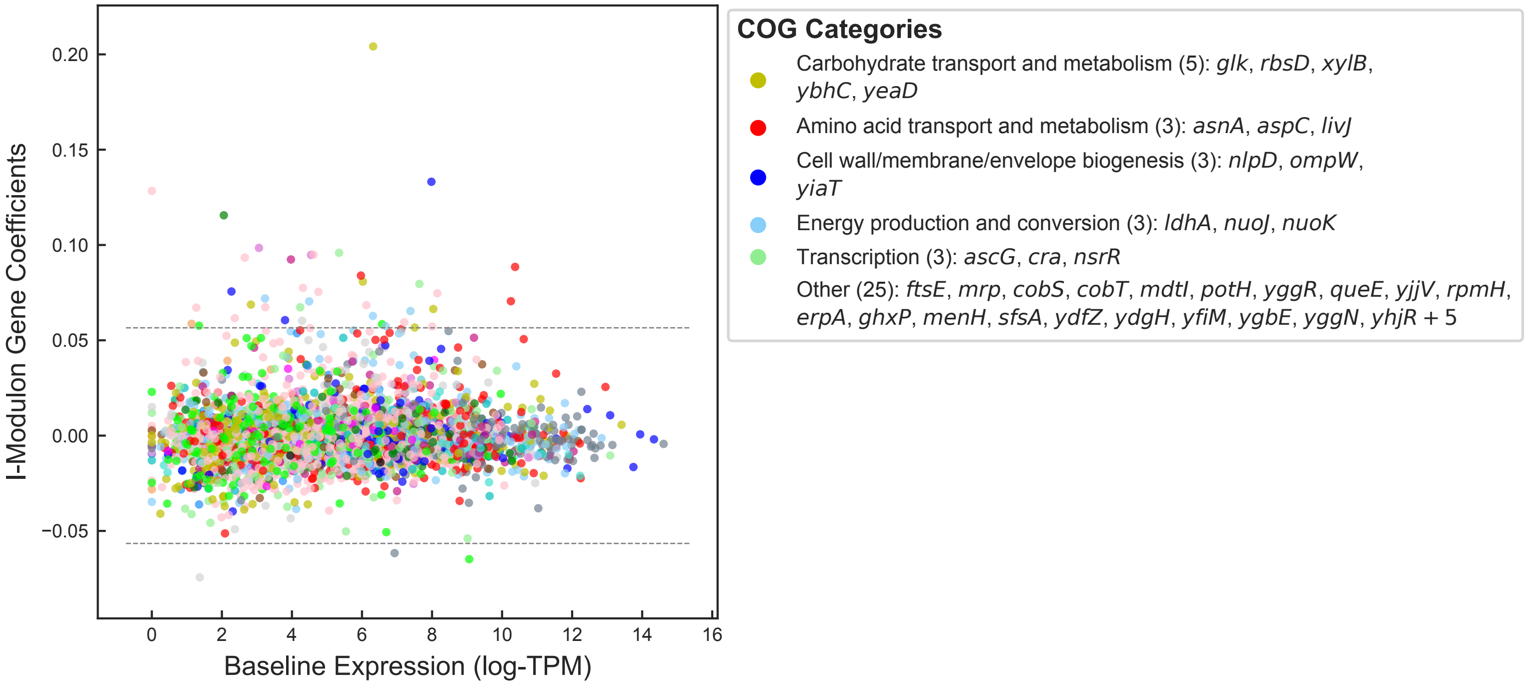

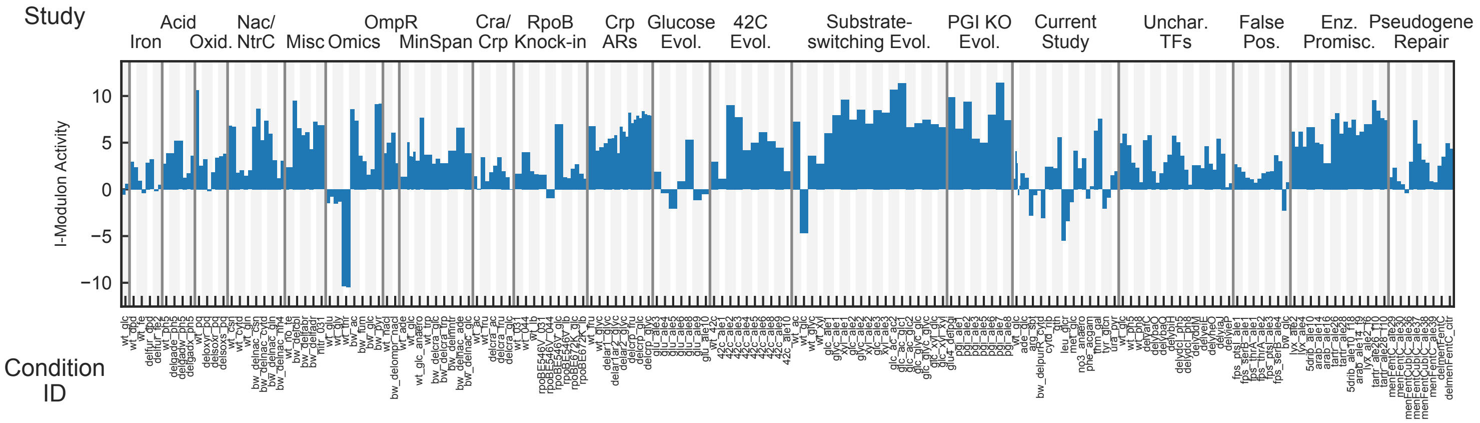

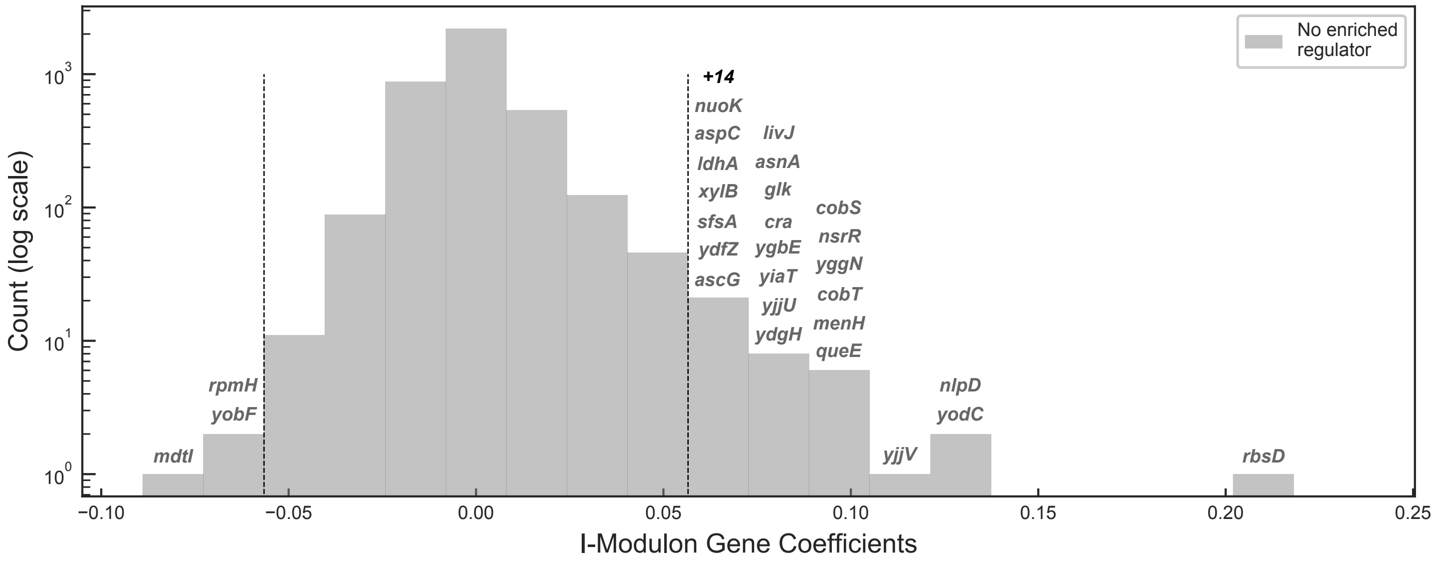

Uncharacterized I-Modulon #3

Biological Function: Unknown Function

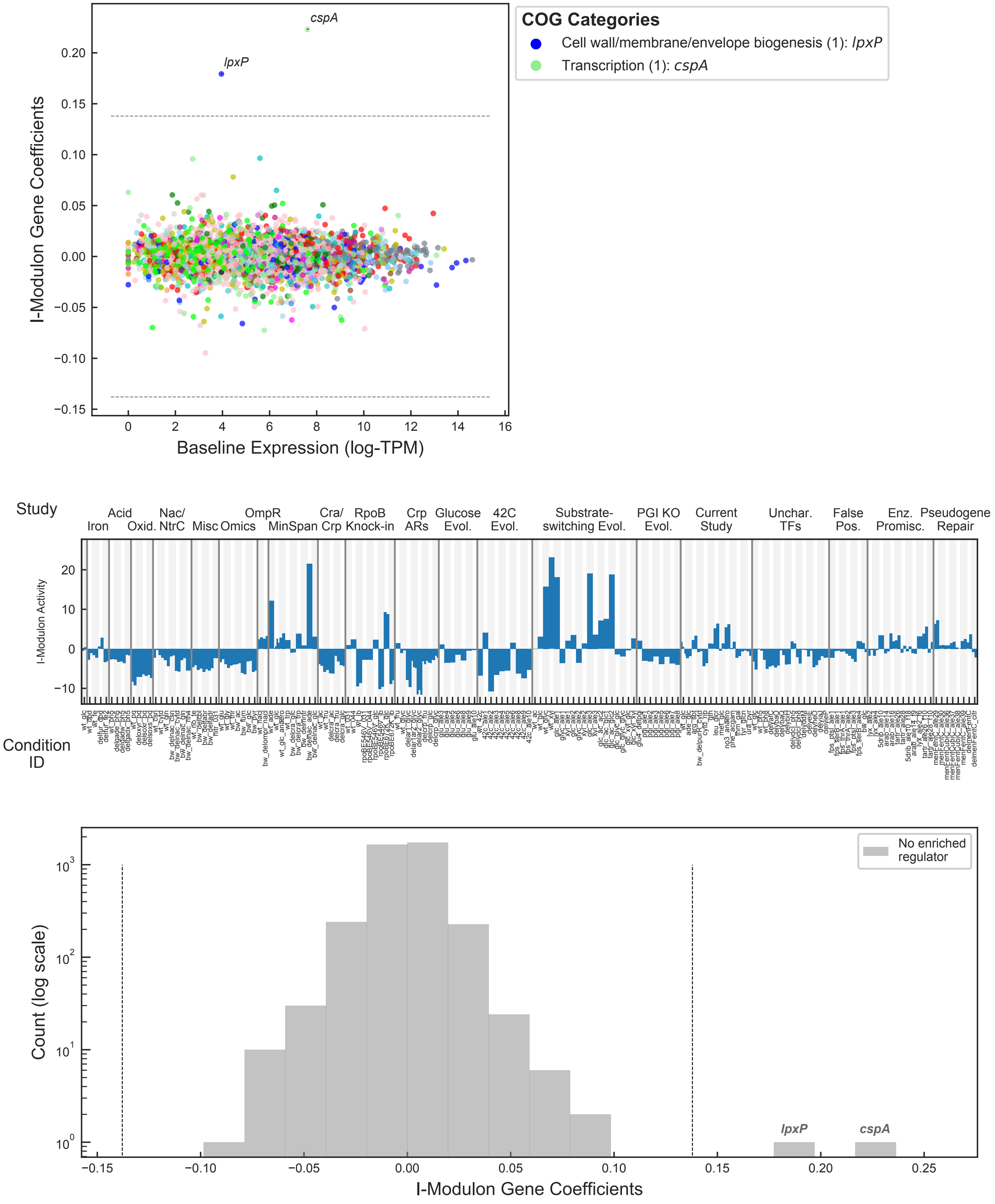

Uncharacterized I-Modulon #4

Biological Function: Unknown Function

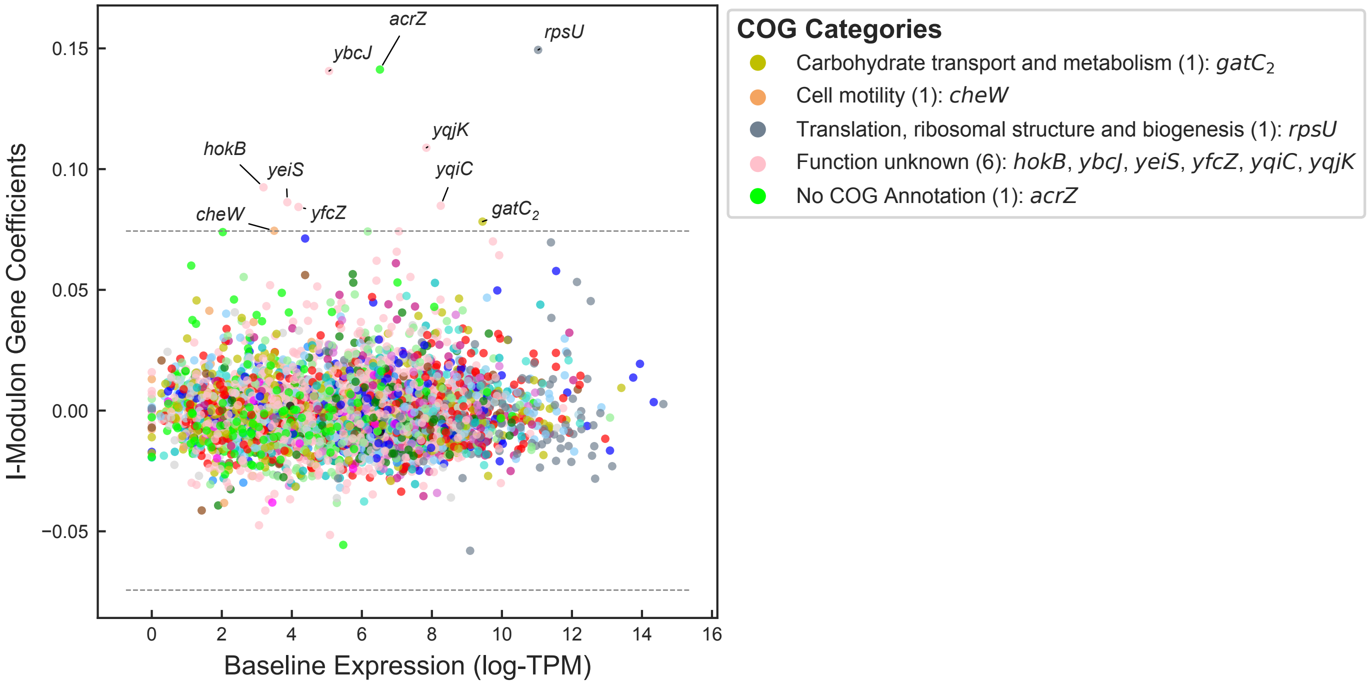

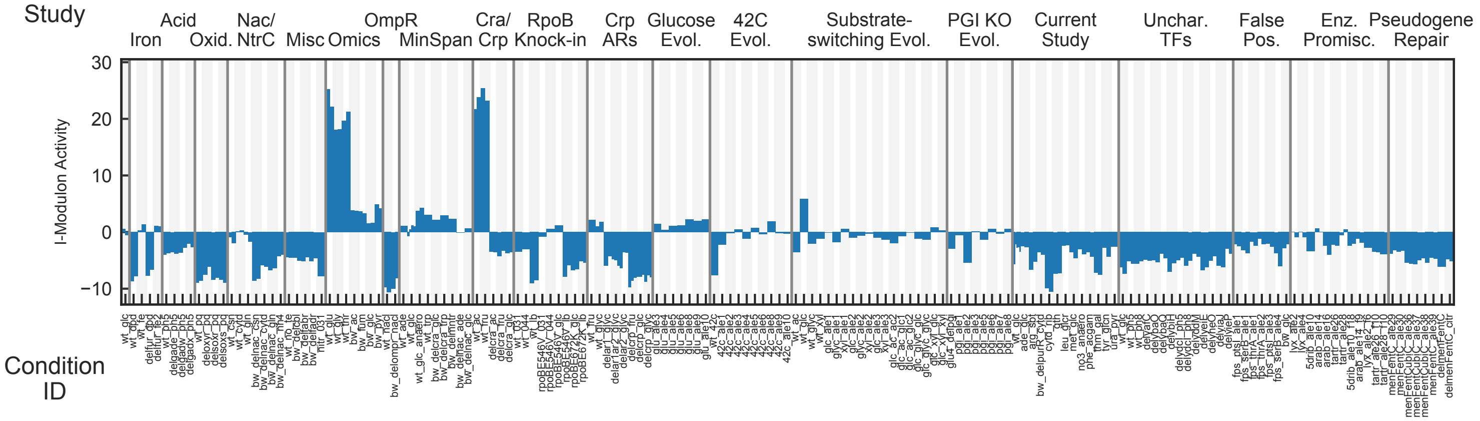

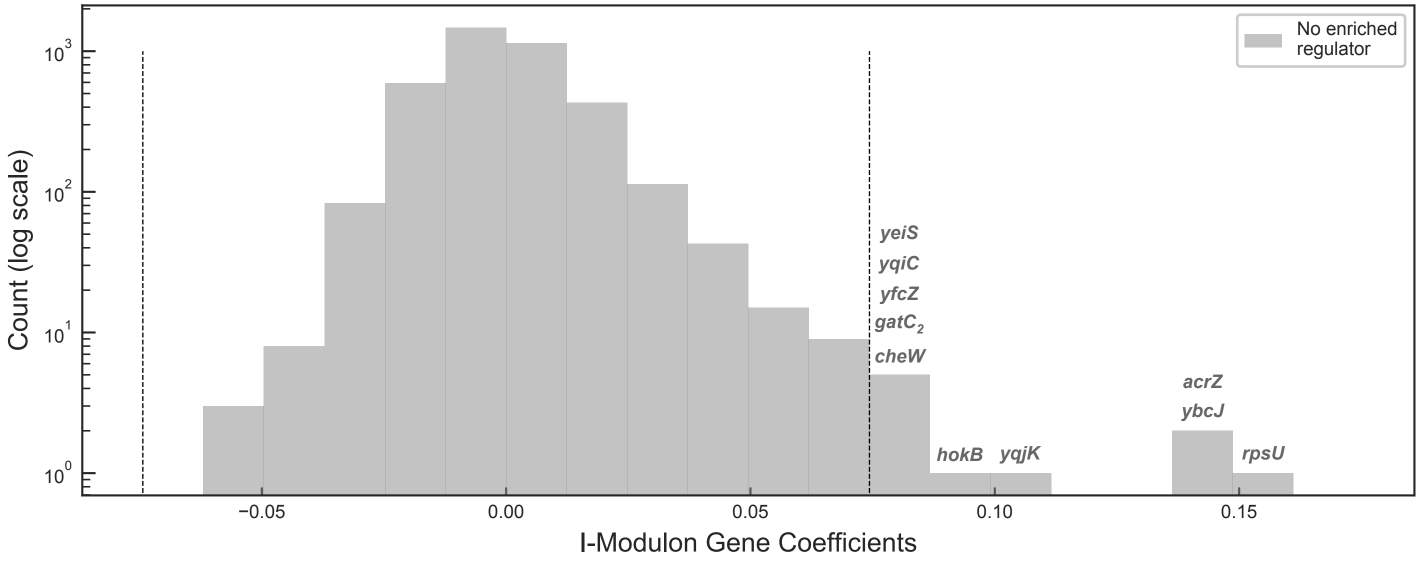

Uncharacterized I-Modulon #5

Biological Function: Unknown Function

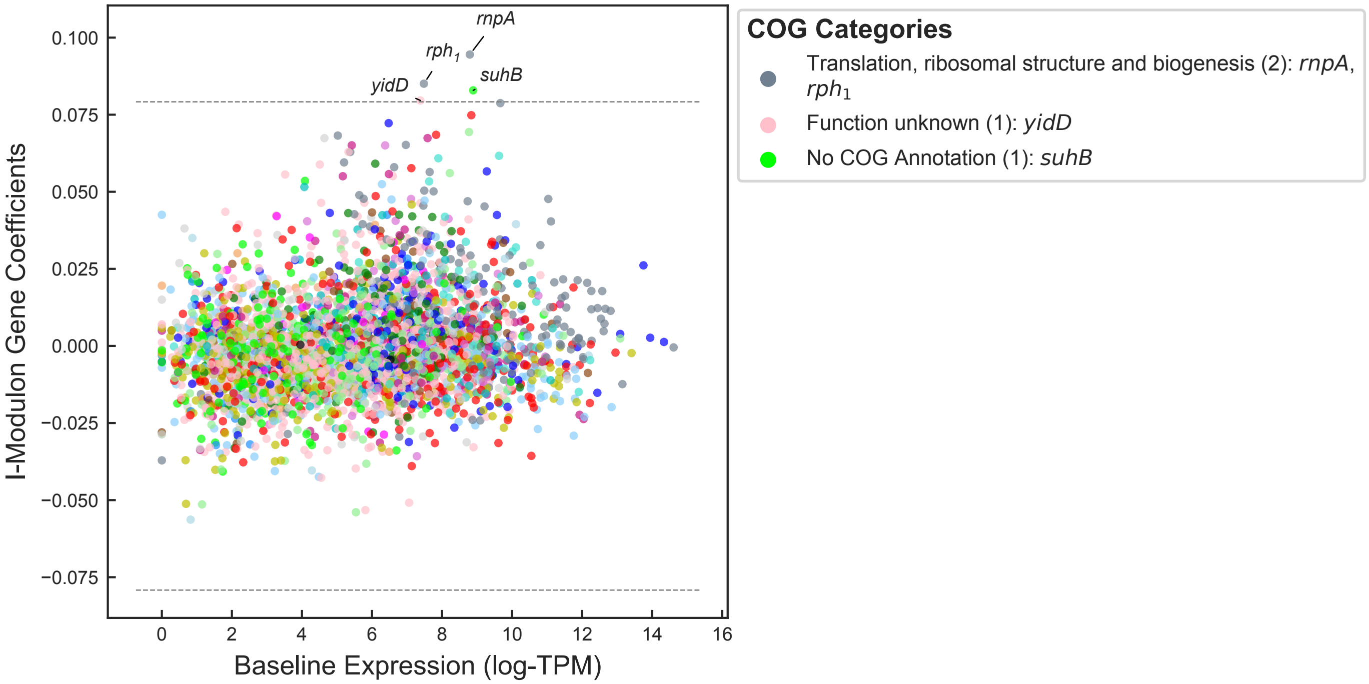

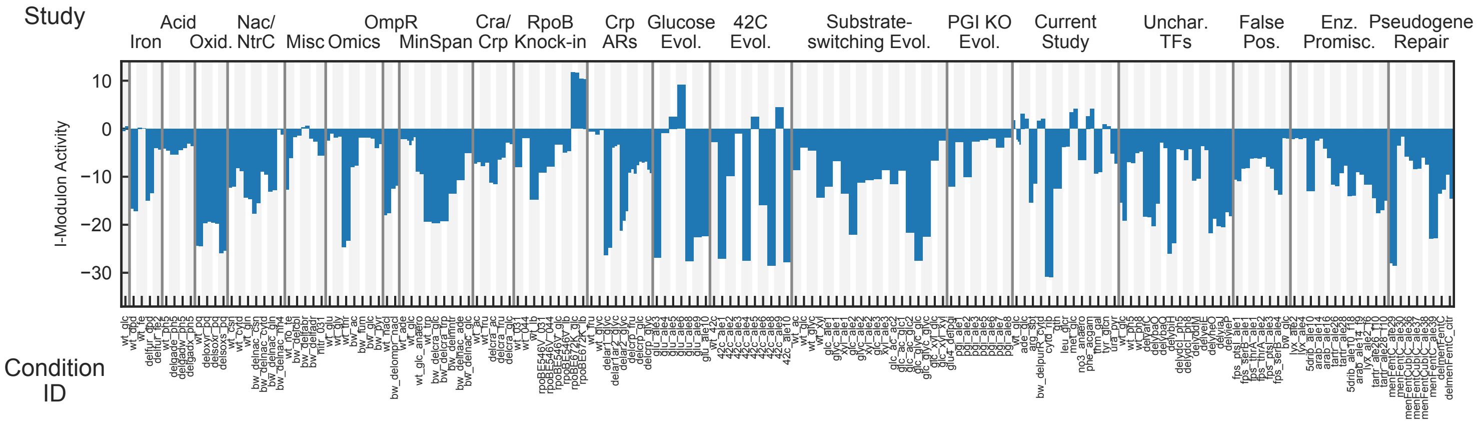

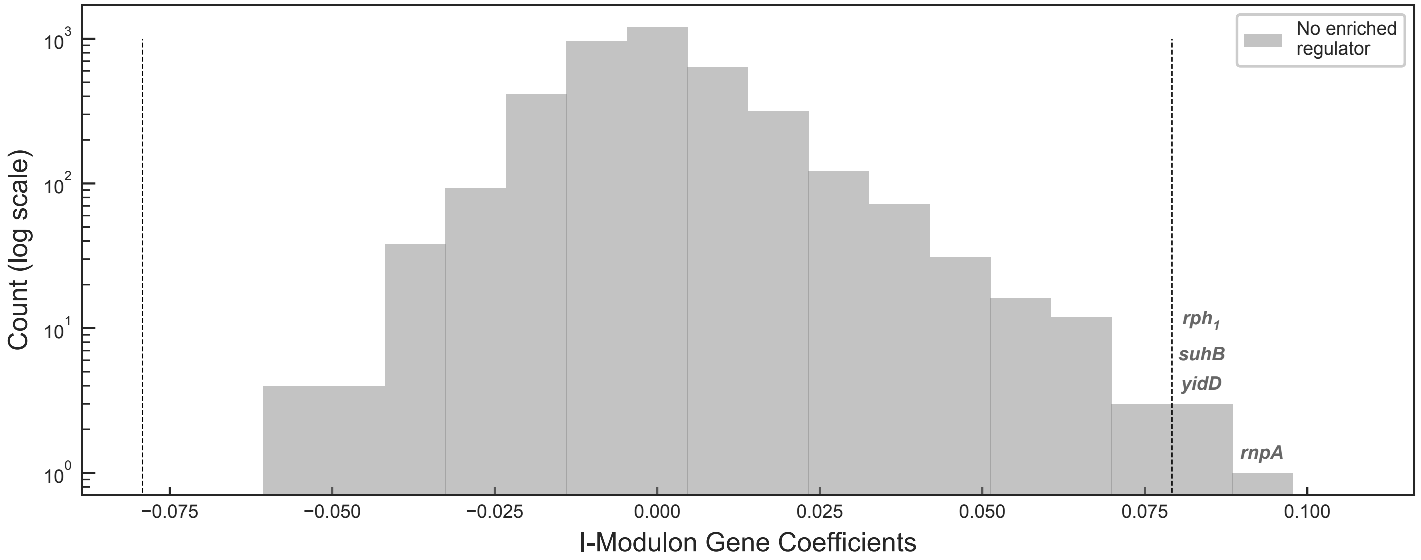

Uncharacterized I-Modulon #6

Biological Function: Unknown Function

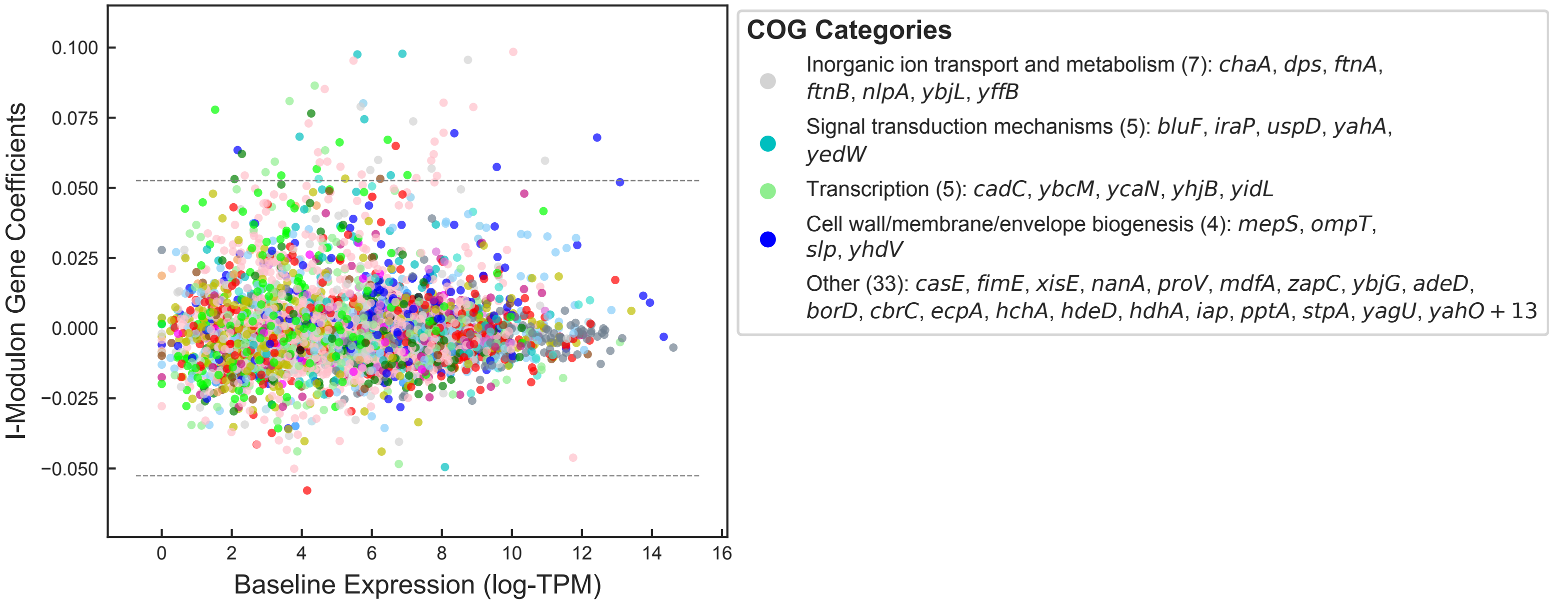

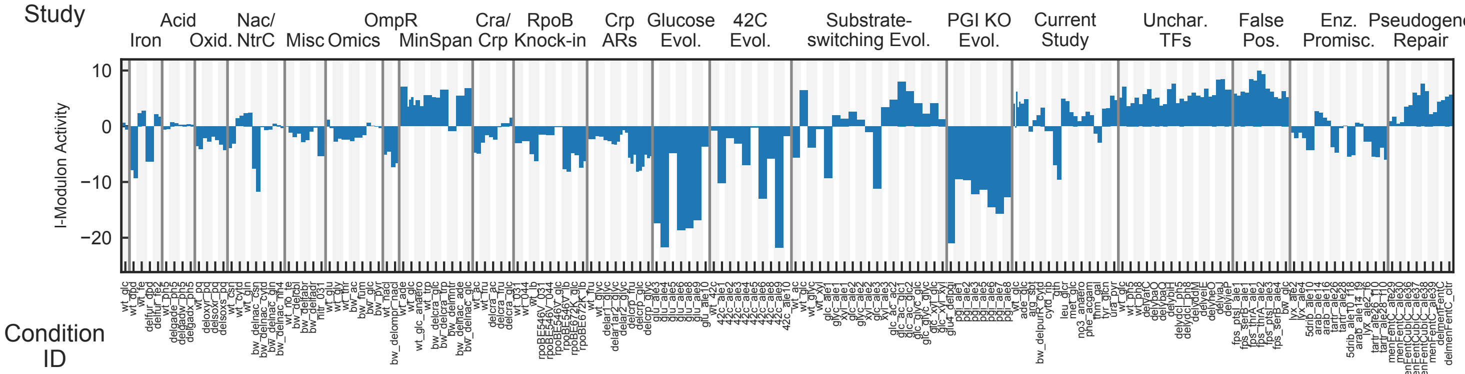

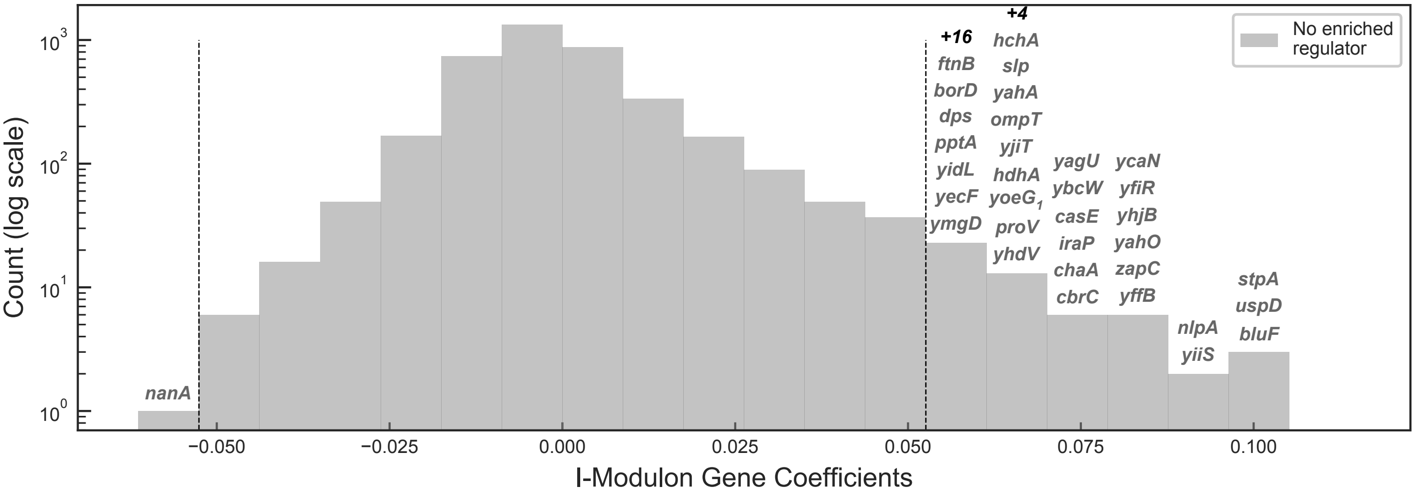

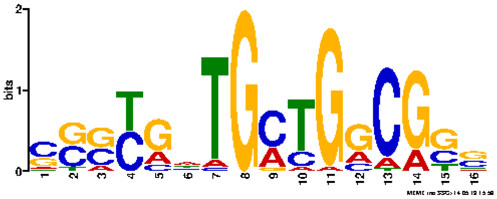

Motif E-value: 1.30e-05
Operons with Upstream Motif: 53%
