# Supplementary material for: The Escherichia coli transcriptome mostly consists of independently regulated modules
Source: Nat Commun. 2019 Dec 4;10:5536. doi: 10.1038/s41467-019-13483-w (PMC6892915; doi:10.1038/s41467-019-13483-w)
Supplement: Supplementary file 6 — Reporting Summary [file 41467_2019_13483_MOESM6_ESM.pdf]

nature research

Corresponding author(s): Bernhard Palsson

Last updated by author(s): Oct 31, 2019

Reporting Summary

Nature Research wishes to improve the reproducibility of the work that we publish. This form provides structure for consistency and transparency in reporting. For further information on Nature Research policies, see Authors & Referees and the Editorial Policy Checklist.

Statistics

For all statistical analyses, confirm that the following items are present in the figure legend, table legend, main text, or Methods section.

n/a	Confirmed	
☐	☒	The exact sample size (n) for each experimental group/condition, given as a discrete number and unit of measurement
☐	☒	A statement on whether measurements were taken from distinct samples or whether the same sample was measured repeatedly
☐	☒	The statistical test(s) used AND whether they are one- or two-sided<br>*Only common tests should be described solely by name; describe more complex techniques in the Methods section.*
☐	☒	A description of all covariates tested
☐	☒	A description of any assumptions or corrections, such as tests of normality and adjustment for multiple comparisons
☐	☒	A full description of the statistical parameters including central tendency (e.g. means) or other basic estimates (e.g. regression coefficient) AND variation (e.g. standard deviation) or associated estimates of uncertainty (e.g. confidence intervals)
☐	☒	For null hypothesis testing, the test statistic (e.g. F, t, r) with confidence intervals, effect sizes, degrees of freedom and P value noted<br>*Give P values as exact values whenever suitable.*
☐	☒	For Bayesian analysis, information on the choice of priors and Markov chain Monte Carlo settings
☐	☒	For hierarchical and complex designs, identification of the appropriate level for tests and full reporting of outcomes
☐	☒	Estimates of effect sizes (e.g. Cohen's d, Pearson's r), indicating how they were calculated

Our web collection on statistics for biologists contains articles on many of the points above.

Software and code

Policy information about availability of computer code

Data collection	RNA-seq collection: sra-toolkit v2.9.2, TRN gold standard: RegulonDB 10.0
Data analysis	RNA-seq Processing: bowtie (1.1.2), GenomicAlignments R package (1.18.0), DESeq2 R package (1.22.1)<br>ChIP-exo Processing: bowtie 1.1.2, MACE 1.0, Metascope (https://sites.google.com/view/systemskimlab/software?authuser=0)<br>Other software: R 3.5.3, python 2.7.12, Scikit-learn python package (0.19.2), GraphViz python package (0.9), SciPy python package (1.1.0), affy R package (1.50), elasticnet (1.1.1)

For manuscripts utilizing custom algorithms or software that are central to the research but not yet described in published literature, software must be made available to editors/reviewers. We strongly encourage code deposition in a community repository (e.g. GitHub). See the Nature Research guidelines for submitting code & software for further information.

Data

Policy information about availability of data

All manuscripts must include a data availability statement. This statement should provide the following information, where applicable:

- Accession codes, unique identifiers, or web links for publicly available datasets
- A list of figures that have associated raw data
- A description of any restrictions on data availability

The datasets analysed during the current study are available in the NCBI GEO repository under accession numbers GSE122211, GSE122295, GSE122296, and GSE122320. Additional datasets analysed during the current study are available in the NCBI GEO repository as described in Supplementary Data 1.

Field-specific reporting

Please select the one below that is the best fit for your research. If you are not sure, read the appropriate sections before making your selection.

☒ Life sciences ☐ Behavioural & social sciences ☐ Ecological, evolutionary & environmental sciences

For a reference copy of the document with all sections, see nature.com/documents/nr-reporting-summary-flat.pdf

Life sciences study design

All studies must disclose on these points even when the disclosure is negative.

Sample size	No sample size calculation was performed as high correlation was consistently observed between biological duplicates
Data exclusions	Biological replicates with R2 < 0.9 between log-TPM were removed from PRECISE to reduce noise
Replication	All RNA-seq and ChIP-exo samples were performed in biological duplicates
Randomization	This is not relevant to the study as the dataset was analyzed as a whole, rather than in subgroups.
Blinding	Blinding was not relevant to the study

Reporting for specific materials, systems and methods

We require information from authors about some types of materials, experimental systems and methods used in many studies. Here, indicate whether each material, system or method listed is relevant to your study. If you are not sure if a list item applies to your research, read the appropriate section before selecting a response.

Materials & experimental systems

n/a	Involved in the study	
☐	☒	Antibodies
☒	☐	Eukaryotic cell lines
☒	☐	Palaeontology
☐	☒	Animals and other organisms
☒	☐	Human research participants
☒	☐	Clinical data

Methods

n/a	Involved in the study	
☐	☒	ChIP-seq
☒	☐	Flow cytometry
☒	☐	MRI-based neuroimaging

Antibodies

Antibodies used	myc tag recognizing antibodies (9E10, Santa Cruz Biotechnology, Catalog# sc-40)
Validation	Validation was performed in:<br>PMID: # 27126587 Petsalaki, E. et al. 20163. Nature communications. 7: 11451.<br>PMID: # 30723194 Watanabe, S. et al. 2019. Nat Commun. 10: 603.<br>PMID: # 30804394 Georges, A. et al. 2019. Sci Rep. 9: 2724.

Animals and other organisms

Policy information about studies involving animals; ARRIVE guidelines recommended for reporting animal research

Laboratory animals	The following Escherichia coli strains were used in the study: K-12 MG1655, BW25113, W3110, Crooks, BL21(DE3), HS, O157:H7 EDL933, and CFT073
Wild animals	*Provide details on animals observed in or captured in the field; report species, sex and age where possible. Describe how animals were caught and transported and what happened to captive animals after the study (if killed, explain why and describe method; if released, say where and when) OR state that the study did not involve wild animals.*
Field-collected samples	*For laboratory work with field-collected samples, describe all relevant parameters such as housing, maintenance, temperature, photoperiod and end-of-experiment protocol OR state that the study did not involve samples collected from the field.*
Ethics oversight	*Identify the organization(s) that approved or provided guidance on the study protocol, OR state that no ethical approval or guidance was required and explain why not.*

Note that full information on the approval of the study protocol must also be provided in the manuscript.

ChIP-seq

Data deposition

☒ Confirm that both raw and final processed data have been deposited in a public database such as GEO.

☒ Confirm that you have deposited or provided access to graph files (e.g. BED files) for the called peaks.

Data access links<br>*May remain private before publication.*	GSE122320 (reviewer token: urojwussdxmtruh).
Files in database submission	cysB_taur_1_ChIPexo<br>cysB_taur_2_ChIPexo<br>metJ_meio_1_ChIPexo<br>metJ_meio_2_ChIPexo
Genome browser session<br>(e.g. UCSC)	N/A

Methodology

Replicates	Two biological replicates were performed from distinct samples
Sequencing depth	cysB_taur_1_ChIPexo R1: Single end, Total Reads=6089418, Uniquely mapped reads=5868665,length=101<br>cysB_taur_1_ChIPexo R2: Single end, Total Reads=6089418, Uniquely mapped reads=5554852,length=101<br>cysB_taur_2_ChIPexo R1: Single end, Total Reads=4693115, Uniquely mapped reads=4532052,length=101<br>cysB_taur_2_ChIPexo R2: Single end, Total Reads=4693115, Uniquely mapped reads=4243749,length=101<br>metJ_meio_1_ChIPexo R1: Single end, Total Reads=6079071, Uniquely mapped reads=6013264,length=101<br>metJ_meio_1_ChIPexo R2: Single end, Total Reads=6079071, Uniquely mapped reads=5521307,length=101<br>metJ_meio_2_ChIPexo R1: Single end, Total Reads=5406770, Uniquely mapped reads=5349381,length=101<br>metJ_meio_2_ChIPexo R2: Single end, Total Reads=5406770, Uniquely mapped reads=4956118,length=101
Antibodies	c-Myc Antibody from Santa cruz biotechnology (9E10)
Peak calling parameters	-- Sequence reads generated from ChIP-exo were mapped onto the reference genome (NC_000913.2) using bowtie with default options to generate SAM output files<br><br>-- MACE program was used to define peak candidates from biological duplicates for each experimental condition with sequence depth normalization by using MACE_MACEOPT ='-m 50'. To reduce false-positive peaks, we set up filter threshold='0.95'. The noise level was set to the top 5% of signals at genomic positions because top 5% resembles background level in plateau and top 5% intensities from each ChIP-exo replicates across conditions correlate well with the total number of reads. The calculation of S/N ratio resembles the way to calculate ChIP-chip peak intensity where IP signal was divided by Mock signal. Then, each peak was assigned to the nearest gene.
Data quality	The method to generate the data has been published in the Nucleic acid research journal: https://doi.org/10.1093/nar/gku846. This manuscript describes the effectiveness and robustness of the algorithm used in the method.
Software	Read mapping was performed using bowtie v1.1.2. MACE v1.0 was used to define peak candidates.
